# Dihydroartemisinin–piperaquine plus sulfadoxine–pyrimethamine versus either drug alone for intermittent preventive treatment of malaria in pregnancy: A double-blind, randomized, controlled phase 3 trial from Uganda

**DOI:** 10.1371/journal.pmed.1004582

**Published:** 2025-09-18

**Authors:** Abel Kakuru, Jimmy Kizza, Miriam Aguti, Harriet Adrama, John Ategeka, Peter Olwoch, Miriam Nakalembe, Joaniter I. Nankabirwa, Bishop Opira, Nida Ozarslan, Anju Ranjit, Erin dela Cruz, Tamara D. Clark, Michelle E. Roh, Stephanie L. Gaw, Prasanna Jagannathan, Philip J. Rosenthal, Moses R. Kamya, Grant Dorsey

**Affiliations:** 1 Infectious Diseases Research Collaboration, Kampala, Uganda; 2 Department of Community and Public Health, Busitema University, Tororo, Uganda; 3 School of Medicine, Makerere University, Kampala, Uganda; 4 Department of Obstetrics, Gynecology, and Reproductive Sciences, University of California, San Francisco, California, United States of America; 5 Department of Medicine, University of California, San Francisco, California, United States of America; 6 Institute for Global Health Sciences, University of California, San Francisco, California, United States of America; 7 Department of Medicine, Stanford University, Stanford, California, United States of America; Lao-Oxford-Mahosot Hospital-Wellcome Trust Research Unit, LAO PEOPLE'S DEMOCRATIC REPUBLIC

## Abstract

**Background:**

To mitigate adverse consequences of malaria in pregnancy, the World Health Organization recommends intermittent preventive treatment of malaria in pregnancy (IPTp) with sulfadoxine–pyrimethamine. However, the effectiveness of IPTp with sulfadoxine–pyrimethamine has been threatened by widespread *Plasmodium falciparum* resistance, especially in East and Southern Africa. For IPTp, dihydroartemisinin–piperaquine has shown superior antimalarial effects compared to sulfadoxine–pyrimethamine, but sulfadoxine–pyrimethamine has been associated with improved birth outcomes compared to dihydroartemisinin–piperaquine. We hypothesized that a combination of both dihydroartemisinin–piperaquine and sulfadoxine–pyrimethamine would provide superior birth outcomes compared to either drug alone.

**Methods and findings:**

We conducted a double-blinded, randomized, controlled trial of 2,757 pregnant women in Uganda, where resistance of malaria parasites to sulfadoxine–pyrimethamine is widespread. Women were randomly assigned (1:1:1) to monthly IPTp with sulfadoxine–pyrimethamine, dihydroartemisinin–piperaquine, or dihydroartemisinin–piperaquine plus sulfadoxine–pyrimethamine. The primary outcome was the risk of a composite adverse birth outcome defined as any of the following: spontaneous abortion, stillbirth, low birthweight (LBW, < 2,500 g), preterm delivery (<37 weeks), small-for-gestational age, or neonatal death. Secondary outcomes included specific individual adverse birth outcomes, measures of malaria during pregnancy, and safety/tolerability. Combining dihydroartemisinin–piperaquine plus sulfadoxine–pyrimethamine did not reduce the risk of a composite adverse birth outcome compared to dihydroartemisinin–piperaquine (30.0% versus 30.9%, relative risk (RR) 0.97 [95% CI 0.84–1.12]; *p* = 0.70) or sulfadoxine–pyrimethamine (30.0% versus 26.4%, RR 1.14 [95% CI 0.98–1.33]; *p* = 0.10). The risk of a composite adverse birth outcome was higher with dihydroartemisinin–piperaquine compared to sulfadoxine–pyrimethamine (30.9% versus 26.4%, RR 1.17 [95% CI 1.01–1.36]; *p* = 0.04). Considering individual adverse birth outcomes, combining dihydroartemisinin–piperaquine plus sulfadoxine–pyrimethamine was associated with a higher risk of small-for-gestational age (23.4% versus 18.7%, RR 1.25 [95% CI 1.04–1.51]; *p* = 0.02) and low birthweight (8.6% versus 5.8%, RR 1.48 [95 CI 1.04–2.12]; *p* = 0.03) compared to sulfadoxine–pyrimethamine and a higher risk of preterm delivery (5.3% versus 3.1%, RR 1.73 [95% CI 1.07–2.79]; *p* = 0.03) compared to dihydroartemisinin–piperaquine. During pregnancy, compared to sulfadoxine–pyrimethamine, dihydroartemisinin–piperaquine was associated with a 94% reduction in the incidence of symptomatic malaria (0.46 versus 0.03 episodes per person-year, incidence rate ratio 0.06 [95% CI 0.03–0.12]; *p* < 0.001) and a 97% reduction in the risk of microscopic parasitemia (17.7% versus 0.6%, RR 0.03 [95% CI 0.02–0.05]; *p* < 0.001), but dihydroartemisinin–piperaquine plus sulfadoxine–pyrimethamine was not associated with improved malaria outcomes over dihydroartemisinin–piperaquine alone. There were no significant differences in the incidence of any grade 3–4 adverse events between the treatment arms. As this study was conducted in an area of high transmission intensity with widespread resistance to sulfadoxine–pyrimethamine, findings may not be generalizable to other settings.

**Conclusions:**

Despite the superior antimalarial activity of dihydroartemisinin–piperaquine, sulfadoxine–pyrimethamine alone was associated with improved birth outcomes. Combining dihydroartemisinin–piperaquine plus sulfadoxine–pyrimethamine for IPTp did not improve birth outcomes compared to either sulfadoxine–pyrimethamine or dihydroartemisinin–piperaquine alone.

**Trial registration:**

ClinicalTrials.gov (NCT04336189; https://clinicaltrials.gov/study/NCT04336189).

## Author summary

**Why was this study done?:**

- Intermittent preventive treatment of malaria in pregnancy (IPTp) with sulfadoxine–pyrimethamine is standard policy throughout Africa for reducing the burden of malaria in pregnant women and improving birth outcomes.
- The effectiveness of IPTp with sulfadoxine–pyrimethamine has been threatened by drug resistance, especially in East and southern Africa, leading to a search for alternative regimens.
- Dihydroartemisinin–piperaquine has been the most widely studied alternative for IPTp. In prior studies comparing dihydroartemisinin–piperaquine to sulfadoxine–pyrimethamine for IPTp, dihydroartemisinin–piperaquine was much better at preventing malaria but women who received sulfadoxine–pyrimethamine gave birth to larger babies, possibly due to “non-malarial” benefits of sulfadoxine–pyrimethamine.
- These findings provided the rationale for evaluating a combination IPTp regimen of dihydroartemisinin–piperaquine plus sulfadoxine–pyrimethamine, which could harness both the antimalarial and non-malarial benefits of these drugs.

**What did the researchers do and find?:**

- To our knowledge this is the first study to evaluate the combination of dihydroartemisinin–piperaquine plus sulfadoxine–pyrimethamine for IPTp compared to sulfadoxine–pyrimethamine alone (the current standard of care) or dihydroartemisinin–piperaquine alone.
- The study was done in a setting where the burden of malaria was very high and resistance to sulfadoxine–pyrimethamine widespread.
- We found that IPTp with dihydroartemisinin–piperaquine alone or combined with sulfadoxine–pyrimethamine was far superior to IPTp with sulfadoxine–pyrimethamine alone in preventing malaria and reducing the risk of anemia during pregnancy. However, IPTp with dihydroartemisinin–piperaquine plus sulfadoxine–pyrimethamine did not result in improved birth outcomes compared to either sulfadoxine–pyrimethamine alone or dihydroartemisinin–piperaquine alone.

**What do these findings mean?:**

- We recommend against IPTp with dihydroartemisinin–piperaquine plus sulfadoxine–pyrimethamine.
- This study was conducted in an area where the burden of malaria was very high and resistance to sulfadoxine–pyrimethamine widespread, therefore findings may not be generalizable to other settings.
- Further research is warranted to identify new interventions, such as alternative IPTp regimens, vaccines, or monoclonal antibodies to better prevent malaria in pregnancy, reduce the risk of adverse birth outcomes, and ultimately improve the health of pregnant women and their infants.

## Abstract 3

In a randomized clinical trial of two drug regimens for intermittent preventive treatment of malaria in pregnancy (IPTp), Abel Kakuru and colleagues report that, unexpectedly, combining the two regimens did not result in fewer adverse birth outcomes than either regimen alone. As the effectiveness of standard IPTp is jeopardized by resistance of the Plasmodium falciparum parasite, these findings underscore the need for continued efforts to identify new effective and safe antimalarial drug regimens for use during pregnancy.

## Introduction

There were an estimated 36 million pregnancies in African countries with moderate to high malaria transmission in 2023, of which 12.4 million included infection with malaria parasites [1]. Infection with *Plasmodium falciparum* during pregnancy is associated with adverse consequences for the mother and fetus, including symptomatic malaria, maternal anemia, fetal loss, preterm birth, low birthweight (LBW), and neonatal mortality [2]. To mitigate these risks, the World Health Organization has recommended intermittent preventive treatment of malaria in pregnancy (IPTp) with sulfadoxine–pyrimethamine since 1998. This strategy involves the administration of full malaria treatment courses at least one month apart to all at-risk pregnant women starting in the second trimester, and it is currently a component of national malaria policy in 35 African countries [1,3]. However, the effectiveness of IPTp with sulfadoxine–pyrimethamine has been threatened by widespread *P. falciparum* drug resistance, especially in East and Southern Africa, leading to a search for alternative regimens [4].

Dihydroartemisinin–piperaquine has been the most widely studied alternative for IPTp. In a recent meta-analysis of six randomized trials, IPTp with dihydroartemisinin–piperaquine was associated with markedly lower risks of both malaria during pregnancy and placental malaria compared to sulfadoxine–pyrimethamine [5]. However, the superior antimalarial activity of dihydroartemisinin–piperaquine did not translate into improved birth outcomes. Rather, compared to sulfadoxine–pyrimethamine, dihydroartemisinin–piperaquine was associated with lower birthweight and a higher risk of infants born small-for-gestational-age.

It remains unclear why IPTp with sulfadoxine–pyrimethamine has been associated with higher birthweight despite the far superior antimalarial activity of dihydroartemisinin–piperaquine. One possible explanation is that sulfadoxine–pyrimethamine, which has antimicrobial activity, improves birthweight through mechanisms independent of its antimalarial activity. This hypothesis is supported by causal mediation analyses showing that effects on birthweight were primarily via non-malarial mechanisms for sulfadoxine–pyrimethamine and antimalarial mechanisms for dihydroartemisinin–piperaquine [5,6].

We hypothesized that a combination regimen that offered benefits of both dihydroartemisinin–piperaquine and sulfadoxine–pyrimethamine would provide superior birth outcomes compared to either drug alone. The underlying premise for this hypothesis was that dihydroartemisinin–piperaquine would best reduce the risk of malaria-attributable adverse birth outcomes, while sulfadoxine–pyrimethamine would best reduce the risk of adverse birth outcomes attributed to non-malarial mechanisms. To test this hypothesis, we conducted a randomized controlled trial of monthly IPTp with dihydroartemisinin–piperaquine plus sulfadoxine–pyrimethamine compared with either regimen alone in an area of high malaria transmission intensity and widespread resistance to sulfadoxine–pyrimethamine.

## Methods

### Study design and setting

This was a three-arm, individually randomized, double-blind, controlled trial conducted in Busia District, southeastern Uganda, where malaria transmission is perennial and intense. The study was registered with ClinicalTrials.gov (NCT04336189 https://clinicaltrials.gov/) and was approved by the Makerere University School of Biomedical Sciences Research Ethics Committee (SBS 714), the Uganda National Council for Science and Technology (HS 2746), the Uganda National Drug Authority (CTC 0135/2020), and the University of California San Francisco Human Research Protection Program (19-29105). An independent data and safety monitoring board constituted by the study team in cooperation with the sponsor conducted annual reviews to assess study participants' safety and study progress.

### Participants

Study participants were HIV-uninfected women 16 years of age or older with a viable, singleton intrauterine pregnancy at 12–20 weeks gestational age confirmed by ultrasound. Study participants provided written informed consent, agreed to come to a dedicated study clinic for all routine medical care and avoid medication given outside the study clinic, and planned to deliver at a health facility. Women were excluded if they had a history of a serious adverse event from sulfadoxine–pyrimethamine or dihydroartemisinin–piperaquine, active labor, active medical problems requiring inpatient evaluation, chronic medical conditions, intention to move outside the study area during the study period, a history of long QT syndrome, or a history of taking SP or any antimalarial during this pregnancy.

### Randomization and masking

Women were randomly assigned to receive monthly IPTp with sulfadoxine–pyrimethamine, dihydroartemisinin–piperaquine, or dihydroartemisinin–piperaquine plus sulfadoxine–pyrimethamine Randomization was done in a 1:1:1 ratio using permuted blocks of 6 or 9. A computer-generated randomization list including consecutive treatment numbers with corresponding random treatment assignments was generated by a study investigator (GD) not involved with the conduct of the fieldwork. Prior to the onset of the study, a set of sequentially numbered, opaque, sealed envelopes containing treatment allocation numbers was prepared. Study pharmacists who were not otherwise involved in the trial were responsible for treatment assignment and preparation of study drugs. Placebos were used such that all participants received the same number of pills with the same appearance. Study participants, investigators, and study staff involved in the daily care of study participants and assessing study outcomes were blinded to the intervention assigned.

### Study procedures

At enrollment, participants received a long-lasting insecticidal net (LLIN), underwent a standardized examination, and had blood samples collected. Participants received all their medical care at a study clinic open every day. Each course of sulfadoxine–pyrimethamine consisted of three tablets (500 mg sulfadoxine and 25 mg pyrimethamine [Kamsidar, Kampala Pharmaceutical Industries]) as a single dose. Each course of dihydroartemisinin–piperaquine consisted of three tablets (40 mg dihydroartemisinin and 320 mg piperaquine [Duo-Cotecxin, Holley-Cotec]) once daily for three consecutive days. Study drugs were administered every 4 weeks, starting at 16 or 20 weeks gestation and continued to delivery, up to 40 weeks gestation. Placebos were used such that every 4 weeks participants received either active sulfadoxine–pyrimethamine plus placebo dihydroartemisinin–piperaquine, or placebo sulfadoxine–pyrimethamine plus active dihydroartemisinin–piperaquine, or active sulfadoxine–pyrimethamine plus active dihydroartemisinin–piperaquine. Administration of the first daily dose was directly observed in the clinic, with the second and third doses administered at home (S1 Fig). Adherence to study drugs administered at home was assessed by participant recall at their next routine visit.

Routine visits were scheduled every 4 weeks for administration of study drugs, a clinical examination, and a blood draw for detection of malaria parasites by microscopy and quantitative PCR (qPCR). Complete blood counts were performed at 20, 28, and 36 weeks gestation. Participants were encouraged to come to the clinic any time they required medical care. At any visit, participants with a history of fever in the past 24 h or a tympanic temperature ≥38.0°C had a thick blood smear assessed for malaria parasites. If the smear was positive, the participant was diagnosed with symptomatic malaria and treated with artemether-lumefantrine. Participants with asymptomatic parasitemia detected at the time of routine visits were not provided additional antimalarial therapy beyond their assigned IPTp drugs in accordance with local guidelines.

Study participants were encouraged to deliver at a health facility. Those who delivered at home were visited by study staff shortly after delivery and encouraged to come to the study clinic. At delivery, a standardized assessment was conducted, including birthweight, evaluation for congenital anomalies, and collection of placental tissue and maternal, placental, and cord blood. Participants were followed for 4 weeks postpartum. Adverse events were assessed and graded at every clinic visit according to standardized criteria [7]. For the first 300 participants enrolled, electrocardiograms were done at 20, 28, and 36 gestation weeks just before the first dose and 2–6 h after the third dose of study drugs.

Blood smears were stained with 2% Giemsa and considered negative when 100 high-power fields did not reveal asexual parasites. All slides were read by a second microscopist, and a third reviewer settled any discrepant readings. A highly sensitive *P. falciparum* qPCR assay with a lower limit of detection of 1 parasite/µL was performed on blood collected at enrollment, routine visits, and delivery [8].

### Outcomes

The primary outcome was the risk of a composite adverse birth outcome defined as any of the following: spontaneous abortion (fetal loss at <28 weeks gestational age), stillbirth (infants born deceased at ≥28 weeks gestational age), and among live births: LBW (< 2,500 g), preterm delivery (<37 weeks), small-for-gestational age (birthweight <10th percentile for gestational age by INTERGROWTH-21st standards) [9], or neonatal death (within the first 28 days of life). Secondary outcomes included individual components of the primary outcome, birthweight, gestational age at birth, birthweight-for-gestational age z-score [9], and gestational weight gain (maternal weight at the time of delivery—maternal weight at the time study drugs were initiated). Malaria-related outcomes measured following the initiation of study drugs included the incidence of symptomatic malaria; prevalence of parasitemia by microscopy or qPCR at routine visits; prevalence of any (Hb < 11 g/dL) or severe (Hb < 8 g/dL) anemia at 20, 28, or 36 gestational weeks; detection of malaria parasites by microscopy or qPCR at birth from maternal, placental, or cord blood; and measures of placental malaria by histopathology, including detection of parasites (active infection), malaria pigment in ≥30% of high-powered fields (high-grade past infection) [10], or any parasites or malaria pigment (any active or past infection). Measures of tolerability and safety included the incidence of vomiting, common adverse events of any severity, and serious adverse events following administration of study drugs.

### Statistical analysis

We assumed a risk of composite adverse birth outcome of 22.6% in the sulfadoxine–pyrimethamine arm and 25.1% in the dihydroartemisinin–piperaquine arm based on prior data [11]. A sample size of 2,757 (assuming 15% loss to follow-up) was required to achieve 80% power (two-sided alpha = 0.05) to detect a ≥25% reduction with dihydroartemisinin–piperaquine plus sulfadoxine–pyrimethamine compared to the other arms. Statistical analyses were conducted using Stata (version 18). All analyses used a modified intention-to-treat approach and included all participants with evaluable outcomes. Comparisons of the primary outcome and dichotomous secondary outcomes were made using log-binomial regression to obtain relative risks (RRs). Comparisons of dichotomous secondary outcomes with repeated measures (parasite prevalence and anemia during pregnancy) were performed using generalized estimating equations with a log-binomial model and robust standard errors. Comparisons of incidence measures were performed using negative binomial regression to obtain incidence rate ratios (IRRs). Comparisons of continuous outcomes were performed using linear regression to obtain mean differences. The number needed to treat to avert one event was calculated as the inverse of the incidence rate difference per 21.4 weeks (the average duration of follow-up following study drug initiation). Subgroup analyses by maternal age, gravidity, infant sex, and gestational age at the time study drugs were first administered were pre-specified, and differences were tested using two-way interaction terms between treatment arm and subgroups (*P*_interaction_). Only subgroup analyses stratified by gravidity demonstrated significant interaction and are reported. Statistical significance was defined as a two-sided *p-*value of <0.05 for hypothesis testing and <0.1 for interaction terms.

## Results

### Study participants and follow-up

**Fig 1:**
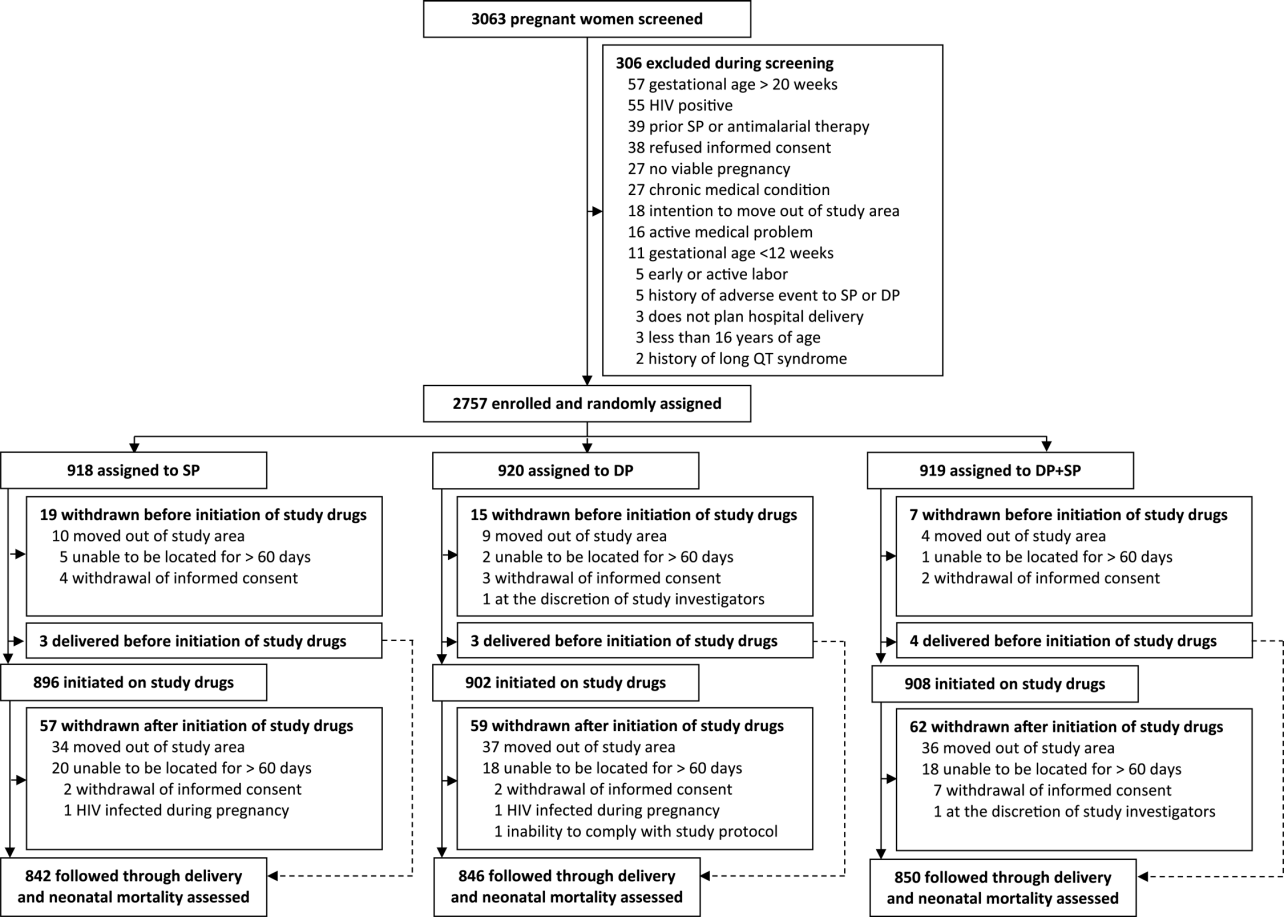
Trial profile.

Between December 28, 2020, and December 18, 2023, 3,063 women were screened, of whom 2,757 were enrolled and underwent randomization (Fig 1). The first participant was enrolled on December 28, 2020, and the last on December 18, 2023. Baseline characteristics were similar between treatment arms (Table 1). The mean age at enrollment was 24.5 years (standard deviation 6.2 years), 1,533 (55.6%) of 2,757 participants were enrolled between 12 and 16 gestational weeks, 726 (26.3%) were primigravidae, and 1,360 (49.3%) reported owning an LLIN at the time of enrollment (all participants were given an LLIN after enrollment). Parasite prevalence was 38.0% (1047/2757) by microscopy and 70.3% (1937/2757) by microscopy or qPCR. Of those with microscopic parasitemia, 1.9% (20/1047) were febrile and treated with artemether-lumefantrine. In a random subset of 200 enrollment samples with parasite densities >100/µL, 198 (99%) had five *P. falciparum* dihydrofolate reductase (PfDHFR; N51I, C59R, and S108N) and dihydropteroate synthetase (PfDHPS; A437G and K540E) mutations associated with resistance to sulfadoxine–pyrimethamine and known to be common in Uganda [12]. Considering mutations associated with higher-level resistance, 41 (20.5%) of 200 had the PfDHFR I164L and four (2%) had the PfDHPS A581G mutation. A total of 2,706 (98.2%) participants enrolled received at least one dose of study drugs, and 2,538 (92.1%) were followed through delivery (Fig 1).

**Table 1:** Baseline characteristics of enrolled study participants.

Characteristic	Treatment arm		
	SP^1^ (*n* = 918)	DP^1^ (*n* = 920)	DP + SP^1^ (*n* = 919)
Age in years, mean (SD)	24.5 (6.2)	24.7 (6.3)	24.2 (6.0)
Gestational age in weeks, mean (SD)	15.8 (2.6)	15.7 (2.5)	16.0 (2.6)
Gestational age categories, *n* (%)			
12–16 weeks	522 (56.9%)	530 (57.6%)	481 (52.3%)
>16–20 weeks	396 (43.1%)	390 (42.4%)	438 (47.7%)
Gravidity, *n* (%)			
Primigravidae	230 (25.1%)	234 (25.4%)	262 (28.5%)
Multigravidae	688 (74.9%)	686 (74.6%)	657 (71.5%)
Bednet ownership, *n* (%)			
None	423 (46.1%)	434 (47.2%)	415 (45.2%)
Untreated net	43 (4.7%)	40 (4.4%)	42 (4.6%)
Long-lasting insecticide-treated net	452 (49.2%)	446 (48.5%)	462 (50.3%)
Household wealth index, *n* (%)			
Lowest tertile	308 (33.9%)	294 (32.3%)	295 (32.4%)
Middle tertile	293 (32.3%)	319 (35.1%)	309 (33.9%)
Highest tertile	307 (33.8%)	296 (32.6%)	307 (33.7%)
Highest level of education, *n* (%)			
None	44 (4.8%)	31 (3.4%)	36 (3.9%)
Primary school	592 (64.5%)	593 (64.5%)	600 (65.3%)
Secondary school	263 (28.7%)	276 (30.0%)	264 (28.7%)
Higher	19 (2.1%)	20 (2.2%)	19 (2.1%)
Weight in kg, mean (SD)	57.3 (9.4)	57.6 (9.8)	57.1 (8.9)
Height in cm, mean (SD)	159 (6)	159 (7)	159 (6)
Maternal MUAC, mean (SD)	26 (4)	26 (3)	26 (3)
Mean hemoglobin g/dL (SD)	11.6 (1.3)	11.7 (1.3)	11.5 (1.4)
Detection of malaria parasites by microscopy, *n* (%)	332 (36.2%)	347 (37.7%)	368 (40.0%)
Detection of malaria parasites by microscopy or qPCR, *n* (%)	643 (70.1%)	637 (69.2%)	657 (71.5%)
Prevalence of markers of antifolate resistance, *n* (%)^2^	*N* = 72	*N* = 65	*N* = 63
*dhfr/dhps quintuple mutant*^3^	72 (100%)	64 (98.5%)	62 (98.4%)
*dhfr* I164L	14 (19.4%)	19 (29.2%)	8 (12.7%)
*dhps A581G*	1 (1.4%)	2 (3.1%)	1 (1.6%)

^1^ SP, sulfadoxine–pyrimethamine; DP, dihydroartemisinin–piperaquine.

^2^ Random subset of 200 samples with parasite density >100/µL; mixed genotypes were categorized as mutant.

^3^* dhfr* N51I+C59R+S108N*; dhps* A437G+K540E.

### Efficacy outcomes

**Fig 2:**
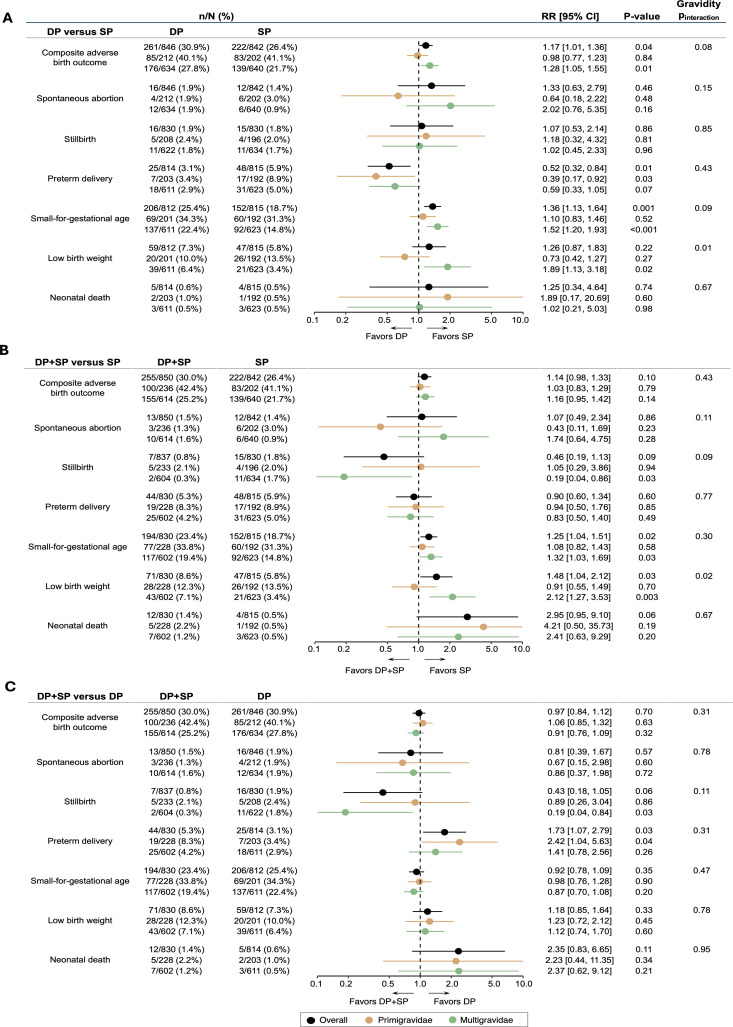
Primary endpoint and individual adverse birth outcomes, including stratification by gravidity. All pairwise comparisons included in sub-figures **A**–**C**. DP, dihydroartemisinin–piperaquine; SP, sulfadoxine–pyrimethamine; and RR, relative risk. *P*_interaction_ denotes *p*-values from two-way interaction terms evaluating differences in treatment effects between gravidity subgroups.

IPTp with dihydroartemisinin–piperaquine was associated with a higher risk of the composite adverse birth outcome compared to sulfadoxine–pyrimethamine (261 [30.9%] of 846 versus 222 [26.4%] of 842; RR 1.17 [95% CI 1.01–1.36], *p* = 0.04) (Fig 2A). Similarly, IPTp with dihydroartemisinin–piperaquine plus sulfadoxine–pyrimethamine was associated with a higher risk of the composite adverse birth outcome compared to sulfadoxine–pyrimethamine alone, although this difference did not reach statistical significance (255 [30.0%] of 850 versus 222 [26.4%] of 842; RR 1.14 [95% CI 0.98–1.33], *p* = 0.10) (Fig 2B). IPTp with dihydroartemisinin–piperaquine plus sulfadoxine–pyrimethamine was associated with a similar risk of the composite adverse birth outcome compared to dihydroartemisinin–piperaquine alone (255 [30.0%] of 850 versus 261 [30.9%] of 846; RR 0.97 [95% CI 0.84–1.12], *p* = 0.70) (Fig 2C). When stratified by gravidity, there was no evidence of interaction when comparing dihydroartemisinin–piperaquine plus sulfadoxine–pyrimethamine to sulfadoxine–pyrimethamine alone or dihydroartemisinin–piperaquine alone. When comparing dihydroartemisinin–piperaquine to sulfadoxine–pyrimethamine, the higher risk of a composite adverse birth outcome was present among multigravidae (176 [27.8%] of 634 versus 139 [21.7%] of 640, RR 1.28 [95% CI 1.05–1.55], *p* = 0.01) but not primigravidae (85 of [40.1%] of 212 versus 83 [41.1%] of 202; RR 0.98 [95% CI 0.77–1.23], *p* = 0.84). In summary, dihydroartemisinin–piperaquine was associated with a higher risk of the composite adverse birth outcome compared to sulfadoxine–pyrimethamine, but only among multigravidae. Combining the two regimens did not lower this risk compared to using either drug alone.

Considering individual adverse birth outcomes, IPTp with dihydroartemisinin–piperaquine was associated with a higher risk of small-for-gestational age (206 [25.4%] of 812 versus 152 [18.7%] of 815, RR 1.36 [95% CI 1.13–1.64], *p* = 0.001) but a lower risk of preterm birth (25 [3.1%] of 814 versus 48 [5.9%] of 815, RR 0.52 [95% CI 0.32–0.84], *p* = 0.01) compared to sulfadoxine–pyrimethamine (Fig 2A). The combination of dihydroartemisinin–piperaquine plus sulfadoxine–pyrimethamine was associated with a higher risk of small-for-gestational age (194 [23.4%] of 830 versus 152 [18.7%] of 815, RR 1.25 [95% CI 1.104–1.51], *p* = 0.02) and LBW (71 [8.6%] of 830 versus 47 [5.8%] of 815, RR 1.48 [95% CI 1.04–2.12], *p* = 0.03) compared to sulfadoxine–pyrimethamine alone and a higher risk of preterm delivery (44 [5.3%] of 830 versus 25 [3.1%] of 814, RR 1.73 [95% CI 1.07–2.79], *p* = 0.03) compared to dihydroartemisinin–piperaquine alone (Fig 2B and 2C). When stratified by gravidity, dihydroartemisinin–piperaquine was associated with a significantly higher risk of small-for-gestational age and LBW compared to sulfadoxine–pyrimethamine only among multigravidae (Fig 2A). Similarly, the combination of dihydroartemisinin–piperaquine plus sulfadoxine–pyrimethamine was associated with a significantly higher risk of small-for-gestational age and LBW compared to sulfadoxine–pyrimethamine only among multigravidae women (Fig 2B). There was no interaction by gravidity when comparing individual adverse birth outcomes between the combination of dihydroartemisinin–piperaquine plus sulfadoxine–pyrimethamine and dihydroartemisinin–piperaquine alone (Fig 2C).

**Fig 3:**
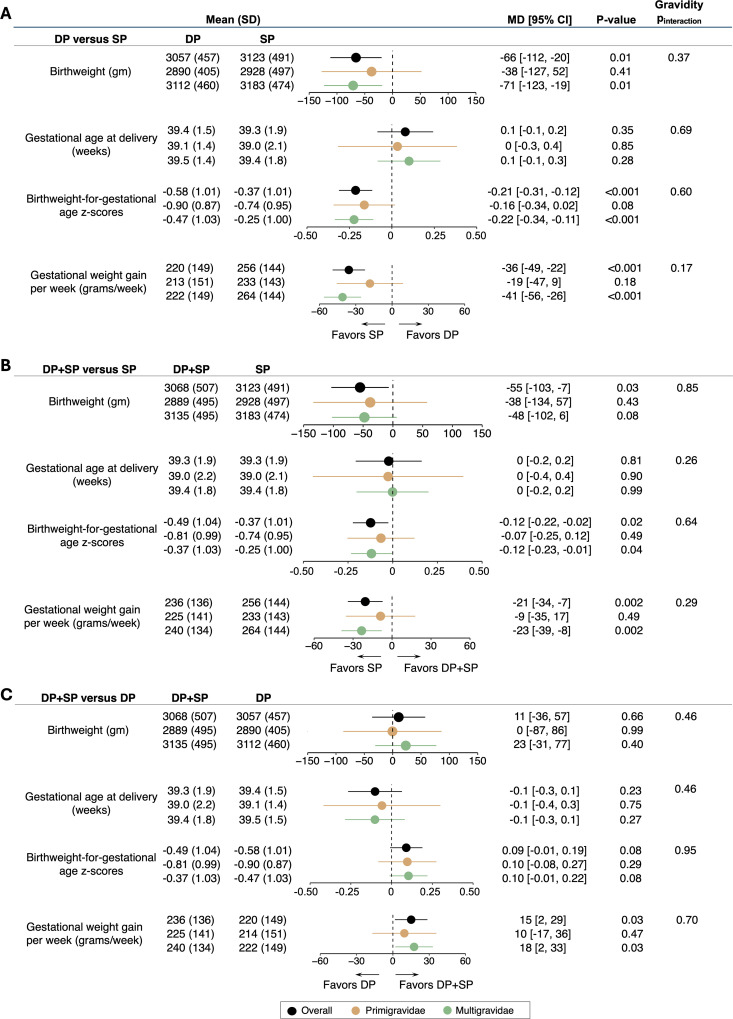
Continuous maternal and birth outcomes, including stratification by gravidity. All pairwise comparisons included in sub-figures **A**–**C**. DP, dihydroartemisinin–piperaquine; SP, sulfadoxine–pyrimethamine; and MD, mean difference. *P*_interaction_ denotes *p*-values from two-way interaction terms evaluating differences in treatment effects between gravidity subgroups.

Considering continuous birth outcomes, dihydroartemisinin–piperaquine was associated with a lower mean birthweight (3,057 versus 3,123 g; MD −66 [95% CI −112, −20], *p* = 0.01), lower birthweight-for-gestational age *z*-scores (−0.58 versus −0.37; MD −0.21 [95% CI −0.31, −0.12], *p* < 0.001), and lower gestational weight gain (220 versus 256 g/week; MD −36 [95% CI −49, −22], *p* < 0.001) compared to sulfadoxine–pyrimethamine (Fig 3A). Similarly, dihydroartemisinin–piperaquine plus sulfadoxine–pyrimethamine was associated with a lower mean birthweight (3,068 versus 3,123 g; MD −55 [95% CI −103, −7], *p* = 0.03), lower birthweight-for-gestational age *z*-scores (−0.49 versus −0.37; MD −0.12 [95% CI −0.22, −0.02], *p* = 0.02), and lower gestational weight gain (236 versus 256 g/week; MD −21 [95% CI −34, −7], *p* = 0.002) compared to sulfadoxine–pyrimethamine alone (Fig 3B). There were no significant differences in gestational age at delivery between any of the treatment arms or other continuous outcomes when comparing dihydroartemisinin–piperaquine plus sulfadoxine–pyrimethamine to dihydroartemisinin–piperaquine alone with the exception of higher gestational weight gain (236 versus 220 g/week; MD 15 [95% CI 2, 29], *p* = 0.03) in the dihydroartemisinin–piperaquine plus sulfadoxine–pyrimethamine arm (Fig 3C). There was no interaction by gravidity when comparing continuous birth outcomes across any of the three IPTp regimens.

During pregnancy, dihydroartemisinin–piperaquine was associated with a 94% reduction in the incidence of symptomatic malaria (0.03 versus 0.46 episodes per person year; IRR 0.06 [95% CI 0.03–0.12], *p* < 0.0001), a 97% reduction in the risk of microscopic parasitemia (0.6% versus 17.7%; RR 0.03 [95% CI 0.02–0.05], *p* < 0.0001), a 15% reduction in the risk of any anemia (36.7% versus 43.2%; RR 0.85 [95% CI 0.77–0.94], *p* = 0.0012), and marked reductions in risks of various measures of malaria at delivery compared to sulfadoxine–pyrimethamine (Table 2). As expected, the burden of malaria was higher in primigravidae compared to multigravidae, resulting in greater absolute malaria-related benefits of dihydroartemisinin–piperaquine over sulfadoxine–pyrimethamine among primigravidae compared to multigravidae (Table 3). For example, the number needed to treat with dihydroartemisinin–piperaquine versus sulfadoxine–pyrimethamine to avert one episode of symptomatic malaria was approximately 2 among primigravidae compared to 11 among multigravidae. Similarly, dihydroartemisinin–piperaquine plus sulfadoxine–pyrimethamine was associated with a 95% reduction in the incidence of symptomatic malaria (0.02 versus 0.46 episodes per person year; IRR 0.05 [95% CI 0.03–0.10], *p* < 0.0001), a 92% reduction in the risk of microscopic parasitemia (1.4% versus 17.7%; RR 0.08 [95% CI 0.06–0.10], *p* < 0.0001), a 10% reduction in the risk of any anemia (39.5% versus 43.2%; RR 0.90 [95% CI 0.82–0.99], *p* = 0.034), and marked reductions in risks of various measures of malaria at delivery compared to sulfadoxine–pyrimethamine alone (Table 2). Compared to dihydroartemisinin–piperaquine alone, dihydroartemisinin–piperaquine plus sulfadoxine–pyrimethamine did not improve malaria-related outcomes and was associated with higher risks of parasitemia (1.4% versus 0.6%; RR 2.35 [95% CI 1.34–0.10], *p* = 0.0026) during pregnancy and any evidence of placental malaria by histopathology (50.1% versus 40.8%; RR 1.23 [95% CI 1.10–1.37], *p* = 0.0002) (Table 2). In summary, dihydroartemisinin–piperaquine was markedly superior to sulfadoxine–pyrimethamine in regard to malaria outcomes during pregnancy, but combining the two regimens did not offer benefits against malaria outcomes compared to dihydroartemisinin–piperaquine alone.

**Table 2:** Secondary efficacy outcomes.

Outcome	SP^1^	DP^1^	DP + SP^1^	DP vs. SP^1^		DP + SP vs. SP^1^		DP + SP vs. DP^1^	
				RR^2^ (95% CI)	*p*-value	RR^2^ (95% CI)	*p*-value	RR^2^ (95% CI)	*p*-value
**Outcomes measured during pregnancy**									
Symptomatic malaria^3^	170 (0.46)	11 (0.03)	9 (0.02)	0.06 (0.03–0.12)	<0.001	0.05 (0.03–0.10)	<0.001	0.81 (0.32–2.04)	0.66
Microscopic parasitemia^4^	771/4350 (17.7%)	25/4348 (0.6%)	60/4354 (1.4%)	0.03 (0.02–0.05)	<0.001	0.08 (0.06–0.10)	<0.001	2.35 (1.34–4.09)	0.003
Microscopic or sub-microscopic parasitemia^4^	2099/4350 (48.3%)	551/4348 (12.7%)	716/4354 (16.4%)	0.27 (0.24–0.29)	<0.001	0.34 (0.31–0.38)	<0.001	1.29 (1.15–1.45)	<0.001
Any anemia (Hb < 11 g/dL)^5^	965/2232 (43.2%)	827/2252 (36.7%)	880/2229 (39.5%)	0.85 (0.77–0.94)	0.001	0.90 (0.82–0.99)	0.03	1.06 (0.96–1.17)	0.28
Severe anemia (Hb < 8 g/dL)^5^	29/2232 (1.3%)	9/2252 (0.4%)	12/2229 (0.5%)	0.28 (0.10–0.77)	0.01	0.40 (0.17–0.91)	0.03	1.38 (0.43–4.36)	0.59
**Outcomes measured at delivery**									
Maternal blood parasitemia by microscopy	47/839 (5.6%)	4/842 (0.5%)	5/848 (0.6%)	0.08 (0.03–0.23)	<0.001	0.11 (0.04–0.26)	<0.001	1.24 (0.33–4.61)	0.75
Maternal blood parasitemia by qPCR	297/828 (35.9%)	57/827 (6.9%)	77/839 (9.2%)	0.19 (0.15–0.25)	<0.001	0.26 (0.20–0.32)	<0.001	1.33 (0.96–1.85)	0.09
Placental blood parasitemia by microscopy	57/782 (7.3%)	4/794 (0.5%)	7/798 (0.9%)	0.07 (0.03–0.19)	<0.001	0.12 (0.06–0.26)	<0.001	1.74 (0.51–5.92)	0.38
Placental blood parasitemia by qPCR	201/773 (26.0%)	40/786 (5.1%)	44/790 (5.6%)	0.20 (0.14–0.27)	<0.001	0.21 (0.16–0.29)	<0.001	1.09 (0.72–1.66)	0.67
Cord blood parasitemia by microscopy	2/766 (0.3%)	0/773 (0%)	0/780 (0%)	NA		NA		NA	
Cord blood parasitemia by qPCR	29/755 (3.8%)	7/761 (0.9%)	9/765 (1.2%)	0.24 (0.11–0.54)	0.001	0.31 (0.15–0.64)	0.002	1.28 (0.48–3.42)	0.62
Parasites detected by histopathology	60/776 (7.7%)	8/795 (1.0%)	8/784 (1.0%)	0.13 (0.06–0.27)	<0.001	0.13 (0.06–0.27)	<0.001	1.01 (0.38–2.69)	0.98
High-grade pigment detected by histopathology	82/776 (10.6%)	8/795 (1.0%)	13/784 (1.7%)	0.10 (0.05–0.20)	<0.001	0.16 (0.09–0.28)	<0.001	1.65 (0.69–3.95)	0.26
Any parasites or pigment detected by histopathology	472/776 (60.8%)	324/795 (40.8%)	393/784 (50.1%)	0.67 (0.61–0.74)	<0.001	0.82 (0.75–0.90)	<0.001	1.23 (1.10–1.37)	<0.001

^1^ SP, sulfadoxine–pyrimethamine; DP, dihydroartemisinin–piperaquine.

^2^ Relative risk for all outcomes with the exception of symptomatic malaria, where the incidence rate ratio was used.

^3^ Episodes of symptomatic malaria (incidence per person-year at risk following initiation of study drugs).

^4^ Measured at the time of routine visits conducted every 4 weeks following initiation of study drugs.

^5^ Hemoglobin measured at 20, 28, and 36 gestational weeks following initiation of study drugs.

**Table 3:** Secondary efficacy outcomes stratified by gravidity.

Outcome	SP^1^	DP^1^	DP + SP^1^	DP vs. SP^1^		DP + SP vs. SP^1^		DP + SP vs. DP^1^	
				RR^2^ (95% CI)	*p*-value	RR^2^ (95% CI)	*p*-value	RR^2^ (95% CI)	*p*-value
**Primigravidae**									
Symptomatic malaria^3^	99 (1.15)	2 (0.02)	4 (0.04)	0.02 (0.005–0.08)	<0.001	0.03 (0.01–0.09)	<0.001	1.79 (0.26–12.4)	0.56
Microscopic parasitemia^4^	360/1004 (35.9%)	8/1044 (0.8%)	21/1179 (1.8%)	0.02 (0.01–0.04)	<0.001	0.05 (0.03–0.08)	<0.001	2.35 (0.97–5.68)	0.06
Microscopic or sub-microscopic parasitemia^4^	641/1004 (63.8%)	161/1044 (15.4%)	236/1179 (20.0%)	0.25 (0.21–0.29)	<0.001	0.32 (0.27–0.37)	<0.001	1.30 (1.06–1.59)	0.01
Any anemia (Hb < 11 g/dL)^5^	300/511 (58.7%)	227/542 (41.9%)	255/596 (42.8%)	0.70 (0.60–0.82)	<0.001	0.73 (0.62–0.85)	<0.001	1.04 (0.87–1.25)	0.66
Severe anemia (Hb < 8 g/dL)^5^	14/511 (2.7%)	0/542 (0%)	3/596 (0.5%)	NA		0.16 (0.03–0.74)	0.02	NA	
Maternal blood parasitemia by microscopy	23/201 (11.4%)	1/212 (0.5%)	3/236 (1.3%)	0.04 (0.01–0.30)	0.002	0.11 (0.03–0.36)	<0.001	2.69 (0.28–25.7)	0.39
Maternal blood parasitemia by qPCR	103/198 (52.0%)	14/210 (6.7%)	24/233 (10.3%)	0.13 (0.08–0.22)	<0.001	0.20 (0.13–0.30)	<0.001	1.55 (0.82–2.91)	0.18
Placental blood parasitemia by microscopy	29/183 (15.9%)	1/194 (0.5%)	4/220 (1.8%)	0.03 (0.004–0.24)	<0.001	0.11 (0.04–0.32)	<0.001	3.53 (0.40–31.3)	0.26
Placental blood parasitemia by qPCR	75/182 (41.2%)	7/194 (3.6%)	15/222 (6.8%)	0.09 (0.04–0.18)	<0.001	0.16 (0.10–0.28)	<0.001	1.87 (0.78–4.50)	0.16
Cord blood parasitemia by microscopy	1/181 (0.6%)	0/188 (0%)	0/215 (0%)	NA		NA		NA	
Cord blood parasitemia by qPCR	10/177 (5.7%)	1/186 (0.5%)	5/211 (2.4%)	0.10 (0.01–0.74)	0.02	0.42 (0.15–1.20)	0.11	4.41 (0.52–37.4)	0.17
Parasites detected by histopathology	33/177 (18.6%)	2/196 (1.0%)	5/218 (2.3%)	0.05 (0.01–0.22)	<0.001	0.12 (0.05–0.31)	<0.001	2.25 (0.44–11.5)	0.33
High-grade pigment detected by histopathology	55/177 (31.1%)	7/196 (3.6%)	8/218 (3.7%)	0.11 (0.05–0.25)	<0.001	0.12 (0.06–0.24)	<0.001	1.03 (0.38–2.78)	0.96
Any parasites or pigment detected by histopathology	156/177 (88.1%)	145/196 (74.0%)	167/218 (76.6%)	0.84 (0.76–0.93)	<0.001	0.87 (0.79–0.93)	0.003	1.03 (0.93–1.16)	0.54
**Multigravidae**									
Symptomatic malaria^2^	71 (0.25)	9 (0.03)	5 (0.02)	0.13 (0.06–0.26)	<0.001	0.07 (0.03–0.18)	<0.001	0.58 (0.19–1.73)	0.33
Microscopic parasitemia^4^	411/3346 (12.3%)	17/3304 (0.5%)	39/3175 (1.2%)	0.04 (0.02–0.08)	<0.001	0.10 (0.07–0.15)	<0.001	2.28 (1.12–4.63)	0.02
Microscopic or sub-microscopic parasitemia^4^	1458/3346 (43.6%)	390/3304 (11.8%)	480/3175 (15.1%)	0.27 (0.24–0.31)	<0.001	0.35 (0.31–0.39)	<0.001	1.28 (1.11–1.47)	<0.001
Any anemia (Hb < 11 g/dL)^5^	665/1721 (38.6%)	600/1710 (35.1%)	625/1633 (38.3%)	0.93 (0.82–1.04)	0.20	0.98 (0.87–1.10)	0.74	1.06 (0.94–1.20)	0.35
Severe anemia (Hb < 8 g/dL)^5^	15/1721 (0.9%)	9/1710 (0.5%)	9/1633 (0.6%)	0.60 (0.19–1.87)	0.37	0.66 (0.22–1.93)	0.45	1.10 (0.32–3.73)	0.88
Maternal blood parasitemia by microscopy	24/638 (3.8%)	3/630 (0.5%)	2/612 (0.3%)	0.13 (0.04–0.42)	<0.001	0.09 (0.02–0.37)	<0.001	0.69 (0.12–4.09)	0.68
Maternal blood parasitemia by qPCR	194/630 (30.8%)	43/617 (7.0%)	53/606 (8.8%)	0.23 (0.17–0.31)	<0.001	0.28 (0.21–0.38)	<0.001	1.25 (0.85–1.85)	0.25
Placental blood parasitemia by microscopy	28/599 (4.7%)	3/600 (0.5%)	3/578 (0.5%)	0.11 (0.03–0.35)	<0.001	0.11 (0.03–0.36)	<0.001	1.04 (0.21–5.12)	0.96
Placental blood parasitemia by qPCR	126/591 (21.3%)	33/592 (5.6%)	29/568 (5.1%)	0.26 (0.18–0.38)	<0.001	0.24 (0.16–0.35)	<0.001	0.92 (0.56–1.49)	0.72
Cord blood parasitemia by microscopy	1/585 (0.2%)	0/585 (0%)	0/565 (0%)	NA		NA		NA	
Cord blood parasitemia by qPCR	19/578 (3.3%)	6/575 (1.0%)	4/554 (0.7%)	0.32 (0.13–0.79)	0.01	0.22 (0.08–0.64)	0.006	0.69 (0.20–2.44)	0.57
Parasites detected by histopathology	27/599 (4.5%)	6/599 (1.0%)	3/566 (0.5%)	0.22 (0.09–0.53)	<0.001	0.12 (0.04–0.39)	<0.001	0.53 (0.13–2.11)	0.37
High-grade pigment detected by histopathology	27/599 (4.5%)	1/599 (0.2%)	5/566 (0.9%)	0.04 (0.01–0.27)	<0.001	0.20 (0.08–0.51)	<0.001	5.29 (0.62–45.2)	0.13
Any parasites or pigment detected by histopathology	316/599 (52.8%)	179/599 (29.9%)	226/566 (39.9%)	0.57 (0.49–0.65)	<0.001	0.76 (0.67–0.86)	<0.001	1.34 (1.14–1.57)	0.004

^1^ SP, sulfadoxine–pyrimethamine; DP, dihydroartemisinin–piperaquine.

^2^ Relative risk for all outcomes with the exception of symptomatic malaria, where the incidence rate ratio was used.

^3^ Episodes of symptomatic malaria (incidence per person-year at risk following initiation of study drugs).

^4^ Measured at the time of routine visits conducted every 4 weeks following initiation of study drugs.

^5^ Hemoglobin measured at 20, 28, and 36 gestational weeks following initiation of study drugs.

### Safety and tolerability outcomes

Compliance with study drugs was high, with only 0.5% (81/15568) of routine visits missed when participants were scheduled to receive their first daily dose of study drugs. In addition, only 0.1% (13/15195) and 0.4% (57/15182) of day 2 and day 3 doses of study drugs were reported to have not been taken at home, respectively. Study drugs were well tolerated, with <0.1% of doses associated with vomiting. There were no significant differences in the incidence of any grade 3–4 adverse events, serious adverse events, or grade 3–4 adverse events possibly related to study drugs between the three treatment arms (Table 4). Of note, congenital anomalies occurred in 23 of 1,696 (1.4%) deliveries in dihydroartemisinin–piperaquine-containing arms compared to 4 of 842 (0.5%) in the sulfadoxine–pyrimethamine arm (*p* = 0.042). Congenital anomalies included 12 episodes of polydactyly, with 11 of 12 in the dihydroartemisinin–piperaquine arms.

**Table 4:** Safety outcomes.

Outcome	SP^1^	DP^1^	DP + SP^1^	DP vs. SP^1^		DP + SP vs. SP^1^		DP + SP vs. DP^1^	
Prevalence measures	*n*/*N* (%)			RR (95% CI)	*P*-value	RR (95% CI)	*P*-value	RR (95% CI)	*P*-value
Post-dosing QTc > 500 ms or interval increase ≥ 60 ms^2^	3/263 (1.1%)	4/279 (1.4%)	4/265 (1.5%)	1.36 (0.24–7.82)	0.73	1.31 (0.23–7.46)	0.76	1.06 (0.27–4.14)	0.93
**Incidence measures**	**Events (incidence per person-year at risk)**			**IRR (95% CI)**	***P*-value**	**IRR (95% CI)**	***P*-value**	**IRR (95% CI)**	***P*-value**
Common adverse events of any severity^3^									
Abdominal pain	1,532 (3.54)	1,431 (3.30)	1,364 (3.15)	0.94 (0.85–1.03)	0.18	0.89 (0.81–0.99)	0.03	0.95 (0.86–1.06)	0.37
Cough	1,064 (2.46)	1,130 (2.61)	1,004 (2.32)	1.06 (0.95–1.19)	0.27	0.94 (0.84–1.05)	0.26	0.88 (0.79–0.98)	0.03
Headache	947 (2.19)	865 (1.99)	769 (1.78)	0.91 (0.82–1.02)	0.12	0.81 (0.72–0.91)	<0.001	0.89 (0.79–1.00)	0.05
Dysuria	379 (0.88)	324 (0.75)	324 (0.75)	0.85 (0.72–1.01)	0.07	0.86 (0.72–1.02)	0.09	1.00 (0.84–1.20)	0.96
Rhinorrhea	189 (0.44)	164 (0.38)	167 (0.39)	0.87 (0.69–1.09)	0.22	0.88 (0.70–1.10)	0.27	1.02 (0.81–1.28)	0.88
Diarrhea	174 (0.40)	143 (0.33)	140 (0.32)	0.82 (0.64–1.05)	0.12	0.80 (0.62–1.04)	0.10	0.98 (0.75–1.27)	0.87
Fatigue	101 (0.23)	79 (0.18)	56 (0.13)	0.78 (0.55–1.11)	0.17	0.56 (0.38–0.81)	0.002	0.71 (0.48–1.07)	0.10
Chills	67 (0.15)	90 (0.21)	66 (0.15)	1.32 (0.91–1.92)	0.14	0.97 (0.67–1.42)	0.89	0.73 (0.51–1.05)	0.09
Epigastric pain	83 (0.19)	79 (0.18)	62 (0.14)	0.96 (0.68–1.35)	0.80	0.75 (0.53–1.06)	0.10	0.78 (0.53–1.15)	0.21
Grade 3–4 adverse events^4^									
Anemia	53 (0.12)	28 (0.06)	44 (0.10)	0.59 (0.37–0.94)	0.03	0.82 (0.55–1.23)	0.34	1.38 (0.87–2.23)	0.17
Stillbirth	15 (0.03)	16 (0.04)	7 (0.016)	1.19 (0.59–2.42)	0.62	0.46 (0.19–1.13)	0.09	0.39 (0.16–0.94)	0.04
Congenital anomaly	4 (0.009)	13 (0.03)	10 (0.023)	3.64 (1.19–11.2)	0.02	2.47 (0.78–7.90)	0.13	0.68 (0.30–1.55)	0.36
Neutropenia	5 (0.012)	10 (0.023)	7 (0.016)	2.24 (0.77–6.56)	0.14	1.39 (0.44–4.37)	0.58	0.61 (0.24–1.63)	0.33
Proteinuria	3 (0.007)	4 (0.009)	8 (0.018)	1.49 (0.33–6.68)	0.60	2.64 (0.70–9.96)	0.15	1.77 (0.53–5.87)	0.35
Thrombocytopenia	7 (0.016)	1 (0.002)	2 (0.004)	0.16 (0.02–1.30)	0.09	0.28 (0.06–1.36)	0.12	1.77 (0.16–19.5)	0.64
Postpartum hemorrhage	2 (0.005)	3 (0.007)	2 (0.004)	1.68 (0.28–10.1)	0.57	0.99 (0.14–7.04)	0.99	0.59 (0.10–3.53)	0.56
Pre-eclampsia	3 (0.007)	0 (0)	2 (0.004)	NA		0.66 (0.11–3.95)	0.65	NA	
Preterm birth < 28 weeks gestational age	0 (0)	0 (0)	3 (0.007)	NA		NA		NA	
Elevated temperature	2 (0.005)	1 (0.002)	0 (0)	0.56 (0.05–6.18)	0.64	NA		NA	
Any grade 3–4 adverse events	99 (0.23)	81 (0.19)	90 (0.21)	0.92 (0.68–1.23)	0.56	0.90 (0.68–1.20)	0.48	0.98 (0.73–1.33)	0.91
Any serious adverse events	25 (0.06)	26 (0.06)	27 (0.06)	1.17 (0.67–2.02)	0.58	1.07 (0.62–1.84)	0.81	0.92 (0.54–1.57)	0.76
Grade 3–4 adverse events possible related to study drugs	50 (0.12)	29 (0.07)	41 (0.09)	0.65 (0.41–1.03)	0.07	0.81 (0.54–1.23)	0.33	1.25 (0.79–2.01)	0.36

^1^ SP, sulfadoxine–pyrimethamine; DP, dihydroartemisinin–piperaquine.

^2^ For the first 300 participants enrolled, electrocardiograms were done at 20, 28, and 36 weeks gestation weeks, just before the first dose and 2–6 h after the third dose of study drugs.

^3^ Includes only those with at least 200 total events.

^4^ Includes only those with at least 3 total events

## Discussion

In this double-blind, randomized controlled trial of monthly IPTp, the malaria burden was high in women who received sulfadoxine–pyrimethamine, the current standard-of-care, with malaria parasites detected in nearly half of these participants at the time of monthly routine visits after initiation of IPTp. Dihydroartemisinin–piperaquine markedly reduced the malaria burden, but this did not translate into overall improvements in birth outcomes. While infants born to women who received dihydroartemisinin–piperaquine had a lower risk of preterm birth than those born to women who received sulfadoxine–pyrimethamine, they also had a lower mean birthweight and higher risk of being small-for-gestational age. Combining dihydroartemisinin–piperaquine plus sulfadoxine–pyrimethamine did not improve malaria-related outcomes compared to dihydroartemisinin–piperaquine alone and was associated with a lower mean birthweight and higher risk of small-for-gestational age compared to sulfadoxine–pyrimethamine alone. Thus, although the combination of dihydroartemisinin–piperaquine plus sulfadoxine–pyrimethamine did provide superior antimalarial activity compared to sulfadoxine–pyrimethamine alone, the combination did not result in an improvement in birth outcomes compared to the current standard-of-care.

In this study, IPTp with dihydroartemisinin–piperaquine offered clear benefits over sulfadoxine–pyrimethamine for the prevention of malaria-related outcomes of clinical relevance to pregnant women, including symptomatic malaria and anemia. The finding that IPTp with dihydroartemisinin–piperaquine had far superior antimalarial activity compared to IPTp with sulfadoxine–pyrimethamine is consistent with findings from other studies from eastern and southern Africa, where *P. falciparum* resistance to sulfadoxine–pyrimethamine is widespread [11,13–16]. In a recent meta-analysis of six trials contributing data on 6,646 pregnancies, IPTp with dihydroartemisinin–piperaquine was associated with a 69% lower incidence of symptomatic malaria, a 62% lower risk of placental parasitemia, and a 17% lower risk of maternal anemia compared to IPTp with sulfadoxine–pyrimethamine. However, the superior antimalarial activity of dihydroartemisinin–piperaquine did not translate into an improvement in birth outcomes. Indeed, in this meta-analysis, sulfadoxine–pyrimethamine was associated with a 34 g/week higher mean maternal weight gain, a 50 g higher mean birthweight, and a 15% lower risk of small-for-gestational age compared to dihydroartemisinin–piperaquine [5]. Observational studies also support these results, showing that the use of IPTp with sulfadoxine–pyrimethamine was associated with higher birthweights and a decreased risk of LBW in a dose-response manner. This effect was observed in areas where antimalarial resistance to sulfadoxine–pyrimethamine was high [17] and in regions with very low malaria transmission, suggesting benefits were independent of the antimalarial activity of sulfadoxine–pyrimethamine [18,19]. These findings might be explained by non-malarial activities of sulfadoxine–pyrimethamine, which has antibacterial properties, and which may act to improve fetal growth and maternal weight gain. The specific mechanisms of this effect, if present, are unclear, although several studies have demonstrated positive impacts of sulfadoxine–pyrimethamine on the intestinal flora and/or nutrient absorption [2,6,20,21], maternal inflammation [22], and prevention of non-malarial infections [15,23]. However, in contrast to summary estimates from the meta-analysis, our study, conducted in a region with a very high malaria burden, found that compared to sulfadoxine–pyrimethamine, dihydroartemisinin–piperaquine was associated with a 48% reduction in the risk of preterm birth, which likely can be explained by its potent antimalarial activity, particularly against active placental malaria infection and symptomatic malaria [24].

We hypothesized that IPTp combining the superior antimalarial properties of dihydroartemisinin–piperaquine with the non-malarial activities of sulfadoxine–pyrimethamine would provide superior prevention against adverse birth outcomes compared to either drug used alone. However, the combination failed to improve birth outcomes and was associated with lower maternal weight gain, lower birthweight, and a higher risk of being born small-for-gestational age compared to sulfadoxine–pyrimethamine alone. These results suggest the possibility that rather than sulfadoxine–pyrimethamine improving fetal growth through non-malarial mechanisms, dihydroartemisinin–piperaquine may negatively affect fetal growth, potentially via adversely affecting maternal weight gain. The optimal study design to test the hypothesis that sulfadoxine–pyrimethamine improves fetal growth independent of its antimalarial activity would be to conduct a randomized controlled trial of sulfadoxine–pyrimethamine versus placebo in an area where malaria is not endemic. We are unaware of such a study being conducted, but a recent randomized controlled trial compared daily trimethoprim-sulfamethoxazole (an antifolate similar to sulfadoxine–pyrimethamine) versus placebo in 993 pregnant women living in an area of Zimbabwe where malaria is not endemic [25]. In this study there was no significant difference in mean birthweight between the daily trimethoprim-sulfamethoxazole and placebo arms (3,040 versus 3,019 g, *p* = 0.53). Similarly, the optimal study design to test the hypothesis that dihydroartemisinin–piperaquine negatively impacts fetal growth independent of its antimalarial activity would be to conduct a randomized controlled trial of dihydroartemisinin–piperaquine versus placebo in an area where malaria is not endemic. This scenario was close to being realized in a study of 2,279 pregnant women in Indonesia who were randomized to receive monthly IPT with dihydroartemisinin–piperaquine, intermittent screening and treatment (IST) with dihydroartemisinin–piperaquine, or single screening and treatment (SST) with dihydroartemisinin–piperaquine [26]. Because this study was done in a low endemic area, it resembled a placebo controlled trial, as 96% of women in the IST arm received no dihydroartemisinin–piperaquine and 94% of women in the SST arm received no dihydroartemisinin–piperaquine, while women in the IPT arm received an average of 3 courses of dihydroartemisinin–piperaquine. Interestingly, in this study women that were randomized to IPT with dihydroartemisinin–piperaquine had significantly lower mean birthweights (2,899 g) compared to women randomized to IST with dihydroartemisinin–piperaquine (2,980 g, *p* = 0.005) or SST with dihydroartemisinin–piperaquine (2,971 g, *p* = 0.02). The possibility that dihydroartemisinin–piperaquine negatively impacts fetal growth is further supported by a randomized trial in which HIV-infected pregnant women given monthly IPTp with dihydroartemisinin–piperaquine plus daily trimethoprim-sulfamethoxazole had significantly lower maternal weight gain and non-significantly lower infant birthweight compared to those given daily co-trimoxazole alone [27].

Alternatively, drug-drug interactions may have reduced individual drug exposure, compromising the antimalarial activity of dihydroartemisinin–piperaquine and/or other activities of sulfadoxine–pyrimethamine. This hypothesis is supported by results from a pharmacokinetic study nested in this trial in which women who received the combination had 25% and 34% lower area under the concentration–time curves (AUC) for sulfadoxine and pyrimethamine, respectively, compared to those who received sulfadoxine–pyrimethamine alone and a 19% lower AUC for piperaquine compared to those who received dihydroartemisinin–piperaquine alone [28]. The pharmacokinetic study finding may also explain why the combination of dihydroartemisinin–piperaquine plus sulfadoxine–pyrimethamine did not improve malaria-related outcomes compared to dihydroartemisinin–piperaquine alone, and indeed was associated with a higher risk of maternal parasitemia and the detection of placental parasites or pigment by histopathology. Regardless of the mechanisms involved, the results of this study do not support the use of a combination of dihydroartemisinin–piperaquine plus sulfadoxine–pyrimethamine for IPTp.

As expected, the burden of malaria was much higher in primigravidae than multigravidae [2]. As a result, the absolute benefit of dihydroartemisinin–piperaquine over sulfadoxine–pyrimethamine in reducing the risk of malaria-related outcomes was much greater in primigravidae compared to multigravidae. Additionally, the benefit of dihydroartemisinin–piperaquine over sulfadoxine–pyrimethamine in reducing the risk of anemia was only observed in primigravidae; this benefit may have decreased the likelihood of adverse birth outcomes. The risk of adverse birth outcomes was also higher in primigravidae than multigravidae, which may be due to the development of “gravidity-dependent immunity” [2]. This likely explains why associations between IPTp regimens and birth outcomes differed by gravidity. Among primigravidae, who benefitted the most from protection against malaria-associated adverse birth outcomes, the net effect of IPTp with dihydroartemisinin–piperaquine was a decreased risk of preterm delivery, but no significant differences in other adverse birth outcomes compared to IPT with sulfadoxine–pyrimethamine. In contrast, the impact of IPTp with dihydroartemisinin–piperaquine on malaria-related birth outcomes was less pronounced among multigravidae, with the net effect being lower birthweight and an increased risk of small-for-gestational age compared to IPTp with sulfadoxine–pyrimethamine. These findings suggest that, with high malaria transmission intensity, IPTp with dihydroartemisinin–piperaquine may offer benefits over sulfadoxine–pyrimethamine in primigravidae, but not in multigravidae.

The results of this study must be considered in light of the particularly high-level resistance of *P. falciparum* to sulfadoxine and pyrimethamine in Uganda, mediated by five mutations in PfDHFR (N51I, C59R, and S108N) and PfDHPS (A437G and K540E) that are associated with poor preventive efficacy of sulfadoxine–pyrimethamine and highly prevalent in Uganda [12,29]. Of concern, two additional mutations that mediate even higher-level resistance, PfDHFR I164L and PfDHPS A581G, are increasing in prevalence in Uganda [12,30]. Thus, it is not surprising that the malarial preventive efficacy of sulfadoxine–pyrimethamine appears to be very limited in Uganda, and the efficacy of this regimen might be better in regions, such as West Africa, with lower levels of drug resistance.

Tolerability and safety are important considerations when evaluating drugs for routine use during pregnancy. All three IPTp regimens were well tolerated, with no significant differences in the incidence of any grade 3–4 adverse events or serious adverse events. Of concern, 11 of 12 infants born with polydactyly had mothers who received dihydroartemisinin–piperaquine. These findings are similar to those from a previous study by our group from the same study area comparing monthly IPTp with dihydroartemisinin–piperaquine versus sulfadoxine–pyrimethamine, in which 9 of 10 infants born with polydactyly had mothers who received dihydroartemisinin–piperaquine [11]. Polydactyly has been amongst the most commonly observed congenital anomalies in African populations, and genetic factors have been postulated to explain this high frequency [31,32]. A causal link between IPTp with dihydroartemisinin–piperaquine and polydactyly appears unlikely from an embryologic standpoint, given limb development occurs in the first trimester, before initiation of IPTp [33]. In addition, an increased risk of congenital anomalies or polydactyly has not been reported in other studies of IPTp with dihydroartemisinin–piperaquine [13–16]. However, a significantly increased risk of polydactyly in the dihydroartemisinin–piperaquine arms of two independent trials of IPTp offers some reason for caution when considering potential benefits of this regimen.

This study had some limitations. It was conducted in an area of high transmission intensity with widespread resistance to sulfadoxine–pyrimethamine, limiting generalizability to other settings. Only the first daily dose of study drugs was directly observed, which could have differentially affected exposure to dihydroartemisinin–piperaquine, a three-day regimen, although reported compliance to the second and third doses administered at home was high. *P*-values were not adjusted for multiple comparisons, warranting cautious interpretation. The study was not powered to detect differences in individual components of the primary endpoint or between gravidity subgroups. Thus, non-statistically significant associations should not necessarily be interpreted as an absence of effect. Findings presented in this report did not include investigations of potential mechanisms by which IPTp regimens may have affected birth outcomes independent of their antimalarial activity. Lastly, a limitation of IPTp in general is the fact that drugs are not administered until the second trimester due to safety concerns. Indeed, in this study from an area of high malaria endemicity, over 70% of women were infected with malaria parasites at the time of enrollment which could have adverse consequences on birth outcomes that cannot be fully prevented even with the subsequent use of highly effective IPTp regimens.

This study provides further evidence that, in areas with high-level *P. falciparum* resistance to sulfadoxine–pyrimethamine, the burden of malaria may be unacceptably high among pregnant women administered the current standard of care for IPTp. Replacing sulfadoxine–pyrimethamine with dihydroartemisinin–piperaquine for IPTp would likely result in significant reductions in clinical malaria and maternal anemia, especially among primigravidae. However, such a change may negatively impact fetal growth. Combining dihydroartemisinin–piperaquine and sulfadoxine–pyrimethamine offered promise, but provided no clear benefits, raising the unexpected possibility that dihydroartemisinin–piperaquine adversely affects maternal weight gain and fetal growth, although the mechanism of this effect is unknown. Overall, the relative contributions to birth outcomes of potential negative impacts of DP, potential positive impacts of SP, and decreased drug exposure with coadministration are uncertain. Further studies are needed to better elucidate the mechanisms by which dihydroartemisinin–piperaquine and sulfadoxine–pyrimethamine affect fetal weight gain independent of antimalarial activity. Research is also needed to identify new interventions, such as alternative IPTp regimens, vaccines, or monoclonal antibodies, to better prevent malaria in pregnancy, reduce the risk of adverse birth outcomes, and ultimately improve the health of pregnant women and their infants.

## Supporting information

S1 Fig Illustration of administration of each course of active study drugs and/or placebos. (EPS)

S1 File CONSORT checklist. This checklist is licensed under the Creative Commons Attribution 4.0 International License (CC BY 4.0; https://creativecommons.org/licenses/by/4.0/).
(PDF)

S2 File Study protocol. (PDF)

S3 File Statistical analysis plan. (PDF)

## Abbreviations

**:**
AUC: area under the concentration–time curves
IPTp: intermittent preventive treatment of malaria in pregnancy
IRRs: incidence rate ratios
IST: intermittent screening and treatment
LBW: low birth weight
LLIN: long-lasting insecticidal net
qPCR: quantitative PCR
RR: relative risk
SST: single screening and treatment
