# Supplementary material for: Dihydroartemisinin–piperaquine plus sulfadoxine–pyrimethamine versus either drug alone for intermittent preventive treatment of malaria in pregnancy: A double-blind, randomized, controlled phase 3 trial from Uganda
Source: PLoS Med. 2025 Sep 18;22(9):e1004582. doi: 10.1371/journal.pmed.1004582 (PMC12445490; doi:10.1371/journal.pmed.1004582)
Supplement: S2 File — (PDF) [file pmed.1004582.s003.pdf]

Title: Optimal chemopreventive regimens to prevent malaria and improve birth outcomes in Uganda

Short title: Optimizing IPTp in Uganda

A UCSF/ IDRC COLLABORATION

**DMID Protocol Number:**	19-0014
**DMID Funding Mechanism:**	U01 AI141308-01A1
**Principal Investigators:**	Grant Dorsey, MD, PhD<br>University California San Francisco<br><br>Philip Rosenthal, MD<br>University California San Francico<br><br>Moses Kamya, MBChB, MMed, PhD<br>Makerere University College of Health Sciences
**DMID Medical Officer:**	Dr. Greg Deye
**DMID Medical Monitor:**	Dr. Mohamed Elsafy
**DMID Clinical Project Manager:**	Walt Jones

Protocol Version 8.0

September 11, 2023

* Ultrasound provided by Foundation for Society of Maternal Fetal Medicine

STATEMENT OF COMPLIANCE

The study will be carried out in accordance with Good Clinical Practice (GCP) as required by the following:

- United States (US) Code of Federal Regulations (CFR) applicable to clinical studies (45 CFR Part 46; 21 CFR Part 50, 21 CFR Part 56, and 21 CFR Part 312)
- ICH E6; 62 Federal Register 25691 (1997)
- Ugandan National Council for Science and Technology
- Completion of Human Subjects Protection Training
- NIH Clinical Terms of Award

SIGNATURE PAGE

The signature below constitutes the approval of this protocol and the attachments, and provides the necessary assurances that this study will be conducted according to all stipulations of the protocol, including all statements regarding confidentiality, and according to local legal and regulatory requirements and applicable US federal regulations and ICH guidelines.

Site Principal Investigator: Professor Moses Kamya, MBChB, MPH, PhD

Signed: ____

Name Prof Moses Kamya

Title Professor and Dean, School of Medicine, Makerere University College of Health Sciences
Executive Director, Infectious Diseases Research Collaboration (IDRC)

TABLE OF CONTENTS

STATEMENT OF COMPLIANCE 2

SIGNATURE PAGE 3

PROTOCOL TEAM ROSTER 8

LIST OF ABBREVIATIONS 10

CLINICAL PROTOCOL SYNOPSIS 11

1. INTRODUCTION 12

1.1. Background and preliminary studies 12

1.2. Rationale 18

2. STUDY OBJECTIVES 20

2.1. Objective 1 20

2.2. Objective 2 20

2.3. Objective 3 20

3. STUDY DESIGN 20

4. SELECTION AND ENROLLMENT OF SUBJECTS 21

4.1. Inclusion Criteria 21

4.2. Exclusion Criteria 21

4.3. Initial Screening 22

4.4. Study Enrollment Procedures and Baseline Evaluation 22

5. STUDY TREATMENT 23

5.1. Treatment Group Assignments 23

5.2. Treatment Allocation 24

5.3. Study Drug Dosing and Formulations 24

5.4. Blinding, Study Drug Administration, and Duration 25

5.5. Study Drug Accountability 25

6. SUBJECT MANAGEMENT 26

6.1. Subject Follow-up 26

6.2. Diagnosis and Management of Malaria....26
6.3. Management of Non-Malaria Illnesses....27
6.4. After Hours Visits....28
6.5. Routine Assessments....28
6.6. Delivery visit....30
6.7. Postpartum visits.....30
6.8. Medical Care Outside the Study Clinic....30
6.9. Duration of Follow-up and Criteria for Premature Study Withdrawal....31
6.10. Diagnostic and Laboratory Testing....31
6.10.1. Microscopy....31
6.10.2. Clinical Laboratory Studies....32
6.10.3. Placental Studies....32
6.10.4. Dried blood spots collected on filter paper....33
6.10.5. Quantitative PCR....33
6.10.6 Ex vivo drug sensitivity of malaria parasites....34
6.10.7 Microbiome studies....34
6.10.8 Reproductive tract infections (RTIs)....37
6.10.9 Drug sensitivity of urinary tract infection (UTI) pathogens and *Neisseria gonorrhea*....38
6.10.10 Molecular markers of SP and DP resistance....39
6.10.11 Pharmacokinetic assessments.....40
6.10.12 Immunology Studies....41
6.10.13 Host red blood cell polymorphisms.....42
6.10.14 SARS-CoV-2 Testing.....43
6.10.15 Fetal Ultrasounds during and after febrile illness....45
6.10.16 Sample storage for future use....47
6.11. Co-enrollment Guidelines....48
6.12. Management of Adverse Events Potentially Related to Study Drugs....48
6.12.1. Grade 1 or 2 Adverse Events....48
6.12.2. Grade 3 or 4 Adverse Events....48
7. MONITORING OF ADVERSE EVENTS AND MANAGEMENT....49
7.1 Monitoring and Reporting of Adverse Events....49
7.1.1 Definitions....49
7.1.2 Identification of Adverse Events....49
7.1.3 Reporting of Adverse Events....50
8. STATISTICAL CONSIDERATIONS....51
8.1 Hypothesis 1....52
8.1.1. Primary Outcome....52

8.1.2 Analysis 52

8.2 Hypothesis 2 52

8.2.1. Primary Outcome 52
8.2.2. Secondary Outcomes 53
8.2.3. Analysis 53

8.3 Hypothesis 3 53

8.3.1. Outcomes for objective 3a 53
8.3.2. Outcomes for objective 3b 54
8.3.3. Analyses 54

8.4 Sample size and power 54

8.5 Data and Safety Monitoring Plan 55

8.6 Data and Safety Monitoring Board 55

8.7 Interim safety analysis 57

8.8 Stopping rules 58

9 DATA COLLECTION AND MONITORING 58

9.1 Record Keeping 58

9.2 Data Quality Assurance and Monitoring 58

10. HUMAN SUBJECTS 58

10.1 Subject Selection Criteria 59

10.2 Risks and Discomforts 59

10.2.1 Privacy 59
10.2.2 Finger Pricks and Venipuncture 59
10.2.3 Risks of Study Medications 59

10.3 Treatment and Compensation for Injury 66

10.4 Costs to the Subjects 66

10.5 Reimbursement of Subjects 66

10.6 IRB Review and Informed Consent 66

10.7 Publication of Research Findings 67

10.8 Biohazard Containment 67

11. REFERENCES 67

12. APPENDICES 77

Appendix A. Information Sheet 77

Appendix B. Determination of Gestational Age 78

Appendix C. Household survey 79
Appendix D. Administration of study drugs and placebos...83
Appendix E. WHO Criteria for Severe Malaria 84
Appendix F. Uganda Ministry of Health Guidelines for Routine Care of Pregnant and Postpartum Women, and Newborns 85
Appendix G. Schedule of routine assessments and procedures in pregnant women 86
Appendix H. Division of AIDS (DAIDS) Table for Grading the Severity of Adult and Pediatric Adverse Events (Corrected Version 2.1 - July 2017) 88
Appendix I. Pharmacokinetic Sub-Study..116

PROTOCOL TEAM ROSTER

Investigators

Grant Dorsey, MD, PhD, University of California, San Francisco
Address: San Francisco General Hospital, 995 Potrero Avenue,
Building 30, Room 3420, UCSF Box 0811, San Francisco CA, 94110, U.S.A.
Phone Number: +1-415-206-4680
Fax Number: +1-415-648-8425
Email: grant.dorsey@ucsf.edu

Philip Rosenthal, MD, University of California, San Francisco
Address: San Francisco General Hospital, 995 Potrero Avenue,
Building 30, Room 3421, UCSF Box 0811, San Francisco CA, 94110, U.S.A.
Phone Number: +1-415-206-8845
Fax Number: +1-415-648-8425
Email: Philip.Rosenthal@ucsf.edu

Stephanie Gaw, MD, PhD, University of California, San Francisco
Address: 513 Parnassus Ave, 16HSE, San Francisco CA 94143, U.S.A.
Phone Number: +1- 415-476-0535
Email: Stephanie.Gaw@ucsf.edu

Moses R. Kamya, MBChB, MMed, PhD, Makerere University College of Health Sciences
Address: Department of Medicine, P.O. Box 7072, Kampala, Uganda
Phone Number: +256-414-533200
Fax Number: +256-414-540524
Email: mkamya@infocom.co.ug

Miriam Nakalembe, MBChB, MMed Ob/Gyn, Makerere University College of Health Sciences
Address: Department of Medicine,
P.O. Box 7072, Kampala, Uganda
Phone Number: +256 753 857 433 (Mobile), +256 414 534 361 (Office)
Fax +256 414 533 451 (AOGU Office)
Email: ivuds@yahoo.com

Abel Kakuru, MBChB, MSc, Infectious Diseases Research Collaboration
Address: Infectious Diseases Research Collaboration,
P.O. Box 7475, Kampala, Uganda
Phone Number: +256-414-530692
Fax Number: +256-414-540524
Email: abelkakuru@gmail.com

Prasanna Jagannathan, MD, Stanford University
Address: 300 Pasteur Drive, Lane Building, Suite 134, L133,
Stanford, California 94305, U.S.A.
Phone Number: +1-650-724-5343
Fax Number: +1-650-498-9876
Email: prasj@stanford.edu

Francesca Aweeka, PharmD, University of California, San Francisco
Address: 1001 Potrero Ave, SFGH 100, Rm 158, San Francisco, CA 94110, U.S.A.
Phone Number: +1 415 476-0339
Fax Number: +1 415 476-0307
Email: fran.aweeka@ucsf.edu

LIST OF ABBREVIATIONS

AE	Adverse event
ACT	Artemisinin-based combination therapy
AL	Artemether-lumefantrine
BV	Bacterial vaginosis
CAB	Community advisory board
CBC	Complete blood cell count
CHR	Committee on Human Research
CRF	Case report form
DP	Dihydroartemisinin-piperaquine
DAIDS	National Institutes of Health Division of AIDS
DMID	National Institutes of Health Division of Microbiology and Infectious Diseases
DSMB	Data and Safety Monitoring Board
ECG	Electrocardiogram
FDA	Food and Drug Administration
GBS	Group B Streptococcus
ICH	International Conference on Harmonisation
IDRC	Infectious Diseases Research Collaboration
IPT	Intermittent preventive treatment
IPTp	Intermittent preventive treatment in pregnancy
IRB	Institutional review board
IST	Intermittent screening and treatment
LLIN	Long lasting insecticide treated net
LBW	Low birth weight
LMP	Last menstrual period
MOH	Ministry of Health
MU	Makerere University
MU-SBSREC	MU School of Biomedical Sciences Research and Ethics Committee
NDA	National Drug Authority
NIH	National Institute of Health
qPCR	Quantitative polymerase chain reaction
RCT	Randomized controlled trial
RTIs	Reproductive tract infections
SAE	Serious adverse event
SNP	Single nucleotide polymorphisms
SGA	Small-for-gestational age
SP	Sulfadoxine-pyrimethamine
TMP-SMX	Trimethoprim-sulfamethoxazole
UCSF	University California San Francisco
UNCST	Uganda National Council of Science and Technology
UTI	Urinary Tract Infection
WHO	World Health Organization

Clinical Protocol Synopsis

**Title**	Optimal chemopreventive regimens to prevent malaria and improve birth outcomes in Uganda
Description	Double blinded randomized controlled trial
Study Objectives	1) To compare the risk of adverse birth outcomes among pregnant women randomized to receive monthly IPTp with SP vs. DP vs. SP+DP.<br>2) To compare safety and tolerability of IPTp regimens among pregnant women randomized to receive monthly IPTp with SP vs. DP vs. SP+DP.<br>3) To compare risks of malaria-specific and non-malarial outcomes among pregnant women randomized to receive monthly IPTp with SP vs. DP vs. SP+DP.
**Participants and Sample Size**	2757 HIV-uninfected pregnant women
**Clinical Site**	The study will be conducted in Busia District, Uganda. A designated study clinic located within Masafu General Hospital will be open daily from 8:00 am to 5:00 pm and after-hours care will be available within the hospital complex.
**Selection Criteria**	<u>Inclusion Criteria</u><br>1. Viable singleton pregnancy confirmed by ultrasound<br>2. Estimated gestational age between 12-20 weeks<br>3. Confirmed to be HIV- uninfected by rapid test<br>4. 16 years of age or older<br>5. Residency within Busia District of Uganda<br>6. Provision of informed consent<br>7. Agreement to come to the study clinic for any febrile episode or other illness and avoid medications given outside the study protocol<br>8. Willing to deliver in the hospital<br><u>Exclusion Criteria:</u><br>1. History of serious adverse event to SP or DP<br>2. Active medical problem requiring inpatient evaluation at the time of screening<br>3. Intention of moving outside of Busia District Uganda<br>4. Chronic medical condition requiring frequent medical attention<br>5. Prior chemopreventive therapy or any other antimalarial therapy during this pregnancy<br>6. Early or active labor (documented by cervical change with uterine contractions)<br>7. Multiple pregnancies (i.e. twins/triplets)<br>8. History of long QT syndrome
**Treatment assignment**	HIV-uninfected pregnant women will be randomized at the time of enrollment
**Treatment arms**	1. SP given every 4 weeks<br>2. DP given every 4 weeks<br>3. SP+DP given every 4 weeks
**Follow-up**	Study participants will be followed for all of their outpatient medical care in our study clinic. Routine assessments will be performed in the study clinic for all study participants every 4 weeks. Participants will be followed until 28 days postpartum.
**Primary study outcome**	The risk of having a composite adverse birth outcome defined as the occurrence of any of the following:<br>• Spontaneous abortion (fetal loss at < 28 weeks gestational age)<br>• Stillbirth (infant born deceased at $\geq$ 28 weeks gestational age)<br>• Low birth weight (< 2500 gm)<br>• Preterm birth (< 37 weeks gestational age)<br>• Small-for-gestational age (< 10th percentile relative to an external growth reference)<br>• Neonatal death (death of infant within the first 28 days)

1. INTRODUCTION

1.1. Background and preliminary studies

Malaria in pregnancy: burden of disease and prevention. Malaria in pregnancy remains a major challenge in sub-Saharan Africa, where *P. falciparum* is the predominant species and transmission remains moderate to high in many areas. In this setting a high proportion of infected women are asymptomatic, with low-level parasitemia often missed by microscopy or rapid diagnostic tests.[1] These infections may lead to placental sequestration resulting in LBW due to preterm delivery and/or intrauterine growth restriction.[2] Other complications of malaria in pregnancy include maternal anemia, spontaneous abortions, and stillbirths, which combined with LBW are estimated to cause over 100,000 fetal and neonatal deaths each year.[3]

The only widely available tools for the prevention of malaria in pregnancy are LLINs and IPTp-SP. Resistance to the pyrethroid class of insecticides used in LLINs has become widespread throughout Africa, potentially compromising their efficacy and increasing reliance on antimalarial drugs for the prevention of malaria during pregnancy.[4-6] SP was the first drug endorsed for IPTp, and it remains the only drug recommended by the WHO. In a Cochrane review of IPTp-SP vs. placebo from 6 randomized controlled trials conducted in Africa primarily in the 1990s before widespread SP resistance, IPTp-SP was associated with a 62% reduction in the risk of maternal parasitemia, 55% reduction in the risk of placental parasitemia, and 19% reduction in the risk of LBW.[7] The meta-analysis of these trials was underpowered to evaluate the effects of IPTp-SP on preterm delivery and fetal/neonatal deaths, as these outcomes were not always measured and/or relatively uncommon. In a separate retrospective analysis of cross-sectional datasets from 25 African countries from 2000-10, a combination of LLINs and IPTp-SP was associated with a 21% reduction in the risk of LBW and 19% reduction in the risk of neonatal mortality.[8] In the early 2000s the WHO began recommending IPTp with at least 2 doses of SP beginning in the second trimester, and subsequently placebo controlled trials of IPTp have been deemed unethical. However, given the emergence of SP resistance in Africa and the potential for shortening the period of post-treatment prophylaxis, a number of studies have looked at the impact of more frequent dosing of SP for IPTp. In a meta-analysis of 7 studies from Africa, 3 or more doses of SP was associated with a 49% reduction in the risk of placental malaria and a 20% reduction in the risk of LBW compared to 2 doses of SP.[9] Interestingly, these differences were consistent across a wide range of prevalence of SP resistance markers. In 2012 the WHO updated their recommendations, increasing the frequency of dosing of IPTp with SP to up to once a month, beginning as early in the 2nd trimester as possible.

Despite the strong historical evidence for the benefits of IPTp-SP, there is concern that increasing spread of SP resistance will compromise its effectiveness. SP was abandoned as a recommended treatment for malaria in Africa in the early-2000’s and it has had limited or no

protective efficacy when used for chemoprevention in non-pregnant populations. In a randomized controlled trial from our group in Uganda, monthly SP provided no significant protection against malaria among children 6-24 months of age.[10] In many parts of Africa (especially Eastern and Southern Africa), more than 90% of parasites harbor five *dhfr/dhps* mutations that have been associated with SP failure to clear existing infections and prevent new infections in pregnant women.[11,12] Indeed, the presence of a sixth mutation (*dhps* 581G), which confers high-level SP resistance, is emerging in East Africa; this mutation will likely further compromise the efficacy of IPTp-SP.[13-15] The spread of SP resistance has prompted the evaluation of alternative regimens for IPTp. In a randomized controlled trial conducted in Ghana, IPTp with amodiaquine alone or in combination with SP was poorly tolerated and did not improve outcomes compared to SP alone.[16] In a multicenter randomized controlled trial, mefloquine provided in two dosing strategies lowered the risk of malaria during pregnancy compared to SP but did not improve birth outcomes and was poorly tolerated.[17] In a multicenter randomized controlled trial, a combination of chloroquine and azithromycin also lowered the risk of malaria during pregnancy compared to SP but did not improve birth outcomes and was poorly tolerated due to vomiting.[18] In a randomized controlled trial from Malawi, a combination of monthly SP plus two doses of azithromycin was not associated with significant improvement in the risks of LBW, preterm delivery, or placental malaria compared to monthly SP alone.[19,20] In summary, results of studies evaluating alternatives to IPTp-SP using older antimalarial drugs have been disappointing and have not led to changes in policy recommendations.

ACTs for the treatment and prevention of malaria in pregnancy. The ACT class of drugs offers an attractive alternative to SP for use in pregnancy. In a systematic review of parasitological efficacy for the treatment and prevention of falciparum malaria in pregnancy, placenta-positive rates were unacceptably high in a majority of SP trial arms and ACTs provided the lowest parasitological failure rates.[21] The authors recommended that SP should no longer be used for treatment or prevention of malaria in pregnancy and that ACTs provide the most efficacious and safe alternative therapy. Several randomized controlled trials have shown that ACTs are safe and effective for the treatment of malaria during pregnancy[22-25] and the introduction of DP as first line-treatment in Papua, Indonesia was temporally associated with a significant decrease in the risk of severe maternal anemia and LBW.[26] Although the WHO has now endorsed ACTs as first-line therapy for malaria in the 2nd and 3rd trimesters of pregnancy, this is likely to have limited impact in endemic areas of Africa, where most infections during pregnancy are asymptomatic. This has stimulated interest in expanding the role of ACTs to IPTp. The most attractive option for IPTp is DP, given its excellent safety/efficacy profile and long post-treatment prophylactic effect.

IPTp with DP vs. SP. There have been 3 published RCTs evaluating DP for IPTp, one in Kenya and 2 from our group in Uganda (Table 1). The Kenyan study evaluated IPTp with SP or DP given at the time of each antenatal visit (median of 3 doses) and intermittent screening and treatment with DP, in which women were screened for parasitemia with a rapid diagnostic test (RDT) at each antenatal visit, and only given DP if parasitemia was detected.[27] In Uganda we compared IPTp with SP given every 8 weeks with DP given every 8 or 4 weeks[28] and then compared IPTp with SP or DP given every 4 weeks.[29] In all three studies, DP was associated with significant reductions in the incidence of malaria, parasite prevalence, and parasitemia at delivery compared to SP (Table 1). Indeed, in our latest study from Busia District (the site of our proposed trial), IPTp with DP was associated with $\geq$ 94% protective efficacy against these malaria specific outcomes. However, despite marked reductions in the burden of malaria during pregnancy, none of these studies demonstrated significant differences in the risks of adverse birth outcomes; indeed, mean birth weights were highest in the SP arms in all three studies (Table 1). Other key findings included: 1) Intermittent screening and treatment was ineffective in Kenya, as seen in studies of intermittent screening and treatment with DP in Malawi[30] and AL in West Africa;[31] the WHO recently concluded that intermittent screening and treatment should not be recommended.[32] 2) In Uganda, DP every 4 weeks was superior to DP every 8 weeks for several malaria specific outcomes. 3) In all 3 studies IPTp with DP was safe and as well tolerated as that with SP. Based on these data, the WHO Malaria Policy Advisory Committee recently concluded that DP is promising for IPTp, but that SP is still recommended in the absence of clear data showing improvements in birth outcomes with DP.[32]

Table 1. Results of randomized controlled trials comparing IPTp with DP vs SP

Location	Drug	Frequency of doses	Enrolled	Malaria incidence[a]	Parasite prevalence[b]	Parasitemia at delivery[c]	LBW[d]	Preterm birth[d]	SGA[d]	Mean birth weight (gm)
Western Kenya[27]	SP	each visit	515	0.38	19.1%	10.2%	4.4%	4.7%	5.9%	3276
	DP	each visit	516	0.06	7.1%	3.3%	5.3%	5.1%	6.5%	3188
	DP	IST	515	0.53	15.9%	12.6%	4.6%	6.4%	8.7%	3234
Tororo, Uganda[28]	SP	every 8 weeks	106	0.95	40.5%	19.4%	13.3%	8.2%	20.4%	2950
	DP	every 8 weeks	94	0.31	16.6%	4.6%	14.8%	11.4%	18.2%	2904
	DP	every 4 weeks	100	0	5.2%	2.1%	7.2%	4.1%	18.6%	2939
Busia, Uganda[29]	SP	every 4 weeks	391	0.53	30.8%	12.5%	8.2%	7.3%	13.2%	3038
	DP	every 4 weeks	391	0.03	0.5%	0.3%	7.1%	4.8%	16.7%	3018

[a] Episodes of symptomatic malaria per person; [b] Detection of malaria parasites by microscopy (Kenya and Busia, Uganda) or LAMP (Tororo, Uganda); [c] Malaria parasites detected in maternal blood, placental blood, or placental tissue at the time of delivery; [d] Among live births

In summary, a clear pattern has emerged, with IPTp-DP being far superior to IPTp-SP in reducing the burden of malaria during pregnancy, but showing no clear benefit in improving birth outcomes. <u>One logical explanation of this paradox, and the central hypothesis of this proposal, is that IPTp-SP improves birth outcomes independent of its limited antimalarial</u>

activity, offering the potential for benefit in addition to that afforded by IPTp-DP when administered to pregnant women.

Evidence for IPTp with SP improving birth outcomes independent of its antimalarial properties.

IPTp-SP improves birth outcomes due to non-antimalarial effects. We conducted a meta-analysis of individual level data comparing IPTp with DP vs SP, including only dosing regimens that were directly comparable for each study described above. Causal mediation analysis was used to distinguish antimalarial and non-antimalarial effects based on placental malaria outcomes (Figure 1). In all 3 studies, SP was associated with a relative increase in birth weight due to non-antimalarial effects after adjusting for other mediators in the causal pathway (overall gain of 84 grams, 95% CI 41-128), and DP was associated with a relative increase in birth weight due to antimalarial effects (overall gain of 46 grams, 95% CI 28-66). These data support our central hypothesis and complement other studies that have shown positive impacts of IPTp-SP on LBW in areas where antimalarial resistance to SP is very common[11,33] and areas where the prevalence of malaria infection is very low.[34]

Figure 1. Antimalarial and non-antimalarial effects of IPTp with SP vs DP on birth weight using causal mediation analysis

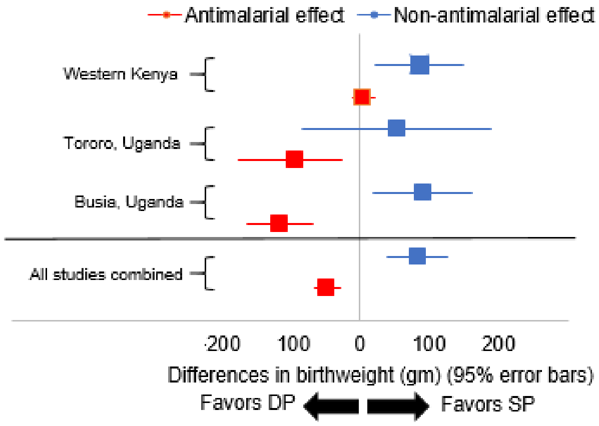

Potential impacts of SP on RTIs and birth outcomes. Treatable sexually transmitted pathogens, notably *Chlamydia trachomatis*, *Neisseria gonorrhea*, and *Trichomonas vaginalis*, are estimated to cause >350 million infections annually.[35] These infections are associated with adverse birth outcomes, including fetal growth restriction, preterm birth, LBW, fetal loss, and neonatal death.[36-39] Other infections also impact upon birth outcomes. Studies of association between colonization with GBS and preterm birth have yielded conflicting results,[37] but true GBS infections are important causes of preterm birth, stillbirth, and puerperal sepsis, and maternal colonization is the main risk factor for early-onset neonatal GBS infection, a primary cause of neonatal sepsis.[40] Thus, antibiotic prophylaxis to prevent early-onset GBS infection in high-risk deliveries is now standard practice in developed countries, but typically not in the developing world.[41] Infection with *Mycoplasma genitalum*,[42] related *Mycoplasma* and *Ureaplasma* species,[43] and *Candida*,[44] and UTIs[37] may also be associated with preterm birth. BV, or overgrowth of vaginal bacteria other than lactobacilli, in particular *Gardnerella*, *Prevotella*, and *Atopobium*, is a strong predictor of preterm birth.[45] BV is also associated with increased risk of

other RTIs associated with adverse birth outcomes, notably trichomonal, gonococcal, and chlamydial infections.[46] A Cochrane review suggested that treatment of BV early in pregnancy may reduce the risk of preterm birth.[47] Chorioamnionitis, caused by *Ureaplasma*, *Mycoplasma*, *Gardnerella*, *Bacteroides*, aerobic gram negative rods, and GBS, is present in an estimated 25% of preterm deliveries and strongly associated with adverse birth outcomes, including neonatal sepsis, fetal growth restriction, and death.[48,49]

Our understanding of antibacterial activities of IPTp-SP is limited, but in a region of Zambia with widespread SP resistance in *P. falciparum*, increasing the dosing frequency of IPTp-SP was associated with decreasing risk of RTIs and improved birth outcomes.[34,50] One study showed at least intermediate activity of SP against multiple gram positive pathogens, including GBS.[51] In a recent trial, IPTp with SP did not reduce recto-vaginal colonization with GBS, but this study used an outmoded regimen (SP twice during pregnancy).[52] In predicting activity of SP against RTI pathogens, it is useful to consider the antifolate TMP-SMX, which is better studied and has a similar antibacterial spectrum. TMP-SMX is a primary therapy for many infections that may impact on birth outcomes in Africa, including common UTI pathogens, salmonellosis, brucellosis, melioidosis, and Q fever. Additional pathogens are likely impacted by antifolates; in the only available comprehensive review TMP-SMX was noted to be active against >75% of strains of *C. trachomatis*, *N. gonorrhea*, and GBS.[53] Studies in HIV-infected individuals have suggested additional benefits for TMP-SMX related to anti-inflammatory activities and impacts on the gut microbiome.[54]

Potential impacts of SP and DP on the gastrointestinal and vaginal microbiome. Non-malarial impacts of SP may be due in part to effects on the complex microbial communities found in and on the human body (the microbiota) or their aggregate genes and metabolic activities (the microbiome). Antibiotics have demonstrated remarkable effects independent of elimination of pathogenic microbes. These include the promotion of weight gain in agricultural animals,[63] improvement in severely malnourished children,[64] and a survival advantage in healthy children receiving infrequent azithromycin to prevent trachoma.[65,66] In rodents, antibiotics during pregnancy altered microbiome composition and promoted weight gain,[67,68] but data in humans are limited. As expected, antibiotics with different spectra differ in their impacts on gut microbiota.[69] Effects of metronidazole, which targets anaerobes, were more modest than those of vancomycin, which targets aerobic gram positives.[70,71] Although studies of effects of SP or TMP-SMX are not available, extrapolation from results for vancomycin suggests that SP, which targets aerobic gram positive and gram negative organisms, would be expected to have profound impacts on the gut microbiome. Considering the vaginal microbiota, lactobacilli,[72] including vaginal isolates from pregnant women,[73] were universally resistant to TMP-SMX, suggesting that SP will not foster loss of these organisms, which appear to be important for maintenance of vaginal homeostasis.[74]

Pregnancy is accompanied by dramatic changes in the gut microbiota, including reduced individual richness (total number of species), increased between-subject diversity, increased abundance of the Proteobacteria and Actinobacteria phyla, and decreased abundance of *Faecalibacteria* and related genera.[75] Gut microbiota may play a role in weight gain during pregnancy via increased absorption of glucose and fatty acids, increased adipocyte factor secretion, and induction of catabolic pathways.[76,77] The vaginal microbiome also undergoes major changes during pregnancy including, in one small study, decreased overall diversity and increased abundance of *Lactobacillus* species.[78] Lactobacilli normally dominate the vaginal microbiota, and their increase during pregnancy correlates with a decrease in the vaginal pH, creating a barrier against pathogenic bacteria.[79,80] The fetus and placenta have until recently been considered sterile, but recent data suggest that bacteria reside in a healthy placenta, with demonstration of a unique placental microbiome,[81] although these data are controversial.[75] A recent study used 16S rRNA sequencing to demonstrate that chorioamnionitis is associated with marked changes in the placental microbiota that are in turn associated with adverse birth outcomes.[82] Microbiomes differ among ethnic groups,[79] between developed world and African populations,[83] and depending on dietary intake,[84] but impacts of interventions on the microbiota are not yet well characterized, especially in African populations. Considering the importance of changes in microbiota during pregnancy and marked impacts of antibiotics, it seems likely that SP alters the maternal microbiome in a manner that affects birth outcomes.

Very little information is available on impacts of the components of DP on the microbiome, but as these compounds do not have known antibacterial activity, their impact is anticipated to be modest. One study showed changes in the rat gut microbiome caused by artesunate, but this was in the context of long-term, high-dose therapy that led to cirrhosis, so likely of little relevance to IPTp-DP.[85] While it is impossible to predict the specific changes associated with DP or SP, we expect that these will be greater with SP, and that, considering other evidence regarding antibiotic effects, these changes may mediate non-malarial benefits of SP.

IPTp regimens and the selection of antimalarial drug resistance. Resistance to SP is well characterized, with 5 mutations in PfDHFR (51I, 59R, and 108N) and PfDHPS (437G and 540E) now common in much of Africa and associated with an intermediate level of resistance.[55] Additional mutations, notably *pfdhfr* 164L and *pfdhps* 581G, lead to high-level resistance; these were rare in older surveys, but recent studies,[56,57] including our surveys in Uganda,[58] have shown emergence of these mutations. Of concern is whether use of SP will select for mutations that mediate high-level resistance, rendering SP nearly worthless as an antimalarial. There have been important recent insights into resistance to both components of DP. Piperaquine is an aminoquinoline related to chloroquine and amodiaquine, but mechanisms of resistance are only partially shared with those agents. Mutations in two putative drug transporters, PfCRT and PfMDR1, are associated with decreased aminoquinoline sensitivity. For piperaquine,

associations are clearest with the *pfdhfr* 86Y mutation, which is selected by prior use of DP[59] and associated with decreased in vitro drug sensitivity.[60] In southeast Asia, resistance to piperaquine has emerged, associated with increased plasmepsin gene copy number[61,62] and a novel PfCRT mutation.[63] These newly identified polymorphisms are uncommon in Uganda, and piperaquine sensitivity and DP efficacy remain excellent.[60,64] Resistance to artemisinins has emerged in southeast Asia, mediated principally by mutations in the K13 gene propeller domain.[65] K13 mutations are uncommonly detected in Ugandan samples,[66] and these do not appear to be associated with altered in vitro sensitivity to DHA[67] or clinical drug resistance.[68] Overall, resistance to DP has not clearly been identified in Uganda, but it is very important to study potential selection of resistance by IPTp-DP.

Potential safety concerns for SP+DP. IPTp with SP has an excellent safety profile. Severe cutaneous reactions are well documented with SP, but are rare and primarily noted with long-term prophylaxis in non-African populations. DP also has an excellent safety profile, with similar risks of adverse events for IPTp with SP or DP.[27-29] The primary adverse effect noted with DP is dose dependent QT interval prolongation from piperaquine.[69] However, in clinical trials of IPTp with DP, QT interval prolongation was transient and not associated with arrhythmias or cardiovascular symptoms.[28,29,70] In a recent meta-analysis of almost 200,000 individuals who received DP, the risk of sudden unexplained death was not higher than baseline and the authors concluded that concerns about cardiotoxicity need not limit its use.[71] In pharmacokinetic studies, our group and others reported that pregnancy decreases piperaquine and sulfadoxine exposure, with mixed results for pyrimethamine, suggesting no pregnancy-associated increased risk, although data are limited.[72-76] Piperaquine is metabolized by CYP P450 3A45 and 2C8.[77-79] Sulfadoxine is cleared renally through glomerular filtration[80,81] and pyrimethamine is primarily hepatically metabolized, with 15–30% excreted unchanged in urine.[82] Available studies report no clinically relevant drug interactions for DP and SP, and there is no evidence that they alter the disposition of other drugs, except for one small study reporting increased exposure to halofantrine, a CYP P450 3A4 substrate, after a single dose of SP.[81,83-86]

Although no trials have studied SP+DP, we have shown that daily TMP-SMX+DP was as safe and well tolerated for IPTp as daily TMP-SMX alone among HIV infected pregnant women.[70] To confirm that administration of SP+DP does not result in clinically relevant changes in piperaquine exposure, we will evaluate exposure to piperaquine at the onset of our trial.[87]

1.2. Rationale

Malaria in pregnancy remains a major challenge in Africa, where approximately 50 million women are at risk for *P. falciparum* infection during pregnancy each year.[88] Among pregnant

women living in malaria endemic areas characteristic of much of Africa, symptomatic disease is uncommon, but infection with malaria parasites is associated with maternal anemia and adverse birth outcomes including abortions, stillbirth, preterm birth, LBW, and infant mortality.[2] Thus, malaria in pregnancy causes an estimated 900,000 LBW deliveries and 100,000 infant deaths each year.[3,89] The WHO recommends the use of LLINs and IPTp-SP for the prevention of malaria in pregnancy in endemic areas of Africa. However, there is concern for diminishing efficacy of these interventions due to the spread of vector resistance to the pyrethroid insecticides used in LLINs and parasite resistance to SP.[13,90] Thus, there is an urgent need for new strategies for the prevention of malaria in pregnancy and improving birth outcomes.

ACTs are now the standard treatment for malaria in Africa. DP is a fixed-dose ACT and an attractive alternative to IPTp-SP. DP is highly efficacious, and the long half-life of piperaquine provides at least 4 weeks of post-treatment prophylaxis.[91,92] Recent randomized controlled trials from our group and others have shown that, compared to IPTp with SP, IPTp with DP dramatically reduced risks of maternal parasitemia and symptomatic malaria, maternal anemia, and placental malaria.[27-29] In addition, IPTp with DP was as safe and well tolerated as IPTp with SP. Surprisingly, despite significant reductions in risks of malaria-specific outcomes afforded by DP in these studies, there were no differences between the SP and DP groups in risks of adverse birth outcomes, including LBW and preterm birth. Indeed, in 2015 a WHO Malaria Policy Advisory Committee concluded that, although IPTp with DP merits further study, SP should remain the recommended drug for IPTp until there is conclusive evidence that alternative regimens improve birth outcomes.[93] Thus, the standard-of-care remains use of a poorly effective antimalarial to prevent malaria in pregnancy.

The key question motivating this proposal is why IPTp with either SP or DP is associated with similar risks of adverse birth outcomes despite the far superior antimalarial activity of DP. The likely explanation is that SP, a broad-spectrum antibiotic, protects against non-malarial causes of LBW and preterm birth.[94] In this regard, several studies reported that increased frequency of doses of IPTp with SP was associated with improved birth outcomes and a lower risk of RTIs, despite widespread SP resistance among malaria parasites.[34,50] Our central hypothesis is that SP improves birth outcomes independent of its antimalarial activity and that IPTp with a combination of SP+DP will offer antimalarial and non-antimalarial benefits, thus providing superior prevention of adverse birth outcomes compared to either drug used alone. To test this hypothesis we will conduct a double-blinded randomized clinical trial in a rural area of Uganda with very high malaria transmission intensity where our group already has an established infrastructure for clinical research.

2. STUDY OBJECTIVES

We will test the hypothesis that IPTp with SP+DP will significantly reduce the risk of adverse birth outcomes compared to IPTp with SP alone or DP alone. The specific study objectives are as follows:

2.1. Objective 1

To compare the risk of adverse birth outcomes among pregnant women randomized to receive monthly IPTp with SP vs. DP vs. SP+DP. We will conduct a randomized controlled trial in 2757 women to test the hypothesis that pregnant women who receive IPTp with SP+DP will have a lower risk of a composite outcome of LBW, preterm birth, small-for-gestational age, fetal loss or neonatal death compared to those who receive either SP or DP alone.

2.2. Objective 2

To compare safety and tolerability of IPTp regimens among pregnant women randomized to receive monthly IPTp with SP vs. DP vs. SP+DP. We will test the hypothesis that pregnant women who receive IPTp with SP+DP will have non-inferior risks of adverse events and measures of tolerability compared to those who receive either SP or DP alone.

2.3. Objective 3

To compare risks of malaria-specific and non-malarial outcomes among pregnant women randomized to receive monthly IPTp with SP vs. DP vs. SP+DP. We will test the hypotheses that a) pregnant women who receive IPTp with DP containing regimens will have a lower risk of maternal and placental malaria but a higher risk of *P. falciparum* markers of DP resistance compared to those who receive SP alone; and b) pregnant women who receive IPTp with SP containing regimens will have a lower risk of RTIs and a higher risk of *P. falciparum* markers of SP resistance compared to those who receive DP alone. Further, we will explore impacts of study drugs on the gut and vaginal microbiomes, as differences are likely to impact upon birth outcomes.

3. STUDY DESIGN

This will be a double-blinded randomized controlled phase III trial of 2757 HIV uninfected pregnant women. HIV uninfected women at 12-20 weeks gestation will be randomized in equal

proportions to one of three IPTp treatment arms: 1) SP given every 4 weeks, or 2) DP given every 4 weeks, or 3) SP+DP given every 4 weeks. SP or DP placebos will be used to ensure adequate blinding is achieved in the study as outlined in appendix D. Follow-up for the pregnant women will end 28 days after giving birth.

We will recruit pregnant women presenting for routine care at antenatal clinics at 9 government health facilities in Busia District, including Masafu General Hospital. Based on our previous study we estimate that ~200 women will be available for screening each month. Recruitment will take place in two phases. In phase 1, 300 participants will be enrolled over 2-3 months. Phase 1 participants will undergo intensive safety and pharmacokinetic assessment. In phase 2, the remaining 2457 participants will be enrolled. After all participants enrolled in phase 1 have delivered, an interim analysis of safety data will be performed and presented to the DSMB as described in detail in the Protection of Human Subjects section and study protocol. If the DSMB approves, phase 2 of recruitment will continue until the target sample size has been reached.

4. SELECTION AND ENROLLMENT OF SUBJECTS

4.1. Inclusion Criteria

1) Viable singleton pregnancy confirmed by ultrasound
2) Estimated gestational age between 12-20 weeks
3) Confirmed to be HIV uninfected by rapid test
4) 16 years of age or older
5) Resident of Busia District, Uganda
6) Provision of informed consent
7) Agreement to come to the study clinic for any febrile episode or other illness and avoid medications given outside the study protocol
8) Willing to deliver in the hospital

4.2. Exclusion Criteria

1) History of serious adverse event to SP or DP
2) Active medical problem requiring inpatient evaluation at the time of screening
3) Intention of moving outside of Busia District, Uganda
4) Chronic medical condition requiring frequent medical attention
5) Prior chemopreventive therapy or any other antimalarial therapy during this pregnancy
6) Early or active labor
7) Multiple pregnancies (i.e. twins/triplets)

8) History of long QT syndrome

4.3. Initial Screening

We will recruit pregnant women presenting for routine care at the Masafu General Hospital antenatal clinic, presenting at local health centers within Busia District, or referred by the Uganda government voluntary health teams. Pregnant women will be approached about participating in the study and will be provided an information sheet about the requirements of the study (Appendix A). If women are initially agreeable to screening for participation in the study and are $\leq$ 20 weeks gestation by LMP, 16 years of age or older, and not known to be HIV infected, they will either be escorted to the study clinic or given an appointment to return at a later date. At our study clinic, study physicians will assess for initial eligibility criteria through conversation with the woman (confirming the age of the woman, location of residence, agreement to come to the study clinic for any febrile episode or other illness and avoid medications given outside the study protocol, plan to deliver in the hospital, no history of serious adverse event to SP or DP, no active medical problems requiring inpatient evaluation at the time of screening, no intention of moving outside the study district, no chronic medical condition requiring frequent medical attention, no prior SP preventive therapy or any other antimalarial therapy during this pregnancy, and no history of long QT syndrome). Women who pass initial screening based on conversation with study physicians will undergo the following additional screening procedures: 1) rapid HIV testing to confirm HIV negative status, 2) ultrasound dating to confirm viable singleton pregnancy and gestational age of 12-20 weeks in conjunction with LMP (Appendix B), and 3) a pelvic examination to exclude early or active labor if clinically indicated. Women who are not eligible for the study will be referred back to their local antenatal clinic.

4.4. Study Enrollment Procedures and Baseline Evaluation

Informed consent will be obtained from women who pass initial screening. Study physicians will conduct the informed consent discussion in the study clinic. Informed consent will be conducted in the appropriate language and a translator will be used if necessary. The study will be described and consent obtained in one of 4 languages (Samia, Swahili, Luganda, or English). The consent forms will be translated into each language and back-translated into English to check for any loss or change of meaning. Following the informed consent discussion, pregnant women will be asked by the study physicians to sign a written consent form approved by the UCSF CHR, Makerere University School of Biomedical Sciences - Research and Ethics Committee, and the UNCST for their participation in the research study and a second approved consent form for the future use of biological specimens obtained during the course of the

study. If the pregnant woman is unable to read or write, her fingerprint will substitute for a signature, and a signature from an impartial witness to the informed consent procedures will be obtained.

Women will be enrolled in the study on the same day that they provide informed consent for participation in the research study. On the day of enrollment, women will undergo a standardized history and physical examination, and have blood collected by venipuncture (15 cc's) for filter paper sample (for future molecular studies), routine baseline laboratory testing and storage. Routine baseline laboratory testing will consist of a thick blood smear for malaria parasites (to be read later by laboratory technicians not involved in study participant care; will not be used to guide clinical care) and CBC measurement.

Subjects will also provide samples for gut and vaginal microbiome studies. A clinician will collect a vaginal swab for microbiome analysis in a private area. The clinician will use an Endocervical Specimen Collection Kit, inserting the vaginal swab approximately 4-5 cm into the vagina for the collection. The swab will then be placed into the collection container and frozen in liquid nitrogen.

The study participant will be provided with a stool collection container and can provide the stool sample either at the clinic or at home. If the sample is to be collected at home, the participant will be asked to bring the sample back to the clinic as soon as possible. The laboratory procedures for microbiome analysis are described in Section 6.10.7.

Women who have history of fever in the previous 24 hours or a temperature $\geq$ 38.0ºC (tympanic) will have a thick blood smear read urgently in the study clinic. Women with history of fever in the previous 24 hours or a temperature $\geq$ 38.0ºC (tympanic) and a positive blood smear will be diagnosed with malaria and treated as described in section 6.2. At the end of the enrollment visit all study participants will be given a LLIN and a household survey appointment will be scheduled within 2 weeks to collect household-level information on the use of bednets, house members, household characteristics, and GPS coordinates (Appendix C).

5. STUDY TREATMENT

5.1. Treatment Group Assignments

An enrolled pregnant woman will be randomized to one of 3 treatment arms: SP given every 4 weeks, DP given every 4 weeks or the combination of SP+DP given every 4 weeks. We will use a 1:1:1 randomization scheme targeting 919 pregnant women in each treatment arm. A randomization list will be computer generated by a member of the project who will not be directly involved in the conduct of the study. The randomization list will include consecutive treatment numbers with corresponding random treatment assignments. Randomized codes will correspond to the 3 treatment arms using permuted variable sized blocks of 6 and 9. Sealed

copies of the original randomization list and documentation of the procedure used to generate the lists will be stored in the project administrative offices in San Francisco and Kampala. Prior to the onset of the study, a set of sequentially numbered, opaque, sealed envelopes will be prepared. Each envelope will be marked on the outside with the treatment allocation number. The inside of the envelope will contain a piece of paper with the treatment allocation number and treatment group assignment along with a piece of carbon paper.

5.2. Treatment Allocation

On the day of enrollment, pregnant women will be referred to a study pharmacist responsible for treatment allocation. The study pharmacist will assign treatment arms as follows:

1. Select next available envelope
2. Note treatment number on the outside of the envelope
3. Write date, time, and study number on the outside of the envelope
4. Open envelope
5. Remove form containing code for treatment arm and date, time, and study number (transferred to form via carbon paper inside of envelope)
6. Store form in lockable file box in study pharmacy
7. Record onto the treatment allocation master list the study number, enrollment date, treatment assignment code, treatment arm, and study medications to be given during pregnancy
8. Store treatment allocation Master list in a lockable cabinet in study pharmacy
9. Record treatment number in the study participant's file

5.3. Study Drug Dosing and Formulations

During pregnancy, women will be given 1 of 3 treatment regimens: 1) SP given every 4 weeks during pregnancy, 2) DP given every 4 weeks during pregnancy, or 3) SP+DP given every 4 weeks during pregnancy. Each treatment with SP will be given as a single dose consisting of 3 full strength tablets. Each treatment with DP will consist of 3 full strength tablets given once a day for 3 consecutive days. In addition, placebos will be used to mimic the identical dosing strategy such that every 4 weeks women will receive two drugs on day 1 (SP and placebo or DP and placebo or SP and DP) followed by one drug on days 2 and 3 (DP or placebo). Two placebos will be used, one that mimics the appearance of SP and one that mimics the appearance of DP. Dosing schedules for each treatment regimen according to gestational age are presented in Appendix D. The study drugs, DP (Duo-Cotecxin) and SP (Kamsidar) will be supplied by Holley-

Cotec, Beijing and Kampala Pharmaceutical Industries (KPI), Uganda, respectively. Holley-Cotec will manufacture the DP placebo of exact specifications as that of the active DP. The DP placebo will be exactly the same in color, size, shape and packaging as the active DP. KPI will manufacture the SP placebo of exact specifications as that of the active SP. The SP placebo will be exactly the same in color, size, shape and packaging as the active SP. Details of the study drug formulations are included in Table 2.

Table 2. Drug formulation and labeling

Drug	Formulations	Trade name (Manufacturer)
Sulfadoxine-Pyrimethamine (SP)	500mg/25mg tabs	Kamsidar (KPI)
Dihydroartemisinin-Piperaquine (DP)	40mg/320mg tabs	Duo-Cotexin (Holley-Cotec)

5.4. Blinding, Study Drug Administration, and Duration

Administration of all study drugs will be double blinded such that study participants and study staff will be blinded to study treatments with the exception of the study pharmacist and pharmacy technician, who will not be involved with patient care or assessment of study outcomes. All doses of study drugs will be prepackaged by a study pharmacist and administered by a study nurse blinded to the study participant's treatment regimen. All doses of SP (or SP placebo) administered will be directly observed in the clinic. For DP (or DP placebo), the first of the 3 daily doses will be directly observed in the clinic and the 2nd and 3rd daily doses will be administered at home using pre-packaged study drugs in opaque envelopes with dosing instructions written on the outside. For doses of study drugs administered in the clinic, if a study participant vomits the study drug within 30 minutes of administration, the drug will be re-administered. For doses of study drugs administered at home, if a study participant vomits the study drug within 30 minutes of administration or study drug is lost, the study participant will be instructed to come to the study clinic as soon as possible where the study drug will be re-administered/replaced. For pregnant women all doses of study drugs will be given between 16 and 40 weeks gestation as outlined in Appendix D.

5.5. Study Drug Accountability

The study pharmacist will maintain complete records of all study drugs received in the study pharmacy. Lot number and number of pills given to each study participant will be recorded. A registry of all study medication, current product labels, and Certificates of Analysis, provided by suppliers will be maintained within the regulatory binder for the study. The date received, lot number, expiration date, and date used will be recorded for each of the study medications. Monthly inventory of all study medications will be conducted and a record log of investigational medications will be kept at the study clinic.

6. SUBJECT MANAGEMENT

6.1. Subject Follow-up

Pregnant women will be scheduled to be seen in the clinic every 4 weeks during their pregnancy and then 1 and 4 weeks following delivery. In addition, study participants will be instructed to come to the study clinic for all medical care and avoid the use of any outside medications. The study clinic will remain open 7 days a week from 8 a.m. to 5 p.m. Pregnant women who are not seen on the day of their regularly scheduled visits will be visited at home and instructed to come to the clinic as soon as possible.

Each time a study participant is seen in the clinic a standardized history and physical exam will be performed including temperature, pulse, weight, mid-upper arm circumference, and blood pressure measurement. Patients who are febrile (tympanic temperature $\geq$ 38.0˚C) or report history of fever in the past 24 hours will have blood obtained by finger prick for a thick blood smear and have fetal ultrasounds performed. If the thick blood smear is positive, the patient will be diagnosed with malaria (Section 6.2). If the thick blood smear is negative, the patient will be managed by study physicians for a non-malarial febrile illness (Section 6.3). If the patient is afebrile and does not report a recent fever, a thick blood smear will not be obtained, except when following routine testing schedules (Section 6.5).

6.2. Diagnosis and Management of Malaria

Patients found to have malaria based on laboratory confirmation will have a second finger prick for hemoglobin measurement using a portable spectrophotometer (HemoCue), and collection of plasma. Patients with malaria may be asked to donate 5 cc of blood before treatment is initiated to allow studies of cultured malaria parasites. All episodes of malaria will be classified as uncomplicated or complicated based on the following criteria:

Uncomplicated malaria (all of the following)

1) Fever ($\geq$ 38.0ºC tympanic) or history of fever in the previous 24 hours
2) Positive thick blood smear
3) Absence of complicated malaria

Complicated malaria

1) Clinical evidence of severe malaria (Appendix E)
2) Positive thick blood smear

Episodes of malaria will also be classified into the following categories according to the timing of previous malaria episodes for treatment purposes:

1. New episodes of malaria will be defined as any first episode during the study period or any episode occurring > 14 days after the diagnosis of a previous episode
2. Treatment failures will be defined as any of the following:
    a. Complicated malaria occurring 1-14 days after the diagnosis of malaria
    b. Fever ($\geq$ 38.0°C tympanic) or history of fever in the previous 24 hours with a parasite density $\geq$ the parasite density of an episode of malaria diagnosed 2 days prior
    c. Fever ($\geq$ 38.0°C tympanic) or history of fever in the previous 24 hours with a parasite density $\geq$ 25% of the parasite density of an episode of malaria diagnosed 3 days prior
    d. Fever ($\geq$ 38.0°C tympanic) or history of fever in the previous 24 hours with a positive thick blood smear of any parasite density occurring 4-14 days after the diagnosis of a previous episode

All patients diagnosed with new episodes of uncomplicated malaria will be prescribed AL, the recommended first-line treatment in Uganda for pregnant women. Patients with complicated malaria will be prescribed IV artesunate according to national malaria treatment guidelines. Patients with treatment failure within 14 days following treatment with AL will be prescribed pyronaridine-artesunate (Pyramax) according to national malaria treatment guidelines. Patients with treatment failure within 14 days following treatment with Pyramax will be treated with quinine. Patients with treatment failure within 14 days following treatment with quinine will be treated with quinine plus clindamycin. Treatment of symptomatic malaria will have no impact on the administration of study drugs for IPTp.

6.3. Management of Non-Malaria Illnesses

Patients who are found to have illnesses other than malaria will receive standard-of-care treatment in the study clinic, according to standardized algorithms, or will be referred to the local hospital. We will avoid the routine use of non-study medications with antimalarial activity, including antifolates (with the exception of assigned IPTp regimens) and macrolide or tetracycline antibiotics, when acceptable alternatives are available. In addition, drugs with known risk of torsades de pointes or CYP3A4 inhibitors (Appendix J and K) will be avoided if possible. If the study clinician deems that treatment with one of the drugs listed on Appendix J or K is required, the clinical management team will be consulted. During follow-up for non-malarial illnesses, blood smears will be done at the discretion of the study physician if the subjects are febrile (tympanic temperature $\geq$ 38.0˚C) or report history of fever in the past 24

hours. If the blood smear is positive, the patient will be diagnosed with a new episode of malaria and managed per study protocol. We will be assessing for RTIs at delivery. For 3 infections for which treatment is clinically indicated (*C. trachomatis*, *N. gonorrhea*, and *T. vaginalis*), women will be treated promptly, generally within one day of delivery, following standard guidelines for the treatment of these infections.

6.4. After Hours Visits

Pregnant women will be encouraged to come to the Masafu General Hospital maternity ward (open 24 hours a day) when urgent care is needed outside of study clinic hours. Pregnant women will be instructed to inform hospital personnel of their involvement in the study at the time of registration and to visit the study clinic on the following day. If a patient is diagnosed with uncomplicated malaria she will receive treatment from a hospital supply of AL and clinicians will be instructed to refer patients to our study clinic when it opens at 8 am the following day. If a patient is diagnosed with severe malaria, she will receive I.V. artesunate following standard treatment guidelines. Patients with non-malarial illnesses will be managed at the discretion of the hospital staff. Upon discharge, patients will receive follow-up at the study clinic as outlined above.

6.5. Routine Assessments

Routine assessments will be done in the clinic every 4 weeks (Appendix G). Study participants not seen in the clinic for routine visits will be visited at home and requested to come to the study clinic as soon as possible. Pregnant women will receive standards of care as designated in the Uganda MOH guidelines (Appendix F). Routine antenatal care will include screening and treatment for sexually transmitted infections; blood pressure assessment; urine dipstick for proteinuria; and prescription of iron, folate, multivitamins and mebendazole. During routine assessments subjects will be asked about visits to outside health facilities and the use of any medications outside the study protocol. The study protocol will be reinforced with discussion regarding the need to come to the study clinic promptly upon the onset of any illness and to avoid use of outside medications. Standardized assessment of adherence will also be done for study drugs administered at home and LLIN use. A routine history and physical exam will be performed using a standardized clinical assessment form. Blood will be collected by finger prick or phlebotomy (alternate routine visits) for thick smear, qPCR testing, CBC testing, immunology studies, and filter paper samples as described in Appendix G. Thick smears, qPCR testing, and collection of filter paper samples will be done every 4 weeks. Phlebotomy for CBC, for other laboratory tests to monitor for potential adverse events from study medications, for

immunology studies, and to collect blood for storage will be performed every 8 weeks. For participants enrolled between 12-<18 weeks gestational age, blood will be collected by phlebotomy at the time of their week 20 routine visit. For participants enrolled between 18-20 weeks gestational age, blood will be collected by finger prick at the time of their week 20 routine visit. A repeat HIV test will be done at 28 weeks gestational age. If women are found to have become HIV-infected during pregnancy, they will be immediately referred for care following local prevention of mother-to-child transmission guidelines. If a pregnant woman reports a fever in the last 24 hours or the patient has a documented temperature ≥ 38.0˚C tympanic, the patient's thick blood smear will be read immediately and if positive the patient will be diagnosed and treated for malaria (see section 6.2). Thick blood smears other than those done when a woman has fever will not be used for clinical care of study participants. Study drugs will be administered at the time of each routine visit as described in sections 5.3 and 5.4.

A pregnancy ultrasound will be conducted at 24, 32 and 36 weeks gestational age to assess fetal size. Images and videos clips from the ultrasound will be saved, and read by trained study staff members who are not directly involved in patient care. A stool sample and a clinician collected vaginal swab for microbiome studies will also be collected at 32 weeks gestation, using similar procedures as at enrollment (Section 4.4, Section 6.10.7). If the study participant misses their 32 week appointment or delivers before the stool and vaginal samples can be collected, these samples will be collected as soon as possible after 32 weeks.

For the 300 women enrolled in phase 1 of the study the following additional procedures will be conducted at 20, 28, and 36 weeks of gestation: 1) the administration of all 3 daily doses of study drugs will be directly observed in the clinic. 2) ECGs will be performed to measure the QTc interval just prior to the first dose and 2-6 hours after the third dose of study drugs. 3) Blood will be collected by phlebotomy just prior to performing each ECG to measure trough and peak piperaquine levels. These data will be used to assess for potential cardiotoxicity, impacts of SP on peak piperaquine drug levels, and other potential adverse events (which will be rigorously assessed in both phase 1 and phase 2).

In a subset of women actively followed in the study after October 1st 2023, an additional stool sample will be collected 1 week after their week 28 gestational age routine visit to better assess potential changes to the stool microbiome shortly after peak concentrations of study drugs. Participants will be given a stool collection vial at the time of their week 28 gestational age routine visit with instructions to collect a stool sample at home 1 week later and then bring it as soon as possible to the study clinic. Participants will be provided transport reimbursement when they bring this stool sample to the study clinic but will not need to be seen by the study clinicians or have any additional evaluations. This additional stool sample will be processed and analyzed as described in Section 6.10.7

6.6. Delivery visit

Systems will be in place to facilitate a birth plan which will encourage women to come to the hospital for delivery, including access to transportation 24 hours a day. However, for women who are unable to travel to the hospital for delivery or choose to deliver at home, a study staff member will be driven to the home to follow study procedures. Study staff will document details of the delivery, including date and time, type of delivery, blood pressure during labor, estimated blood loss and any maternal, obstetrical or neonatal complications. Study staff will document the infant's Apgar score and birth weight with calibrated scales. Biological samples collected at the time of delivery will include maternal venous blood (for thick blood smear, filter paper samples, CBC, and storage of blood for immunology and other studies), cord blood (for thick blood smear, filter paper samples, hemoglobin measurement, and storage of blood for immunology and other studies), and placental tissue. A vaginal swab will be collected for the diagnosis of common infectious diseases (section 6.10.8). The GeneXpert/Endocerivcal Collection Kit will be used to collect the sample. The clinician will insert the swab 4-5 cm into the vagina, making a circular motion along the vaginal wall. If any infections are diagnosed, the participant will receive appropriate treatment after delivery. Following delivery, neonatal care, as per national guidelines, will include polio and BCG immunization, ophthalmic tetracycline, and vitamin K.

At the time of delivery, women will undergo repeat rapid HIV testing based on national guidelines. If women are found to have become HIV-infected during pregnancy, they will be immediately referred for care following local prevention of mother-to-child transmission guidelines.

6.7. Postpartum visits.

Women will be evaluated 1 and 4 weeks following delivery as part of routine care. Both visits will include an abdominal exam, syndromic management of RTIs, and follow-up on any obstetrical complications that occurred including evaluation of the neonate for any congenital abnormalities. Pelvic and breast exam will be done if clinically indicated. Contraceptive counseling, nutritional assessment and infant feeding and support will be provided.

Summaries of all procedures done during routine visits in pregnant women are presented in Appendix G.

6.8. Medical Care Outside the Study Clinic

We will provide routine medical care in our clinic free of charge, including medications, to the extent possible given resources available. Study participants will be reimbursed for costs of transportation to and from our clinic. In addition, we will reimburse the cost of tests and drugs not available in the study clinic, referrals for study participants made by study physicians to other clinics and services, and after-hours hospital visits. We anticipate reimbursing the cost of most diagnostic tests (including laboratory tests, X-rays, and ultrasounds) and medications resulting from these referrals, using available funds. However, reimbursement of all diagnostic tests and treatment recommended outside the study clinic cannot be guaranteed in all circumstances. Decisions on reimbursement will be made by the study coordinator and the investigators, in conjunction with the funding agency if necessary.

6.9. Duration of Follow-up and Criteria for Premature Study Withdrawal

Study participants will be followed until their 4 week postpartum visit. Study participants will be prematurely withdrawn from the study for: 1) movement out of study area, 2) inability to be located for > 60 consecutive days, 3) withdrawal of informed consent, 4) inability to comply with the study schedule and procedures, 5) at the discretion of the site investigator if the study is not in the best interest of the participant, 6) subject judged by the site investigator to be at significant risk of failing to comply with the study protocol as to cause them harm or seriously interfere with the validity of study results, and 7) subject found to be HIV positive at the time of their 28 week gestational age routine visit. If a subject is withdrawn for reasons # 1-3, we will be unable to perform any additional study procedures. If a subject is withdrawn for reasons # 4-7, plans to obtain appropriate follow-up tests outside of the study will be individualized for each subject depending on the health status of the subject at the time of withdrawal and the willingness of the participant and his or her parent/guardian to proceed with additional testing.

6.10. Diagnostic and Laboratory Testing

Diagnostic and laboratory testing shall take place using accredited local laboratories, as described below. In the event that local capacity is not available for specialized testing, reasonable efforts will be made to build local capacity. In the event that local capacity is not available, samples will be exported with all required permissions and permits to external testing laboratories.

6.10.1. Microscopy

Thick blood smears will be stained with 2% Giemsa and read by experienced laboratory technologists. Parasite densities will be calculated by counting the number of asexual parasites

per 200 leukocytes (or per 500 leukocytes, if the count is <10 asexual parasites/200 leukocytes), assuming a leukocyte count of 8,000/µl. A blood smear will be considered negative when the examination of 100 high power fields does not reveal asexual parasites. Gametocytemia will also be determined from thick smears. For quality control, all slides will be read by a second microscopist and a third reviewer will settle any discrepant readings.

6.10.2. Clinical Laboratory Studies

At enrollment and every 8 weeks during follow-up for pregnant women venipuncture blood samples will be collected for CBC testing. Additional venipuncture will be performed, as appropriate, for laboratory testing to evaluate non-malarial medical illnesses at the discretion of study physicians. Results will be made available to study physicians in a timely manner for patient management decision-making. Additional hemoglobin measurements will be performed each time a patient is diagnosed with malaria using a portable spectrophotometer (HemoCue, Angholm, Sweden), and results will be immediately available.

6.10.3. Placental Studies

For assessment of placental malaria, two 1 cm-wide full thickness biopsies, obtained about 5 cm from the cord, will be obtained within 1 hour of delivery and placed in 10% neutral buffered formalin. Biopsy specimens will be embedded in paraffin wax, sectioned into 3 µm slices using a rotary microtome, fixed to glass slides, and dehydrated in sequential ethanol baths. Separate slides will be stained in 0.1% hematoxylin/1% eosin (H&E) for 5 and 1 min, respectively, or in 2% Giemsa for 30 min. Placentas will be graded into 5 categories using a standardized approach.[95] The presence of intervillous parasite-infected erythrocytes and of pigment in monocyte/macrophages or fibrin will be noted. Quantitative assessments of placental malaria will involve counting of 1000 intervillous blood cells under high power and determination of percentages of intervillous infected erythrocytes and monocyte/macrophages containing malarial pigment. Additional placental biopsies may be taken and preserved for genomic analysis. Briefly, any additional placental biopsies will be preserved in RNAlater (an RNA preservation agent) and stored per manufacturer’s directions, and/or flash frozen in liquid nitrogen and embedded in OCT mounting medium. For assessment of chorioamnionitis, placental membrane roll, umbilical cord, and chorionic plate/villous parenchyma specimens will be collected, dehydrated through an ethanol series, cleared in xylene, and embedded in paraffin wax blocks. A 3 µm thick section from each tissue block will be obtained using a rotary microtome, and sections will be mounted onto glass slides via a floatation water bath. The slides will be baked at 60°C for 30 min, de-paraffinized in xylene, hydrated through a series of ethanols, stained with H&E, dehydrated through an ethanol series, cleared with xylene, and

mounted with organic media. Slides will be examined by light microscopy, and chorioamnionitis diagnosed based on detection of neutrophils, based on a standardized diagnostic framework.[96] In addition, the chorioamniotic space will be sampled for genomic assessment; after delivery of the placenta, the chorion and amnion will be peeled apart,[97] taking care to avoid contamination, the exposed surface will be swabbed, the swab placed in PBS, and sample flash frozen in liquid nitrogen for shipment and processing as described in Section 6.10.7. Of note, our group already has expertise in all described techniques for studying placental histopathology.[98] Cord blood and placental intravillous blood will be collected for immunology studies as described below.

6.10.4. Dried blood spots collected on filter paper

Each time a thick blood smear is obtained blood will also be collected onto filter paper. Samples will be collected by venipuncture or by finger-prick sampling. Filter paper (Whatman no 1, Whatman 3MM; Whatman, Maidstone, UK) will be pre-cut into individual squares and stapled to a thick card which will serve as its cover. Blood spots will be collected onto the filter paper in volumes of approximately 25 µl per blood spot (4 blood spots per sample). Filter paper samples will be labelled with bar codes on the covering cardboard and will be allowed to dry at ambient temperature and relative humidity before closing the card over the filter paper (like closing a matchbook). Filter paper samples will be transported from the field in zip lock bags and placed into a stock card filter paper box for final storage at ambient temperature or at -20°C with a desiccant. Filter paper samples will be stored in Tororo or a central laboratory in Kampala. Molecular studies will include the extraction of DNA and/or RNA from filter paper, followed by characterization of parasite and host nucleic acid sequences using standard molecular procedures including PCR, DNA hybridization, restriction enzyme digestion, and/or nucleic acid sequencing. Future molecular studies will be performed only for research purposes and will have no impact on the clinical management of study participants.

6.10.5. Quantitative PCR

qPCR will be performed at the time of enrollment and at each routine visit (every 4 weeks). At each of these time points, 200 µL of whole blood will be used and DNA will be extracted using Qiagen spin columns or related methods. Extraction products will be tested for the presence and quantity of *P. falciparum* DNA via a highly sensitive qPCR assay targeting the multicopy conserved *var* gene acidic terminal sequence.[99] This assay is now routinely performed by our group and demonstrates sensitivity and reproducible quantification to 0.1 parasites/µL. qPCR

for other potential pathogens may also be performed. These assays will be performed at our laboratory in Tororo where qPCR capacity is already established.

6.10.6 Ex vivo drug sensitivity of malaria parasites

Blood will be collected from select subjects upon diagnosis of malaria for culture of infecting malaria parasites. After consent is obtained for this procedure, up to 5ml of blood will be drawn by venipuncture and collected in heparin containing tubes for spotting on filter paper and ex vivo culture of malaria parasites. Blood will be transported to our laboratory at Tororo District Hospital and drug susceptibilities assessed. Samples will be processed according to our previously published protocols, with detection of parasites based on SYBR green fluorescence or related methods.[59,60] In brief, blood will be centrifuged, plasma and buffy coat removed, and erythrocyte pellets washed three times with RPMI 1640 medium. 200 µl aliquots of washed pellets will be added to 10 ml of RPMI 1640 medium supplemented with 25 mM HEPES, 0.2% NaHCO3, 0.1 mM hypoxanthine, 100 µg/ml gentamicin, 0.5% AlbuMAX II serum substitute, and serial dilutions of drugs of interest. Drug sensitivities will be determined with a 96-well microplate fluorescence assay with SYBR green I detection. In brief, plates will be maintained for 72h in a modular incubator, plates will be frozen and thawed, 100_µl from each well will be transferred to a black 96-well plate containing 100 µl/well SYBR green lysis buffer, plates will be incubated for 1 h in the dark at room temperature, and fluorescence will be measured with a plate reader. IC50s will then be derived by plotting percent growth against log drug concentration, and fitting the data by variable slope, sigmoidal curve fit. Studies of ex vivo drug sensitivities will have no impact on patient care.

6.10.7 Microbiome studies

We will comprehensively compare the gut and vaginal microbiomes between our 3 treatment arms. We will begin with multiplex 16S rRNA gene amplicon sequencing of gut and vaginal microbiota and then advance to metagenomics sequencing to provide greater depth of analysis.

Collection of samples for microbiota evaluation. Care is required to avoid undue degradation or contamination of samples, but studies of gut and vaginal microbiota have shown rather little impact of short term (2 weeks) storage, even at room temperature.[106,107] All samples will be handled in the same manner, as inconsistent methods of collection and storage can impact on results[106]. Samples will be collected near the time of study enrollment (before first dose of study drug(s)) and at approximately 32 weeks gestation, before the scheduled monthly dose of study drug(s). We have chosen the 32 weeks gestation for the second time point because the majority of women, including those with preterm deliveries at <37 weeks gestation, will deliver after 32 weeks. Thus we will avoid collecting samples for microbiome studies during labor or

immediately after delivery, which may be contaminated. At enrollment and 32 weeks gestation, stool and vaginal swabs will be collected as described in section 4.4. Clinician collected rather than self-collected vaginal swabs were selected to reduce variability in longitudinal sample collection technique. Samples (~ 1 ml stool and vaginal swab tips) will be placed in storage containers, labelled, snap frozen, and stored in liquid nitrogen. Liquid nitrogen is continuously available at Masafu General Hospital, delivered as needed from a supplier in Iganga, Uganda. We will take standard precautions to avoid contamination between samples or from the environment. However, samples with high concentrations of microbes, such as stool and vaginal samples, are less subject to misrepresentation of results due to contamination than are samples with low microbial concentrations.[106]

Preparation of samples. Stool and vaginal swab samples will be shipped in liquid nitrogen to UCSF. For processing, samples will be thawed, placed promptly into lysis buffer (Wizard SV 96 Genome DNA kit, Promega), and homogenized by beating for 5 min with beads of mixed size. The samples will then be centrifuged for 10 min, supernatants transferred to kit plates, and DNA purified according to kit instructions.

Absolute abundance of bacterial DNA. For stool specimens, qPCR will be carried out in triplicate 10µL reactions with 200 nM 340F/514R primers with SYBRSelect for CFX Master Mix (Life Technologies). Absolute quantifications will be determined based against a standard curve of amplified *E. coli* MG1655 DNA,[108] adjusting for dilutions during DNA extraction, normalization, and PCR reaction preparation, and dividing by the total fecal mass used for DNA extraction. Differences in microbiota between samples from the 3 study arms will be assessed using non-parametric tests (Wilcoxon rank sum) and validated, in a representative subset of samples, by plating bacteria under anaerobic conditions and counting colony forming units and/or by counting cells with FACS, in both cases using established protocols.[109]

Relative abundance of bacteria. To quantify relative abundances of bacterial taxonomic groups in stool and vaginal samples, variable region 4 rRNA will be amplified with barcoded 515F (GTGCCAGCMGCCGCGGTAA)/806R (GGACTACHVGGGTWTCTAAT) primers according to the methods of the Earth Microbiome Project (earthmicrobiome.org). Amplicons will be quantified with PicoGreen, pooled at equimolar concentrations, and purified. Libraries will then be quantified and sequenced with a 600 cycle MiSeq Reagent Kit (Illumina) with ~10% control PhiX DNA. Samples will be pooled such that the average number of reads/sample is >20,000.

Relative abundance of fungi. Quantification of fungi may be particularly relevant, as by depleting bacteria SP may encourage fungal overgrowth. We will amplify the internal transcribed spacer (ITS), a region of the nuclear ribosomal RNA cistron shown to enable

successful identification across a broad range of fungal taxa.[109,110] Barcoded amplicons will be cleaned, quantified, and pooled to achieve equal amounts of DNA per sample, as for 16S analysis. We will select gel purified 150-500 bp amplicons for sequencing, with ~10% PhiX DNA, on the MiSeq platform. Taxonomic assignments of ITS sequencing reads will be performed using the UNITE ITS database (unite.ut.ee) and an in-house database of curated ITS1 sequences from human-associated fungi.[109]

Informatic analysis of 16S and fungal microbiota. Informatic methods will be based on those optimized by the Turnbaugh lab. Reads will be demultiplexed using QIIME[111] before denoising and processing with DADA2.[112] Taxonomy will be assigned using the DADA2 implementation of the RDP classifier using the DADA2 formatted training sets for SILVA123.[113] A phylogenetic tree will be constructed using MUSCLE and MacQIIME using the FastTree algorithm with midpoint rooting. Samples with <10,000 reads will be filtered out. Diversity metrics will be generated using Vegan and Phyloseq, with principal coordinate analysis (PCoA) carried out with Ape. Principal component analysis (prcomp; PCA) will be carried out on centered log2-ratio normalized genus abundances calculated as $A_{clr}=[\log_2(A_1/g_a), \log_2(A_2/g_a),\ldots \log_2(A_n/g_a),]$, where A is a vector of read counts with a prior of 0.5 added and g_a is the geometric mean of all values of A. Cross sectional analysis of significant features will be carried out using ALDEx2[114] using 256 simulations and Benjamini-Hochberg corrected (for false discovery) Wilcoxon *p*-values (paired where appropriate; FDR<0.1 used to indicate statistical significance). Gene content based on 16S rRNA gene sequencing data will be inferred using PICRUSt.[115] KEGG Pathway enrichment will be carried out using clusterProfiler.[116] Characterization of microbiota will enable comparison of gut and vaginal microbiota between members of different study arms and, within individuals, evaluation of changes in microbiota over the course of pregnancy. These comparisons will allow us to test the hypothesis that study regimens will differentially impact on microbiota, thereby affecting birth outcomes.

Metagenomics. Metagenomic assessment (full genome sequencing) has multiple advantages over 16S analysis, including improved reliability, more in-depth analysis of bacterial species and strains, identification of other organisms (viruses and eukaryotic pathogens), and identification of specific features of interest in addition to taxonomic analysis (e.g. presence of drug resistance genes). However, due to simplicity and low cost, 16S analysis is still standard in microbiome analyses. Due to our cost considerations 16S bacterial and ITS fungal profiling will be conducted in years 2-4, and metagenomics assessment primarily in year 5 of this project. However, rapid reductions of costs for deep sequencing may enable earlier initiation of these studies. Aliquots of DNA analyzed by 16S/ITS sequencing will be fragmented by sonication and prepared for sequencing using the Illumina TruSeq PCR-free kit using our established protocols.[109,117] Barcoded libraries will be pooled into groups of 10, and each pool will be

sequenced on a single lane of the Illumina HiSeq 2500 platform (or alternative platforms, as appropriate over time) at the UCSF Genomics Core Facility (15-20 Gbp per sample). Quality control will be performed in fastq-mcf,[109,117] and host reads removed by mapping to the human genome with Bowtie.[118] Filtered reads will be mapped to the KEGG database[119] using RAPSearch2.[120] Length-normalized read counts will be tallied for each KEGG orthology, with the exclusion of reads mapping to metazoans. Adjustment for average genome size will be done with MicrobeCensus.[121] Differential abundance analysis will be done via the limma package in R, using precision weights obtained with voom.[122] The differential abundance of KEGG orthologies will be further summarized at the module level using CAMERA.[123] Hierarchical clustering will be performed using the “hclust” function in R. Multiple testing will be adjusted by controlling for false discovery rate. Shotgun metagenomic sequencing data will be further leveraged to identify bacterial species and strains using Metaphlan2[124] and ConStrains.[125] As noted above for 16S and ITS analyses, metagenomic characterization will enable more in depth testing of our study hypothesis and also allow exploratory studies of impacts of study arms on specific features of microbiota including specific strains of interest, drug resistance determinants, and non-bacterial microbiota.

6.10.8 Reproductive tract infections (RTIs)

To test the hypothesis that study interventions will offer differential impact upon RTIs, enrolled subjects will be evaluated for RTIs upon delivery. The following tests, based on state-of-the-art technology, will be conducted. Vaginal swab samples will be stored at -80°C, enabling additional molecular studies, eg for *Mycoplasma*, *Ureaplasma, Candida,* or other pathogens if deemed relevant over the course of the project.

Sample collection for RTI analysis. Vaginal samples will be obtained during labor using the GeneXpert Vaginal/Endocervical Specimen Collection Kit. The swab will be inserted to ~4-5 cm, rotating against the vaginal wall 2-3 times, rolled on a glass microscope slide (for gram staining) and then placed into GeneXpert collection tubes containing transport reagent. Molecular testing will use GeneXpert qPCR technology, which is ideally suited for field use,[100] and provides excellent sensitivity and specificity for diagnosis of these infections.[101-103]

***C. trachomatis*, *N. gonorrhea*, *T. vaginalis*, and GBS.** Assays will be conducted based on the manufacturer’s recommendations. Assay samples will be collected into GeneXpert transport reagent and then pipetted into assay cassettes. Assays will be performed and read, and quality control tested following GeneXpert recommendations, as explained in package inserts.

BV. This syndrome entails overgrowth of normal commensal bacteria by a range of other organisms. Gold standard diagnosis requires characterization of flora by gram stain of vaginal secretions. Slides obtained as noted above will be air-dried and gram stained. The Nugent score, the gold standard for evaluation of BV,[104] will be used to score samples. In brief, trained microscopists (blinded to clinical information) will assess the presence of large gram-positive rods (principally lactobacilli; scored inversely 0-4), small Gram-variable rods (principally *Gardnerella*, scored 0 to 4), and curved Gram-variable rods (principally *Mobiluncus*, scored 0 to 2). Scores of 0 to 3 will indicate normal findings, 4 to 6 intermediate findings, and 7-10 BV.[105] Of note, new point-of-care technologies, measuring pH or sialidase activity are available, and if fully validated in the near future, a simpler assay might replace gram staining for diagnosis of BV.

6.10.9 Drug sensitivity of urinary tract infection (UTI) pathogens and *Neisseria gonorrhea*

Drug sensitivity patterns for common bacterial pathogens are poorly characterized in Uganda. SP has activity against several bacterial pathogens including *N. gonorrhea*, and is thought to have a similar spectrum of activity as the related antifolate drug trimethoprim-sulfamethoxazole (TMP-SMX). To test the hypothesis that SP increases the risk of detecting antifolate resistance among pathogenic bacteria isolated from women receiving SP for ITPp, we will culture urinary tract organisms and *N. gonorrhea* from study participants and determine sensitivities of these pathogens to commonly used antibiotics.

Urinary tract infections. If a study participant presents to the clinic with symptoms of a UTI including dysuria, hematuria, suprapubic tenderness, and/or flank pain and a urine dipstick is positive for nitrites or leukocyte esterase, a sterile urine specimen will be collected. The woman will be treated empirically for UTI after specimen collection per Uganda National Guidelines. The urine specimen will be plated on Cystine Lactose Electrolyte Deficient or Oxoid Brilliance UTI or another appropriate culture agar. Gram stain will be obtained from colonies with sufficient growth, and suspected pathogens will be identified using conventional biochemical tests. The susceptibility of the isolates to select antibiotics (e.g. antibiotics prioritized by the Uganda National AMR Surveillance Program) will be determined by gradient strip (E-test or similar) or disk diffusion devices, and minimum inhibitory concentration (MIC) values will be based on the European Committee on Antimicrobial Susceptibility (EUCAST) guidelines. Results from the urine cultures and antibiotic sensitivities will be used for research purposes only.

Neisseria gonorrhea. If *N. gonorrhea* is detected by GeneXpert from a vaginal swab sample at delivery, a sterile cotton swab collected concurrently with the GeneXpert sample at delivery will be cultured for *N. gonorrhea*. This swab will be cultured on G.C. selective agar in a CO2 rich atmosphere. Any growth will be provisionally identified using an oxidase test and gram stain, and confirmed with *N. gonorrhea* specific antisera. The susceptibility of the isolates to select antibiotics (e.g. antibiotics prioritized by the WHO Global Antimicrobial Resistance Surveillance System database) will be determined by gradient strip devices (E-test or similar) or disk diffusion devices and minimum inhibitory concentration (MIC) values will be based on the EUCAST guidelines. Results from the vaginal swab cultures and *N. gonorrhea* antibiotic sensitivities will be used for research purposes only.

6.10.10 Molecular markers of SP and DP resistance

We will survey *P. falciparum* isolates identified during monthly screening or when study subjects present with symptomatic malaria for polymorphisms associated with altered sensitivity to SP or DP (Table 3). If malaria prevalence and incidence remain high, as expected, a randomly chosen subset of samples will be studied to avoid study of many more samples than needed to identify meaningful associations. Assessment of SNPs will initially utilize a multiplex ligase detection reaction fluorescent microsphere assay, utilizing the Luminex MAGPIX platform. This system is well-validated and used routinely in our labs at UCSF and Kampala for this purpose.[126,127] In brief, DNA will be extracted from filter paper blood spots using Chelex, genes of interest will be amplified by nested PCR, and ligase detection reaction will be performed with allele-specific (5’ sequence complementing a sequence attached to a MagPlex-Tag bead) and common (corresponding to a particular SNP and modified by 5’ phosphorylation and 3’ biotinylation) primers. A multiplex reaction will assess SNPs of interest, using established protocols (Table 4).[126,128] Products will be hybridized to MagPlex-Tag beads, streptavidin-R-phycoerythrin will be added, and the abundance of alleles will be determined by assessing fluorescence. Multiple K13 SNPs are associated with artemisinin resistance in SE Asia; these will be assessed by Sanger sequencing of the amplified propeller domain, as previously described.[66] Amplification of *pfmdr1* (potentially important for piperaquine resistance, but rare to date in Africa)[128] and plasmepsin genes (associated with piperaquine resistance in SE Asia),[61,62] will be assessed by qPCR methods already standard in our laboratory.[60,128] Consideration of additional polymorphisms may be appropriate over the course of this project, based on advances in our understanding of drug resistance. For broader consideration of parasite polymorphisms we are exploring deep sequencing approaches, in particular molecular inversion probes[129] both to fully sequence genes of interest (Table 3) and to consider other potential resistance determinants, and these newer technologies will be incorporated as appropriate to best evaluate impacts of study regimens on parasite genomes.

Table 3. *P. falciparum* polymorphisms to be evaluated in clinical samples.

Gene	Polymorphism	Relevance
pfcrt	76T	Principal mediator of resistance to CQ, AQ; ? selected by PQ
pfmdr1	86Y, 184F, 1246Y	YYY haplotype associated with ↓S to CQ, AQ; 86Y selected by PQ
	S1034C, N1042D	Assoc with alterations in drug S, but not seen in Africa to date
	Gene amplification	Assoc with ↑S to CQ, AQ; relevance for PQ unknown
pfdhfr	51I, 59R, 108N, 164L	Mediate step-wise increases in R to SP; 164L mediates high-level R
pfdhps	437G, 540E, 581G	Mediate step-wise increases in R to SP; 581G mediates high-level R
K13	Multiple	Multiple SNPs associated with artemisinin resistance
PMP	Gene amplification	Mediates piperaquine R in SE Asia
Abbrev.: CQ, chloroquine; AQ, amodiaquine; PQ, piperaquine; S, sensitivity; R, resistance		

6.10.11 Pharmacokinetic assessments.

Plasma drug concentrations for piperaquine, sulfadoxine, and pyrimethamine will be obtained to assess for potential drug-drug interactions between DP and SP in the combined treatment study arm, assess relationships between drug exposure and QT interval prolongation, and quantify drug-exposure response relationships for IPTp and clinical outcomes. During phase 1, all women will undergo venous plasma sampling to quantify piperaquine, sulfadoxine and pyrimethamine concentrations immediately prior to the first daily dose of study drugs (28-day trough) and 2-6 hours after the third daily dose of study drugs (corresponding with the piperaquine peak concentration) at 20, 28 and 36 weeks gestational age. ECG measurements will be paired with each plasma collection. Due to DHA's short half-life, it is not responsible for the antimalarial protective efficacy of DP, and will only be measured after the 3rd daily dose. During phase 2, pre-treatment 28-day trough piperaquine, sulfadoxine, and pyrimethamine plasma concentrations may be quantified from a venous sample at weeks 20, 28, 36, and at delivery, from a capillary stick at weeks 24, 32, and 40 or when malaria is diagnosed. Blood samples will be centrifuged to isolate plasma, flash frozen on site, and plasma will be stored and shipped to UCSF in liquid nitrogen or on dry ice. After unblinding by an individual not involved in patient care or data analysis, drug levels will be quantified at UCSF using tandem mass spectrometry (AB Sciex API5000) coupled with Shimadzu Prominence 20AD^{XR} UFLC pumps using established methods. The lower limit of quantitation will be 0.5 ng/mL for piperaquine, 0.5 ng/mL for dihydroartemisinin, 2.7 mcg/mL for sulfadoxine, and 10 ng/mL for PYR.[74,130]

Nonlinear mixed effects modeling will be used to analyze the sparse PK data. Previously developed population PK models for each drug during pregnancy will serve as Bayesian priors, and collected sparse PK data will be used to update and adapt the PK models. Covariate analysis will be conducted, including quantification of any effect of co-administered drugs on population PK parameters including relative clearance (CL/F), volumes of distribution (V/F) and relative bioavailability. Derived PK parameters for all drugs including area under the curve from 0 to 28 days for piperaquine, sulfadoxine and pyrimethamine ($AUC_{0\text{-}28}$) or AUC from 0 to 24 hours for

dihydroartemisinin. C_{max} for pipearquine, and C_{trough} for sulfadoxine, pyrimethamine, and piperaquine will also be reported for each study drug regimen as recommended by U.S. Federal Drug Administration (FDA) guidance. Absence of a drug- drug interaction effect will be defined as the 90% confidence interval for the PK parameters of the drug when administered in combination falls within 80 to 125% of the parameter when the drug is administered alone. Relationships between drug exposure and outcomes of interest including malaria parasitemia and prevalence of placental malaria and toxicity (QTc prolongation or other adverse events) will also be assessed. If a clinically significant drug-drug interaction is detected after the initial analysis of the first 300 participants, simulations of the population PK model will be conducted to predict optimal dosing of the drug and a dose adjustment will be considered for Phase 2.

6.10.12 Immunology Studies

Protective immunity against the adverse effects of malaria in pregnancy increases with successive pregnancies. An important question is what impact interventions such as IPTp have on the acquisition of this gravidity-dependent immunity against malaria in pregnancy. We will evaluate the malaria-specific immune response in women between our 3 treatment arms. Venipuncture blood samples collected in select subjects already undergoing phlebotomy, and cord and placental blood collected as above will be made available for immunology studies. Due to limitations in cost of processing and storage capacity, samples will not be collected for all individuals and/or all time points. Approximately 15mls of blood will be collected into PAXgene RNA or standard blood collection tubes. Following collection, blood will be transported to our immunology laboratory located on the campus of Tororo District Hospital and separated into plasma and peripheral blood mononuclear cells (PBMCs), following standard protocols. Plasma will be stored at -80°C for future immunology studies, which may include measurement of levels of cytokines, antibodies, and other features related to the host immune response. PBMCs will be isolated by Ficoll centrifugation and counted, and cells not used immediately for assays will be cryopreserved in liquid nitrogen to maintain viability. Fresh PBMCs will be used to evaluate the malaria-specific cellular immune response by flow cytometry, ELISPOT, surface phenotyping and intracellular cytokine staining, and other assays of immune function in our research laboratory in Tororo on the campus of Tororo District Hospital. Additional immunologic assays will be performed in research laboratories at the Central Public Health Laboratories in Butabika, Uganda. For assays requiring advanced instrumentation not available in Uganda (e.g. RNA sequencing, epigenetic profiling, time of flight mass cytometry), cryopreserved samples will be shipped to the University of California San Francisco or Stanford University.

6.10.13. Host red blood cell polymorphisms.

Some inherited blood disorders are known to have a protective effect on human health, typically with protection in heterozygotes, but harmful effects in homozygotes. In Uganda, these polymorphisms are prevalent in some regions, with sickle cell trait (HbAS), alpha-thalassemia, and the African variant G6PD A- present in 28%, 53%, and 29% of residents of Tororo District (a district adjacent to Busia). [A1] Studies of associations between inherited blood disorders and malaria have largely focused on children. HbAS is known to protect against malaria, and particularly against severe malaria, among children.[A2-3] The African variant G6PD A- was protective among homozygous males in some studies,[A4] and alpha-thalassemia protected against severe malaria in some studies[A2] but not in others.[A3] It is unknown if RBC polymorphisms convey antimalarial benefits in pregnant women, but there are data showing RBC polymorphisms associated with adverse pregnancy outcomes. Studies have reported a higher prevalence of low birth weight among women with alpha- and beta-thalassemia trait compared to women without these traits.[A5-6] Studies have shown an increased risk of maternal mortality, pre-eclampsia, stillbirth, preterm birth and small-for-gestational age among pregnancies complicated with the sickle cell disease (HbSS) genotype compared to women without HbSS.[A7] Associations between sickle cell trait and adverse outcomes have yielded conflicting results.[A8-9] Malaria and adverse birth outcomes are common in Uganda, and understanding relationships between RBC polymorphisms and malaria and birth outcomes could allow risk stratifying pregnancies and targeting antimalarial or other pregnancy interventions.

We will characterize genetic polymorphisms such as alpha thalassemia, hemoglobin variants, and G6PD A-, and assess their associations with maternal and birth outcomes. Maternal outcomes of interest will include clinical malaria, placental malaria, asymptomatic parasitemia, anemia and pre-eclampsia. Birth outcomes will include composite birth outcomes, as well as, low birth weight, preterm birth and small-for-gestational age. Molecular methods will be used to characterize host hemoglobin genotypes (including HbAA, HbAC, HbCC, HbSC, HbAS, HbSS), the alpha thalassemia 3.7kb deletion (wild type, heterozygous and homozygotes), G6PD A- (wild type, heterozygous and homozygotes) and possibly other genes using DNA extracted from filter paper blood spots which are collected at routine visits.[A1] DNA will be extracted using Chelex, DNA for the genes of interest will be amplified by PCR with specific primers for each gene of interest, and polymorphisms will be characterized by dideoxy sequencing or analysis of gene fragments by electrophoresis after incubation with polymorphism-specific endonucleases. If an abnormal hemoglobin genotype is detected by the molecular assays and the participant remains under active follow-up during the study, the results will be confirmed by hemoglobin electrophoresis for sickle cell trait or sickle cell disease, clinically approved globin gene testing

for alpha thalassemia, and G6PD activity testing for G6PD A-. Results will be disclosed to the study participant by a study physician.

Added References

A1. Walakira A, Tukwasibwe S, Kiggundu M, et al. Marked variation in prevalence of malaria-protective human genetic polymorphisms across Uganda. Infect Genet Evol 2017:281–287.
A2. Mpimbaza A, Walakira A, Ndeezi G, et al. Associations between erythrocyte polymorphisms and risks of uncomplicated and severe malaria in Ugandan children: A case control study. PLoS One. 2018;13(9):1–15.
A3. Kakande E, Greenhouse B, Bajunirwe F, et al. Associations between red blood cell variants and malaria among children and adults from three areas of Uganda: A prospective cohort study. Malar J 2020;19(1):1–9.
A4. Guindo A, Fairhurst RM, Doumbo OK, Wellems TE, Diallo DA. X-linked G6PD deficiency protects hemizygous males but not heterozygous females against severe malaria. PLoS Med. **2007**; 4(3):516–522.
A5. Vafaei H, Karimi S, Akbarzadeh Jahromi M, Asadi N, Kasraeian M. The effect of mother's β-thalassemia minor on placental histology and neonatal outcomes. J Matern Neonatal Med **2020**; 0(0):1–8.
A6. Charoenboon C, Jatavan P, Traisrisilp K, Tongsong T. Pregnancy outcomes among women with beta-thalassemia trait. Arch Gynecol Obstet **2016**; 293(4):771–774.
A7. Oteng-Ntim E, Meeks D, Seed PT, et al. Adverse maternal and perinatal outcomes in pregnant women with sickle cell disease: Systematic review and meta-analysis. Blood. American Society of Hematology; 2015. p. 3316–3325.
A8. Bryant AS, Cheng YW, Lyell DJ, Laros RK, Caughey AB. Presence of the sickle cell trait and preterm delivery in African-American women. Obstet Gynecol **2007**; 109(4):870–874.
A9. Wellenstein WL, Sullivan S, Darbinian J, Ritterman Weintraub ML, Greenberg M. Adverse Pregnancy Outcomes in Women with Sickle Cell Trait. AJP Rep **2019**; 9(4):E389.

6.10.14. SARS-CoV-2 Testing.

Rationale for SARS-CoV-2 testing in the DP-SP cohort

SARS-CoV-2 has led to hundreds of millions of cases and millions of deaths worldwide,(1) yet the true extent of the SARS-CoV-2 epidemic in sub-Saharan Africa, including Uganda, remains unclear.(2) A recent study documented 12% SARS-CoV-2 seroprevalence of HCWs in Western Kenya, bordering Uganda(3), however this data was before the recent surge in SARS-CoV-2 infections in Uganda which peaked in June 2021 at 32 daily new confirmed cases per million people (likely an underestimate due to limited testing).(4) Pregnant women can be an important sentinel population for infectious diseases such as SARS-CoV-2, due to frequent

interactions with health systems. Further, pregnant women and their fetuses are particularly vulnerable to SARS-CoV-2, with infection during pregnancy being associated with placental perfusion abnormalities(5), preterm delivery(1), and low birth weight.(6) These are similar to effects seen from malaria infection during pregnancy, and it is as yet unknown if an interaction between malaria and SARS-CoV-2 co-infection during pregnancy would have a synergistic effect on adverse birth outcomes, or if, as has been postulated(7), one may mitigate the other, perhaps due to epitope cross-reactivity.(8) For these reasons, we are adding systematic SARS-CoV-2 testing to the study protocol.

Current SARS-CoV-2 testing algorithm in Busia, Uganda

Current procedures for SARS-CoV-2 testing are as follows, according to ministry of health guidelines: Any participant who is suspected to have contracted or been exposed to SARS-CoV-2 is referred to Masafu General Hospital for testing. If testing is available, a rapid diagnostic test is performed. If the rapid diagnostic test is negative, no further testing occurs. If the rapid diagnostic test is positive, then a confirmatory PCR test is performed. If positive, the participant would be managed according to local guidelines.(9) However, SARS-CoV-2 testing has been limited by availability of testing materials, and the true burden of COVID-19 in this population remains unknown; therefore we propose additional sample collection for systematic SARS-CoV-2 testing in this clinical trial cohort.

Additional sample collection

Participants presenting either at routine or unscheduled visit with fever, history of fever, symptoms suggestive of SARS-CoV-2 infection (fatigue/malaise, nausea, vomiting, diarrhea, cough, headache, rhinorrhea, abdominal pain, loss of taste/smell), or exposure to a person suspected/diagnosed with COVID-19 within the past 14 days, will be referred for rapid antigen testing according to local protocols described above. In addition, selected participants will also have a nasopharyngeal swab specimen collected, frozen and stored in RNA preservative. As resources allow, these banked specimens will be tested later for SARS-CoV-2 PCR and other pathogens potentially causing febrile illness using nucleic acid amplification and deep sequencing-based methods. One experimental method involves RNA extraction, cDNA library synthesis, and meta-genomic next-generation sequencing, using a specific informatic pipeline to identify pathogens after filtering out human sequence information. These newer tests may be performed at UCSF, or at reference centers depending on availability of technology and funding for additional pathogen detection work. These studies will be for research purposes only and will not affect patient care.

Additionally, serial testing of stored plasma samples will be used to assess for SARS-CoV-2 seroconversion during pregnancy. Plasma will be obtained from blood spots already being collected as part of the approved study protocol, therefore no additional specimens will be collected for SARS-CoV-2 serology testing. Seroconversion will be determined as follows: plasma at time of delivery will be tested for SARS-CoV-2 antibodies; if positive at the time of delivery, stored plasma samples will be tested to determine whether seroconversion occurred during pregnancy. Stored specimens from the time of study enrollment in the first trimester will first be tested. If negative, then additional stored specimens from each routine visit during pregnancy will be tested, working backwards from the time of delivery, until a negative specimen is found, in order to determine the gestational age at time of seroconversion.

Added References

1. Martinez-Perez O, Prats Rodriguez P, Muner Hernandez M, Encinas Pardilla MB, Perez Perez N, Vila Hernandez MR, et al. The association between SARS-CoV-2 infection and preterm delivery: a prospective study with a multivariable analysis. BMC Pregnancy Childbirth. 2021;21(1):273.
2. Nachega JB, Sam-Agudu NA, Budhram S, Taha TE, Vannevel V, Somapillay P, et al. Effect of SARS-CoV-2 Infection in Pregnancy on Maternal and Neonatal Outcomes in Africa: An AFREhealth Call for Evidence through Multicountry Research Collaboration. Am J Trop Med Hyg. 2020.
3. Etyang AO, Lucinde R, Karanja H, Kalu C, Mugo D, Nyagwange J, et al. Seroprevalence of Antibodies to SARS-CoV-2 among Health Care Workers in Kenya. Clin Infect Dis. 2021.
4. Mathieu E, Ritchie H, Ortiz-Ospina E, Roser M, Hasell J, Appel C, et al. A global database of COVID-19 vaccinations. Nature Human Behaviour. 2021;5(7):947-53.
5. Glynn SM, Yang YJ, Thomas C, Friedlander RL, Cagino KA, Matthews KC, et al. SARS-CoV-2 and Placental Pathology: Malperfusion Patterns Are Dependent on Timing of Infection During Pregnancy. Am J Surg Pathol. 2021.
6. Lassi ZS, Ana A, Das JK, Salam RA, Padhani ZA, Irfan O, et al. A systematic review and meta-analysis of data on pregnant women with confirmed COVID-19: Clinical presentation, and pregnancy and perinatal outcomes based on COVID-19 severity. J Glob Health. 2021;11:05018.
7. Kalungi A, Kinyanda E, Akena DH, Kaleebu P, Bisangwa IM. Less Severe Cases of COVID-19 in Sub-Saharan Africa: Could Co-infection or a Recent History of Plasmodium falciparum Infection Be Protective? Front Immunol. 2021;12:565625.
8. Iesa MAM, Osman MEM, Hassan MA, Dirar AIA, Abuzeid N, Mancuso JJ, et al. SARS-CoV-2 and Plasmodium falciparum common immunodominant regions may explain low COVID-19 incidence in the malaria-endemic belt. New Microbes New Infect. 2020;38:100817.
9. Revised National Guidelines for Management fo COVID-19, 3rd Edition. In: Health TRoUMo, editor. Kampala, Uganda2021.

6.10.15 Fetal Ultrasounds during and after febrile illness

<u>Rationale for additional ultrasounds during and after febrile illness in the DP-SP study cohort</u>

Malaria in pregnancy is responsible approximately 70% of fetal growth restriction (FGR) cases and 36% of preterm deliveries in high transmission areas (1). Placental malaria is characterized

by the sequestration of infected RBCs in the intervillous spaces of the placenta and subsequent inflammatory response with infiltration of maternal monocytes/macrophages (2). However, the effect of malaria parasitemia on the uteroplacental and fetoplacental blood flow and its association with pregnancy outcomes remains unknown. Assessment of the vascular dynamics at multiple fetal vessels such as umbilical artery (UA) and middle cerebral artery (MCA) in addition to the estimated fetal weight via ultrasounds can provide a more comprehensive picture of fetal compensatory mechanisms to ongoing intrauterine insult during malaria in pregnancy (3).

Doppler ultrasound allows for non-invasive real-time assessment of maternal-fetal vascular dynamics (3,4). Pregnancies complicated by FGR with abnormal UA Dopplers have decreased villous development and reduced vascularization, suggesting that aberrant fetoplacental vascular development is a mechanism for FGR (5,6). Alterations in UA or MCA Dopplers indicate increased placental insufficiency and the resultant fetal physiologic adaptation, respectively (3,4). Abnormalities in fetal Dopplers are associated with stillbirth and increased risk of adverse long term neurodevelopmental and cardiovascular outcomes (7,8,9). However, the onset and progression of these abnormalities after febrile illness remains unknown.

Longitudinal monitoring of fetal blood flow by Doppler ultrasound is an integral part of optimal FGR management and reduces perinatal morbidity in high resource settings (4). The effect of infected RBC sequestration during acute malaria presenting with febrile illness in the intervillous space on the uteroplacental and fetoplacental blood flow is unknown. Dopplers studies are safe in pregnancy, relatively easy to perform, and can be performed using ultrasound machines used for growth assessment. Utilization of Doppler studies can aid in the diagnosis and management of FGR fetus at high risk of perinatal morbidity and mortality and may be a useful intervention to reduce perinatal mortality in LMIC. Fetal surveillance with Doppler studies in malaria-endemic areas is seldom accessible, despite the high incidence of FGR and malaria. For these reasons, we propose to study fetal vascular changes at the maternal and fetal interface in pregnancies affected by acute malaria presenting with febrile illness via ultrasounds in the DPSP cohort.

Additional data collection:

During febrile illness:
We will perform additional fetal ultrasound assessments in study participants presenting with acute fever at the time of visit or within past 24 hours of their visit. Participants are instructed to present to the study clinic with fever, at which time they are evaluated for malaria with peripheral blood smear. We will perform a fetal ultrasound at the time of presentation for fever. We will then follow the patient approximately every 4 weeks at the time of previously scheduled routine assessments (see section 6.5) for additional ultrasound studies until delivery to determine if there are acute or chronic fetoplacental adaptations to malaria. For participants with abnormal ultrasound findings, weekly follow-up will be continued until > 2 subsequent studies are normal. This data will not require and additional clinical visits and be used for research purposes only. Pregnancies will be otherwise managed per local guidelines.

Added References

1. Katz J, Wu LA, Mullany LC, Coles CL, Lee ACC, Kozuki N, et al. Prevalence of Small-for-Gestational-Age and Its Mortality Risk Varies by Choice of Birth-Weight-for-Gestation Reference Population. PLoS One [Internet]. 2014 Mar 18;9(3):e92074. Available from: https://doi.org/10.1371/journal.pone.0092074
2. Zakama AK, Ozarslan N, Gaw SL. Placental Malaria. Curr Trop Med reports. 2020 Sep;1–10.
3. Abuhamad A, Martins JG, Biggio JR. Diagnosis and management of fetal growth restriction: the SMFM guideline and comparison with the ISUOG guideline. Vol. 57, Ultrasound in Obstetrics and Gynecology. 2021.
4. Leavitt K, Odibo L, Nwabuobi C, Tuuli MG, Odibo A. The value of introducing cerebroplacental ratio (CPR) versus umbilical artery (UA) Doppler alone for the prediction of neonatal small for gestational age (SGA) and short-term adverse outcomes. J Matern neonatal Med Off J Eur Assoc Perinat Med Fed Asia Ocean Perinat Soc Int Soc Perinat Obstet. 2021 May;34(10):1565–9.
5. Chen C-P, Bajoria R, Aplin JD. Decreased vascularization and cell proliferation in placentas of intrauterine growth–restricted fetuses with abnormal umbilical artery flow velocity waveforms. Am J Obstet Gynecol. 2002;187(3):764–9.
6. Jackson MR, Walsh AJ, Morrow RJ, Mullen JB, Lye SJ, Ritchie JW. Reduced placental villous tree elaboration in small-for-gestational-age pregnancies: relationship with umbilical artery Doppler waveforms. Am J Obstet Gynecol. 1995;172(2 Pt 1):518–25.
7. Leitner Y, Fattal-Valevski A, Geva R, Eshel R, Toledano-Alhadef H, Rotstein M, et al. Neurodevelopmental outcome of children with intrauterine growth retardation: a longitudinal, 10-year prospective study. J Child Neurol. 2007;22(5):580–7.
8. Crispi F, Miranda J, Gratacós E. Long-term cardiovascular consequences of fetal growth restriction: biology, clinical implications, and opportunities for prevention of adult disease. Am J Obstet Gynecol. 2018;218(2):S869–79.
9. Ayoola OO, Omotade OO, Gemmell I, Clayton PE, Cruickshank JK. The impact of malaria in pregnancy on changes in blood pressure in children during their first year of life. Hypertension. 2014;63(1):167–72.

6.10.16 Sample storage for future use

Certain biologic specimens (placenta, filter paper, whole blood, plasma, stool, vaginal fluid, nasopharyngeal swabs) will be stored in our laboratories for future laboratory studies, pending participant consent for future use of biological specimens obtained during the course of the study. Samples will generally be stored in our laboratory in Tororo, Uganda. Certain specimens requiring long term freezer storage may be transferred for long-term storage to our laboratory in Kampala, Uganda (IDRC Molecular laboratory) or a specialized specimen bank with our collaborators at UCSF (Department of Medicine, 1001 Potrero Avenue, Building 3, Room 511).

With consent for future use, sample retention will generally be open ended. Samples that do not have consent for future use, as detailed above, will be destroyed upon completion of clinical studies. Samples will be destroyed following SOPs which comply with local regulations and guidance.

6.11. Co-enrollment Guidelines

The pregnant women from this study may be co-enrolled in observational studies. They may not be co-enrolled in protocols that utilize concomitant study medications. Co-enrollment in other studies will be determined on a case-by-case basis by the protocol team.

6.12. Management of Adverse Events Potentially Related to Study Drugs

The following section outlines management of adverse events potentially related to study drugs (SP or DP) among pregnant women. Given the double-blinded nature of the intervention, study clinicians and study participants will not be aware of what study drugs are being administered. Therefore, all women will be considered potentially exposed to either study drug during the period between when study drugs are first given through completion of follow-up (4 weeks post-partum).

6.12.1. Grade 1 or 2 Adverse Events

Participants who develop grade 1 or 2 adverse events may continue study drugs. The study clinicians will manage the grade 1 or 2 events according to standard practice.

6.12.2. Grade 3 or 4 Adverse Events

<u>Management will be as follows:</u>

- Repeat observation or lab test within 72 hours of observation or of receiving lab results report.
- Work-up to exclude other causes.
- For grade 3 or non-life threatening grade 4 adverse events, subjects may continue taking study drugs pending clinic visit or repeat laboratory tests. The study clinician has the option of immediately stopping the study drugs if the subject cannot be examined in clinic, if a repeat laboratory test cannot be performed within 72 hours, or if the clinician determines that the continuation of study drugs is unsafe while awaiting the clinic exam or test results.
- For grade 4 life-threatening adverse events, study clinicians should hold study drugs pending laboratory confirmation.

- For all grade 3 or 4 adverse events confirmed by repeat clinical exam or laboratory test results, study drugs will be held, and laboratory tests will be repeated every 1-2 weeks, until the adverse event resolves to ≤ grade 2 unless there is strong evidence that the adverse event is not related to either study drug.
- If the adverse event persists at grade 3 or 4 for more than 28 days or recurs on re-challenge, and the adverse event is thought to be possibly related to one of the study drugs, the study drugs will be permanently discontinued.

In the event that study drugs are permanently discontinued, study participants will remain in the study, following our intention-to-treat analysis approach. In the event that study drugs are permanently discontinued, study clinicians may become un-blinded to the study participant assigned treatment regimen if this knowledge may assist in the management of the grade 3 or 4 adverse event(s) that lead to the permanent discontinuation of the study drugs.

7. MONITORING OF ADVERSE EVENTS AND MANAGEMENT

7.1 Monitoring and Reporting of Adverse Events

7.1.1 Definitions

An adverse event is defined as "any untoward medical occurrence in a patient or clinical investigation subject administered a pharmaceutical product that does not necessarily have a causal relationship with this treatment" (ICH Guidelines E2A). An adverse event can further be broadly defined as any untoward deviation from baseline health, which includes:

- Worsening of conditions present at the onset of the study
- Deterioration due to the primary disease
- Concurrent illness
- Events related or possibly related to concomitant medications

(International Centers for Tropical Disease Research Network Investigator Manual, Monitoring and Reporting Adverse Events, 2003).

7.1.2 Identification of Adverse Events

At each scheduled and unscheduled visit to the clinic, study clinicians will assess for adverse events in all pregnant women who have started study drugs, according to a standardized case

record form. A severity grading scale, based on toxicity grading scales developed by DAIDS and DMID Toxicity Tables, will be used to grade severity of all symptoms, physical exam findings, and laboratory results (Appendix H). All pregnant women, regardless of treatment arm, will be assessed using the same standardized case record form. Adverse event monitoring will occur during the period when study drugs are given and up to 4 weeks post-partum.

Data will be captured on the incidence of all adverse events, regardless of severity. For each adverse event identified and graded as severe or life threatening and felt to be possibly, probably or definitely related to study drugs, an adverse event report form will be completed. In addition, an adverse event form will be completed for all serious adverse events and unexpected events, regardless of severity. An adverse event report form will not be completed for events classified as mild or moderate (unless they are serious or unexpected), as mild and moderate symptoms are common and difficult to distinguish from signs and symptoms due to malaria and other common illnesses. The following information will be recorded for all adverse experiences that are reported:

1) Description of event
2) Date of event onset
3) Date event reported
4) Maximum severity of the event
5) Maximum suspected relationship of the event to study drugs (either SP or DP)
6) Whether the event is a serious adverse event
7) Initials of the person reporting the event
8) Outcome
9) Date event resolved

7.1.3 Reporting of Adverse Events

Guidelines for reporting of adverse events provided by DMID, UCSF CHR, and the FDA in the U.S. and the MU-SBSREC or MU-SOMREC, and NDA in Uganda will be followed as summarized in Table 4 below.

Table 4. Guidelines for reporting adverse events

Institution	Type of Adverse Events	When to Report
**UCSF-CHR**	External (off-site) Adverse Events are:<br>• AEs that occur in study participants who are not enrolled at a UCSF or affiliated study site. These AEs occur at sites that are under the oversight of another IRB.	• Not applicable
**MU-SBSREC or MU-SOMREC**	• All Serious and Unexpected events irrespective of relationship	• Fatal or life-threatening events within 3 working days of awareness<br>• All other SAEs within 7 calendar days

**NDA**	• All Serious and Unexpected events irrespective of relationship	• Within 7 calendar days of awareness
**FDA**	• Definitely, Probably or Possibly related **AND BOTH** Serious* AND Unexpected±	• For fatal or life-threatening events, by telephone or fax within 7 calendar days of first awareness<br>• All other reportable events within 15 calendar days of first awareness
**NIH/DMID**	• All Serious and Unexpected events irrespective of relationship	• All SAEs must be submitted within 24 hours of site awareness on an SAE form to the DMID pharmacovigilance contractor, at the following address:<br>DMID Pharmacovigilance Group<br>Clinical Research Operations and Management Support (CROMS)<br>6500 Rock Spring Dr. Suite 650<br>Bethesda, MD 20817, USA<br><br>SAE Hot Line: 1-800-537-9979 (US) or 1-301-897-1709 (outside US)<br>SAE FAX Phone Number: 1-800-275-7619 (US) or 1-301-897-1710 (outside US)<br>SAE Email Address: PVG@dmidcroms.com

SAE is any AE that results in any of the following outcomes:

- Death,
- Life-threatening adverse experience,
- Inpatient hospitalization or prolongation of existing hospitalization,
- Persistent or significant disability/incapacity,
- Congenital anomaly/birth defect, or cancer, or
- Any other experience that suggests a significant hazard, contraindication, side effect or precaution that may require medical or surgical intervention to prevent one of the outcomes listed above,
- Event that changes the risk/benefit ratio of the study.

Unexpected Adverse Event An adverse event is defined as being unexpected if the event exceeds the nature, severity, or frequency described in the protocol, consent form and investigator brochure (when applicable). An unexpected AE also includes any AE that meets any of the following criteria:

- Results in subject withdrawal from study participation,
- Due to an overdose of study medication, or
- Due to a deviation from the study protocol

8. STATISTICAL CONSIDERATIONS

All analyses will be conducted using a modified intention-to-treat approach including all women randomized to therapy who have an evaluable outcome of interest.

8.1 Hypothesis 1

We will test the hypothesis that pregnant women who receive IPTp every 4 weeks with a combination of SP+DP will have a lower risk of a composite adverse birth outcome compared to those who receive either SP or DP alone.

8.1.1. Primary Outcome

The primary outcome will be the risk of having a composite adverse birth outcome defined as the occurrence of any of the following:

Outcomes	Definition
Spontaneous abortion	Fetal loss at < 28 weeks gestational age
Stillbirth	Infant born deceased at ≥ 28 weeks gestational age
LBW	Live birth with birth weight < 2500 gm
Preterm birth	Live birth at < 37 weeks gestational age
SGA	Live birth with weight-for-gestational age < 10th percentile of reference population[131]
Neonatal death	Live birth with neonatal death within the first 28 days of life

8.1.2 Analysis

Primary analysis. Using a modified intent-to-treat approach, we will compare the prevalence of our primary outcome between the study arms using unadjusted log-binomial models. We will explore for any differences of potential confounders between the treatment arms and if necessary adjust our analysis using multivariate log-binomial models.

Secondary analyses. We will compare the prevalence of our individual adverse birth outcomes using the same approach as our primary analysis.

8.2 Hypothesis 2

We will test the hypothesis that pregnant women who receive IPTp with SP+DP will have non-inferior risks of adverse events and measures of tolerability compared to those who receive either SP or DP alone.

8.2.1. Primary Outcome

The primary outcome will be the incidence of any grade 3-4 AE or SAE per time at risk. Time at risk will begin the day study drugs are first given and will end when study participants reach 4 week postpartum or early study termination.

8.2.2. Secondary Outcomes

Secondary outcomes will include the incidence of individual grade AEs, the incidence of AEs related to study drugs, vomiting following administration of study drugs, and measure of non-adherence with study drugs.

8.2.3. Analysis

Primary analysis. Using a modified intent-to-treat approach, we will compare the incidence of our primary outcome between the study arms using a negative binomial regression model. Conclusions will be based on tests of non-inferiority (see section 8.4).

Secondary analyses. We will compare the incidence of our other measures of safety using the same approach as our primary analysis. Comparisons of repeated measures of tolerability will be made using generalized estimating equations with a log-binomial regression model and robust standard errors.

8.3 Hypothesis 3

For objective 3a we will test the hypotheses that pregnant women who receive IPTp regimens containing DP will have a lower risk of measures of malaria during pregnancy and at delivery but a higher risk of infection with malaria parasites containing mutations associated with DP resistance compared to those who receive SP alone. For objective 3b we will test the hypotheses that pregnant women who receive IPTp regimens containing SP will have a lower risk of RTIs, greater changes in their vaginal/intestinal microbiota, and a higher risk of infection with malaria parasites containing mutations associated with SP resistance compared to those who receive DP alone.

8.3.1. Outcomes for objective 3a

Outcomes	Definition
Placental malaria	Detection of malaria parasites or pigment by histopathology

Incidence of malaria during pregnancy	New episodes of fever plus positive blood smear per person time
Prevalence of parasitemia during pregnancy	Proportion of routine samples with asexual parasites detected by microscopy or qPCR
Prevalence of anemia during pregnancy	Proportion of routine hemoglobin measurements < 11 g/dL
Prevalence of markers of DP resistance	Proportion of parasite positive samples with molecular markers of DP resistance

8.3.2. Outcomes for objective 3b

Outcomes	Definition
Prevalence of RTIs at delivery	Proportion of vaginal samples collected as the time of delivery positive for RTIs
Changes in the vaginal/intestinal microbiota	Measures of relative and absolute abundance of microorganisms
Prevalence of markers of SP resistance	Proportion of parasite positive samples with molecular markers of SP resistance

8.3.3. Analyses

Prevalence outcomes measured at a single time point will be compared using a log-binomial model. Prevalence outcomes involving repeated measures will be compared using generalized estimating equations with a log-binomial regression model and robust standard errors. Comparisons of malaria incidence will be made using a negative binomial regression model.

8.4 Sample size and power

Our sample size calculation is based on testing the primary hypothesis for specific aim 1. Based on our previous study from the same site, the risk of the composite adverse birth outcome as defined for aim 1 was 22.6% in the SP and 25.1% in the DP arm among women meeting the eligibility criteria for this proposal. Given these estimates, we would need to enroll 2757 women (assuming 15% loss to follow-up) to have 80% power (2-sided alpha = 0.05) to detect a 25% or greater reduction in the risk of our composite adverse birth outcome in the SP+DP arm compared to either of the other arms. For the primary outcome of specific aim 2, we assume that the incidence of any grade 3-4 AE or SAE will be 0.37 per person year (SD=0.57) in the SP

arm and 0.18 per person year (SD=0.35) in the DP arm, based on our previous study. Given these estimates we will have 80% power (1-sided alpha = 0.025) for our non-inferiority margin to be set at 0.07 when comparing the SP+DP arm to the SP arm and at 0.03 when comparing the SP+DP arm to the DP arm. For the primary outcome of specific aim 3a, we assume that the risk of placental malaria will be 61% in the SP arm based on our previous study. Given our sample size, we will have 80% power (2-sided alpha = 0.05) to detect a 12% or greater relative reduction in the prevalence of malaria parasites at delivery in the SP+DP arm or the DP arm compared to the SP arm.

8.5 Data and Safety Monitoring Plan

The proposed study will conform to rigorous standard monitoring procedures, standardized reporting of adverse events (Adverse Event Report Forms are completed by study coordinators and sent immediately to the investigators), and regular review of the study by a DSMB. The PIs have primary responsibility for the overall conduct of the study, including the safety of human subjects. The PIs will ensure appropriate (1) conduct of the informed consent process (e.g. that informed consent is obtained before proceeding with study procedures); (2) enrollment of study subjects; (3) collection and analysis of data; (4) implementation of study procedures to ensure consistent monitoring of subjects for possible adverse events; (5) review of adverse events and reporting to the DSMB and the IRBs; and (6) maintenance of the privacy and confidentiality of study subjects. The PIs maintain ultimate responsibility for the project and for the safety of study participants. The PIs will be in contact with the research team on a regular basis to review the progress of the study and address any human subject issues that occur. These discussions may involve adverse event prevention measures, recruiting of appropriate study subjects, research staff training on protection of human subjects, as well as occurrence of adverse events, unexpected incidents, or protocol problems.

8.6 Data and Safety Monitoring Board

A DSMB will be established by the study team in cooperation with the sponsor to assess at intervals the progress of a clinical trial, safety data, and critical efficacy variables and recommend to the sponsor whether to continue, modify or terminate a trial. The DSMB will have written operating procedures and maintain records of all its meetings, including interim results; these will be available for review when the trial is complete. The DSMB will be a separate entity from the US and host-country IRBs. The independence of the DSMB is intended to control the sharing of important comparative information and to protect the integrity of the clinical trial from adverse impact resulting from access to trial information. DSMB members will

not participate in the study as investigators and will not have conflicts of interest regarding the study or the investigational product. The composition of the DSMB will include at minimum:

- DSMB Chair, having experience and expertise in clinical trials
- Scientist with expertise in malaria and malaria in pregnancy
- Biostatistician with expertise in clinical trials.

A member of the sponsor, will be invited to attend and thus have access to un-blinded information, and control of dissemination of interim trial results within the sponsor organization. The DSMB will meet at least annually to review progress of the clinical trial and safety data.

The DSMB will review the study for progress and safety. The PIs will provide information that will allow the DSMB to review and assess the following:

- The research protocol, informed consent documents and plans for data safety and monitoring;
- Periodic assessments of data quality and timeliness, participant recruitment, accrual and retention, participant risk versus benefit, and other factors that can affect study outcome;
- Factors external to the study when relevant information, such as scientific or therapeutic developments, may have an impact on the safety of the participants or the ethics of the trial;
- Study performance to make recommendations and assist in the resolution of problems;
- The safety of the study participants;
- The safety and scientific progress of the trial;
- The continuation, termination or other modifications of the trial based on the observed beneficial or adverse effects of the treatment under study;
- The confidentiality of the data and the results of monitoring; and
- Any problems with study conduct, enrollment, sample size and/or data collection.

The first meeting of the DSMB will take place prior to the initiation of the study to discuss the protocol and the Data Safety Monitoring Plan. Meetings of the DSMB shall be held according to the plan outlined above. Meetings shall be closed to the public because discussions may address confidential patient data. Meetings may be convened as conference calls as well as in person. An emergency meeting of the Board may be called at any time should questions of patient safety arise. The DSMB may request the presence of study investigators at such meetings.

The study PIs will distribute study information to the DSMB at least 10 days prior to a scheduled meeting. The DSMB may request additions and other modifications to this information on a one-time or continuing basis. This information will consist of two parts: (1) information on study progress such as accrual, baseline characteristics, and other general information on study status and (2) any confidential data on study outcomes, including safety data. A formal report from the DSMB should be supplied to the PI within 6 weeks of each meeting. Each report should conclude with a recommendation to continue or to terminate the study. This recommendation should be made by formal majority vote. A recommendation to terminate the study should be transmitted to the PI, IRBs and NIH as rapidly as possible, by immediate telephone and fax if sufficiently urgent. In the event of a split vote in favor of continuation, a minority report should be contained within the regular DSMB report.

8.7 Interim safety analysis

Over the course of the trial, interim safety analyses will be performed when the first 300 women enrolled have been followed through delivery (phase 1) and then every 6 months during phase 2 of the study. A standardized test statistic will be calculated for the incident rate ratio of significant adverse events (grade 3/4 & SAEs). If this statistic exceeds the nominal critical value calculated using the error spending function (Table 5), then a statistically significant result will have been achieved at the time of the analysis. In that event, the sponsor will be notified and a report submitted for review by the DSMB. For the interim safety analyses, the study team will present information on recruitment and the results of interim safety analyses to the DSMB, which will review the data and recommend a course of action.

Table 5. Schedule of interim safety analysis and boundaries to monitor study outcome

Number of Evaluable Subjects Accrued	Test Statistic		Alpha	Cumulative Alpha
	Lower Bound	Upper Bound		
N=300 (phase 1)	-5.37	5.37	0.0000001	0.0000001
N=791 (phase 2)	-3.71	3.71	0.0002069	0.000207
N=1282 (phase 2)	-2.97	2.97	0.002844	0.003051
N=1773 (phase 2)	-2.54	2.54	0.009046	0.0121
N=2264 (phase 2)	-2.25	2.25	0.01605	0.02815
N=2757 or 100% of accrual	-2.04	2.04	0.02185	0.05

This analysis assumes α=0.05 (two-sided test), O'Brien-Fleming boundaries (DeMets error-spending function) and 2757 trial participants. We will utilize Programs for Computing Group Sequential Boundaries Using the Lan-DeMets Method.

8.8 Stopping rules

The DSMB will determine whether to stop the study for early evidence of intervention safety problems after a thorough review of interim data. Interim reports will provide cumulative enrollment figures and cumulative adverse birth outcomes, serious adverse events (classified according to grade), sorted by study arm. Brief clinical descriptions of key events will also be provided. The PIs will be responsible for immediately reporting to the funding agency any temporary or permanent suspension of the project and the reason for the suspension.

9 DATA COLLECTION AND MONITORING

9.1 Record Keeping

All clinical data will be recorded onto standardized CRFs by study clinicians. Blood smear results will be recorded in a laboratory record book by the study laboratory technologists and then transferred to the case record forms by study coordinators, who will review the case record forms frequently for completeness and accuracy. Other laboratory data (CBC, ALT measurements) will be entered into the CRFs and hard copies of the original results will be stored in a file. Data will be entered directly from CRFs into a computerized database or transferred from the CRFs onto standardized data extraction forms and then into a computerized database. All computerized data will be double entered to verify accuracy of entry. Electronic data including all study databases and supporting electronic documentation will be archived to large-scale digital tape on a daily basis. On a monthly basis, a complete backup tape will be transported off-site to the Kampala Data Management Center for rotating secure storage.

9.2 Data Quality Assurance and Monitoring

In order to ensure data quality, the study Data Manager will perform a quarterly data quality audit. For this audit a 1% random sample of study forms entered into the data management system from the previous 2 weeks will be selected and compared for accuracy with the original case-report forms and source documents. In addition, the study Data Manager will perform monthly reviews of the 100% double data entry data verification logs and the data management system audit trail log to identify potential data quality issues**.** The data will be owned by the IDRC-UCSF Research Collaboration.

10. HUMAN SUBJECTS

10.1 Subject Selection Criteria

Study subjects will be HIV-uninfected pregnant women who meet our selection criteria and provide informed consent. We plan to recruit only female Ugandan residents age 16 and above.

10.2 Risks and Discomforts

10.2.1 Privacy

Care will be taken to protect the privacy of subjects, as described in this protocol. However, there is a risk that others may inadvertently see patients' medical information, and thus their privacy may be compromised.

10.2.2 Finger Pricks and Venipuncture

Risks of these procedures include pain, transient bleeding and soft-tissue infection.

10.2.3 Risks of Study Medications

10.2.3.1 Risk of Sulfadoxine-Pyrimethamine

Although technically a combination regimen, SP is generally considered a single antimalarial agent, as its success depends on the synergistic action of its two component inhibitors of folate synthesis. SP is currently the standard of care for IPTp throughout sub-Saharan Africa. Adverse reactions listed on the SP package insert are blood dyscrasias (agranulocytosis, aplastic anemia, thrombocytopenia), allergic reactions (erythema multiforme and other dermatological conditions), gastrointestinal reactions (glossitis, stomatitis, nausea, emesis, abdominal pain, hepatitis, diarrhea), central nervous system reactions (headache, peripheral neuritis, convulsions, ataxia, hallucinations), respiratory reactions (pulmonary infiltrates), and miscellaneous reactions (fever, chills, nephrosis); based on widespread experience with the drug, all of these reactions appear to be uncommon or rare with short-term therapeutic use. The best-documented severe adverse effects with SP are cutaneous reactions, primarily noted when SP was used for long-term chemoprophylaxis in non-African populations. Reported rates of serious reactions to SP in the UK, with long-term use for chemoprophylaxis, were 1:2100, with 1:4900 serious dermatological reactions and 1:11,100 deaths.[132] Estimated rates of toxicity in the US were 1:5000-8000 severe cutaneous reactions and 1:11,000-25,000 deaths.[133] Clinical

experience suggests that risks of severe toxicity are much lower with malaria treatment regimens in Africa.

The WHO currently recommends IPTp with SP in areas with moderate-to-high malaria transmission.[134] In a recent systematic review and meta-analysis of 7 trials from sub-Saharan Africa, IPTp with 3 or more doses of SP was associated with a higher birth weight and lower risk of low birth weight compared to 2 doses of SP. In addition there were no differences in the rates of serious adverse events between the two groups.

10.5.3.2. Risk of Dihydroartemisinin-piperaquine

Minyt and colleagues conducted a systematic review of DP efficacy and safety for treatment of malaria using data from 14 clinical trials involving adults and children.[135] There were 2636 study participants treated with DP in 13 trials in which safety data were reported. Overall, DP was associated with fewer adverse events compared to comparator medications. The most common adverse events were dizziness, nausea and vomiting, though generally the medication was well-tolerated by both adults and children (Table 6). Of note, the only serious adverse events in these 14 studies included 5 deaths (2 adults, 3 children) that were thought unrelated to DP.

Table 6. Summary of adverse events following treatment with dihydroartemisinin-piperaquine

Study/site	Nausea	Vomiting	Anorexia	Dizziness	Headache	Diarrhoea	Abdominal pain	Sleep disturbance	Neuropsychiatric adverse events	Cardiovascular dysfunction	Haematological dysfunction	Hepatological dysfunction	Dermatological adverse events	Total no. of evaluated patients
Denis et al. (2002) Cambodia	5 (4.7)	NR	4 (3.8)	5 (4.7)	0 (0)	5 (4.7)	5 (4.7)	NR	NR	NR	NR	NR	1 (0.9)	106
Wilairatana et al. (2002) Thailand	8 (3.4)	0 (0)	NR	11 (4.7)	9 (3.8)	NR	NR	0 (0)	0 (0)	NR	NR	NR	NR	234
Ying et al. (2003) China	3 (5)	1 (1.6)	NR	NR	NR	NR	NR	NR	NR	NR	NR	NR	NR	60
Hien et al. (2004) Vietnam	8 (2)	NR	NR	NR	NR	NR	NR	NR	NR	NR	NR	NR	NR	399
Karunajeewa et al. (2004) Cambodia	NR	NR	NR	NR	NR	NR	NR	NR	NR	0 (0)	0 (0)	0 (0)	NR	62
Hung et al. (2004) Cambodia	3 (4)	NR	1 (1)	12 (14)	30 (36)	NR	9 (11)	1 (1)	NR	NR	NR	NR	NR	80
Giao et al. (2004) Vietnam	NR	NR	NR	NR	1 (1.2)	NR	NR	NR	NR	NR	NR	NR	1 (1.2)	82
Ashley et al. (2004) Bangkok, Thailand	11 (9.3)	NR	NR	9 (7.6)	12 (10.2)	NR	NR	NR	NR	NR	NR	NR	NR	118
Ashley et al. (2004) Mae Sot, Thailand	30 (8.5)	18 (1.7)	NR	51 (14.5)	NR	20 (5.7)	35 (9.9)	20 (7.4)	NR	0 (0)	0 (0)	0 (0)	1 (0.3)	353
Ashley et al. (2005) Thailand	37 (11.1)	23 (6.9)	NR	37 (11.1)	NR	33 (9.9)	28 (8.4)	42 (12.6)	NR	NR	NR	NR	3 (0.9)	333
Tangpukdee et al. (2005) Thailand	5 (4.2)	0 (0)	0 (0)	4 (3.3)	4 (3.3)	0 (0)	0 (0)	0 (0)	0 (0)	0 (0)	0 (0)	0 (0)	0 (0)	120
Smithuis et al. (2006) Myanmar	39 (11.9)	6 (1.8)	10 (8.3)	104 (31.8)	0 (0)	11 (9.2)	3 (0.9)	0 (0)	NR	NR	0 (0)	NR	0 (0)	327
Mayxay et al. (2006) Lao PDR	6 (5.5)	3 (3)	10 (9)	12 (11)	11 (10)	8 (7)	12 (11)	17 (15.5)	0 (0)	NR	NR	NR	0 (0)	110
Karema et al. (2006) Rwanda	2 (0.8)	5 (2)	3 (1.2)	1 (0.4)	2 (0.8)	8 (3.2)	6 (2.4)	0 (0)	0 (0)	NR	NR	NR	0 (0)	252

NR: not reported.
Data are given as *n* (%).

Lwin et al conducted a randomized controlled trial of monthly vs. bimonthly DP IPT among 961 adults at high risk of malaria at the Northwest border of Thailand.[136] Overall, 69% of the participants included in the final analysis reported at least one adverse event. There was no difference in the proportion of those reporting at least one adverse event among participants in

the monthly vs. bimonthly vs. placebo arms. There was an increased risk of joint pain among participants randomized to the placebo arm, but otherwise there were no differences noted in adverse events by study arm. There was only one serious adverse event not related to the use of DP (Table 7). In a recent systematic review and meta-analysis of repeated treatment, monthly DP was found to be effective, well tolerated, and safe.[137]

Table 7. Frequency, incidence, and risk of the 20 most frequently reported adverse events

	No. of participants in indicated treatment group who reported adverse event (%)[a]			Incidence rate for indicated group[b]				
Adverse event	DPm	DPalt	Placebo	DPm	DPalt	Placebo	IRR (95% CI) for DPm and DPalt	IRR (95% CI) for DPm and placebo
Dizziness	127 (32.8)	119 (31.2)	49 (25.4)	0.66	0.62	0.69	0.93 (0.72–1.21)	1.05 (0.74–1.47)
Headache	115 (29.7)	108 (28.4)	49 (25.4)	0.60	0.56	0.69	0.93 (0.71–1.23)	1.16 (0.81–1.63)
Soft stool	99 (25.6)	82 (21.5)	29 (15.0)	0.52	0.42	0.41	0.82 (0.61–1.12)	0.80 (0.51–1.22)
Abdominal pain	67 (17.3)	57 (15.0)	28 (14.5)	0.35	0.30	0.40	0.85 (0.58–1.22)	1.14 (0.70–1.79)
Muscle pain	65 (16.8)	63 (16.5)	35 (18.1)	0.34	0.33	0.50	0.96 (0.67–1.38)	1.46 (0.94–2.24)
Fever	47 (12.1)	50 (13.1)	27 (14.0)	0.24	0.26	0.38	1.06 (0.70–1.61)	1.56 (0.94–2.56)
Cough	44 (11.4)	42 (11.9)	17 (8.81)	0.23	0.22	0.24	0.95 (0.61–1.48)	1.05 (0.56–1.88)
Joint pain	40 (10.3)	50 (13.1)	30 (15.5)	0.21	0.26	0.42	1.24 (0.80–1.93)	2.04 (1.23–3.36)
Dry mouth	33 (8.53)	33 (8.66)	11 (5.70)	0.17	0.17	0.16	0.99 (0.60–1.66)	0.91 (0.41–1.84)
Insomnia	34 (8.79)	36 (9.45)	17 (8.81)	0.18	0.19	0.24	1.05 (0.64–1.73)	1.36 (0.71–2.50)
Sleep disturbance.	32 (8.27)	33 (8.66)	14 (7.25)	0.17	0.17	0.20	1.03 (0.61–1.72)	1.19 (0.59–2.29)
Anorexia	24 (6.20)	26 (6.82)	10 (5.18)	0.13	0.13	0.14	1.08 (0.59–1.96)	1.13 (0.48–2.46)
Nausea	22 (5.68)	24 (6.30)	11 (5.70)	0.11	0.12	0.16	1.09 (0.58–2.03)	1.36 (0.60–2.92)
Diarrhea	20 (5.17)	24 (6.30)	5 (2.59)	0.10	0.12	0.07	1.19 (0.63–2.28)	0.68 (0.20–1.87)
Itching	15 (3.88)	8 (2.10)	7 (3.63)	0.08	0.04	0.10	0.53 (0.19–1.33)	1.27 (0.44–3.31)
Vomiting	15 (3.88)	10 (2.62)	3 (1.55)	0.08	0.05	0.04	0.66 (0.27–1.58)	0.54 (0.10–1.92)
Fatigue	15 (3.88)	17 (4.46)	11 (5.70)	0.08	0.09	0.16	1.13 (0.53–2.42)	1.99 (0.83–4.65)
Skin rash	4 (1.03)	4 (1.05)	2 (1.04)	0.02	0.02	0.03	0.99 (0.19–5.34)	1.36 (0.12–9.48)
Palpitation	3 (0.78)	5 (1.31)	3 (1.55)	0.02	0.03	0.04	1.66 (0.32–10.7)	2.72 (0.36–20.3)
Back pain	2 (0.52)	4 (1.05)	2 (1.04)	0.01	0.02	0.03	1.99 (0.29–22.0)	2.72 (0.20–37.5)

[a] Number of participants who reported an adverse event at least once.
[b] Per person-year at risk.

Risks associated with DP during pregnancy. Preclinical animal studies [138-140] and clinical studies involving pregnant women [141,142] have not demonstrated significant safety concerns with the use of DP. In a recent study from Kenya, serious adverse events were less frequent in women

randomized to IPTp with DP compared to women randomized to IPTp with SP.[28] In a recent study from our group comparing IPTp with 3 dose SP vs. 3 dose DP vs. monthly DP in Tororo Uganda, study drugs were well tolerated and there were no significant differences in the risk of adverse events.[27] Vomiting occurred < 0.2% of the time after administration of study drugs with no differences between study arms (Table 8). There were no significant differences in the incidence of any adverse events apart from dysphagia, which was higher in the monthly DP arm compared to the 3 dose DP arm. All episodes of dysphagia were mild in severity and we are not aware of any previous reports of DP being associated with dysphagia. Only one grade 3-4 adverse event was possibly related to study drugs; an episode of anemia which occurred both after the 1st and 2nd doses of monthly DP (study drug was subsequently withheld after the 2nd dose)(Table 8). Among 42 women who underwent ECG measurements at 28 weeks gestational age, all pre- and post-dosing QTc intervals were within normal limits ($\leq$ 450 msec) and no clinical adverse events consistent with cardiotoxicity occurred during the course of the study. Median change in QTc intervals was greater in the 3 dose DP (20 msec) and monthly DP (30 msec) arms compared to the 3 dose SP arm (5 msec), but these differences were not statistically significant.

Table 8. Measures of safety and tolerability from IPTp study conducted in Tororo, Uganda

**Outcome**	**Treatment arm**		
	**3 dose SP**	**3 dose DP**	**Monthly DP**
**Prevalence measures**	**no./total no. (%)**	**no./total no. (%)**	**no./total no. (%)**
Vomiting following administration of study drugs			
Observed after administration of 1st dose in clinic	2/617 (0.32)	0/542 (0)	1/594 (0.17)
Reported after administration of 2nd or 3rd dose at home	2/1222 (0.16)	0/1067 (0)	5/1180 (0.42)
**Incidence measures**	**Events [a]**	**Events [a]**	**Events [a]**
Individual adverse events of any severity [b]			
Abdominal pain	172 (3.14)	122 (2.52)	132 (2.47)
Cough	94 (1.72)	71 (1.47)	77 (1.44)
Headache	90 (1.64)	70 (1.45)	78 (1.46)
Chills	21 (0.38)	14 (0.29)	12 (0.22)
Diarrhea	12 (0.22)	10 (0.21)	13 (0.24)
Malaise	16 (0.29)	9 (0.19)	8 (0.15)
Dysphagia	9 (0.16)	2 (0.04)	14 (0.26) [c]
Vomiting	8 (0.15)	8 (0.17)	8 (0.15)
Nausea	2 (0.04)	4 (0.08)	2 (0.04)
Urinary tract infection	3 (0.05)	2 (0.04)	2 (0.04)
Anorexia	2 (0.04)	0 (0)	4 (0.07)
Individual grade 3-4 adverse events			
Anemia	12 (0.22)	4 (0.08)	6 (0.11)
Congenital anomaly	2 (0.04)	4 (0.08)	0 (0)
Stillbirth	1 (0.02)	1 (0.02)	1 (0.02)
Thrombocytopenia	2 (0.04)	0 (0)	0 (0)
Threatened abortion	1 (0.02)	0 (0)	0 (0)
Retained products of conception	0 (0)	1 (0.02)	0 (0)
Preeclampsia	0 (0)	0 (0)	1 (0.02)
Hypotension	0 (0)	0 (0)	1 (0.02)
Pyelonephritis	0 (0)	1 (0.02)	0 (0)
Respiratory distress	0 (0)	1 (0.02)	0 (0)

All grade 3-4 adverse events	18 (0.33)	12 (0.25)	9 (0.17)
Grade 3-4 adverse events possibly related to study drugs	0 (0)	0 (0)	1 (0.02)
All serious adverse events	6 (0.11)	9 (0.19)	4 (0.07)

[a] Number of events (incidence per person year at risk)
[b] Includes only those categories with at least five total events
[c] P=0.02 comparing monthly DP with 3 dose DP

More recently, data from a study our group completed in Busia, Uganda also provides evidence for the excellent safety and tolerability of IPTp with DP.[29] In this study, 782 women were randomized to IPTp with SP or DP given every 4 weeks. Vomiting occurred < 0.1% of the time after administration of study drugs, with no differences between study arms (Table 9). The mean change in the QTc interval after administration of study drugs was significantly higher in the DP arm compared to the SP arm (13 ms vs. 0 ms, p<0·0001) (Table 10). Within the DP arm, the mean change in the QTc interval was greater at 20 weeks (18 ms) compared to 28 weeks (12 ms, p<0·0001) and 36 weeks (10 ms, p<0·0001) gestation. There were seven events in which the QTc interval change was > 60 ms (all in the DP arm). No QTc intervals were > 500 ms. No episode of QTc prolongation was associated with arrhythmias, symptoms or clinical adverse events. There were no significant differences in the incidence of adverse events of any severity (Table 9). Among individual grade 3-4 adverse events, the incidence of congenital anomalies was higher in the DP arm (10 vs. 2 events, p=0·04). All congenital anomalies observed were considered minor and included 10 episodes of polydactyly (9 in the DP arm), a club foot (SP arm) and one infant born with a cleft palate and club foot (DP arm). Among ten grade 3-4 adverse events deemed by the investigators to be possibly related to study drugs, nine were QTc prolongation (all in the DP arm) and one was anemia (SP arm).

These findings are consistent with a recent review from the WHO which concluded there were no significant differences in the risks of cardiotoxicity following treatment with standard doses of DP compared to other commonly used antimalarial drugs such as chloroquine and amodiaquine. Furthermore, the risks of cardiotoxicity with DP were felt to be similar for healthy volunteers and malaria patients.[69] In a recent meta-analysis of almost 200,000 individuals who received DP, the risk of sudden unexplained death was not higher than baseline and the authors concluded that concerns about cardiotoxicity need not limit its use.[71]

Table 9. Measures of safety and tolerability from IPTp study conducted in Busia, Uganda

Outcome	Treatment arm		p value
	Monthly SP	Monthly DP	
**Prevalence measures**	**Prevalence**	**Prevalence**	
Vomiting following administration of study drugs	1/2057 (0·1%)	6/2124 (0·3%)	0·10
Observed after administration of 1st dose in clinic	1/2057 (0·1%)	1/2124 (0·1%)	0·98
Reported after administration of 2nd dose at home	0/2057 (0%)	1/2124 (0·1%)	0·51
Reported after administration of 3rd dose at home			
**Continuous measures**	**Mean (SD)**	**Mean (SD)**	
Change in QTc (following 3rd dose – before 1st dose) in ms			
All measurements (n=2003)	0 (23)	13 (23)	<0·0001
Week 20 gestational age measurements (n=697)	0 (23)	18 (23)	<0·0001
Week 28 gestational age measurements (n=677)	0 (22)	12 (22)	<0·0001
Week 36 gestational age measurements (n=629)	-1 (23)	10 (22)	<0·0001
**Incidence measures**	**Events [a]**	**Events [a]**	
Individual adverse events [b] of any severity [c]			
Abdominal pain	822 (4·50)	800 (4·22)	0·34
Cough	603 (3·30)	681 (3·59)	0·29
Headache	586 (3·21)	604 (3·19)	0·87
Pyuria	63 (0·34)	69 (0·36)	0·78
Diarrhea	62 (0·34)	66 (0·35)	0·90
Malaise	54 (0·30)	52 (0·27)	0·72
Vomiting	44 (0·24)	45 (0·24)	0·96
Chills	27 (0·15)	26 (0·14)	0·80
Individual grade 3-4 adverse events [b]			
Anemia	28 (0·153)	8 (0·042)	0·001
Proteinuria	10 (0·055)	6 (0·032)	0·29
Congenital anomaly	2 (0·011)	10 (0·053)	0·04
Thrombocytopenia	7 (0·038)	3 (0·016)	0·20
QTc interval prolongation	0 (0)	9 (0·047)	0·004
Stillbirth	5 (0·027)	2 (0·011)	0·26
Altered mental status	0 (0)	2 (0·011)	0·50
Elevated alanine aminotransferase	1 (0·005)	1 (0·005)	0·98
Respiratory distress	1 (0·005)	0 (0)	0·50
Hemorrhage	0 (0)	1 (0·005)	0·50
Complicated abortion	0 (0)	1 (0·005)	0·50
All grade 3-4 adverse events	54 (0·295)	43 (0·227)	0·22
All serious adverse events	12 (0·066)	19 (0·100)	0·26
Grade 3-4 adverse events possibly related to study drugs	1 (0·005)	9 (0·047)	0·04

[a] Number of events (incidence per person year at risk)
[b] Individual adverse events were assessed and graded according to standardized criteria at every visit to the study clinic.[143]
[c] Grade 1-4 adverse events. Includes only those with at least 50 total events

Although no clinical trials have studied SP+DP, we have shown that another antifolate combination, trimethoprim-sulfamethoxazole (TMP-SMX), given daily with monthly DP was as safe and well tolerated as daily TMP-SMX alone among 200 HIV infected pregnant women (Table 10).[70] Available studies report no clinically relevant drug interactions for DP and SP, and there is no evidence that they alter the disposition of other drugs, except for one small study reporting increased exposure to halofantrine, a CYP P450 3A4 substrate, after a single dose of SP.[81,83-86] In pharmacokinetic studies, our group and others have reported that pregnancy decreases piperaquine and sulfadoxine exposure, with mixed results for pyrimethamine, suggesting no pregnancy-associated increased risk, although data are limited.[72-76] Piperaquine is metabolized by CYP P450 3A4 and 2C8.[77-79] Sulfadoxine is cleared renally through glomerular filtration[80,81] and pyrimethamine is primarily hepatically metabolized, with 15–30% excreted unchanged in urine.[82]

Table 10. Measures of safety and tolerability comparing daily TMP-SMX alone vs daily TMP-SMX + monthly DP during pregnancy

Outcome	Treatment arm		*P* Value
	Daily TMP-SMX	Daily TMP-SMX + monthly DP	
**Prevalence measures**	**n/N (%)**		
Vomiting of study drugs			
After administration of 1st dose in clinic	0/482 (0%)	1/468 (0.2%)	0.49
After administration of 2nd or 3rd dose at home	0/944 (0%)	2/915 (0.2%)	0.24
**Incidence measures**	**Number of events (incidence per person year at risk)**		
Individual adverse events of any severity[a]			
Abdominal pain	85 (1.85)	74 (1.69)	0.57
Cough	69 (1.50)	55 (1.25)	0.34
Headache	39 (0.85)	43 (0.98)	0.51
Anemia	42 (0.91)	40 (0.91)	0.99
Malaise	27 (0.59)	24 (0.55)	0.80
Diarrhea	17 (0.37)	14 (0.32)	0.76
Chills	11 (0.24)	11 (0.25)	0.91
Thrombocytopenia	11 (0.24)	11 (0.25)	0.91
Vomiting	7 (0.15)	7 (0.16)	0.94
Anorexia	6 (0.13)	4 (0.09)	0.58
Individual grade 3-4 adverse events			
Anemia	10 (0.22)	7 (0.16)	0.44
Congenital anomaly	1 (0.02)	4 (0.09)	0.22
Stillbirth	0 (0)	1 (0.02)	0.99
Respiratory distress	1 (0.02)	0 (0)	0.99
Altered mental status	0 (0)	1 (0.02)	0.99
Elevated alanine aminotransferase	0 (0)	1 (0.02)	0.99
All grade 3-4 adverse events	12 (0.26)	14 (0.32)	0.74
All serious adverse events	4 (0.09)	6 (0.14)	0.55

[a] Includes only those categories with at least ten total events

10.3 Treatment and Compensation for Injury

If a participant is injured as a result of being in this study, treatment will be available through Masafu General Hospital. If there is need for compensation for injury as a result of participation in the study, this will be covered by insurance which is purchased for every study participant by IDRC. Makerere University, UCSF, and DMID do not normally provide any other form of compensation for injury.

10.4 Costs to the Subjects

There will be no cost to the participant for participation in this study.

10.5 Reimbursement of Subjects

Participants will not be paid for their participation in the study. The study will provide all routine medical care, including evaluations, medications available in our clinic, and cost of any transportation free of charge. In addition, we will reimburse the cost of referrals made by study physicians to other clinics, including the resulting cost of the visit, most diagnostic tests (including laboratory test, X-rays, and ultrasounds), and medications, using available funds. However, reimbursement of all diagnostic tests and treatment recommended outside the study clinic cannot be guaranteed in all circumstances.

10.6 IRB Review and Informed Consent

This protocol, all procedures and consent forms, and any subsequent modifications must be reviewed and approved by the IRBs of all the participating institutions in both the U.S. and in Uganda. This includes the UCSF CHR, the MU School of Medicine - Research and Ethics Committee (MU-SOMREC) or MU School of Biomedical Sciences - Research and Ethics Committee (MU-SBSREC), Stanford University Research Compliance Office, and the UNCST.

All consent forms will be translated into the local languages (Samia, Swahili, Luganda, and English) and back-translated into English to ensure correct use of language. Consent forms will be read aloud to parents by trained study interviewers. The informed consent will describe the purpose of the study, all the procedures involved, and the risks and benefits of participation. Study physicians will ask parents/guardians of study participants to summarize the study and explain the reasons why they want to participate. Either a signature or a thumbprint (for participants who cannot read) will be acceptable to confirm informed consent for participation in the study.

10.7 Publication of Research Findings

The findings from this study may be published in a medical journal. No individual identities will be used in any reports or publications resulting from the study. The researchers will publish results of the trial in accordance with NIH, UNCST, UCSF, Stanford University, and Makerere University guidelines.

10.8 Biohazard Containment

As the transmission of HIV and other blood-borne pathogens can occur through contact with contaminated needles, blood, and blood products, appropriate blood and secretion precautions will be employed by all personnel involved in the drawing of blood, exposure to blood and secretions, and shipping and handling of all specimens for this study. We will follow the current guidelines set forth by the Centers for Disease Control and Prevention and the NIH. All infectious specimens will be transported using packaging mandated in the Federal Code of Regulations, CDC 42 CFR Part 72.

11. REFERENCES

1. Rogerson SJ, Unger HW. Prevention and control of malaria in pregnancy - new threats, new opportunities? *Expert Rev Anti Infect Ther* 2017; **15**(4): 361-75.
2. Desai M, ter Kuile FO, Nosten F, et al. Epidemiology and burden of malaria in pregnancy. *Lancet Infect Dis* 2007; **7**(2): 93-104.
3. Guyatt HL, Snow RW. Malaria in pregnancy as an indirect cause of infant mortality in sub-Saharan Africa. *Trans R Soc Trop Med Hyg* 2001; **95**(6): 569-76.
4. Katureebe A, Zinszer K, Arinaitwe E, et al. Measures of Malaria Burden after Long-Lasting Insecticidal Net Distribution and Indoor Residual Spraying at Three Sites in Uganda: A Prospective Observational Study. *PLoS Med* 2016; **13**(11): e1002167.
5. Trape JF, Tall A, Diagne N, et al. Malaria morbidity and pyrethroid resistance after the introduction of insecticide-treated bednets and artemisinin-based combination therapies: a longitudinal study. *The Lancet infectious diseases* 2011; **11**(12): 925-32.
6. Zamawe CO, Nakamura K, Shibanuma A, Jimba M. The effectiveness of a nationwide universal coverage campaign of insecticide-treated bed nets on childhood malaria in Malawi. *Malar J* 2016; **15**(1): 505.
7. Radeva-Petrova D, Kayentao K, ter Kuile FO, Sinclair D, Garner P. Drugs for preventing malaria in pregnant women in endemic areas: any drug regimen versus placebo or no treatment. *Cochrane database of systematic reviews (Online)* 2014; (10): CD000169.

8. Eisele TP, Larsen DA, Anglewicz PA, et al. Malaria prevention in pregnancy, birthweight, and neonatal mortality: a meta-analysis of 32 national cross-sectional datasets in Africa. *Lancet Infect Dis* 2012; **12**(12): 942-9.
9. Kayentao K, Garner P, van Eijk AM, et al. Intermittent preventive therapy for malaria during pregnancy using 2 vs 3 or more doses of sulfadoxine-pyrimethamine and risk of low birth weight in Africa: systematic review and meta-analysis. *JAMA* 2013; **309**(6): 594-604.
10. Bigira V, Kapisi J, Clark TD, et al. Protective efficacy and safety of three antimalarial regimens for the prevention of malaria in young ugandan children: a randomized controlled trial. *PLoS Med* 2014; **11**(8): e1001689.
11. Desai M, Gutman J, Taylor SM, et al. Impact of Sulfadoxine-Pyrimethamine Resistance on Effectiveness of Intermittent Preventive Therapy for Malaria in Pregnancy at Clearing Infections and Preventing Low Birth Weight. *Clin Infect Dis* 2016; **62**(3): 323-33.
12. Okell LC, Griffin JT, Roper C. Mapping sulphadoxine-pyrimethamine-resistant Plasmodium falciparum malaria in infected humans and in parasite populations in Africa. *Scientific reports* 2017; **7**(1): 7389.
13. Gutman J, Kalilani L, Taylor S, et al. The A581G Mutation in the Gene Encoding Plasmodium falciparum Dihydropteroate Synthetase Reduces the Effectiveness of Sulfadoxine-Pyrimethamine Preventive Therapy in Malawian Pregnant Women. *J Infect Dis* 2015; **211**(12): 1997-2005.
14. Harrington WE, Mutabingwa TK, Muehlenbachs A, et al. Competitive facilitation of drug-resistant Plasmodium falciparum malaria parasites in pregnant women who receive preventive treatment. *Proc Natl Acad Sci U S A* 2009; **106**(22): 9027-32.
15. Minja DT, Schmiegelow C, Mmbando B, et al. Plasmodium falciparum mutant haplotype infection during pregnancy associated with reduced birthweight, Tanzania. *Emerg Infect Dis* 2013; **19**(9).
16. Clerk CA, Bruce J, Affipunguh PK, et al. A randomized, controlled trial of intermittent preventive treatment with sulfadoxine-pyrimethamine, amodiaquine, or the combination in pregnant women in Ghana. *J Infect Dis* 2008; **198**(8): 1202-11.
17. Gonzalez R, Mombo-Ngoma G, Ouedraogo S, et al. Intermittent preventive treatment of malaria in pregnancy with mefloquine in HIV-negative women: a multicentre randomized controlled trial. *PLoS Med* 2014; **11**(9): e1001733.
18. Kimani J, Phiri K, Kamiza S, et al. Efficacy and Safety of Azithromycin-Chloroquine versus Sulfadoxine-Pyrimethamine for Intermittent Preventive Treatment of Plasmodium falciparum Malaria Infection in Pregnant Women in Africa: An Open-Label, Randomized Trial. *PLoS ONE* 2016; **11**(6): e0157045.
19. Luntamo M, Kulmala T, Cheung YB, Maleta K, Ashorn P. The effect of antenatal monthly sulphadoxine-pyrimethamine, alone or with azithromycin, on foetal and neonatal growth faltering in Malawi: a randomised controlled trial. *Trop Med Int Health* 2013; **18**(4): 386-97.
20. Luntamo M, Kulmala T, Mbewe B, Cheung YB, Maleta K, Ashorn P. Effect of repeated treatment of pregnant women with sulfadoxine-pyrimethamine and azithromycin on preterm delivery in Malawi: a randomized controlled trial. *Am J Trop Med Hyg* 2010; **83**(6): 1212-20.
21. McGready R, White NJ, Nosten F. Parasitological efficacy of antimalarials in the treatment and prevention of falciparum malaria in pregnancy 1998 to 2009: a systematic review. *BJOG : an international journal of obstetrics and gynaecology* 2011; **118**(2): 123-35.

22. Group PS, Pekyi D, Ampromfi AA, et al. Four Artemisinin-Based Treatments in African Pregnant Women with Malaria. *N Engl J Med* 2016; **374**(10): 913-27.
23. Manyando C, Kayentao K, D'Alessandro U, Okafor HU, Juma E, Hamed K. A systematic review of the safety and efficacy of artemether-lumefantrine against uncomplicated Plasmodium falciparum malaria during pregnancy. *Malar J* 2012; **11**: 141.
24. McGready R, Tan SO, Ashley EA, et al. A randomised controlled trial of artemether-lumefantrine versus artesunate for uncomplicated plasmodium falciparum treatment in pregnancy. *PLoS Med* 2008; **5**(12): e253.
25. Piola P, Nabasumba C, Turyakira E, et al. Efficacy and safety of artemether-lumefantrine compared with quinine in pregnant women with uncomplicated Plasmodium falciparum malaria: an open-label, randomised, non-inferiority trial. *The Lancet infectious diseases* 2010; **10**(11): 762-9.
26. Poespoprodjo JR, Fobia W, Kenangalem E, et al. Treatment policy change to dihydroartemisinin-piperaquine contributes to the reduction of adverse maternal and pregnancy outcomes. *Malar J* 2015; **14**: 272.
27. Desai M, Gutman J, L'Lanziva A, et al. Intermittent screening and treatment or intermittent preventive treatment with dihydroartemisinin-piperaquine versus intermittent preventive treatment with sulfadoxine-pyrimethamine for the control of malaria during pregnancy in western Kenya: an open-label, three-group, randomised controlled superiority trial. *Lancet* 2015; **386**(10012): 2507-19.
28. Kakuru A, Jagannathan P, Muhindo MK, et al. Dihydroartemisinin-Piperaquine for the Prevention of Malaria in Pregnancy. *N Engl J Med* 2016; **374**(10): 928-39.
29. Kajubi R, Ochieng T, Kakuru A, et al. Monthly sulfadoxine-pyrimethamine versus dihydroartemisinin-piperaquine for intermittent preventive treatment of malaria in pregnancy: A randomized controlled trial. *The Lancet* 2019.
30. Madanitsa M, Kalilani L, Mwapasa V, et al. Scheduled Intermittent Screening with Rapid Diagnostic Tests and Treatment with Dihydroartemisinin-Piperaquine versus Intermittent Preventive Therapy with Sulfadoxine-Pyrimethamine for Malaria in Pregnancy in Malawi: An Open-Label Randomized Controlled Trial. *PLoS Med* 2016; **13**(9): e1002124.
31. Tagbor H, Cairns M, Bojang K, et al. A Non-Inferiority, Individually Randomized Trial of Intermittent Screening and Treatment versus Intermittent Preventive Treatment in the Control of Malaria in Pregnancy. *PLoS ONE* 2015; **10**(8): e0132247.
32. World Health Organization. Malaria Policy Advisory Committe Meeting: Malaria in pregnancy. Geneva, Switzerland, 2015.
33. Chico RM, Cano J, Ariti C, et al. Influence of malaria transmission intensity and the 581G mutation on the efficacy of intermittent preventive treatment in pregnancy: systematic review and meta-analysis. *Trop Med Int Health* 2015; **20**(12): 1621-33.
34. Chico RM, Chaponda EB, Ariti C, Chandramohan D. Sulfadoxine-Pyrimethamine Exhibits Dose-Response Protection Against Adverse Birth Outcomes Related to Malaria and Sexually Transmitted and Reproductive Tract Infections. *Clin Infect Dis* 2017; **64**(8): 1043-51.
35. Newman L, Rowley J, Vander Hoorn S, et al. Global Estimates of the Prevalence and Incidence of Four Curable Sexually Transmitted Infections in 2012 Based on Systematic Review and Global Reporting. *PLoS ONE* 2015; **10**(12): e0143304.

36. Adachi K, Nielsen-Saines K, Klausner JD. Chlamydia trachomatis Infection in Pregnancy: The Global Challenge of Preventing Adverse Pregnancy and Infant Outcomes in Sub-Saharan Africa and Asia. *Biomed Res Int* 2016; **2016**: 9315757.
37. Cunnington M, Kortsalioudaki C, Heath P. Genitourinary pathogens and preterm birth. *Current opinion in infectious diseases* 2013; **26**(3): 219-30.
38. Newman L, Kamb M, Hawkes S, et al. Global estimates of syphilis in pregnancy and associated adverse outcomes: analysis of multinational antenatal surveillance data. *PLoS Med* 2013; **10**(2): e1001396.
39. Silver BJ, Guy RJ, Kaldor JM, Jamil MS, Rumbold AR. Trichomonas vaginalis as a cause of perinatal morbidity: a systematic review and meta-analysis. *Sex Transm Dis* 2014; **41**(6): 369-76.
40. Edmond KM, Kortsalioudaki C, Scott S, et al. Group B streptococcal disease in infants aged younger than 3 months: systematic review and meta-analysis. *Lancet* 2012; **379**(9815): 547-56.
41. Schrag SJ, Zywicki S, Farley MM, et al. Group B streptococcal disease in the era of intrapartum antibiotic prophylaxis. *N Engl J Med* 2000; **342**(1): 15-20.
42. Wiesenfeld HC, Manhart LE. Mycoplasma genitalium in Women: Current Knowledge and Research Priorities for This Recently Emerged Pathogen. *J Infect Dis* 2017; **216**(suppl_2): S389-S95.
43. Larsen B, Hwang J. Mycoplasma, Ureaplasma, and adverse pregnancy outcomes: a fresh look. *Infect Dis Obstet Gynecol* 2010; **2010**.
44. Kiss H, Petricevic L, Husslein P. Prospective randomised controlled trial of an infection screening programme to reduce the rate of preterm delivery. *BMJ* 2004; **329**(7462): 371.
45. Leitich H, Bodner-Adler B, Brunbauer M, Kaider A, Egarter C, Husslein P. Bacterial vaginosis as a risk factor for preterm delivery: a meta-analysis. *Am J Obstet Gynecol* 2003; **189**(1): 139-47.
46. Brotman RM, Klebanoff MA, Nansel TR, et al. Bacterial vaginosis assessed by gram stain and diminished colonization resistance to incident gonococcal, chlamydial, and trichomonal genital infection. *J Infect Dis* 2010; **202**(12): 1907-15.
47. McDonald HM, Brocklehurst P, Gordon A. Antibiotics for treating bacterial vaginosis in pregnancy. *Cochrane database of systematic reviews (Online)* 2007; (1): CD000262.
48. Johnson CT, Adami RR, Farzin A. Antibiotic Therapy for Chorioamnionitis to Reduce the Global Burden of Associated Disease. *Front Pharmacol* 2017; **8**: 97.
49. Sweeney EL, Dando SJ, Kallapur SG, Knox CL. The Human Ureaplasma Species as Causative Agents of Chorioamnionitis. *Clin Microbiol Rev* 2017; **30**(1): 349-79.
50. ter Kuile FO, van Eijk AM, Filler SJ. Effect of sulfadoxine-pyrimethamine resistance on the efficacy of intermittent preventive therapy for malaria control during pregnancy: a systematic review. *Jama* 2007; **297**(23): 2603-16.
51. Capan M, Mombo-Ngoma G, Makristathis A, Ramharter M. Anti-bacterial activity of intermittent preventive treatment of malaria in pregnancy: comparative in vitro study of sulphadoxine-pyrimethamine, mefloquine, and azithromycin. *Malar J* 2010; **9**: 303.
52. Capan-Melser M, Mombo Ngoma G, Akerey-Diop D, et al. Evaluation of intermittent preventive treatment of malaria against group B Streptococcus colonization in pregnant women: a nested analysis of a randomized controlled clinical trial of sulfadoxine/pyrimethamine versus mefloquine. *J Antimicrob Chemother* 2015; **70**(6): 1898-902.

53. Wormser GP, Keusch GT, Heel RC. Co-trimoxazole (trimethoprim-sulfamethoxazole): an updated review of its antibacterial activity and clinical efficacy. *Drugs* 1982; **24**(6): 459-518.
54. Church JA, Fitzgerald F, Walker AS, Gibb DM, Prendergast AJ. The expanding role of co-trimoxazole in developing countries. *Lancet Infect Dis* 2015; **15**(3): 327-39.
55. Gregson A, Plowe CV. Mechanisms of resistance of malaria parasites to antifolates. *Pharmacol Rev* 2005; **57**(1): 117-45.
56. Kavishe RA, Kaaya RD, Nag S, et al. Molecular monitoring of Plasmodium falciparum super-resistance to sulfadoxine-pyrimethamine in Tanzania. *Malar J* 2016; **15**: 335.
57. Lynch C, Pearce R, Pota H, et al. Emergence of a dhfr mutation conferring high-level drug resistance in Plasmodium falciparum populations from southwest Uganda. *J Infect Dis* 2008; **197**(11): 1598-604.
58. Tumwebaze P, Tukwasibwe S, Taylor A, et al. Changing Antimalarial Drug Resistance Patterns Identified by Surveillance at Three Sites in Uganda. *J Infect Dis* 2017; **215**(4): 631-5.
59. Tumwebaze P, Conrad MD, Walakira A, et al. Impact of antimalarial treatment and chemoprevention on the drug sensitivity of malaria parasites isolated from ugandan children. *Antimicrob Agents Chemother* 2015; **59**(6): 3018-30.
60. Rasmussen SA, Ceja FG, Conrad MD, et al. Changing Antimalarial Drug Sensitivities in Uganda. *Antimicrob Agents Chemother* 2017; **61**(12).
61. Amato R, Lim P, Miotto O, et al. Genetic markers associated with dihydroartemisinin-piperaquine failure in Plasmodium falciparum malaria in Cambodia: a genotype-phenotype association study. *Lancet Infect Dis* 2017; **17**(2): 164-73.
62. Witkowski B, Duru V, Khim N, et al. A surrogate marker of piperaquine-resistant Plasmodium falciparum malaria: a phenotype-genotype association study. *Lancet Infect Dis* 2017; **17**(2): 174-83.
63. Ross LS, Dhingra SK, Mok S, et al. Emerging Southeast Asian PfCRT mutations confer Plasmodium falciparum resistance to the first-line antimalarial piperaquine. *Nat Commun* 2018; **9**(1): 3314.
64. Yeka A, Wallender E, Mulebeke R, et al. Comparative efficacy of artemether-lumefantrine and dihydroartemisinin-piperaquine for the treatment of uncomplicated malaria in Ugandan children. *J Infect Dis* 2018.
65. Ariey F, Witkowski B, Amaratunga C, et al. A molecular marker of artemisinin-resistant Plasmodium falciparum malaria. *Nature* 2014; **505**(7481): 50-5.
66. Conrad MD, Bigira V, Kapisi J, et al. Polymorphisms in K13 and falcipain-2 associated with artemisinin resistance are not prevalent in Plasmodium falciparum isolated from Ugandan children. *PLoS ONE* 2014; **9**(8): e105690.
67. Cooper RA, Conrad MD, Watson QD, et al. Lack of Artemisinin Resistance in Plasmodium falciparum in Uganda Based on Parasitological and Molecular Assays. *Antimicrob Agents Chemother* 2015; **59**(8): 5061-4.
68. Muhindo MK, Kakuru A, Jagannathan P, et al. Early parasite clearance following artemisinin-based combination therapy among Ugandan children with uncomplicated Plasmodium falciparum malaria. *Malar J* 2014; **13**: 32.
69. World Health Organization. Malaria Policy Advisory Committe Meeting: The cardiotoxicity of antimalarials. 2017. http://www.who.int/malaria/mpac/mpac-mar2017-erg-cardiotoxicity-report-session2.pdf.

70. Natureeba P, Kakuru A, Muhindo M, et al. Intermittent Preventive Treatment With Dihydroartemisinin-Piperaquine for the Prevention of Malaria Among HIV-Infected Pregnant Women. *J Infect Dis* 2017; **216**(1): 29-35.
71. Chan XHS, Win YN, Mawer LJ, Tan JY, Brugada J, White NJ. Risk of sudden unexplained death after use of dihydroartemisinin-piperaquine for malaria: a systematic review and Bayesian meta-analysis. *Lancet Infect Dis* 2018; **18**(8): 913-23.
72. de Kock M, Tarning J, Workman L, et al. Pharmacokinetics of Sulfadoxine and Pyrimethamine for Intermittent Preventive Treatment of Malaria During Pregnancy and After Delivery. *CPT Pharmacometrics Syst Pharmacol* 2017; **6**(7): 430-8.
73. Green MD, van Eijk AM, van Ter Kuile FO, et al. Pharmacokinetics of sulfadoxine-pyrimethamine in HIV-infected and uninfected pregnant women in Western Kenya. *J Infect Dis* 2007; **196**(9): 1403-8.
74. Kajubi R, Huang L, Jagannathan P, et al. Antiretroviral Therapy With Efavirenz Accentuates Pregnancy-Associated Reduction of Dihydroartemisinin-Piperaquine Exposure During Malaria Chemoprevention. *Clinical pharmacology and therapeutics* 2017; **102**(3): 520-8.
75. Karunajeewa HA, Salman S, Mueller I, et al. Pharmacokinetic properties of sulfadoxine-pyrimethamine in pregnant women. *Antimicrob Agents Chemother* 2009; **53**(10): 4368-76.
76. Nyunt MM, Adam I, Kayentao K, et al. Pharmacokinetics of sulfadoxine and pyrimethamine in intermittent preventive treatment of malaria in pregnancy. *Clinical pharmacology and therapeutics* 2010; **87**(2): 226-34.
77. Aweeka FT, Hu C, Huang L, et al. Alteration in cytochrome P450 3A4 activity as measured by a urine cortisol assay in HIV-1-infected pregnant women and relationship to antiretroviral pharmacokinetics. *HIV Med* 2015; **16**(3): 176-83.
78. Lee TM, Huang L, Johnson MK, et al. In vitro metabolism of piperaquine is primarily mediated by CYP3A4. *Xenobiotica* 2012; **42**(11): 1088-95.
79. Stek AM, Mirochnick M, Capparelli E, et al. Reduced lopinavir exposure during pregnancy. *AIDS* 2006; **20**(15): 1931-9.
80. Costantine MM. Physiologic and pharmacokinetic changes in pregnancy. *Front Pharmacol* 2014; **5**: 65.
81. German PI, Aweeka FT. Clinical pharmacology of artemisinin-based combination therapies. *Clin Pharmacokinet* 2008; **47**(2): 91-102.
82. Kerb R, Fux R, Morike K, et al. Pharmacogenetics of antimalarial drugs: effect on metabolism and transport. *Lancet Infect Dis* 2009; **9**(12): 760-74.
83. Baune B, Flinois JP, Furlan V, et al. Halofantrine metabolism in microsomes in man: major role of CYP 3A4 and CYP 3A5. *J Pharm Pharmacol* 1999; **51**(4): 419-26.
84. Chhonker YS, Bhosale VV, Sonkar SK, et al. Assessment of Clinical Pharmacokinetic Drug-Drug Interaction of Antimalarial Drugs alpha/beta-Arteether and Sulfadoxine-Pyrimethamine. *Antimicrob Agents Chemother* 2017; **61**(9).
85. Hombhanje FW. Effect of a single oral dose of Fansidar on the pharmacokinetics of halofantrine in healthy volunteers: a preliminary report. *British journal of clinical pharmacology* 2000; **49**(3): 283-4.
86. Tekete MM, Toure S, Fredericks A, et al. Effects of amodiaquine and artesunate on sulphadoxine-pyrimethamine pharmacokinetic parameters in children under five in Mali. *Malar J* 2011; **10**: 275.

87. Savic RM, Jagannathan P, Kajubi R, et al. Intermittent Preventive Treatment for Malaria in Pregnancy: Optimization of Target Concentrations of Dihydroartemisinin-Piperaquine. *Clin Infect Dis* 2018; **67**(7): 1079-88.
88. Dellicour S, Tatem AJ, Guerra CA, Snow RW, ter Kuile FO. Quantifying the number of pregnancies at risk of malaria in 2007: a demographic study. *PLoS Med* 2010; **7**(1): e1000221.
89. Walker PG, ter Kuile FO, Garske T, Menendez C, Ghani AC. Estimated risk of placental infection and low birthweight attributable to Plasmodium falciparum malaria in Africa in 2010: a modelling study. *The Lancet Global health* 2014; **2**(8): e460-7.
90. Ranson H, N'Guessan R, Lines J, Moiroux N, Nkuni Z, Corbel V. Pyrethroid resistance in African anopheline mosquitoes: what are the implications for malaria control? *Trends Parasitol* 2011; **27**(2): 91-8.
91. ABC Study Group. A head-to-head comparison of four artemisinin-based combinations for treating uncomplicated malaria in African children: a randomized trial. *PLoS medicine* 2011; **8**(11): e1001119.
92. White NJ. Intermittent presumptive treatment for malaria. *PLoS Med* 2005; **2**(1): e3.
93. Committee WHOMPA, Secretariat. Malaria Policy Advisory Committee to the WHO: conclusions and recommendations of eighth biannual meeting (September 2015). *Malar J* 2016; **15**: 117.
94. Chico RM, Moss WJ. Prevention of malaria in pregnancy: a fork in the road? *Lancet* 2015; **386**(10012): 2454-6.
95. Rogerson SJ, Hviid L, Duffy PE, Leke RF, Taylor DW. Malaria in pregnancy: pathogenesis and immunity. *The Lancet infectious diseases* 2007; **7**(2): 105-17.
96. Redline RW, Faye-Petersen O, Heller D, et al. Amniotic infection syndrome: nosology and reproducibility of placental reaction patterns. *Pediatr Dev Pathol* 2003; **6**(5): 435-48.
97. Hillier SL, Martius J, Krohn M, Kiviat N, Holmes KK, Eschenbach DA. A case-control study of chorioamnionic infection and histologic chorioamnionitis in prematurity. *N Engl J Med* 1988; **319**(15): 972-8.
98. Kapisi J, Kakuru A, Jagannathan P, et al. Relationships between infection with Plasmodium falciparum during pregnancy, measures of placental malaria, and adverse birth outcomes. *Malar J* 2017; **16**(1): 400.
99. Hofmann N, Mwingira F, Shekalaghe S, Robinson LJ, Mueller I, Felger I. Ultra-sensitive detection of Plasmodium falciparum by amplification of multi-copy subtelomeric targets. *PLoS Med* 2015; **12**(3): e1001788.
100. Wynn A, Ramogola-Masire D, Gaolebale P, et al. Prevalence and treatment outcomes of routine Chlamydia trachomatis, Neisseria gonorrhoeae and Trichomonas vaginalis testing during antenatal care, Gaborone, Botswana. *Sex Transm Infect* 2017.
101. Gaydos CA, Klausner JD, Pai NP, Kelly H, Coltart C, Peeling RW. Rapid and point-of-care tests for the diagnosis of Trichomonas vaginalis in women and men. *Sex Transm Infect* 2017; **93**(S4): S31-S5.
102. Gaydos CA, Van Der Pol B, Jett-Goheen M, et al. Performance of the Cepheid CT/NG Xpert Rapid PCR Test for Detection of Chlamydia trachomatis and Neisseria gonorrhoeae. *Journal of clinical microbiology* 2013; **51**(6): 1666-72.

103. Gouvea MIS, Joao EC, Teixeira MLB, et al. Accuracy of a rapid real-time polymerase chain reaction assay for diagnosis of group B Streptococcus colonization in a cohort of HIV-infected pregnant women. *J Matern Fetal Neonatal Med* 2017; **30**(9): 1096-101.
104. Nugent RP, Krohn MA, Hillier SL. Reliability of diagnosing bacterial vaginosis is improved by a standardized method of gram stain interpretation. *Journal of clinical microbiology* 1991; **29**(2): 297-301.
105. Strauss RA, Eucker B, Savitz DA, Thorp JM, Jr. Diagnosis of bacterial vaginosis from self-obtained vaginal swabs. *Infect Dis Obstet Gynecol* 2005; **13**(1): 31-5.
106. Goodrich JK, Di Rienzi SC, Poole AC, et al. Conducting a microbiome study. *Cell* 2014; **158**(2): 250-62.
107. Quince C, Walker AW, Simpson JT, Loman NJ, Segata N. Shotgun metagenomics, from sampling to analysis. *Nat Biotechnol* 2017; **35**(9): 833-44.
108. Edwards U, Rogall T, Blocker H, Emde M, Bottger EC. Isolation and direct complete nucleotide determination of entire genes. Characterization of a gene coding for 16S ribosomal RNA. *Nucleic Acids Res* 1989; **17**(19): 7843-53.
109. David LA, Maurice CF, Carmody RN, et al. Diet rapidly and reproducibly alters the human gut microbiome. *Nature* 2014; **505**(7484): 559-63.
110. Schoch CL, Seifert KA, Huhndorf S, et al. Nuclear ribosomal internal transcribed spacer (ITS) region as a universal DNA barcode marker for Fungi. *Proc Natl Acad Sci U S A* 2012; **109**(16): 6241-6.
111. Caporaso JG, Kuczynski J, Stombaugh J, et al. QIIME allows analysis of high-throughput community sequencing data. *Nat Methods* 2010; **7**(5): 335-6.
112. Callahan BJ, McMurdie PJ, Rosen MJ, Han AW, Johnson AJ, Holmes SP. DADA2: High-resolution sample inference from Illumina amplicon data. *Nat Methods* 2016; **13**(7): 581-3.
113. Wang Q, Garrity GM, Tiedje JM, Cole JR. Naive Bayesian classifier for rapid assignment of rRNA sequences into the new bacterial taxonomy. *Appl Environ Microbiol* 2007; **73**(16): 5261-7.
114. Fernandes AD, Reid JN, Macklaim JM, McMurrough TA, Edgell DR, Gloor GB. Unifying the analysis of high-throughput sequencing datasets: characterizing RNA-seq, 16S rRNA gene sequencing and selective growth experiments by compositional data analysis. *Microbiome* 2014; **2**: 15.
115. Langille MG, Zaneveld J, Caporaso JG, et al. Predictive functional profiling of microbial communities using 16S rRNA marker gene sequences. *Nat Biotechnol* 2013; **31**(9): 814-21.
116. Yu G, Wang LG, Han Y, He QY. clusterProfiler: an R package for comparing biological themes among gene clusters. *OMICS* 2012; **16**(5): 284-7.
117. David LA, Weil A, Ryan ET, et al. Gut microbial succession follows acute secretory diarrhea in humans. *MBio* 2015; **6**(3): e00381-15.
118. Langmead B, Trapnell C, Pop M, Salzberg SL. Ultrafast and memory-efficient alignment of short DNA sequences to the human genome. *Genome Biol* 2009; **10**(3): R25.
119. Kanehisa M, Goto S, Sato Y, Furumichi M, Tanabe M. KEGG for integration and interpretation of large-scale molecular data sets. *Nucleic Acids Res* 2012; **40**(Database issue): D109-14.
120. Zhao Y, Tang H, Ye Y. RAPSearch2: a fast and memory-efficient protein similarity search tool for next-generation sequencing data. *Bioinformatics* 2012; **28**(1): 125-6.

121. Nayfach S, Pollard KS. Average genome size estimation improves comparative metagenomics and sheds light on the functional ecology of the human microbiome. *Genome Biol* 2015; **16**: 51.
122. Law CW, Chen Y, Shi W, Smyth GK. voom: Precision weights unlock linear model analysis tools for RNA-seq read counts. *Genome Biol* 2014; **15**(2): R29.
123. Wu D, Smyth GK. Camera: a competitive gene set test accounting for inter-gene correlation. *Nucleic Acids Res* 2012; **40**(17): e133.
124. Truong DT, Franzosa EA, Tickle TL, et al. MetaPhlAn2 for enhanced metagenomic taxonomic profiling. *Nat Methods* 2015; **12**(10): 902-3.
125. Scher JU, Sczesnak A, Longman RS, et al. Expansion of intestinal Prevotella copri correlates with enhanced susceptibility to arthritis. *Elife* 2013; **2**: e01202.
126. LeClair NP, Conrad MD, Baliraine FN, Nsanzabana C, Nsobya SL, Rosenthal PJ. Optimization of a ligase detection reaction-fluorescent microsphere assay for characterization of resistance-mediating polymorphisms in African samples of Plasmodium falciparum. *Journal of clinical microbiology* 2013; **51**(8): 2564-70.
127. Nankoberanyi S, Mbogo GW, LeClair NP, et al. Validation of the ligase detection reaction fluorescent microsphere assay for the detection of Plasmodium falciparum resistance mediating polymorphisms in Uganda. *Malar J* 2014; **13**: 95.
128. Conrad MD, LeClair N, Arinaitwe E, et al. Comparative impacts over 5 years of artemisinin-based combination therapies on Plasmodium falciparum polymorphisms that modulate drug sensitivity in Ugandan children. *J Infect Dis* 2014; **210**(3): 344-53.
129. Hathaway NJ, Parobek CM, Juliano JJ, Bailey JA. SeekDeep: single-base resolution de novo clustering for amplicon deep sequencing. *Nucleic Acids Res* 2017.
130. Kjellin LL, Dorsey G, Rosenthal PJ, Aweeka F, Huang L. Determination of the antimalarial drug piperaquine in small volume pediatric plasma samples by LC-MS/MS. *Bioanalysis* 2014; **6**(23): 3081-9.
131. Schmiegelow C, Scheike T, Oesterholt M, et al. Development of a fetal weight chart using serial trans-abdominal ultrasound in an East African population: a longitudinal observational study. *PLoS ONE* 2012; **7**(9): e44773.
132. Phillips-Howard PA, West LJ. Serious adverse drug reactions to pyrimethamine-sulphadoxine, pyrimethamine-dapsone and to amodiaquine in Britain. *Journal of the Royal Society of Medicine* 1990; **83**(2): 82-5.
133. Miller KD, Lobel HO, Satriale RF, Kuritsky JN, Stern R, Campbell CC. Severe cutaneous reactions among American travelers using pyrimethamine-sulfadoxine (Fansidar) for malaria prophylaxis. *Am J Trop Med Hyg* 1986; **35**(3): 451-8.
134. World Health Organization. Updated WHO Policy Recommendation (October 2012). Intermittent Preventive Treatment of malaria in pregnancy using Sulfadoxine-Pyrimethamine (IPTp-SP). Geneva, 2012.
135. Myint HY, Ashley EA, Day NP, Nosten F, White NJ. Efficacy and safety of dihydroartemisinin-piperaquine. *Trans R Soc Trop Med Hyg* 2007; **101**(9): 858-66.
136. Lwin KM, Phyo AP, Tarning J, et al. Randomized, double-blind, placebo-controlled trial of monthly versus bimonthly dihydroartemisinin-piperaquine chemoprevention in adults at high risk of malaria. *Antimicrobial agents and chemotherapy* 2012; **56**(3): 1571-7.

137. Gutman J, Kovacs S, Dorsey G, Stergachis A, Ter Kuile FO. Safety, tolerability, and efficacy of repeated doses of dihydroartemisinin-piperaquine for prevention and treatment of malaria: a systematic review and meta-analysis. *Lancet Infect Dis* 2017; **17**(2): 184-93.
138. Longo M, Zanoncelli S, Della Torre P, et al. Investigations of the effects of the antimalarial drug dihydroartemisinin (DHA) using the Frog Embryo Teratogenesis Assay-Xenopus (FETAX). *Reprod Toxicol* 2008; **25**(4): 433-41.
139. Longo M, Zanoncelli S, Manera D, et al. Effects of the antimalarial drug dihydroartemisinin (DHA) on rat embryos in vitro. *Reprod Toxicol* 2006; **21**(1): 83-93.
140. Longo M, Zanoncelli S, Torre PD, et al. In vivo and in vitro investigations of the effects of the antimalarial drug dihydroartemisinin (DHA) on rat embryos. *Reprod Toxicol* 2006; **22**(4): 797-810.
141. Rijken MJ, McGready R, Boel ME, et al. Dihydroartemisinin-piperaquine rescue treatment of multidrug-resistant Plasmodium falciparum malaria in pregnancy: a preliminary report. *The American journal of tropical medicine and hygiene* 2008; **78**(4): 543-5.
142. Rijken MJ, McGready R, Phyo AP, et al. Pharmacokinetics of dihydroartemisinin and piperaquine in pregnant and nonpregnant women with uncomplicated falciparum malaria. *Antimicrobial agents and chemotherapy* 2011; **55**(12): 5500-6.
143. Division of AIDS (DAIDS) Table for Grading the Severity of Adult and Pediatric Adverse Events. Version 2.0 November 2014. https://rsc.tech-res.com/docs/default-source/safety/daids_ae_grading_table_v2_nov2014.pdf.

12. APPENDICES

Appendix A. Information Sheet

A STUDY ON PREVENTING MALARIA IN PREGNANT WOMEN

Makerere University in Uganda and the University of California, San Francisco in the United States are combining efforts in Busia district, Uganda to study new ways of using malaria drugs to prevent malaria in pregnant women.

Malaria during pregnancy can have a harmful effect on you or your child. We want to study 2 different malaria drugs alone and in combination to see if they can be used to prevent malaria if taken during pregnancy.

Our study clinic is located at Masafu General Hospital next to the antenatal clinic and is open every day from 8:00 am to 5:00 pm

We want to enroll pregnant women who are at least 16 years old and follow them during pregnancy.

Women will receive free medical care.

We shall also give reimbursement for transport to and from our study clinic

For more information, please come to our study clinic where our doctors will be happy to talk to you and see if you can be in the study.

Appendix B. Determination of Gestational Age

Gestational age will be based on the first day of the last menstrual period (LMP) and the earliest available ultrasound performed at ≥ 6 weeks gestation. The estimated due date (EDD) is calculated as 280 days following the LMP.

If the first available ultrasound is consistent with a gestational age of 6 weeks 0 days to 8 weeks and 6 days, and the ultrasound gestational age is within 5 days of that given LMP, then the LMP will be used to determine gestational age. However, if the ultrasound gestational age differs from the LMP gestational age by more than 5 days, then the ultrasound will be used to determine gestational age.

If the first available ultrasound is consistent with a gestational age of 9 weeks 0 days to 15 weeks 6 days, and the ultrasound gestational age is within 7 days of that given LMP, then the LMP will be used to determine gestational age. However, if the ultrasound gestational age differs from the LMP gestational age by more than 7 days, then the ultrasound will be used to determine gestational age.

If the first available ultrasound is consistent with a gestational age of 16 weeks to 21 weeks 6 days, and the ultrasound gestational age is within 10 days of that given by the LMP, then the LMP will be used to determine gestational age. However, if the ultrasound gestational age differs from the LMP by more than 10 days, then the ultrasound will be used to determine gestational age.

If the first available ultrasound is consistent with a gestational age of 22 weeks to 27weeks 6 days, and the ultrasound gestational age is within 14 days of that given by the LMP, then the LMP will be used to determine gestational age. However, if the ultrasound gestational age differs from the LMP by more than 14 days, then the ultrasound will be used to determine gestational age.

If the first available ultrasound is consistent with a gestational age of 28 weeks or more, and the ultrasound gestational age is within 21 days of that given by the LMP, then the LMP will be used to determine gestational age. However, if the ultrasound gestational age differs from the LMP by more than 21 days, then the ultrasound will be used to determine gestational age. Care here should be taken to rule out intrauterine growth restriction.

If the LMP is not known, then the earliest ultrasound performed at ≥ 6 weeks will be used to determine gestational age.

Participants must be between 12 weeks 0 days to 20 weeks 6 days of gestational age by best obstetric determination at the time of enrollment.

Appendix C. Household survey

The household survey will be administered through a completely paperless QDS software system using hand-held tablet computers. A list of questions that will be used in the survey is provided below.

Ques. No.	Variable Name	Question
**Section 1: Identification**		
1	VISDATE	Date of final visit
2	STARTIME	Start time of interview
4	BC	Cohort Number
3	PPTIDA	Study Participant ID
5	PPTID	To ensure data integrity, please re-enter the Participant ID.
7	INTNUM	Interviewer number
8	AGREE	Are you going to conduct the interview with this household?
**Section 2: Household Characteristics**		
9	SWATER	What is the main source of drinking water for members of your household?
10	OTHERSCS	Specify other source of water
11	TFACLTY	What kind of toilet facility do members of your household usually use?
12	OTHERFCY	Specify other kind of toilet facilities
13	ELECTRIC	Does your household have...<br><br>...Electricity?
14	RADIO	...Radio?
15	CASSETTE	…Cassette player?
16	TV	…Television?
17	MOBILE	…Mobile phone?
18	PHONE	…Fixed phone?
19	FRIDGE	…Refrigerator?
20	TABLE	…Table?
21	CHAIR	…Chairs?
22	SOFA	…Sofa set?
23	BED	…Bed?
24	CUPBOARD	…Cupboard?
25	CLOCK	…Clock?
26	FUELTYPE	What type of fuel does your household mainly use for cooking?
27	OTHERFUE	Specify other type of fuel used
28	SENERGY	What is the main source of energy for lighting in the household?
29	OTHERENG	Specify other source of energy for lighting
30	MMFLOOR	MAIN MATERIAL OF THE FLOOR<br><br>RECORD OBSERVATION.<br><br>MARK ONLY ONE.
31	OTHERMMF	Specify other material of the floor
32	MMROOF	MAIN MATERIAL OF THE ROOF.<br><br>RECORD OBSERVATION.<br><br>MARK ONLY ONE.

33	OTHERMMR	Specify other material of the roof
34	MMEWALLS	MAIN MATERIAL OF THE EXTERIOR WALLS.<br><br>RECORD OBSERVATION.<br><br>MARK ONLY ONE.
35	OTHERMME	Specify other material of the exterior walls
36	HHROOMS	How many rooms in your household are used for sleeping?<br><br>(INCLUDING ROOMS OUTSIDE THE MAIN DWELLING)<br><br>If there are 15 or more rooms, enter 15
37	HHSPACES	How many sleeping spaces like mats, mattresses, or beds are available in your household?<br><br>If there are 25 or more sleeping places, enter 25
38	WATCH	Does any member of your household own or have…<br><br>…A watch?
39	BICYCLE	….A bicycle?
40	SCOOTER	…A motorcycle or motor scooter?
41	CART	…An animal-drawn cart?
42	CAR	…A car or truck?
43	MBOAT	…A boat with a motor?
44	NOMBOAT	…A boat without a motor?
45	BANKACCO	…A bank account?
46	NUMALAND	How many acres of agricultural land do members of this household own?
47	DMARKT	How far is it to the nearest market place?
48	HHMEALS	Now I would like to ask you about the food your household eats.<br>How many meals does your household usually have per day?
49	HHNUMT	In the past week, on how many days did the household eat meat?
50	HHPSF	How often in the last year did you have problems in satisfying the food needs of the household?
51	DHFCTY	How far is it to the nearest health facility?
52	MTHFCTY	If you were to go this facility, how would you most likely go there?
53	OTHERMTH	Specify other means of transport to the health facility
54	PSPRAY	At any time in the past 12 months, has anyone asked permission to come into your dwelling to spray the interior walls against mosquitoes?
55	GPSPRAY	Did you grant them permission to spray the interior walls of your dwelling?
56	RGSPRAY	What was the primary reason that you did not grant permission to spray the interior walls of your dwelling against mosquitoes?
57	OTHERRGS	Specify other reasons for not granting permission to spray the interior walls of your dwelling against mosquitoes
58	TSPRAY	How many months ago was the dwelling last sprayed?
59	WSPRAY	Who sprayed the dwelling?
60	OTHERWSP	Specify other people who sprayed the dwelling
61	DSPRAY	Did you pay for the dwelling to be sprayed?
62	PPWALLS	Since the spraying, have the walls in your dwelling been plastered or painted?
63	TPPWALLS	How many months ago were the walls plastered or painted?
64	MSPRAY	In the past 12 months, have you seen or heard any messages about spraying the interior walls of your dwelling against mosquitoes?

65	MSGA	Where did you hear or see message(s)?<br><br>...Radio?
66	MSGB	...TV?
67	MSGC	...Newspaper/Leaflet?
68	MSGD	…Health worker/CMD?
69	MSGE	…Neighbor/Relative/Friend
70	MSGF	...Community Leader?
71	MSGG	…Village public address system
72	MSGH	...Don't know
73	MSGI	…Other
74	OTHERMSG	Specify other
75	AHWKER	Is there a community health worker (community medicine distributor/CMD, village health team/VHT, community own resource person/CORP) who distributes malaria medicines in your village or community?
76	AMCHWKER	Does the community health worker currently have malaria medicines available?
**Section 3: Study Participants Sleeping Area Characteristics** All questions in this section will be repeated with variable names entopen1, entcov1, etc. for each entryway and window in the study participant's room		
77	SRENTRY	OBSERVATION: How many entryways into the room are there?
78	ENTOPN1	OBSERVATION: Does it open to the outside?
79	ENTCOV1	OBSERVATION: Is the entry way covered?
80	ETMM1	OBSERVATION: Main material is the covering made of.
81	OTHCOV1	Specify Other covering type
82	SRWINDOW	OBSERVATION: How many windows are in the room?
83	WNDCOV1	OBSERVATION: Is the window covered?
84	WNDOPN1	OBSERVATION: Does the window open to the outside
85	SREAVES	OBSERVATION: Does the room have eaves?
86	EAVESCOV	OBSERVATION: If room has eaves, are the eaves covered?
87	EAVESOPN	OBSERVATION: Do the eaves open to the outside?
88	AIRBRICK	OBSERVATION: Does the room have airbricks?
89	AIRBRCOV	OBSERVATION: If the room has airbricks, are the airbricks covered?
90	AIRBROPN	OBSERVATION: Do the airbricks open to the outside?
91	AIRBRNUM	OBSERVATION: How many airbricks are in the room?
92	SLEEP	Where does the study participant usually sleep?
93	OTHERSL	Specify other sleeping area
94	SRSLNUM	Usually, how many people sleep in the same room as the study participant (excluding the study participant)?
95	SRSLNUM5	How many of those people are under 5 years old (excluding the study participant)?
96	SLAREAS	How many sleeping areas are in the room where the study participant sleeps?
97	SASLNUM	How many people sleep in the same bed/sleeping area as the study participant under the mosquito net (excluding the study participant)?
98	SASLNUM5	How many of those people are under 5 years old (excluding the study participant)?
**Section 4: Bednets** All questions in this section will be repeated with variable names obs2, mnths2, where2, etc. for each mosquito net in the household (hhnumnet)		
99	HHAMNETS	IMMEDIATELY BEFORE Enrollment, did your household have any mosquito nets that can be used while sleeping?
100	HNUMNETS	IMMEDIATELY BEFORE study enrollment, How many mosquito nets did your household have?
101	OBS1	May I have a look at (all) the net(s) to establish the brand?
102	MNTHS1	How many months ago did your household obtain the mosquito net?

103	WHERE1	Where did you get the mosquito net from?
104	SPCFRO1	Specify other sources of the mosquito net
105	BRAND1	OBSERVE OR ASK THE BRAND OR TYPE OF MOSQUITO NET.
106	OTHERB1	Specify other brands or types of mosquito net
107	SMNET1	Since you got the mosquito net, was it ever soaked or dipped in a liquid to repel mosquitoes or bugs?
108	TSMNET1	How many months ago was the net last soaked or dipped?
109	SLPNET1	Did anyone sleep under this mosquito net last night?
110	NUSED1A	What are some of the reasons why this net was not used?<br><br>...Too hot
111	NUSED1B	...Don't like smell
112	NUSED1C	...No mosquitoes
113	NUSED1D	...Net too old/too many holes
114	NUSED1E	...Net not hung
115	NUSED1F	...Net too dirty
116	NUSED1G	...Net no longer kill insects
117	NUSED1H	...Don’t know
118	NUSED1I	...Other
119	NTHUNG1	If not hung, why not?
120	OTHRNT1	Specify other reason why the net was not hung.
**Section 5: Interviewer Details**		
122	STOPTIME	End time of interview
123	VSTATUS	Result of Visit
124	OVSTATUS	Specify other result
125	TOTVISIT	Total number of visits
126	COMMENTS	Interviewer's Comments

Appendix D. Administration of study drugs and placebos

Timing of administration of study drugs during pregnancy

Weeks of gestation	Treatment arm		
	**SP every 4 weeks**	**DP every 4 weeks**	**SP + DP every 4 weeks**
16	SP + DP placebo	DP + SP placebo	SP + DP
20	SP + DP placebo	DP + SP placebo	SP + DP
24	SP + DP placebo	DP + SP placebo	SP + DP
28	SP + DP placebo	DP + SP placebo	SP + DP
32	SP + DP placebo	DP + SP placebo	SP + DP
36	SP + DP placebo	DP + SP placebo	SP + DP
40	SP + DP placebo	DP + SP placebo	SP + DP

Appendix E. WHO Criteria for Severe Malaria

Detection of malaria parasites by microscopy and any of the following:

Cerebral malaria (unarousable coma not attributable to any other cause)
Generalized convulsions ($\geq$ 3 convulsions over 24 hours period)
Severe normocytic anemia (Hb < 5 gm/dL)
Hypoglycemia
Metabolic acidosis with respiratory distress
Fluid and electrolyte disturbances

1. Acute renal failure
2. Acute pulmonary edema and adult respiratory distress syndrome (ARDS)
3. Circulatory collapse, shock, septicemia ("algid malaria")
4. Abnormal bleeding
5. Jaundice

Appendix F. Uganda Ministry of Health Guidelines for Routine Care of Pregnant and Postpartum Women, and Newborns

In addition to receiving medical care as described above in the protocol, women and their newborns will receive standard routine prenatal and postpartum care according to Uganda Ministry of Health guidelines. These standard procedures are subject to availability at the local health care facilities.

Routine antenatal care. Women enrolled in the study will receive routine care as designated in the Uganda Ministry of Health Guidelines. Routine antenatal care includes screening and treating for syphilis and syndromic management of sexually transmitted infections (STIs). Pregnant women will receive iron and folic acid supplementation. In addition, women will be given multivitamins that will be given once daily. In addition, women receive mebendazole 500mg as a single dose as early as possible after the 1st trimester. Each antenatal visit also includes blood pressure assessment and urine dipstick for proteinuria.

Routine intrapartum/delivery care. Routine delivery care for in-hospital births will include labor management by the midwifery staff and management of obstetrical complications as per Ministry of Health guidelines. Immediate postpartum infant care will include polio and BCG immunization, ophthalmic tetracycline, and vitamin K.

Routine postpartum care. All postpartum women will receive vitamin A supplementation (200,000 IU) immediately following delivery. Depending on clinical circumstances and based on local standard of care, women may receive 2 weeks of multivitamins twice a day. Common indications for postpartum multivitamins include anemia, postpartum hemorrhage and prolonged labor. Women will be seen at 1 week after delivery as per Ugandan standards of care. Women also undergo a 4-week postpartum visit as part of routine care. These visits include an abdominal exam, syndromic management of STIs, and follow-up on any obstetrical complications that occurred. In addition, women receive vitamin A at this visit, if not given immediately postpartum, and are continued on iron and folic acid supplementation. Pelvic and breast exam will be done if clinically indicated. Contraceptive counseling is performed at this visit as is a nutritional assessment and infant feeding and support.

Appendix G. Schedule of routine assessments and procedures in pregnant women

Evaluations and Interventions	Enrollment	Weeks of gestation							Delivery	1 and 4 weeks postpartum
		16*	20	24	28	32	36	40		
**Informed consent**	X									
**HIV testing[1]**	X				X				X	
**Obstetrical ultrasound[2]**	X			X		X	X			
**Blood collected by phlebotomy for blood smear, qPCR, dried blood spots, CBC, plasma for PK, and immunology studies**	X		X**		X		X		X	
**Blood collected by finger prick for blood smear, qPCR, plasma for PK, and dried blood spot**		X	X**	X		X		X		
**Routine assessment in the study clinic[3]**	X	X	X	X	X	X	X	X		X
**Administration of study drugs**		X	X	X	X	X	X	X		
**Collection of cord blood and placental blood/tissue**									X	
**Labor and delivery documentation[4]**									X	
**Collection of gut and vaginal samples for microbiome studies**	X					X				
**Collection samples for testing of RTIs**									X	
Standard Care										
**Obstetrical exam[5]**	X	X	X	X	X	X	X	X	X	X
**Syphilis screening**	X									
**Iron and Folic Acid**	X	X	X	X	X	X	X	X		
**Prenatal vitamins**	X									
**Mebendazole[6]**		X								
**Vitamin A[7]**									X	
**Insecticide treated bednet**	X									

***** Only if study subject enrolled prior to 16 weeks gestation

** For participants enrolled between 12-<18 weeks gestational age, blood will be collected by phlebotomy at the time of their week 20 routine visit. For participants enrolled between 18-20 weeks gestational age, blood will be collected by finger prick at the time of their week 20 routine visit.

Explanation of maternal schedule of events

1. HIV test will be done at enrollment and documented. A repeat rapid HIV test will be done at 28 weeks gestational age and delivery. HIV testing shall be done using standard rapid HIV-testing algorithm.
2. Ultrasound will be done to confirm intrauterine pregnancy and estimate gestational age at enrollment and to assess fetal growth at 24, 32 and 36 weeks gestational age. See Appendix B for dating criteria.
3. Targeted physical exam will include anthropometric measurements (e.g. weight), mid-upper arm circumference, and vital signs (i.e. temperature, pulse, and blood pressure). Measurement of height at the enrollment visit only.
4. Labor & Delivery documentation will include: Peripartum history, mode of delivery, Apgar scores (when available), weight, length, and head circumference of the child at birth, approximate gestational age, duration of labor, maternal blood pressure during labor, signs

of fetal distress (presence of meconium), summary of events in first days of life (including feeding, breathing patterns, jaundice, lethargy, or any additional abnormal findings), duration of admission if delivered in hospital.

5. Obstetrical exam includes estimation of gestational age at study entry, fundal height measurement, fetal heart tones and urine dipstick for protein. A cervical exam will also be performed at screening and during antepartum study visits as clinically indicated.
6. Mebendazole is typically given as 500mg as a single dose as early as possible after the 1st trimester (16 or 20 week visit).
7. Vitamin A supplementation is dosed as 200,000 IU.

Appendix H. Division of AIDS (DAIDS) Table for Grading the Severity of Adult and Pediatric Adverse Events (Corrected Version 2.1 - July 2017)

Citation:
U.S. Department of Health and Human Services, National Institutes of Health, National Institute of Allergy and Infectious Diseases, Division of AIDS. Division of AIDS (DAIDS) Table for Grading the Severity of Adult and Pediatric Adverse Events, Corrected Version 2.1. [July 2017]. Available from: https://rsc.niaid.nih.gov/sites/default/files/daidsgradingcorrectedv21.pdfpdf

Selected sections of the above document are listed here:

Introduction

The Division of AIDS (DAIDS) oversees more than 300 clinical trials domestically and internationally, which evaluate the safety and efficacy of therapeutic products, vaccines, and other preventive modalities. Adverse event (AE) data collected during these clinical trials form the basis for subsequent safety and efficacy analyses of pharmaceutical products and medical devices. Incorrect and inconsistent AE severity grading can lead to inaccurate data analyses and interpretation, which in turn can impact the safety and well-being of clinical trial participants and future patients using pharmaceutical products.

Over the years, DAIDS scientific knowledge and experience have expanded, necessitating revisions of the DAIDS grading table, which serves as a guide for assessing the severity of AEs (including clinical and laboratory abnormalities) in participants enrolled in DAIDS-sponsored and -supported clinical trials. The Division of AIDS (DAIDS) Table for Grading the Severity of Adult and Pediatric Adverse Events, Corrected Version 2.1 (July 2017) updates and replaces version 2.1 (March 2017).

Instructions for Use

General Considerations
The Division of AIDS (DAIDS) Table for Grading the Severity of Adult and Pediatric Adverse Events, Version 2.1 consists of parameters, or AEs, with severity grading guidance that are to be used in DAIDS clinical trials for safety data reporting to maintain accuracy and consistency in the evaluation of AEs. The term "severe" is not the same as the term "serious" in classifying AEs. The severity of a specific event describes its intensity, and it is the intensity which is graded. Seriousness, which is not graded, relates to an outcome of an AE and is a regulatory definition.

Clinical sites are encouraged to report parameters in the DAIDS grading table as they are written to maintain data consistency across clinical trials. However, since some parameters can be reported with more specificity, clinical sites are encouraged to report parameters that convey additional clinical information. For example, diarrhea could be reported as neonatal diarrhea; seizures, as febrile seizures; and pain, as jaw pain.

The DAIDS grading table provides an AE severity grading scale ranging from grades 1 to 5 with descriptions for each AE based on the following general guidelines:

- Grade 1 indicates a mild event
- Grade 2 indicates a moderate event
- Grade 3 indicates a severe event
- Grade 4 indicates a potentially life-threatening event

- Grade 5 indicates death (Note: This grade is not specifically listed on each page of the grading table).

Other points to consider include:
- Use age and sex values as applicable.
- Unless noted, laboratory values are for term neonates. Preterm neonates should be assessed using local laboratory normal ranges.
- Where applicable, Standard International (SI) units are included in italics.

Selecting and Reporting a Primary AE Term
When selecting a primary AE term to report, sites should select the term that best describes what occurred to the participant. For example, a participant may present with itching, urticaria, flushing, angioedema of the face, and dyspnea. If the underlying diagnosis is determined to be an acute allergic reaction, sites should report “Acute Allergic Reaction” as the primary AE term.

Primary AE terms should be reported using the DAIDS Adverse Experience Reporting System (DAERS) only if they meet expedited reporting criteria. However, all primary AE terms should be reported using protocol-specific case report forms (CRFs). Because the reported information is stored in different databases (i.e., safety and clinical), sites should report primary AE terms using the same terminology for data consistency.

When reporting using DAERS, other clinically significant events associated with a primary AE term that more fully describe the nature, severity, or complications of the primary AE term should be entered in the “Other Events” section. However, the severity grade for these events must be lower than or equal to the severity grade of the primary AE term. In the example above, dyspnea and angioedema of the face may be entered in the “Other Events” section, because they are more descriptive and provide additional information on the severity of the acute allergic reaction. However, their severity grades must be lower than or equal to the severity grade of the primary AE term of “Acute Allergic Reaction”.

Differences exist in the reporting and recording of information (e.g., signs and symptoms, clinically significant events) in DAERS and CRFs. Therefore, sites should refer to their protocols and CRF requirements for further instructions.

Grading Adult and Pediatric AEs
When a single parameter is not appropriate for grading an AE in both adult and pediatric populations, separate parameters with specified age ranges are provided. If no distinction between adult and pediatric populations has been made, the listed parameter should be used for grading an AE in both populations.

Reporting Pregnancy Outcomes
In the Pregnancy, Puerperium, and Perinatal section, all parameters are pregnancy outcomes and should be reported using the mother's participant ID. If an infant is not enrolled in the same study as the mother, any identified birth defects should be reported using the mother's participant ID. However, if an infant is enrolled in the same study as the mother or in another study, any identified birth defects should be reported using the infant's participant ID. Sites should refer to the applicable network standards for reporting abnormal pregnancy outcomes on the CRFs.

Determining Severity Grade for Parameters between Grades

If the severity of an AE could fall in either one of two grades (i.e., the severity of an AE could be either grade 2 or grade 3), sites should select the higher of the two grades.

Laboratory Values
General. An asymptomatic, abnormal laboratory finding without an accompanying AE should not be reported to DAIDS in an expedited timeframe unless it meets protocol-specific reporting requirements. Sites should refer to the applicable network standards for reporting abnormal laboratory findings on the clinical case report forms.

Values below Grade 1. Any laboratory value that is between the ULN and grade 1 (for high values) or the LLN and grade 1 (for low values) should not be graded or reported as an AE. Sites should consult the Manual for Expedited Reporting of Adverse Events to DAIDS, Version 2.0 and their protocol when making an assessment of the need to report an AE.

Overlap of Local Laboratory Normal Values with Grading Table Ranges. When local laboratory normal values fall within grading table laboratory ranges, the severity grading is based on the ranges in the grading table unless there is a protocol-specific grading criterion for the laboratory value. For example, “Magnesium, Low" has a grade 1 range of 1.2 to < 1.4 mEq/L, while a particular laboratory’s normal range for magnesium may be 1.3 to 2.8 mEq/L. If a study participant’s magnesium laboratory value is 1.3 mEq/L, the laboratory value should be graded as grade 1.

Estimating Severity Grade for Parameters Not Identified in the Grading Table
The functional table below should be used to grade the severity of an AE that is not specifically identified in the grading table. In addition, all deaths related to an AE are to be classified as grade 5.

PARAMETER	GRADE 1MILD	GRADE 2 MODERATE	GRADE 3 SEVERE	GRADE 4 POTENTIALLY LIFE-THREATENING
**Clinical** adverse event **NOT** identified elsewhere in the grading table	Mild symptoms causing no or minimal interference with usual social & functional activities with intervention not indicated	Moderate symptoms causing greater than minimal interference with usual social & functional activities with intervention indicated	Severe symptoms causing inability to perform usual social & functional activities with intervention or hospitalization indicated	Potentially life-threatening symptoms causing inability to perform basic self-care functions with intervention indicated to prevent permanent impairment, persistent disability, or death

Major Clinical Conditions

Cardiovascular

PARAMETER	GRADE 1 MILD	GRADE 2 MODERATE	GRADE 3 SEVERE	GRADE 4 POTENTIALLY LIFE-THREATENING
**Arrhythmia**<br>(by ECG or physical examination)<br>*Specify type, if applicable*	No symptoms AND No intervention indicated	No symptoms AND Non-urgent intervention indicated	Non-life-threatening symptoms AND Non-urgent intervention indicated	Life-threatening arrhythmia OR Urgent intervention indicated
**Blood Pressure Abnormalities**[1]<br>*Hypertension (with the lowest reading taken after repeat testing during a visit)*<br>*≥ 18 years of age*	140 to < 160 mmHg systolic<br>OR<br>90 to < 100 mmHg diastolic	≥ 160 to < 180 mmHg systolic<br>OR<br>≥ 100 to < 110 mmHg diastolic	≥ 180 mmHg systolic<br>OR<br>≥ 110 mmHg diastolic	Life-threatening consequences in a participant not previously diagnosed with hypertension (e.g., malignant hypertension) OR Hospitalization indicated
< 18 years of age	> 120/80 mmHg	$\geq 95^{th}$ to $< 99^{th}$ percentile + 5 mmHg adjusted for age, height, and gender (systolic and/or diastolic)	$\geq 99^{th}$ percentile + 5 mmHg adjusted for age, height, and gender (systolic and/or diastolic)	Life-threatening consequences in a participant not previously diagnosed with hypertension (e.g., malignant hypertension) OR Hospitalization indicated
Hypotension	No symptoms	Symptoms corrected with oral fluid replacement	Symptoms AND IV fluids indicated	Shock requiring use of vasopressors or mechanical assistance to maintain blood pressure
**Cardiac Ischemia or Infarction**<br>*Report only one*	NA	NA	New symptoms with ischemia (stable angina) OR New testing consistent with ischemia	Unstable angina OR Acute myocardial infarction
**Heart Failure**	No symptoms AND Laboratory or cardiac imaging abnormalities	Symptoms with mild to moderate activity or exertion	Symptoms at rest or with minimal activity or exertion (e.g., hypoxemia) OR Intervention indicated (e.g., oxygen)	Life-threatening consequences OR Urgent intervention indicated (e.g., vasoactive medications, ventricular assist device, heart transplant)

[1] Blood pressure norms for children < 18 years of age can be found in: Expert Panel on Integrated Guidelines for Cardiovascular Health and Risk Reduction in Children and Adolescents. *Pediatrics* 2011;128;S213; originally published online November 14, 2011; DOI: 10.1542/peds.2009-2107C.

Cardiovascular

PARAMETER	GRADE 1 MILD	GRADE 2 MODERATE	GRADE 3 SEVERE	GRADE 4 POTENTIALLY LIFE-THREATENING
**Hemorrhage** (with significant acute blood loss)	NA	Symptoms AND No transfusion indicated	Symptoms AND Transfusion of ≤ 2 units packed RBCs indicated	Life-threatening hypotension OR Transfusion of > 2 units packed RBCs (for children, packed RBCs > 10 cc/kg) indicated
**Prolonged PR Interval or AV Block** *Report only one* *> 16 years of age*	PR interval 0.21 to < 0.25 seconds	PR interval ≥ 0.25 seconds OR Type I 2nd degree AV block	Type II 2nd degree AV block OR Ventricular pause ≥ 3.0 seconds	Complete AV block
≤ 16 years of age	1st degree AV block (PR interval > normal for age and rate)	Type I 2nd degree AV block	Type II 2nd degree AV block OR Ventricular pause ≥ 3.0 seconds	Complete AV block
**Prolonged QTc Interval**[2]	0.45 to 0.47 seconds	> 0.47 to 0.50 seconds	> 0.50 seconds OR ≥ 0.06 seconds above baseline	Life-threatening consequences (e.g., Torsade de pointes, other associated serious ventricular dysrhythmia)
**Thrombosis or Embolism** *Report only one*	NA	Symptoms AND No intervention indicated	Symptoms AND Intervention indicated	Life-threatening embolic event (e.g., pulmonary embolism, thrombus)

[2] As per Bazett's formula.

Dermatologic

PARAMETER	GRADE 1 MILD	GRADE 2 MODERATE	GRADE 3 SEVERE	GRADE 4 POTENTIALLY LIFE-THREATENING
**Alopecia** (scalp only)	Detectable by study participant, caregiver, or physician AND Causing no or minimal interference with usual social & functional activities	Obvious on visual inspection AND Causing greater than minimal interference with usual social & functional activities	NA	NA
**Bruising**	Localized to one area	Localized to more than one area	Generalized	NA
**Cellulitis**	NA	Non-parenteral treatment indicated (e.g., oral antibiotics, antifungals, antivirals)	IV treatment indicated (e.g., IV antibiotics, antifungals, antivirals)	Life-threatening consequences (e.g., sepsis, tissue necrosis)
**Hyperpigmentation**	Slight or localized causing no or minimal interference with usual social & functional activities	Marked or generalized causing greater than minimal interference with usual social & functional activities	NA	NA
**Hypopigmentation**	Slight or localized causing no or minimal interference with usual social & functional activities	Marked or generalized causing greater than minimal interference with usual social & functional activities	NA	NA
**Petechiae**	Localized to one area	Localized to more than one area	Generalized	NA
**Pruritus**[3] (without skin lesions)	Itching causing no or minimal interference with usual social & functional activities	Itching causing greater than minimal interference with usual social & functional activities	Itching causing inability to perform usual social & functional activities	NA
**Rash** *Specify type, if applicable*	Localized rash	Diffuse rash OR Target lesions	Diffuse rash AND Vesicles or limited number of bullae or superficial ulcerations of mucous membrane limited to one site	Extensive or generalized bullous lesions OR Ulceration of mucous membrane involving two or more distinct mucosal sites OR Stevens-Johnson syndrome OR Toxic epidermal necrolysis

[3] For pruritus associated with injections or infusions, see the *Site Reactions to Injections and Infusions* section (page 23).

Endocrine and Metabolic

PARAMETER	GRADE 1 MILD	GRADE 2 MODERATE	GRADE 3 SEVERE	GRADE 4 POTENTIALLY LIFE-THREATENING
**Diabetes Mellitus**	Controlled without medication	Controlled with medication OR Modification of current medication regimen	Uncontrolled despite treatment modification OR Hospitalization for immediate glucose control indicated	Life-threatening consequences (e.g., ketoacidosis, hyperosmolar non-ketotic coma, end organ failure)
**Gynecomastia**	Detectable by study participant, caregiver, or physician AND Causing no or minimal interference with usual social & functional activities	Obvious on visual inspection AND Causing pain with greater than minimal interference with usual social & functional activities	Disfiguring changes AND Symptoms requiring intervention or causing inability to perform usual social & functional activities	NA
**Hyperthyroidism**	No symptoms AND Abnormal laboratory value	Symptoms causing greater than minimal interference with usual social & functional activities OR Thyroid suppression therapy indicated	Symptoms causing inability to perform usual social & functional activities OR Uncontrolled despite treatment modification	Life-threatening consequences (e.g., thyroid storm)
**Hypothyroidism**	No symptoms AND Abnormal laboratory value	Symptoms causing greater than minimal interference with usual social & functional activities OR Thyroid replacement therapy indicated	Symptoms causing inability to perform usual social & functional activities OR Uncontrolled despite treatment modification	Life-threatening consequences (e.g., myxedema coma)
**Lipoatrophy**[4]	Detectable by study participant, caregiver, or physician AND Causing no or minimal interference with usual social & functional activities	Obvious on visual inspection AND Causing greater than minimal interference with usual social & functional activities	Disfiguring changes	NA

[4] Definition: A disorder characterized by fat loss in the face, extremities, and buttocks.

Endocrine and Metabolic

PARAMETER	GRADE 1 MILD	GRADE 2 MODERATE	GRADE 3 SEVERE	GRADE 4 POTENTIALLY LIFE-THREATENING
**Lipohypertrophy**[5]	Detectable by study participant, caregiver, or physician AND Causing no or minimal interference with usual social & functional activities	Obvious on visual inspection AND Causing greater than minimal interference with usual social & functional activities	Disfiguring changes	NA

[5] Definition: A disorder characterized by abnormal fat accumulation on the back of the neck, breasts, and abdomen.

Gastrointestinal

PARAMETER	GRADE 1 MILD	GRADE 2 MODERATE	GRADE 3 SEVERE	GRADE 4 POTENTIALLY LIFE-THREATENING
**Anorexia**	Loss of appetite without decreased oral intake	Loss of appetite associated with decreased oral intake without significant weight loss	Loss of appetite associated with significant weight loss	Life-threatening consequences OR Aggressive intervention indicated (e.g., tube feeding, total parenteral nutrition)
**Ascites**	No symptoms	Symptoms AND Intervention indicated (e.g., diuretics, therapeutic paracentesis)	Symptoms recur or persist despite intervention	Life-threatening consequences
**Bloating or Distension** *Report only one*	Symptoms causing no or minimal interference with usual social & functional activities	Symptoms causing greater than minimal interference with usual social & functional activities	Symptoms causing inability to perform usual social & functional activities	NA
**Cholecystitis**	NA	Symptoms AND Medical intervention indicated	Radiologic, endoscopic, or operative intervention indicated	Life-threatening consequences (e.g., sepsis, perforation)
**Constipation**	NA	Persistent constipation requiring regular use of dietary modifications, laxatives, or enemas	Obstipation with manual evacuation indicated	Life-threatening consequences (e.g., obstruction)
**Diarrhea** *≥ 1 year of age*	Transient or intermittent episodes of unformed stools OR Increase of ≤ 3 stools over baseline per 24-hour period	Persistent episodes of unformed to watery stools OR Increase of 4 to 6 stools over baseline per 24-hour period	Increase of ≥ 7 stools per 24-hour period OR IV fluid replacement indicated	Life-threatening consequences (e.g., hypotensive shock)
< 1 year of age	Liquid stools (more unformed than usual) but usual number of stools	Liquid stools with increased number of stools OR Mild dehydration	Liquid stools with moderate dehydration	Life-threatening consequences (e.g., liquid stools resulting in severe dehydration, hypotensive shock)
**Dysphagia or Odynophagia** *Report only one and specify location*	Symptoms but able to eat usual diet	Symptoms causing altered dietary intake with no intervention indicated	Symptoms causing severely altered dietary intake with intervention indicated	Life-threatening reduction in oral intake
**Gastrointestinal Bleeding**	Not requiring intervention other than iron supplement	Endoscopic intervention indicated	Transfusion indicated	Life-threatening consequences (e.g., hypotensive shock)

Gastrointestinal

PARAMETER	GRADE 1 MILD	GRADE 2 MODERATE	GRADE 3 SEVERE	GRADE 4 POTENTIALLY LIFE-THREATENING
**Mucositis or Stomatitis** *Report only one and specify location*	Mucosal erythema	Patchy pseudomembranes or ulcerations	Confluent pseudomembranes or ulcerations OR Mucosal bleeding with minor trauma	Life-threatening consequences (e.g., aspiration, choking) OR Tissue necrosis OR Diffuse spontaneous mucosal bleeding
**Nausea**	Transient (< 24 hours) or intermittent AND No or minimal interference with oral intake	Persistent nausea resulting in decreased oral intake for 24 to 48 hours	Persistent nausea resulting in minimal oral intake for > 48 hours OR Rehydration indicated (e.g., IV fluids)	Life-threatening consequences (e.g., hypotensive shock)
**Pancreatitis**	NA	Symptoms with hospitalization not indicated	Symptoms with hospitalization indicated	Life-threatening consequences (e.g., circulatory failure, hemorrhage, sepsis)
**Perforation** (colon or rectum)	NA	NA	Intervention indicated	Life-threatening consequences
**Proctitis**	Rectal discomfort with no intervention indicated	Symptoms causing greater than minimal interference with usual social & functional activities OR Medical intervention indicated	Symptoms causing inability to perform usual social & functional activities OR Operative intervention indicated	Life-threatening consequences (e.g., perforation)
**Rectal Discharge**	Visible discharge	Discharge requiring the use of pads	NA	NA
**Vomiting**	Transient or intermittent AND No or minimal interference with oral intake	Frequent episodes with no or mild dehydration	Persistent vomiting resulting in orthostatic hypotension OR Aggressive rehydration indicated (e.g., IV fluids)	Life-threatening consequences (e.g., hypotensive shock)

Musculoskeletal

PARAMETER	GRADE 1 MILD	GRADE 2 MODERATE	GRADE 3 SEVERE	GRADE 4 POTENTIALLY LIFE-THREATENING
**Arthralgia**	Joint pain causing no or minimal interference with usual social & functional activities	Joint pain causing greater than minimal interference with usual social & functional activities	Joint pain causing inability to perform usual social & functional activities	Disabling joint pain causing inability to perform basic self-care functions
**Arthritis**	Stiffness or joint swelling causing no or minimal interference with usual social & functional activities	Stiffness or joint swelling causing greater than minimal interference with usual social & functional activities	Stiffness or joint swelling causing inability to perform usual social & functional activities	Disabling joint stiffness or swelling causing inability to perform basic self-care functions
**Myalgia** (generalized)	Muscle pain causing no or minimal interference with usual social & functional activities	Muscle pain causing greater than minimal interference with usual social & functional activities	Muscle pain causing inability to perform usual social & functional activities	Disabling muscle pain causing inability to perform basic self-care functions
**Osteonecrosis**	NA	No symptoms but with radiographic findings AND No operative intervention indicated	Bone pain with radiographic findings OR Operative intervention indicated	Disabling bone pain with radiographic findings causing inability to perform basic self-care functions
**Osteopenia**[6] *≥ 30 years of age*	BMD t-score -2.5 to -1	NA	NA	NA
< 30 years of age	BMD z-score -2 to -1	NA	NA	NA
**Osteoporosis**[6] *≥ 30 years of age*	NA	BMD t-score < -2.5	Pathologic fracture (e.g., compression fracture causing loss of vertebral height)	Pathologic fracture causing life-threatening consequences
< 30 years of age	NA	BMD z-score < -2	Pathologic fracture (e.g., compression fracture causing loss of vertebral height)	Pathologic fracture causing life-threatening consequences

[6] BMD t and z scores can be found in: Kanis JA on behalf of the World Health Organization Scientific Group (2007). Assessment of osteoporosis at the primary health-care level. Technical Report. World Health Organization Collaborating Centre for Metabolic Bone Diseases, University of Sheffield, UK. 2007: Printed by the University of Sheffield.

Neurologic

PARAMETER	GRADE 1 MILD	GRADE 2 MODERATE	GRADE 3 SEVERE	GRADE 4 POTENTIALLY LIFE-THREATENING
**Acute CNS Ischemia**	NA	NA	Transient ischemic attack	Cerebral vascular accident (e.g., stroke with neurological deficit)
**Altered Mental Status** (for Dementia, see *Cognitive, Behavioral, or Attentional Disturbance* below)	Changes causing no or minimal interference with usual social & functional activities	Mild lethargy or somnolence causing greater than minimal interference with usual social & functional activities	Confusion, memory impairment, lethargy, or somnolence causing inability to perform usual social & functional activities	Delirium OR Obtundation OR Coma
**Ataxia**	Symptoms causing no or minimal interference with usual social & functional activities OR No symptoms with ataxia detected on examination	Symptoms causing greater than minimal interference with usual social & functional activities	Symptoms causing inability to perform usual social & functional activities	Disabling symptoms causing inability to perform basic self-care functions
**Cognitive, Behavioral, or Attentional Disturbance** (includes dementia and attention deficit disorder) *Specify type, if applicable*	Disability causing no or minimal interference with usual social & functional activities OR Specialized resources not indicated	Disability causing greater than minimal interference with usual social & functional activities OR Specialized resources on part-time basis indicated	Disability causing inability to perform usual social & functional activities OR Specialized resources on a full-time basis indicated	Disability causing inability to perform basic self-care functions OR Institutionalization indicated
**Developmental Delay** *< 18 years of age* *Specify type, if applicable*	Mild developmental delay, either motor or cognitive, as determined by comparison with a developmental screening tool appropriate for the setting	Moderate developmental delay, either motor or cognitive, as determined by comparison with a developmental screening tool appropriate for the setting	Severe developmental delay, either motor or cognitive, as determined by comparison with a developmental screening tool appropriate for the setting	Developmental regression, either motor or cognitive, as determined by comparison with a developmental screening tool appropriate for the setting
**Headache**	Symptoms causing no or minimal interference with usual social & functional activities	Symptoms causing greater than minimal interference with usual social & functional activities	Symptoms causing inability to perform usual social & functional activities	Symptoms causing inability to perform basic self-care functions OR Hospitalization indicated OR Headache with significant impairment of alertness or other neurologic function

Neurologic

PARAMETER	GRADE 1 MILD	GRADE 2 MODERATE	GRADE 3 SEVERE	GRADE 4 POTENTIALLY LIFE-THREATENING
**Neuromuscular Weakness** (includes myopathy and neuropathy) *Specify type, if applicable*	Minimal muscle weakness causing no or minimal interference with usual social & functional activities OR No symptoms with decreased strength on examination	Muscle weakness causing greater than minimal interference with usual social & functional activities	Muscle weakness causing inability to perform usual social & functional activities	Disabling muscle weakness causing inability to perform basic self-care functions OR Respiratory muscle weakness impairing ventilation
**Neurosensory Alteration** (includes paresthesia and painful neuropathy) *Specify type, if applicable*	Minimal paresthesia causing no or minimal interference with usual social & functional activities OR No symptoms with sensory alteration on examination	Sensory alteration or paresthesia causing greater than minimal interference with usual social & functional activities	Sensory alteration or paresthesia causing inability to perform usual social & functional activities	Disabling sensory alteration or paresthesia causing inability to perform basic self-care functions
**Seizures** *New Onset Seizure* *≥ 18 years of age*	NA	NA	1 to 3 seizures	Prolonged and repetitive seizures (e.g., status epilepticus) OR Difficult to control (e.g., refractory epilepsy)
< 18 years of age (includes new or pre-existing febrile seizures)	Seizure lasting < 5 minutes with < 24 hours postictal state	Seizure lasting 5 to < 20 minutes with < 24 hours postictal state	Seizure lasting ≥ 20 minutes OR > 24 hours postictal state	Prolonged and repetitive seizures (e.g., status epilepticus) OR Difficult to control (e.g., refractory epilepsy)
Pre-existing Seizure	NA	Increased frequency from previous level of control without change in seizure character	Change in seizure character either in duration or quality (e.g., severity or focality)	Prolonged and repetitive seizures (e.g., status epilepticus) OR Difficult to control (e.g., refractory epilepsy)
**Syncope**	Near syncope without loss of consciousness (e.g., pre-syncope)	Loss of consciousness with no intervention indicated	Loss of consciousness AND Hospitalization or intervention required	NA

Pregnancy, Puerperium, and Perinatal

PARAMETER	GRADE 1 MILD	GRADE 2 MODERATE	GRADE 3 SEVERE	GRADE 4 POTENTIALLY LIFE-THREATENING
**Stillbirth** (report using mother's participant ID)<br>*Report only one*	NA	NA	Fetal death occurring at $\geq$ 20 weeks gestation	NA
**Preterm Birth** (report using mother's participant ID)	Live birth at 34 to < 37 weeks gestational age	Live birth at 28 to < 34 weeks gestational age	Live birth at 24 to < 28 weeks gestational age	Live birth at < 24 weeks gestational age
**Spontaneous Abortion or Miscarriage**[7] (report using mother's participant ID)<br>*Report only one*	Chemical pregnancy	Uncomplicated spontaneous abortion or miscarriage	Complicated spontaneous abortion or miscarriage	NA

[7] Definition: A pregnancy loss occurring at < 20 weeks gestational age.

Psychiatric

PARAMETER	GRADE 1 MILD	GRADE 2 MODERATE	GRADE 3 SEVERE	GRADE 4 POTENTIALLY LIFE-THREATENING
**Insomnia**	Mild difficulty falling asleep, staying asleep, or waking up early causing no or minimal interference with usual social & functional activities	Moderate difficulty falling asleep, staying asleep, or waking up early causing more than minimal interference with usual social & functional activities	Severe difficulty falling asleep, staying asleep, or waking up early causing inability to perform usual social & functional activities requiring intervention or hospitalization	NA
**Psychiatric Disorders** (includes anxiety, depression, mania, and psychosis) *Specify disorder*	Symptoms with intervention not indicated OR Behavior causing no or minimal interference with usual social & functional activities	Symptoms with intervention indicated OR Behavior causing greater than minimal interference with usual social & functional activities	Symptoms with hospitalization indicated OR Behavior causing inability to perform usual social & functional activities	Threatens harm to self or others OR Acute psychosis OR Behavior causing inability to perform basic self-care functions
**Suicidal Ideation or Attempt** *Report only one*	Preoccupied with thoughts of death AND No wish to kill oneself	Preoccupied with thoughts of death AND Wish to kill oneself with no specific plan or intent	Thoughts of killing oneself with partial or complete plans but no attempt to do so OR Hospitalization indicated	Suicide attempted

Respiratory

PARAMETER	GRADE 1 MILD	GRADE 2 MODERATE	GRADE 3 SEVERE	GRADE 4 POTENTIALLY LIFE-THREATENING
**Acute Bronchospasm**	Forced expiratory volume in 1 second or peak flow reduced to ≥ 70 to < 80% <u>OR</u> Mild symptoms with intervention not indicated	Forced expiratory volume in 1 second or peak flow 50 to < 70% <u>OR</u> Symptoms with intervention indicated <u>OR</u> Symptoms causing greater than minimal interference with usual social & functional activities	Forced expiratory volume in 1 second or peak flow 25 to < 50% <u>OR</u> Symptoms causing inability to perform usual social & functional activities	Forced expiratory volume in 1 second or peak flow < 25% <u>OR</u> Life-threatening respiratory or hemodynamic compromise <u>OR</u> Intubation
**Dyspnea or Respiratory Distress** *Report only one*	Dyspnea on exertion with no or minimal interference with usual social & functional activities <u>OR</u> Wheezing <u>OR</u> Minimal increase in respiratory rate for age	Dyspnea on exertion causing greater than minimal interference with usual social & functional activities <u>OR</u> Nasal flaring <u>OR</u> Intercostal retractions <u>OR</u> Pulse oximetry 90 to < 95%	Dyspnea at rest causing inability to perform usual social & functional activities <u>OR</u> Pulse oximetry < 90%	Respiratory failure with ventilator support indicated (e.g., CPAP, BPAP, intubation)

Sensory

PARAMETER	GRADE 1 MILD	GRADE 2 MODERATE	GRADE 3 SEVERE	GRADE 4 POTENTIALLY LIFE-THREATENING
**Hearing Loss** *≥ 12 years of age*	NA	Hearing aid or intervention not indicated	Hearing aid or intervention indicated	Profound bilateral hearing loss (> 80 dB at 2 kHz and above) OR Non-serviceable hearing (i.e., >50 dB audiogram and <50% speech discrimination)
< 12 years of age (based on a 1, 2, 3, 4, 6 and 8 kHz audiogram)	> 20 dB hearing loss at ≤ 4 kHz	> 20 dB hearing loss at > 4 kHz	> 20 dB hearing loss at ≥ 3 kHz in one ear with additional speech language related services indicated (where available) OR Hearing loss sufficient to indicate therapeutic intervention, including hearing aids	Audiologic indication for cochlear implant and additional speech-language related services indicated (where available)
**Tinnitus**	Symptoms causing no or minimal interference with usual social & functional activities with intervention not indicated	Symptoms causing greater than minimal interference with usual social & functional activities with intervention indicated	Symptoms causing inability to perform usual social & functional activities	NA
**Uveitis**	No symptoms AND Detectable on examination	Anterior uveitis with symptoms OR Medical intervention indicated	Posterior or pan-uveitis OR Operative intervention indicated	Disabling visual loss in affected eye(s)
**Vertigo**	Vertigo causing no or minimal interference with usual social & functional activities	Vertigo causing greater than minimal interference with usual social & functional activities	Vertigo causing inability to perform usual social & functional activities	Disabling vertigo causing inability to perform basic self-care functions
**Visual Changes** (assessed from baseline)	Visual changes causing no or minimal interference with usual social & functional activities	Visual changes causing greater than minimal interference with usual social & functional activities	Visual changes causing inability to perform usual social & functional activities	Disabling visual loss in affected eye(s)

Systemic

PARAMETER	GRADE 1 MILD	GRADE 2 MODERATE	GRADE 3 SEVERE	GRADE 4 POTENTIALLY LIFE-THREATENING
**Acute Allergic Reaction**	Localized urticaria (wheals) with no medical intervention indicated	Localized urticaria with intervention indicated <u>OR</u> Mild angioedema with no intervention indicated	Generalized urticaria <u>OR</u> Angioedema with intervention indicated <u>OR</u> Symptoms of mild bronchospasm	Acute anaphylaxis <u>OR</u> Life-threatening bronchospasm <u>OR</u> Laryngeal edema
**Chills**	Symptoms causing no or minimal interference with usual social & functional activities	Symptoms causing greater than minimal interference with usual social & functional activities	Symptoms causing inability to perform usual social & functional activities	NA
**Cytokine Release Syndrome**[8]	Mild signs and symptoms <u>AND</u> Therapy (i.e., antibody infusion) interruption not indicated	Therapy (i.e., antibody infusion) interruption indicated <u>AND</u> Responds promptly to symptomatic treatment <u>OR</u> Prophylactic medications indicated for ≤ 24 hours	Prolonged severe signs and symptoms <u>OR</u> Recurrence of symptoms following initial improvement	Life-threatening consequences (e.g., requiring pressor or ventilator support)
**Fatigue or Malaise** *Report only one*	Symptoms causing no or minimal interference with usual social & functional activities	Symptoms causing greater than minimal interference with usual social & functional activities	Symptoms causing inability to perform usual social & functional activities	Incapacitating symptoms of fatigue or malaise causing inability to perform basic self-care functions
**Fever** (non-axillary temperatures only)	38.0 to < 38.6°C or 100.4 to < 101.5°F	≥ 38.6 to < 39.3°C or ≥ 101.5 to < 102.7°F	≥ 39.3 to < 40.0°C or ≥ 102.7 to < 104.0°F	≥ 40.0°C or ≥ 104.0°F
**Pain**[9] (not associated with study agent injections and not specified elsewhere) *Specify location*	Pain causing no or minimal interference with usual social & functional activities	Pain causing greater than minimal interference with usual social & functional activities	Pain causing inability to perform usual social & functional activities	Disabling pain causing inability to perform basic self-care functions <u>OR</u> Hospitalization indicated

[8] Definition: A disorder characterized by nausea, headache, tachycardia, hypotension, rash, and/or shortness of breath.
[9] For pain associated with injections or infusions, see the *Site Reactions to Injections and Infusions* section (page 23).

Systemic

PARAMETER	GRADE 1 MILD	GRADE 2 MODERATE	GRADE 3 SEVERE	GRADE 4 POTENTIALLY LIFE-THREATENING
**Serum Sickness**[10]	Mild signs and symptoms	Moderate signs and symptoms AND Intervention indicated (e.g., antihistamines)	Severe signs and symptoms AND Higher level intervention indicated (e.g., steroids or IV fluids)	Life-threatening consequences (e.g., requiring pressor or ventilator support)
**Underweight**[11] *> 5 to 19 years of age*	WHO BMI z-score < -1 to -2	WHO BMI z-score < -2 to -3	WHO BMI z-score < -3	WHO BMI z-score < -3 with life-threatening consequences
2 to 5 years of age	WHO Weight-for-height z-score < -1 to -2	WHO Weight-for-height z-score < -2 to -3	WHO Weight-for-height z-score < -3	WHO Weight-for-height z-score < -3 with life-threatening consequences
< 2 years of age	WHO Weight-for-length z-score < -1 to -2	WHO Weight-for-length z-score < -2 to -3	WHO Weight-for-length z-score < -3	WHO Weight-for-length z-score < -3 with life-threatening consequences
**Unintentional Weight Loss** (excludes postpartum weight loss)	NA	5 to < 9% loss in body weight from baseline	≥ 9 to < 20% loss in body weight from baseline	≥ 20% loss in body weight from baseline OR Aggressive intervention indicated (e.g., tube feeding, total parenteral nutrition)

[10] Definition: A disorder characterized by fever, arthralgia, myalgia, skin eruptions, lymphadenopathy, marked discomfort, and/or dyspnea.
[11] WHO reference tables may be accessed by clicking the desired age range or by accessing the following URLs: http://www.who.int/growthref/who2007_bmi_for_age/en/ for participants > 5 to 19 years of age and http://www.who.int/childgrowth/standards/chart_catalogue/en/ for those ≤ 5 years of age.

Urinary

PARAMETER	GRADE 1 MILD	GRADE 2 MODERATE	GRADE 3 SEVERE	GRADE 4 POTENTIALLY LIFE-THREATENING
**Urinary Tract Obstruction**	NA	Signs or symptoms of urinary tract obstruction without hydronephrosis or renal dysfunction	Signs or symptoms of urinary tract obstruction with hydronephrosis or renal dysfunction	Obstruction causing life-threatening consequences

Site Reactions to Injections and Infusions

PARAMETER	GRADE 1 MILD	GRADE 2 MODERATE	GRADE 3 SEVERE	GRADE 4 POTENTIALLY LIFE-THREATENING
**Injection Site Pain or Tenderness** *Report only one*	Pain or tenderness causing no or minimal limitation of use of limb	Pain or tenderness causing greater than minimal limitation of use of limb	Pain or tenderness causing inability to perform usual social & functional activities	Pain or tenderness causing inability to perform basic self-care function OR Hospitalization indicated
**Injection Site Erythema or Redness**[12] *Report only one* *> 15 years of age*	2.5 to < 5 cm in diameter OR 6.25 to < 25 cm² surface area AND Symptoms causing no or minimal interference with usual social & functional activities	≥ 5 to < 10 cm in diameter OR ≥ 25 to < 100 cm² surface area OR Symptoms causing greater than minimal interference with usual social & functional activities	≥ 10 cm in diameter OR ≥ 100 cm² surface area OR Ulceration OR Secondary infection OR Phlebitis OR Sterile abscess OR Drainage OR Symptoms causing inability to perform usual social & functional activities	Potentially life-threatening consequences (e.g., abscess, exfoliative dermatitis, necrosis involving dermis or deeper tissue)
≤ 15 years of age	≤ 2.5 cm in diameter	> 2.5 cm in diameter with < 50% surface area of the extremity segment involved (e.g., upper arm or thigh)	≥ 50% surface area of the extremity segment involved (e.g., upper arm or thigh) OR Ulceration OR Secondary infection OR Phlebitis OR Sterile abscess OR Drainage	Potentially life-threatening consequences (e.g., abscess, exfoliative dermatitis, necrosis involving dermis or deeper tissue)
**Injection Site Induration or Swelling** *Report only one* *> 15 years of age*	Same as for **Injection Site Erythema or Redness**, *> 15 years of age*	Same as for **Injection Site Erythema or Redness**, *> 15 years of age*	Same as for **Injection Site Erythema or Redness**, *> 15 years of age*	Same as for **Injection Site Erythema or Redness**, *> 15 years of age*
≤ 15 years of age	Same as for **Injection Site Erythema or Redness**, *≤ 15 years of age*	Same as for **Injection Site Erythema or Redness**, *≤ 15 years of age*	Same as for **Injection Site Erythema or Redness**, *≤ 15 years of age*	Same as for **Injection Site Erythema or Redness**, *≤ 15 years of age*
**Injection Site Pruritus**	Itching localized to the injection site that is relieved spontaneously or in < 48 hours of treatment	Itching beyond the injection site that is not generalized OR Itching localized to the injection site requiring ≥ 48 hours treatment	Generalized itching causing inability to perform usual social & functional activities	NA

[12] Injection Site Erythema or Redness should be evaluated and graded using the greatest single diameter or measured surface area.

*Laboratory Values**
Chemistries

PARAMETER	GRADE 1<br>MILD	GRADE 2<br>MODERATE	GRADE 3<br>SEVERE	GRADE 4<br>POTENTIALLY LIFE-THREATENING
**Acidosis**	NA	pH ≥ 7.3 to < LLN	pH < 7.3 without life-threatening consequences	pH < 7.3 with life-threatening consequences
**Albumin, Low**<br>(g/dL; *g/L*)	3.0 to < LLN<br>*30 to < LLN*	≥ 2.0 to < 3.0<br>*≥ 20 to < 30*	< 2.0<br>*< 20*	NA
**Alkaline Phosphatase, High**	1.25 to < 2.5 x ULN	2.5 to < 5.0 x ULN	5.0 to < 10.0 x ULN	≥ 10.0 x ULN
**Alkalosis**	NA	pH > ULN to ≤ 7.5	pH > 7.5 without life-threatening consequences	pH > 7.5 with life-threatening consequences
**ALT or SGPT, High**<br>*Report only one*	1.25 to < 2.5 x ULN	2.5 to < 5.0 x ULN	5.0 to < 10.0 x ULN	≥ 10.0 x ULN
**Amylase (Pancreatic) or Amylase (Total), High**<br>*Report only one*	1.1 to < 1.5 x ULN	1.5 to < 3.0 x ULN	3.0 to < 5.0 x ULN	≥ 5.0 x ULN
**AST or SGOT, High**<br>*Report only one*	1.25 to < 2.5 x ULN	2.5 to < 5.0 x ULN	5.0 to < 10.0 x ULN	≥ 10.0 x ULN
**Bicarbonate, Low**<br>(mEq/L; *mmol/L*)	16.0 to < LLN<br>*16.0 to < LLN*	11.0 to < 16.0<br>*11.0 to < 16.0*	8.0 to < 11.0<br>*8.0 to < 11.0*	< 8.0<br>*< 8.0*
**Bilirubin**<br>***Direct Bilirubin*[13]*, High***<br>*> 28 days of age*	NA	NA	> ULN with other signs and symptoms of hepatotoxicity.	> ULN with life-threatening consequences (e.g., signs and symptoms of liver failure)
≤ 28 days of age	ULN to ≤ 1 mg/dL	> 1 to ≤ 1.5 mg/dL	> 1.5 to ≤ 2 mg/dL	> 2 mg/dL
Total Bilirubin, High<br>*> 28 days of age*	1.1 to < 1.6 x ULN	1.6 to < 2.6 x ULN	2.6 to < 5.0 x ULN	≥ 5.0 x ULN
≤ 28 days of age	See Appendix A. Total Bilirubin for Term and Preterm Neonates	See Appendix A. Total Bilirubin for Term and Preterm Neonates	See Appendix A. Total Bilirubin for Term and Preterm Neonates	See Appendix A. Total Bilirubin for Term and Preterm Neonates

*Reminder: An asymptomatic abnormal laboratory finding without an accompanying AE should not be reported to DAIDS in an expedited time frame unless it meets protocol-specific reporting requirements.

[13] Direct bilirubin > 1.5 mg/dL in a participant < 28 days of age should be graded as grade 2, if < 10% of the total bilirubin.

Chemistries

PARAMETER	GRADE 1 MILD	GRADE 2 MODERATE	GRADE 3 SEVERE	GRADE 4 POTENTIALLY LIFE-THREATENING
**Calcium, High**<br>(mg/dL; *mmol/L*)<br>*≥ 7 days of age*	10.6 to < 11.5<br>*2.65* to < *2.88*	11.5 to < 12.5<br>*2.88* to < *3.13*	12.5 to < 13.5<br>*3.13* to < *3.38*	≥ 13.5<br>≥ *3.38*
< 7 days of age	11.5 to < 12.4<br>*2.88* to < *3.10*	12.4 to < 12.9<br>*3.10* to < *3.23*	12.9 to < 13.5<br>*3.23* to < *3.38*	≥ 13.5<br>≥ *3.38*
**Calcium (Ionized), High**<br>(mg/dL; *mmol/L*)	> ULN to < 6.0<br>*> ULN to < 1.5*	6.0 to < 6.4<br>*1.5 to < 1.6*	6.4 to < 7.2<br>*1.6 to < 1.8*	≥ 7.2<br>≥ *1.8*
**Calcium, Low**<br>(mg/dL; *mmol/L*)<br>*≥ 7 days of age*	7.8 to < 8.4<br>*1.95* to < *2.10*	7.0 to < 7.8<br>*1.75* to < *1.95*	6.1 to < 7.0<br>*1.53* to < *1.75*	< 6.1<br>< *1.53*
< 7 days of age	6.5 to < 7.5<br>*1.63* to < *1.88*	6.0 to < 6.5<br>*1.50* to < *1.63*	5.50 to < 6.0<br>*1.38* to < *1.50*	< 5.50<br>< *1.38*
**Calcium (Ionized), Low**<br>(mg/dL; *mmol/L*)	< LLN to 4.0<br>*< LLN to 1.0*	3.6 to < 4.0<br>*0.9 to < 1.0*	3.2 to < 3.6<br>*0.8 to < 0.9*	< 3.2<br>< *0.8*
**Cardiac Troponin I, High**	NA	NA	NA	Levels consistent with myocardial infarction or unstable angina as defined by the local laboratory
**Creatine Kinase, High**	3 to < 6 x ULN	6 to < 10x ULN	10 to < 20 x ULN	≥ 20 x ULN
**Creatinine, High**<br>**Report only one*	1.1 to 1.3 x ULN	> 1.3 to 1.8 x ULN OR Increase to 1.3 to < 1.5 x participant's baseline	> 1.8 to < 3.5 x ULN OR Increase to 1.5 to < 2.0 x participant's baseline	≥ 3.5 x ULN OR Increase of > 2.0 x participant's baseline
**Creatinine Clearance[14] or eGFR, Low**<br>**Report only one*	NA	< 90 to 60 ml/min or ml/min/1.73 m^2<br>OR<br>10 to < 30% decrease from participant's baseline	< 60 to 30 ml/min or ml/min/1.73 m^2<br>OR<br>30 to < 50% decrease from participant's baseline	< 30 ml/min or ml/min/1.73 m^2<br>OR<br>> 50% decrease from participant's baseline or dialysis needed
**Glucose**<br>(mg/dL; *mmol/L*)<br>***Fasting, High***	110 to 125<br>*6.11 to < 6.95*	> 125 to 250<br>*6.95 to < 13.89*	> 250 to 500<br>*13.89 to < 27.75*	≥ 500<br>≥ *27.75*
Nonfasting, High	116 to 160<br>*6.44 to < 8.89*	> 160 to 250<br>*8.89 to < 13.89*	> 250 to 500<br>*13.89 to < 27.75*	≥ 500<br>≥ *27.75*

[14] Use the applicable formula (i.e., Cockcroft-Gault in mL/min or Schwartz, MDRD, CKD-Epi in mL/min/1.73m2). Sites should choose the method defined in their study and when not specified, use the method most relevant to the study population.

*Reminder: Choose the method that selects for the higher grade.

Chemistries

PARAMETER	GRADE 1 MILD	GRADE 2 MODERATE	GRADE 3 SEVERE	GRADE 4 POTENTIALLY LIFE-THREATENING
**Glucose, Low** (mg/dL; *mmol/L*) *≥ 1 month of age*	55 to 64 *3.05 to <3.55*	40 to < 55 *2.22 to < 3.05*	30 to < 40 *1.67 to < 2.22*	< 30 *< 1.67*
< 1 month of age	50 to 54 *2.78 to < 3.00*	40 to < 50 *2.22 to < 2.78*	30 to < 40 *1.67 to < 2.22*	< 30 *< 1.67*
**Lactate, High**	ULN to < 2.0 x ULN without acidosis	≥ 2.0 x ULN without acidosis	Increased lactate with pH < 7.3 without life-threatening consequences	Increased lactate with pH < 7.3 with life-threatening consequences
**Lipase, High**	1.1 to < 1.5 x ULN	1.5 to < 3.0 x ULN	3.0 to < 5.0 x ULN	≥ 5.0 x ULN
**Lipid Disorders** (mg/doll; *mmol/L*) ***Cholesterol, Fasting, High*** *≥ 18 years of age*	200 to < 240 *5.18 to < 6.19*	240 to < 300 *6.19 to < 7.77*	≥ 300 *≥ 7.77*	NA
< 18 years of age	170 to < 200 *4.40 to < 5.15*	200 to < 300 *5.15 to < 7.77*	≥ 300 *≥ 7.77*	NA
LDL, Fasting, High *≥ 18 years of age*	130 to < 160 *3.37 to < 4.12*	160 to < 190 *4.12 to < 4.90*	≥ 190 *≥ 4.90*	NA
> 2 to < 18 years of age	110 to < 130 *2.85 to < 3.34*	130 to < 190 *3.34 to < 4.90*	≥ 190 *≥ 4.90*	NA
Triglycerides, Fasting, High	150 to 300 *1.71 to 3.42*	>300 to 500 *>3.42 to 5.7*	>500 to < 1,000 *>5.7 to 11.4*	> 1,000 *> 11.4*
**Magnesium[15], Low** (mEq/L; *mmol/L*)	1.2 to < 1.4 *0.60 to < 0.70*	0.9 to < 1.2 *0.45 to < 0.60*	0.6 to < 0.9 *0.30 to < 0.45*	< 0.6 *< 0.30*
**Phosphate, Low** (mg/dL; *mmol/L*) *> 14 years of age*	2.0 to < LLN *0.65 to < LLN*	1.4 to < 2.0 *0.45 to < 0.65*	1.0 to < 1.4 *0.32 to < 0.45*	< 1.0 *< 0.32*
1 to 14 years of age	3.0 to < 3.5 *0.97 to < 1.13*	2.5 to < 3.0 *0.81 to < 0.97*	1.5 to < 2.5 *0.48 to < 0.81*	< 1.5 *< 0.48*
< 1 year of age	3.5 to < 4.5 *1.13 to < 1.45*	2.5 to < 3.5 *0.81 to < 1.13*	1.5 to < 2.5 *0.48 to < 0.81*	< 1.5 *< 0.48*
**Potassium, High** (mEq/L; *mmol/L*)	5.6 to < 6.0 *5.6 to < 6.0*	6.0 to < 6.5 *6.0 to < 6.5*	6.5 to < 7.0 *6.5 to < 7.0*	≥ 7.0 *≥ 7.0*
**Potassium, Low** (mEq/L; *mmol/L*)	3.0 to < 3.4 *3.0 to < 3.4*	2.5 to < 3.0 *2.5 to < 3.0*	2.0 to < 2.5 *2.0 to < 2.5*	< 2.0 *< 2.0*

[15] To convert a magnesium value from mg/dL to mmol/L, laboratories should multiply by 0.4114.

Chemistries

PARAMETER	GRADE 1 MILD	GRADE 2 MODERATE	GRADE 3 SEVERE	GRADE 4 POTENTIALLY LIFE-THREATENING
**Sodium, High** (mEq/L; *mmol/L*)	146 to < 150<br>*146 to < 150*	150 to < 154<br>*150 to < 154*	154 to < 160<br>*154 to < 160*	≥ 160<br>*≥ 160*
**Sodium, Low** (mEq/L; *mmol/L*)	130 to < 135<br>*130 to < 135*	125 to < 130<br>*125 to < 130*	121 to < 125<br>*121 to < 125*	≤ 120<br>*≤ 120*
**Uric Acid, High** (mg/dL; *mmol/L*)	7.5 to < 10.0<br>*0.45 to < 0.59*	10.0 to < 12.0<br>*0.59 to < 0.71*	12.0 to < 15.0<br>*0.71 to < 0.89*	≥ 15.0<br>*≥ 0.89*

Hematology

PARAMETER	GRADE 1 MILD	GRADE 2 MODERATE	GRADE 3 SEVERE	GRADE 4 POTENTIALLY LIFE-THREATENING
**Absolute CD4+ Count, Low** (cell/mm^3; *cells/L*) *> 5 years of age (not HIV infected)*	300 to < 400<br>*300 to < 400*	200 to < 300<br>*200 to < 300*	100 to < 200<br>*100 to < 200*	< 100<br>*< 100*
**Absolute Lymphocyte Count, Low** (cell/mm^3; *cells/L*) *> 5 years of age (not HIV infected)*	600 to < 650<br>*0.600 x 10^9 to < 0.650 x 10^9*	500 to < 600<br>*0.500 x 10^9 to < 0.600 x 10^9*	350 to < 500<br>*0.350 x 10^9 to < 0.500 x 10^9*	< 350<br>*< 0.350 x 10^9*
**Absolute Neutrophil Count (ANC), Low** (cells/mm^3; *cells/L*) *> 7 days of age*	800 to 1,000<br>*0.800 x 10^9 to 1.000 x 10^9*	600 to 799<br>*0.600 x 10^9 to 0.799 x 10^9*	400 to 599<br>*0.400 x 10^9 to 0.599 x 10^9*	< 400<br>*< 0.400 x 10^9*
2 to 7 days of age	1,250 to 1,500<br>*1.250 x 10^9 to 1.500 x 10^9*	1,000 to 1,249<br>*1.000 x 10^9 to 1.249 x 10^9*	750 to 999<br>*0.750 x 10^9 to 0.999 x 10^9*	< 750<br>*< 0.750 x 10^9*
≤ 1 day of age	4,000 to 5,000<br>*4.000 x 10^9 to 5.000 x 10^9*	3,000 to 3,999<br>*3.000 x 10^9 to 3.999 x 10^9*	1,500 to 2,999<br>*1.500 x 10^9 to 2.999 x 10^9*	< 1,500<br>*< 1.500 x 10^9*
**Fibrinogen, Decreased** (mg/dL; *g/L*)	100 to < 200<br>*1.00 to < 2.00*<br>OR<br>0.75 to < 1.00 x LLN	75 to < 100<br>*0.75 to < 1.00*<br>OR<br>≥ 0.50 to < 0.75 x LLN	50 to < 75<br>*0.50 to < 0.75*<br>OR<br>0.25 to < 0.50 x LLN	< 50<br>*< 0.50*<br>OR<br>< 0.25 x LLN<br>OR Associated with gross bleeding
**Hemoglobin[16], Low** (g/dL; *mmol/L*)[17] *≥ 13 years of age (male only)*	10.0 to 10.9<br>*6.19 to 6.76*	9.0 to < 10.0<br>*5.57 to < 6.19*	7.0 to < 9.0<br>*4.34 to < 5.57*	< 7.0<br>*< 4.34*
≥ 13 years of age (female only)	9.5 to 10.4<br>*5.88 to 6.48*	8.5 to < 9.5<br>*5.25 to < 5.88*	6.5 to < 8.5<br>*4.03 to < 5.25*	< 6.5<br>*< 4.03*

[16] Male and female sex are defined as sex at birth. For transgender participants ≥13 years of age who have been on hormone therapy for more than 6 consecutive months, grade hemoglobin based on the gender with which they identify (i.e., a transgender female should be graded using the female sex at birth hemoglobin laboratory values).

[17] The most commonly used conversion factor to convert g/dL to mmol/L is 0.6206. For grading hemoglobin results obtained by an analytic method with a conversion factor other than 0.6206, the result must be converted to g/dL using appropriate conversion factor for the particular laboratory.

Hematology

PARAMETER	GRADE 1<br>MILD	GRADE 2<br>MODERATE	GRADE 3<br>SEVERE	GRADE 4<br>POTENTIALLY LIFE-THREATENING
57 days of age to < 13 years of age (male and female)	9.5 to 10.4<br>*5.88 to 6.48*	8.5 to < 9.5<br>*5.25 to < 5.88*	6.5 to < 8.5<br>*4.03 to < 5.25*	< 6.5<br>*< 4.03*
36 to 56 days of age (male and female)	8.5 to 9.6<br>*5.26 to 5.99*	7.0 to < 8.5<br>*4.32 to < 5.26*	6.0 to < 7.0<br>*3.72 to < 4.32*	< 6.0<br>*< 3.72*
22 to 35 days of age (male and female)	9.5 to 11.0<br>*5.88 to 6.86*	8.0 to < 9.5<br>*4.94 to < 5.88*	6.7 to < 8.0<br>*4.15 to < 4.94*	< 6.7<br>*< 4.15*
8 to ≤ 21 days of age (male and female)	11.0 to 13.0<br>*6.81 to 8.10*	9.0 to < 11.0<br>*5.57 to < 6.81*	8.0 to < 9.0<br>*4.96 to < 5.57*	< 8.0<br>*< 4.96*
≤ 7 days of age (male and female)	13.0 to 14.0<br>*8.05 to 8.72*	10.0 to < 13.0<br>*6.19 to < 8.05*	9.0 to < 10.0<br>*5.59 to < 6.19*	< 9.0<br>*< 5.59*
**INR, High** (not on anticoagulation therapy)	1.1 to < 1.5 x ULN	1.5 to < 2.0 x ULN	2.0 to < 3.0 x ULN	≥ 3.0 x ULN
**Methemoglobin** (% hemoglobin)	5.0 to < 10.0%	10.0 to < 15.0%	15.0 to < 20.0%	≥ 20.0%
**PTT, High** (not on anticoagulation therapy)	1.1 to < 1.66 x ULN	1.66 to < 2.33 x ULN	2.33 to < 3.00 x ULN	≥ 3.00 x ULN
**Platelets, Decreased** (cells/mm^3; *cells/L*)	100,000 to < 125,000<br>*100.000 x 10^9 to < 125.000 x 10^9*	50,000 to < 100,000<br>*50.000 x 10^9 to < 100.000 x 10^9*	25,000 to < 50,000<br>*25.000 x 10^9 to < 50.000 x 10^9*	< 25,000<br>*< 25.000 x 10^9*
**PT, High** (not on anticoagulation therapy	1.1 to < 1.25 x ULN	1.25 to < 1.50 x ULN	1.50 to < 3.00 x ULN	≥ 3.00 x ULN
**WBC, Decreased** (cells/mm^3; *cells/L*)<br>*> 7 days of age*	2,000 to 2,499<br>*2.000 x 10^9 to 2.499 x 10^9*	1,500 to 1,999<br>*1.500 x 10^9 to 1.999 x 10^9*	1,000 to 1,499<br>*1.000 x 10^9 to 1.499 x 10^9*	< 1,000<br>*< 1.000 x 10^9*
≤ 7 days of age	5,500 to 6,999<br>*5.500 x 10^9 to 6.999 x 10^9*	4,000 to 5,499<br>*4.000 x 10^9 to 5.499 x 10^9*	2,500 to 3,999<br>*2.500 x 10^9 to 3.999 x 10^9*	< 2,500<br>*< 2.500 x 10^9*

Urinalysis

PARAMETER	GRADE 1 MILD	GRADE 2 MODERATE	GRADE 3 SEVERE	GRADE 4 POTENTIALLY LIFE-THREATENING
**Glycosuria** (random collection tested by dipstick)	Trace to 1+ or $\leq$ 250 mg	2+ or > 250 to $\leq$ 500 mg	> 2+ or > 500 mg	NA
**Hematuria** (not to be reported based on dipstick findings or on blood believed to be of menstrual origin)	6 to < 10 RBCs per high power field	$\geq$ 10 RBCs per high power field	Gross, with or without clots OR with RBC casts OR Intervention indicated	Life-threatening consequences
**Proteinuria** (random collection tested by dipstick)	1+	2+	3+ or higher	NA

Appendix I. Pharmacokinetic Sub-Study

Pharmacokinetics of Dihydroartemisinin-Piperaquine (DP) with Sulfadoxine-Pyrimethamine (SP) in Pregnant Women receiving DP, SP or DP and SP as Chemoprevention for Malaria

1. INTRODUCTION

1.1. Background

Pregnancy is associated with reduced dihydroartemisinin (DHA), piperaquine (PQ), sulfadoxine (SDX) and pyrimethamine (PYR) pharmacokinetic exposure compared to non-pregnant adults. Although dosing of antimalarials is based on non-pregnant adults, physiological changes in pregnancy affect drug exposure, including reduced absorption, increased distribution, diminished protein binding, and altered clearance.[B1-2] In addition, increased hepatic blood flow and CYP activity during pregnancy increases metabolism of many drugs.[B3-4] In our evaluations of IPTp with DP, compared to non-pregnant adults, pregnant women exhibited 30-40% decreased pharmacokinetic exposure to both DHA and PQ,[B5] and the lower exposure to PQ led to increased risk of malaria parasitemia and adverse birth outcomes during pregnancy.[B6] In African women receiving IPTp with SP, SDX exposure was also lower during pregnancy than post-partum.[B7] In another African study, clearance during pregnancy was increased 3-fold for SDX, but decreased 18% for PYR compared to that in non-pregnant adults;[B8] similar findings were reported from Papua New Guinea**.**[B9]

A drug-drug interaction between SP and DP is unlikely, but clinical confirmation is needed. No data are available on the pharmacokinetics or efficacy of concurrent administration of DP and SP for IPTp. There is no evidence that the components of these drugs alter metabolism or renal clearance. One small study reported increased exposure to halofantrine, a CYP3A4 substrate,[B9,B10] after a dose of SP,[B11] but others reported no clinically relevant interactions with SP.[B12-14] We have also shown that daily trimethoprim-sulfamethoxazole, a related antifolate combination, given with DP was as safe and as well tolerated as trimethoprim-sulfamethoxazole alone for IPTp among HIV infected pregnant women.[B15] In addition, others have shown that DP administered as malaria treatment among women receiving SP effectively treats malaria and does not cause additional toxicity. However, as protective efficacy is tightly linked to maintaining protective concentrations of PQ for DP, and even small decreases in PQ exposure could impact antimalarial efficacy, characterization of any potential drug interactions between PQ, SDX and PYR are needed.

1.2. Preliminary Studies:

Pharmacokinetic Studies

We have established the infrastructure to enroll pregnant women into pharmacokinetic (PK) studies at our study site.[B5,B16] Our intensive PK studies in pregnant populations have shown that compared to non-pregnant Ugandan women, pregnancy is associated with a 27% reduction in

area under the curve (AUC) from 0-8 h for DHA, and a 40% reduction in AUC from 0-21 d for PQ.[B5] These data allowed us to propose optimal DP regimens during pregnancy which improved time above malaria protective PQ concentrations. We were also able to identify a critical drug-drug interaction between PQ and efavirenz which caused further reductions in PQ exposure for women with HIV who received efavirenz.

To expand our understanding of DP and SP individually, and in combination, a designated PK study room has been established at the clinical trial site specifically for intensive PK studies. This will allow subjects to remain comfortable for up to eight hours. All PK sample handling is carried out in our onsite research laboratory where plasma samples are adequately stored at -20°C immediately after collection and then transferred from Busia to Tororo to -80°C to await shipment to UCSF for analysis. In addition to this proposed study in pregnant women, prior PK investigations have included a) artemether-lumefantrine in HIV-uninfected and HIV infected children,[B17] b) DP in HIV-uninfected infants,[B18] and c) DP in pregnant women with and without HIV.[B5]

Our studies are supported through our state-of-the-art pharmacology laboratory, the Drug Research Unit, located at UCSF with >35 years of experience in pharmacology where specialized methods for antimalarials are continuously developed and improved to support field investigations.[B19]

1.3. Rationale

This study is designed to directly address the pharmacology of DP and SP, alone and in combination, among pregnant women at high risk of malaria in Busia District, Uganda. Results will inform dosing guidelines for DP and SP alone and in combination during pregnancy. Traditionally, studies have focused on non-pregnant adults, largely ignoring the effects of pregnancy and concomitant drugs on drug disposition. This study utilizes state-of-the-art PK designs and drug assay methods. The intensive PK design in a relatively small number of women is needed to address these focused PK questions for PQ, SDX, and PYR.

Rationale for this study is:

- Dosing guidelines for pregnant women have historically relied on studies in non-pregnant adults even though pregnancy impacts how drugs are handled by the body.
- If DP and SP provide additive or even synergistic benefit for birth outcomes, it will be important to understand how each drug should be dosed for safety and efficacy during pregnancy.
- PK studies of antimalarials in vulnerable populations and drug interaction studies are deemed a high priority by the Worldwide antimalarial resistance network (WWARN) and National Institutes of Allergy and Infectious Diseases to reduce the threat of ACT drug resistance and treatment failure.
- An intensive PK design yielding a precise estimate of drug exposure (i.e. AUC) permits robust comparisons informing treatment guidelines and policy for pregnant women living in malaria endemic settings.

2. PHARMACOLOGY OBJECTIVES:

2.1. Primary Objectives

- **To evaluate the PK exposure of PQ, SDX, and PYR during pregnancy and investigate the potential drug-drug interaction between PQ and SP.** 90 pregnant women (approximately 30 each in DP, SP, or DP+SP IPTp arms) will undergo intensive PK evaluations over 21 days for PQ, SDX and/or PYR at 28 weeks gestation. We hypothesize that there will be no changes to drug exposure of PQ, SDX or PYR when DP is given with SP compared to each drug being given separately.

2.2. Secondary Objectives

- **To quantify relationships between the PK of DP and SP and toxicity.**
In addition to PK data described above, the 90 pregnant women will be assessed at each study visit for adverse events that may be drug related. We will assess associations between drug concentration or area under the curve and any grade 3 or 4 adverse event including anemia. We hypothesize that variable exposure to each drug in the context of standard dosing will not be associated with an increased risk for adverse events.

3. STUDY DESIGN

This will be a PK sub-study carried out to reach a sample size of 90 HIV-uninfected pregnant women from the DP+SP study. The PK sub-study will be carried out at the time of study drug administration at 28 weeks of gestation and sampling specific for the PK sub-study will continue until 23 days after dosing. If a woman is lost to follow up during the PK sub-study, additional women will be enrolled to reach the 90 women with PK data at all time points (>90 women may be enrolled in the study to reach this goal). The design is an intensive study that entails multiple blood collections in a relatively small sample of women in order to accurately estimate the area under the concentration time curve (AUC) for a) SDX and PYR prior to the 1st dose on day 1 and then following to 23 days post-dosing, and b) PQ prior to the 3rd daily dose of a 3 day regimen and then following to 23 days after the first dose (e.g. all women will undergo the same PK sampling regardless of which arm they are assigned to since study staff and subjects are blinded).

DP and SP PK: Women in the DP only or SP only arms will serve as the control group for the DP+SP arm for the primary outcome of assessing a drug interaction between DP+SP.

Sample size: A combined enrollment of 90 women, to achieve approximately 30 from each IPTp arm will complete the intensive PK evaluations. All participants and on-site study personnel will remain blinded regarding the assigned treatment group for all participants of this PK sub-study. Any pregnant woman who meet eligibility criteria will be approached to participate in the PK study until enrollment for the PK sub-study is complete.

4. PK SUB-STUDY SELECTION AND ENROLLMENT

4.1. Inclusion Criteria

- Enrollment in DP+SP IPTp study
- Provision of informed consent for the PK sub-study
- Willingness to undergo intensive PK sampling, starting with the 1st dose of study drugs scheduled for 28 weeks gestation and continuing through day 23 after the first dose.

4.2. Exclusion Criteria

- Receipt of any medications affecting CYP450 metabolism (as per section 4.3) within 14 days of the scheduled intensive PK evaluation.
- Hemoglobin < 8.0 g/dL at the start of the PK sub-study
- Prior treatment for clinical malaria within 14 days of the scheduled intensive PK evaluation

4.3. Disallowed Medication Guidelines

The following medications are disallowed within 14 days prior to the intensive PK evaluations. The medications are in addition to disallowed medications for the parent study:

- Carbamazepine
- Ketoconazole
- Phenobarbital
- Phenytoin
- Rifabutin
- Rifampin
- Grapefruit juice should be avoided during the study due to its potential effects on CYP3A4.

4.4. Study Enrollment Procedures

Women may be approached for this PK sub-study at any point after women enroll in the parent study and will be provided information about the study. Consent will be obtained at or before 28 weeks gestation. Prior to obtaining consent, women will be assessed for eligibility which includes determining that they are not on any disallowed medications, their hemoglobin was

≥8.0 g/dL at enrollment, and they have not had malaria diagnosed within 14 days of their 28-week study visit.

As done for the primary study, study physicians will conduct the informed consent discussion for the PK sub-study in the study clinic in the appropriate language and an interpreter will be used if necessary. Following the informed consent discussion, participants will be asked by the study physicians to sign a research participation informed consent form specific for the PK sub-study, approved by the UCSF Committee for Human Research (UCSF CHR), MU School of School of Biomedical Sciences - Research and Ethics Committee (SBS-REC), and the Uganda National Council for Science and Technology (UNCST). If the participant is unable to read or write, her fingerprint will substitute for a signature, and a signature from a witness to the informed consent procedures will be obtained as is done for the parent study.

5. PK SUB-STUDY PROCEDURES AT 28 WEEKS GESTATION

- Women will begin the study at their routine visit for 28 weeks gestation and will receive their assigned 3-day treatment course per their blinded assignment in parent study: DP + SP placebo **or** SP + DP placebo **or** DP + SP. Women will be instructed to come to the study clinic at a specified time for directly observed administration of the study drug for all doses. In all cases, the exact time of each daily dose of study drug will be recorded by study staff.

- On the first and third dosing days, an indwelling catheter will be placed in a vein to allow for serial PK sampling. A single venous sample (0.5 ml) will be collected just prior to each daily dose. After doses on the first and third days, additional blood samples (0.5 ml each) will be collected at 0.5, 1, 2, 3, 4, 6 and 8 hours after the doses are administered. Women will remain in the clinic until completion of the 8-hour blood sample, the catheter will be removed and women will be discharged home.

- On the second day of dosing, participants will come to clinic for a pre-drug venous or capillary sample collection followed by administration of study drug (with precise times recorded)

- In addition to the blood samples described above, women will return to the clinic for PK sample collections 72 h after the first study drug dose and on days 7, 9, 16, and 23 (Figure 1). The PK sample at 72 hours post first dose will be collected by venous stick (0.5 ml) and capillary stick (200 µl) to permit correlation of venous and capillary plasma drug concentrations for PQ, SDX and PYR. Samples on days 7, 9, 16 and 23 can either be collected via venous (0.5 mL) or capillary stick (200 µl each) and processed for plasma. The method and precise time of collection (venous or capillary) will always be recorded.

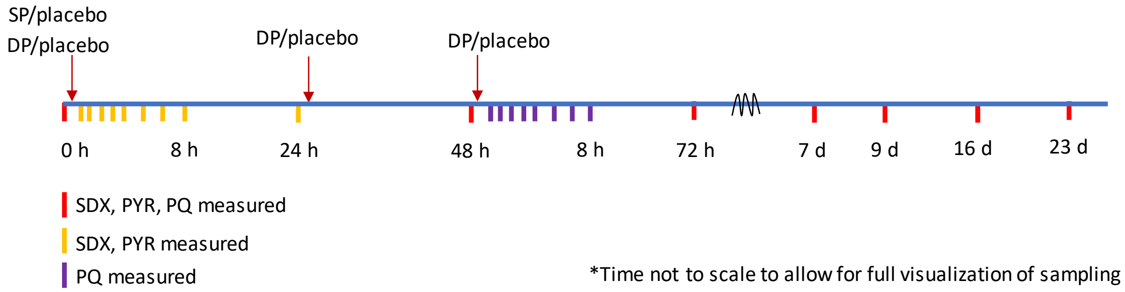

Figure 1. PK sub-study clinical visits

- Women will be provided modest compensation for their time.

- Samples from 0.5-24 hours on the first day of dosing will be used to determine SDX and PYR concentrations. Samples from 0.5-8 hours the 3rd day will be used to determine PQ concentrations, and for all remaining samples concentrations of all three drugs will be quantified.

- Subjects with malaria infection during the 21 days of the PK study will provide an additional PK sample via venous (0.5 mL) or capillary (200 µL) collection at malaria diagnosis.

- After blood is collected, plasma will be separated immediately and aliquoted into cryovials. Plasma samples will be initially placed at -20°C, then transported to the Tororo Lab within 24 hours for storage in -80°C freezers. Samples will be shipped to the UCSF Drug Research Unit on dry ice or liquid nitrogen for drug concentration quantification.

6. **ANALYTICAL METHODS.** PQ, SDX and PYR will be measured in plasma using optimized methods validated in the UCSF Drug Research Unit with all methods utilizing liquid chromatography tandem mass spectrometry. A non-study staff member will be made aware the study drug assignments for the PK sub-study participants to allow quantification

of relevant analytes in the sample. This information will not be shared with other study investigators until unblinding has occurred.

6.1. Blood Volumes

The total blood volumes collected from intensive PK study participants during the 23 days will be no more than 20 mL.

7. MISSED DOSES OF STUDY DRUG

Study drugs will be re-dosed if vomiting occurs within 30 minutes of administration. At the time of the third daily treatment dose, PK sampling will only continue if the subject can be re-dosed with a full dose and vomiting occurred ≤30 minutes post-administration of DP. The re-dosing will be carefully noted on the study case report forms. Participants in the PK sub-study must have taken all 3 daily doses successfully in order to participate in the intensive PK sampling.

8. STATISTICAL CONSIDERATIONS

Primary Analysis: We will evaluate the PK exposure of PQ, SDX, and PYR in women who receive DP, SP or DP+SP at 28 weeks gestation for women enrolled the PK sub-study. PK parameters of PQ, SDX and PYR will be estimated using non-compartmental analysis via the linear up-log down trapezoidal rule in conjunction with first-order input using WinNonlin 6.0 (Pharsight Corporation, Mountain View, USA). PK parameters, including elimination rate constant (λz) and half-life (t1/2) will be estimated with t1/2 calculated as ln2/ λz. The AUC will be estimated as the sum of AUC_{last} (AUC to the end of the sampling period) and $AUC_{last-\infty}$ (AUC from the end of sampling to infinity (∞)). If PK parameters are found to have skewed distributions and data transformations do not induce symmetry, rank-based tests will be used.

<u>**Comparison Groups**</u>

**Primary**<br>1. **To evaluate the PK exposure of PQ, SDX, and PYR during pregnancy and determine if there is a drug-drug interaction between PQ and SP.**	Pregnant women receiving monthly DP+SP in the 2nd and 3rd trimesters of pregnancy vs pregnant receiving DP only or SP only during the 2nd and 3rd trimesters of pregnancy.

**Secondary**<br>1. **To quantify relationships between the PK exposure of DP and/or SDX/PYR and toxicity.**	Pregnant women who only receive SP will be the control group for women receiving DP or DP+SP. Pregnant women who only receive DP will serve as the control group for women receiving SP or DP+SP.

8.1. <u>Outcome Measures</u>

- **Primary outcome**

Primary outcome measurement is AUC for PQ, SDX and PYR.

- **Secondary outcomes**

Grade 3 and 4 adverse events.

8.2. Sample Size and Power for primary outcome. Using measures of observed mean AUC and standard deviations from our prior studies and those published in the literature and assuming a group sample size of 30 subjects with complete data for each study drug regimen, this study will achieve 80% power to detect a difference in mean AUC between groups of 23% for PQ and 30% for SDX, and 19% for PYR with a significance level (alpha) of 0.05 using a two-sided two-sample t-test (coefficients of variation [CV] for AUC for PPQ=35%, DHA=38%, sulfadoxine=40%, pyrimethamine=25%).

Added References for Appendix I

1. Dawes M, Chowienczyk PJ. Drugs in pregnancy. Pharmacokinetics in pregnancy. Best Pract Res Clin Obstet Gynaecol. 2001;15(6):819-26. 10.1053/beog.2001.0231.

2. Aweeka FT, Stek A, Best BM, Hu C, Holland D, Hermes A, et al. Lopinavir protein binding in HIV-1-infected pregnant women. HIV Med. 2010;11(4):232-8. 10.1111/j.1468-1293.2009.00767.x.

3. Mirochnick M, Best BM, Stek AM, Capparelli E, Hu C, Burchett SK, et al. Lopinavir exposure with an increased dose during pregnancy. J Acquir Immune Defic Syndr. 2008;49(5):485-91. 10.1097/QAI.0b013e318186edd0.

4. Read JS, Best BM, Stek AM, Hu C, Capparelli EV, Holland DT, et al. Pharmacokinetics of new 625 mg nelfinavir formulation during pregnancy and postpartum. HIV Med. 2008;9(10):875-82. 10.1111/j.1468-1293.2008.00640.x.

5. Kajubi R, Huang L, Jagannathan P, Chamankhah N, Were M, Ruel T, et al. Antiretroviral therapy with efavirenz accentuates pregnancy-associated reduction of dihydroartemisinin-piperaquine exposure during malaria chemoprevention. Clin Pharmacol Ther. 2017;102(3):520-8. 10.1002/cpt.664.

6. Savic RM, Jagannathan P, Kajubi R, Huang L, Zhang N, Were M, et al. Intermittent Preventive Treatment for Malaria in Pregnancy: Optimization of Target Concentrations of Dihydroartemisinin-Piperaquine. Clin Infect Dis. 2018;67(7):1079-88. 10.1093/cid/ciy218.
7. Nyunt MM, Adam I, Kayentao K, van Dijk J, Thuma P, Mauff K, et al. Pharmacokinetics of sulfadoxine and pyrimethamine in intermittent preventive treatment of malaria in pregnancy. Clin Pharmacol Ther. 2010;87(2):226-34. 10.1038/clpt.2009.177.
8. de Kock M, Tarning J, Workman L, Nyunt MM, Adam I, Barnes KI, et al. Pharmacokinetics of Sulfadoxine and Pyrimethamine for Intermittent Preventive Treatment of Malaria During Pregnancy and After Delivery. CPT Pharmacometrics Syst Pharmacol. 2017;6(7):430-8. 10.1002/psp4.12181.
9. Karunajeewa HA, Salman S, Mueller I, Baiwog F, Gomorrai S, Law I, et al. Pharmacokinetics of chloroquine and monodesethylchloroquine in pregnancy. Antimicrob Agents Chemother. 2010;54(3):1186-92. 10.1128/AAC.01269-09.
10. Baune B, Furlan V, Taburet AM, Farinotti R. In vitro effects of racemates, separate enantiomers and major metabolites of mefloquine and halofantrine on metoprolol biotransformation by rat liver microsomes. Xenobiotica. 1999;29(6):595-601. 10.1080/004982599238416.
11. Hombhanje FW. Effect of a single oral dose of Fansidar on the pharmacokinetics of halofantrine in healthy volunteers: a preliminary report. Br J Clin Pharmacol. 2000;49(3):283-4. 10.1046/j.1365-2125.2000.00142.x.
12. Tekete MM, Toure S, Fredericks A, Beavogui AH, Sangare CP, Evans A, et al. Effects of amodiaquine and artesunate on sulphadoxine-pyrimethamine pharmacokinetic parameters in children under five in Mali. Malar J. 2011;10:275. 10.1186/1475-2875-10-275.
13. German PI, Aweeka FT. Clinical pharmacology of artemisinin-based combination therapies. Clin Pharmacokinet. 2008;47(2):91-102. 10.2165/00003088-200847020-00002.
14. Chhonker YS, Bhosale VV, Sonkar SK, Chandasana H, Kumar D, Vaish S, et al. Assessment of Clinical Pharmacokinetic Drug-Drug Interaction of Antimalarial Drugs alpha/beta-Arteether and Sulfadoxine-Pyrimethamine. Antimicrob Agents Chemother. 2017;61(9). 10.1128/AAC.02177-16.
15. Natureeba P, Kakuru A, Muhindo M, Littmann E, Ochieng T, Ategeka J, et al. Intermittent Preventive Treatment with Dihydroartemisinin-piperaquine for the Prevention of Malaria among HIV-infected Pregnant Women. J Infect Dis. 2017;216(1):29-35. 10.1093/infdis/jix110.
16. Kajubi R, Ochieng T, Kakuru A, Jagannathan P, Nakalembe M, Ruel T, et al. Monthly sulfadoxine-pyrimethamine versus dihydroartemisinin-piperaquine for intermittent preventive treatment of malaria in pregnancy: a double-blind, randomised, controlled, superiority trial. Lancet. 2019;393(10179):1428-39. 10.1016/S0140-6736(18)32224-4.
17. Parikh S, Kajubi R, Huang L, Ssebuliba J, Kiconco S, Gao Q, et al. Antiretroviral Choice for HIV Impacts Antimalarial Exposure and Treatment Outcomes in Ugandan Children. Clin Infect Dis. 2016;63(3):414-22. 10.1093/cid/ciw291.
18. Whalen ME, Kajubi R, Chamankhah N, Huang L, Orukan F, Wallender E, et al. Reduced Exposure to Piperaquine, Compared to Adults, in Young Children Receiving

Dihydroartemisinin-Piperaquine as Malaria Chemoprevention. Clin Pharmacol Ther. 2019. 10.1002/cpt.1534.

19. Mwebaza N, Cheah V, Forsman C, Kajubi R, Marzan F, Wallender E, et al. Determination of piperaquine concentration in human plasma and the correlation of capillary versus venous plasma concentrations. PLoS One. 2020;15(5):e0233893. 10.1371/journal.pone.0233893.
