# Supplementary material for: Dihydroartemisinin–piperaquine plus sulfadoxine–pyrimethamine versus either drug alone for intermittent preventive treatment of malaria in pregnancy: A double-blind, randomized, controlled phase 3 trial from Uganda
Source: PLoS Med. 2025 Sep 18;22(9):e1004582. doi: 10.1371/journal.pmed.1004582 (PMC12445490; doi:10.1371/journal.pmed.1004582)
Supplement: S3 File — (PDF) [file pmed.1004582.s004.pdf]

Statistical Analysis Plan

TRIAL FULL TITLE	**Optimal chemopreventive regimens to prevent malaria and improve birth outcomes in Uganda**
ClinicalTrials.gov Identifier:	NCT04336189
VERSION	1.0
VERSION DATE	20-November-2023
TRIAL STATISTICIAN	Grant Dorsey, MD, PhD<br>Michelle Roh, PhD MPH
TRIAL PRINCIPAL INVESTIGATORS	Grant Dorsey, MD, PhD<br>Moses Kamya, MBChB, MMed, PhD<br>Philip Rosenthal, MD

Table of Contents

Table of Contents 2

1 Abbreviations and Definitions 4

2 Introduction 5

2.1 Preface 5

2.2 Purpose of the analyses 5

3 Study Objectives and Endpoints 6

3.1 Study Objectives 6

3.2 Endpoints 6

3.3 General Study Design and Plan 8

3.4 Equivalence or Non-Inferiority Studies 8

3.5 Inclusion-Exclusion Criteria and General Study Population 8

3.6 Randomization and Blinding 9

3.7 Study Variables 9

Table 2. Schedule of routine assessments and procedures in pregnant women 9

Table 3. Study Case Report Forms 10

4 Sample Size 10

5 General Considerations 11

5.1 Timing of Analyses 11

5.2 Analysis Populations 11

5.2.1 Full Analysis Population 11

5.2.2 Per Protocol Population 11

5.3 Covariates and Subgroups 11

5.4 Missing Data 12

5.5 Interim Analyses and Data Monitoring 12

5.5.1 Purpose of Interim Analyses 12

5.5.2 Planned Schedule of Interim Analyses 12

Table 4. Schedule of interim safety analysis and boundaries to monitor study outcome 12

5.5.3 Scope of Adaptations 13

5.5.4 Stopping Rules 13
5.5.5 Adjustment of Confidence Intervals and p-values 13
5.5.6 Interim Analysis for Sample Size Adjustment 13
5.5.7 Practical Measures to Minimize Bias 13
5.5.8 Documentation of Interim Analyses 13
5.6 Multi-center Studies 13
5.7 Multiple Testing 13
6 Summary of Study Data 14
6.1 Trial Profile 14
Table 5. Trial profile 14
6.2 Baseline Characteristics 15
Table 6. Baseline characteristics of study participants randomized to IPTp 15
6.3 Treatment Adherence 15
Table 7. Measures of treatment adherence 16
7 Efficacy Analyses (Objectives 1 and 3) 17
7.1 Efficacy outcomes 17
Table 8. Primary and secondary outcomes 17
7.2 Primary Efficacy Analysis 19
Table 9. Outcomes assessed at the time of delivery 20
8 Safety and Tolerability Analyses (Objective 2) 22
8.1 Adverse Events 22
8.2 Serious Adverse Events 22
8.3 Analytical Methods 23
Table 11. Measures of safety and tolerability 23
9 References 24

1 Abbreviations and Definitions

ACT	Artemisinin-based combination therapy
AE	Adverse Event
AL	Artemether-lumefantrine
CAB	Community advisory board
CBC	CBC Complete blood cell
CRF	Case Report Form
DP	Dihydroartemisinin-piperaquine
DSMB	Data and Safety Monitoring Board
IDRC	Infectious Diseases Research Collaboration
IPT	Intermittent preventive therapy
IPTp	Intermittent preventive therapy in pregnancy
IRB	Institutional review board
LLIN	Long-lasting insecticide treated net
MGH	Masafu General Hospital
MOH	Ministry of Health
MU	Makerere University
NIH	National Institute of Health
SAE	Serious adverse event
SAP	Statistical Analysis Plan
SP	Sulfadoxine-pyrimethamine
UCSF	University California San Francisco
WHO	WHO World Health Organization

2 Introduction

2.1 Preface

Malaria in pregnancy remains a major challenge in Africa, where approximately 50 million women are at risk for *P. falciparum* infection during pregnancy each year.[1] Among pregnant women living in malaria endemic areas characteristic of much of Africa, symptomatic disease is uncommon, but infection with malaria parasites is associated with maternal anemia and adverse birth outcomes including abortions, stillbirth, preterm birth, low birth weight (LBW), and infant mortality.[2] Thus, malaria in pregnancy causes an estimated 900,000 LBW deliveries and 100,000 infant deaths each year.[3,4] The World Health Organization (WHO) recommends the use of long-lasting insecticidal nets (LLINs) and intermittent preventive treatment with sulfadoxine-pyrimethamine (IPTp-SP) for the prevention of malaria in pregnancy in endemic areas of Africa. However, there is concern for diminishing efficacy of these interventions due to the spread of vector resistance to the pyrethroid insecticides used in LLINs and parasite resistance to SP.[5,6] Thus, there is an urgent need for new strategies for the prevention of malaria in pregnancy and improving birth outcomes.

Artemisinin-based combination therapies (ACTs) are now the standard treatment for malaria in Africa. Dihydroartemisin-piperaquine (DP) is a fixed-dose ACT and an attractive alternative to SP for IPTp. DP is highly efficacious and the long half-life of piperaquine provides at least 4 weeks of post-treatment prophylaxis.[7,8] Three randomized controlled trials from our group and others have shown that, compared to IPTp with SP, IPTp with DP dramatically reduced risks of maternal parasitemia and symptomatic malaria, maternal anemia, and placental malaria.[9-11] In addition, IPTp with DP was as safe and well tolerated as SP. Surprisingly, despite significant reductions in risks of malaria-specific outcomes afforded by DP in these studies, there were no differences between the SP and DP groups in risks of adverse birth outcomes, including LBW and preterm birth. Indeed, in a recent WHO Malaria Policy Advisory Committee concluded that, although IPTp with DP merits further study, SP should remain the recommended drug for IPTp until there is conclusive evidence that alternative regimens improve birth outcomes.[12] Thus, the standard-of-care remains use of a poorly effective antimalarial to prevent malaria in pregnancy.

2.2 Purpose of the analyses

The key question motivating this proposal is why IPTp with either SP or DP is associated with similar risks of adverse birth outcomes despite the far superior antimalarial activity of DP. The likely explanation is that SP, a broad-spectrum antibiotic, protects against non-malarial causes of LBW and preterm birth.[13] In this regard, several studies reported that increased frequency of doses of IPTp with SP was associated with improved birth outcomes and a lower risk of reproductive tract infections (RTIs), despite widespread SP resistance among malaria parasites.[14,15] Our central hypothesis is that SP improves birth outcomes independent of its antimalarial activity and that IPTp with a combination of SP+DP will offer antimalarial and non-antimalarial benefits, thus providing superior prevention of adverse birth outcomes compared to either drug used alone. To test this hypothesis we will conduct a double-blinded randomized clinical trial in a rural area of Uganda with very high malaria transmission intensity where our group already has an established infrastructure for clinical research.

3 Study Objectives and Endpoints

3.1 Study Objectives

(ICH E3; 8.)

Objective 1: **To compare the risk of adverse birth outcomes among pregnant women randomized to receive monthly IPTp with SP vs. DP vs. SP+DP.** We will conduct a randomized controlled trial in 2757 women to test the hypothesis that pregnant women who receive IPTp with SP+DP will have a lower risk of a composite outcome of LBW, preterm birth, small-for-gestational age, fetal loss or neonatal death compared to those who receive either SP or DP alone.

Objective 2: To compare safety and tolerability of IPTp regimens among pregnant women randomized to receive monthly IPTp with SP vs. DP vs. SP+DP. We will test the hypothesis that pregnant women who receive IPTp with SP+DP will have non-inferior risks of adverse events and measures of tolerability compared to those who receive either SP or DP alone.

Objective 3: To compare risks of malaria-specific and non-malarial outcomes among pregnant women randomized to receive monthly IPTp with SP vs. DP vs. SP+DP. We will test the hypotheses that a) pregnant women who receive IPTp with DP containing regimens will have a lower risk of maternal and placental malaria but a higher risk of *P. falciparum* markers of DP resistance compared to those who receive SP alone; and b) pregnant women who receive IPTp with SP containing regimens will have a lower risk of RTIs and a higher risk of *P. falciparum* markers of SP resistance compared to those who receive DP alone. Further, we will explore impacts of study drugs on the gut and vaginal microbiomes, as differences are likely to impact upon birth outcomes.

3.2 Endpoints

(ICH E9; 2.2.2)

3.2.1. Primary Outcomes

The primary outcome for objective 1 will be a composite adverse birth outcome, defined as any of the following: spontaneous abortion, stillbirth, LBW, preterm birth, SGA, or neonatal death.

The primary outcome for objective 2 will be the incidence of any grade 3-4 adverse event (AE) or serious adverse event (SAE).

The primary outcome for objective 3a will be the detection of malaria parasites at delivery.

The primary outcome for objective 3b will be the prevalence of reproductive tract infections (RTIs) during pregnancy.

3.2.2. Secondary Outcomes

Secondary outcomes for objective 1 will include individual adverse birth outcomes that make up our composite indicator, birthweight, gestational age at delivery, birthweight-for-gestational age z-score, composite of fetal loss and neonatal mortality, and composite adverse birth outcome among live births.

Secondary outcomes for objective 2 will include the incidence of individual AEs, AEs related to study drugs, measures of tolerability of study drugs, congenital anomalies, and maternal mortality.

Secondary outcomes for objective 3a will include measures of malaria, parasitemia, anemia, and DP drug resistance markers during pregnancy.

Secondary outcomes for objective 3b will be changes in the vaginal/gut microbiota, prevalence of STIs/RTIs at delivery, measures of SP drug resistance, change in maternal nutritional status, and gestational weight gain per week.

Study outcomes and definitions are summarized below in Table 1.

Table 1. Study outcomes

Aim	Outcomes	Definition
1	Composite adverse birth outcome	Spontaneous abortion, stillbirth, LBW, preterm birth, SGA, or neonatal death
1	Spontaneous abortion	Fetal loss at < 28 weeks gestational age
1	Stillbirth	Infant born deceased at ≥ 28 weeks gestational age
1	LBW	Live birth with birth weight < 2500 gm
1	Preterm birth	Live birth at < 37 weeks gestational age
1	SGA	Live birth with weight-for-gestational age < 10th percentile of reference population[16]
1	Neonatal death	Live birth with neonatal death within the first 28 days of life
1	Birthweight	Birthweight in grams among live births
1	Gestational age at delivery	Gestational age in weeks estimated by ultrasound among live births
1	Birthweight-for-gestational age z-score	Birthweight-for-gestational age z-scores among live births. Z-scores calculated based on INTERGROWTH-21st standards[17]
1	Composite of fetal loss and neonatal mortality	Spontaneous abortion, stillbirth, or neonatal death
2	Incidence of any grade 3-4 AE or SAE	Based on a standardized AE grading scale[18]
2	Incidence of individual AEs	
2	Incidence of AEs related to study drugs	
2	Congenital anomalies	Physical abnormality of live newborn or stillbirths detected at delivery
2	Maternal mortality	Maternal death up to 4 weeks after delivery
2	Tolerability of study drugs	Vomiting following administration of study drugs and measures of non-adherence
3a	Detection of malaria parasites at delivery	Parasites detected by microscopy or qPCR in maternal blood, placental blood/tissue
3a	Placental malaria	Detection of malaria parasites or pigment by placental histopathology
3a	Incidence of symptomatic malaria during pregnancy	New episodes of fever plus positive blood smear per person time
3a	Prevalence of parasitemia during pregnancy	Proportion of routine samples with asexual parasites detected by microscopy or qPCR
3a	Prevalence of congenital malaria infection	Proportion of women with parasites detected by microscopy or qPCR in fetal cord blood
3a	Prevalence of anemia during pregnancy	Proportion of routine hemoglobin measurements < 11 g/dL
3a	Prevalence of markers of DP resistance	Proportion of parasite positive samples with molecular markers of DP resistance
3b	Prevalence of STIs/RTIs at delivery	Proportion of vaginal samples collected at delivery positive for STIs/RTIs
3b	Prevalence of markers of SP resistance	Proportion of parasite positive samples with molecular markers of SP resistance
3b	Changes in the vaginal/intestinal microbiota	Relative proportions of different taxonomic groups in vaginal or gut microbiota
3b	Maternal mid-upper arm circumference at last clinic visit before delivery	Mid-upper arm circumference (MUAC) measured at last clinic visit before delivery (continuous; cm); adjusted for values measured on the day study drugs first given
3b	Gestational weight gain per week	Maternal weight measured at the last clinic visit before delivery minus the maternal weight at on the day study drugs first given, divided by the number of weeks between those dates; adjusted for values on the day study drugs first given

Study methods

3.3 General Study Design and Plan

(ICH E3;9)

This will be a double-blinded randomized controlled phase III trial of 2757 HIV uninfected pregnant women. HIV uninfected women at 12-20 weeks gestation will be randomized in equal proportions to one of three IPTp treatment arms: 1) SP given every 4 weeks, or 2) DP given every 4 weeks, or 3) SP+DP given every 4 weeks. SP or DP placebos will be used to ensure adequate blinding is achieved. Follow-up for the pregnant women will end 28 days after giving birth.

Recruitment will take place in two phases. In phase 1, 300 participants will be enrolled over 2-3 months. Phase 1 participants will undergo intensive safety and pharmacokinetic assessment. At the end of phase 1, an interim analysis of safety data will be performed and presented to the Data and Safety Monitoring Board (DSMB) as described in detail in the Protection of Human Subjects section and study protocol. If the DSMB approves, phase 2 of recruitment will begin and continue until the target sample size has been reached.

3.4 Equivalence or Non-Inferiority Studies

(ICH E3; 9.2, 9.7.1, 11.4.2.7. ICH E9; 3.3.2)

N/A - This trial is designed as a superiority trial.

3.5 Inclusion-Exclusion Criteria and General Study Population

(ICH E3;9.3. ICH E9;2.2.1)

3.5.1. Inclusion Criteria

1) Viable singleton pregnancy confirmed by ultrasound
2) Estimated gestational age between 12-20 weeks
3) Confirmed to be HIV uninfected by rapid test
4) 16 years of age or older
5) Resident of Busia District, Uganda
6) Provision of informed consent
7) Agreement to come to the study clinic for any febrile episode or other illness and avoid medications given outside the study protocol
8) Willing to deliver in the hospital

3.5.2. Exclusion Criteria

1) History of serious adverse event to SP or DP
2) Active medical problem requiring inpatient evaluation at the time of screening
3) Intention of moving outside of Busia District, Uganda
4) Chronic medical condition requiring frequent medical attention
5) Prior chemopreventive therapy or any other antimalarial therapy during this pregnancy
6) Early or active labor
7) Multiple pregnancies (i.e. twins/triplets)

3.6 Randomization and Blinding

(ICH E3; 9.4.3, 9.4.6. ICH E9; 2.3.1, 2.3.2)

There will be 3 treatment arms for the woman during pregnancy; SP given every 4 weeks, DP given every 4 weeks or a combination of SP+DP given every 4 weeks. We will use a 1:1:1 randomization scheme targeting 919 pregnant women in each treatment arm. A randomization list will be computer generated by a member of the project who will not be directly involved in the conduct of the study. The randomization list will include consecutive treatment numbers with corresponding random treatment assignments. Randomized codes will correspond to the 3 treatment arms using permuted variable sized blocks of 6 and 9. Sealed copies of the original randomization list and documentation of the procedure used to generate the lists will be stored in the project administrative offices in San Francisco and Kampala. Prior to the onset of the study, a set of sequentially numbered, opaque, sealed envelopes will be prepared. Each envelope will be marked on the outside with the treatment allocation number. The inside of the envelope will contain a piece of paper with the treatment allocation number and treatment group assignment along with a piece of carbon paper.

3.7 Study Variables

(ICH E3; 9.5.1. ICH E9; 2.2.2)

Table 2. Schedule of routine assessments and procedures in pregnant women

Evaluations and Interventions	Enrollment	Weeks of gestation								Delivery	1 and 4 weeks postpartum
		16*	20	24	28	29	32	36	40		
**Informed consent**	X										
**HIV testing[1]**	X									X	
**Obstetrical ultrasound[2]**	X			X			X	X**			
**Blood collected by phlebotomy for CBC**	X		X		X			X		X	
**Blood collected by finger prick for blood smear and dried blood spot**	X	X	X	X	X		X	X	X	X	
**Routine assessment in the study clinic[3]**	X	X	X	X	X		X	X	X		X
**ECG testing and collection of plasma samples for PK studies (phase 1 only)[4]**			X		X			X			
**Administration of study drugs**		X	X	X	X		X	X	X		
**Collection of cord blood and placental blood/tissue**										X	
**Labor and delivery documentation[5]**										X	
**Collection of gut and vaginal samples for microbiome studies**	X					X**	X				
**Collection samples for testing of STIs/RTIs**										X	
Standard Care											
**Obstetrical exam[6]**	X	X	X	X	X		X	X	X	X	X
**Syphilis screening**	X										
**Iron and Folic Acid**	X	X	X	X	X		X	X	X		
**Prenatal vitamins**	X										
**Mebendazole[7]**		X									
**Vitamin A[8]**										X	
**Insecticide treated bednet**	X										

* Only if study subject enrolled prior to 16 weeks gestation
** For a subset of individuals

Explanation of maternal schedule of events

1. HIV test will be done at enrollment and documented. A repeat rapid HIV test will be done at delivery. HIV testing shall be done using standard rapid HIV-testing algorithm.
2. Ultrasound will be done to confirm intrauterine pregnancy and estimate gestational age at enrollment.
3. Targeted physical exam will include anthropometric measurements (e.g. weight) and vital signs (i.e. temperature, pulse, and blood pressure). Measurement of height at the enrollment visit only.
4. For women enrolled in phase 1 of the study, pre- and post-study drug ECG will be performed and finger-prick blood samples will be collected to measure piperaquine levels.
5. Labor & Delivery documentation will include: Peripartum history, mode of delivery, Apgar scores (when available), weight, length, and head circumference of the child at birth, approximate gestational age, duration of labor, signs of fetal distress (presence of meconium), summary of events in first days of life (including feeding, breathing patterns, jaundice, lethargy, or any additional abnormal findings), duration of admission if delivered in hospital.
6. Obstetrical exam includes estimation of gestational age at study entry, fundal height measurement, fetal heart tones and urine dipstick for protein. A cervical exam will also be performed at screening and during antepartum study visits as clinically indicated.
7. Mebendazole is typically given as 500mg as a single dose as early as possible after the 1st trimester (16 or 20 week visit).
8. Vitamin A supplementation is dosed as 200,000 IU.

Table 3. Study Case Report Forms

Screening Form
Enrollment Form
Clinic Visit Form
AE Form
Mother Delivery Form
Ultrasound Form
Placental Histopathology Form
Hospital Admission Form
Postpartum Form
Study Drug Dispensing Form
Subject Death Form
Subject Withdrawal or Study Completion Form
ECG Form

4 Sample Size

(ICH E3; 9.7.2. ICH E9; 3.5)

Our sample size calculation is based on testing the primary hypothesis for specific aim 1. Based on our previous study from the same site, the risk of the composite adverse birth outcome as defined for aim 1 was 22.6% in the SP and 25.1% in the DP arm among women meeting the eligibility criteria

for this proposal. Given these estimates, we would need to enroll 2757 women (assuming 15% loss to follow-up) to have 80% power (2-sided alpha = 0.05) to detect a 25% or greater reduction in the risk of our composite adverse birth outcome in the SP+DP arm compared to either of the other arms. For the primary outcome of specific aim 2, we assume that the incidence of any grade 3-4 AE or SAE will be 0.37 per person year (SD=0.57) in the SP arm and 0.18 per person year (SD=0.35) in the DP arm, based on our previous study. Given these estimates we will have 80% power (1-sided alpha = 0.025) for our non-inferiority margin to be set at 0.07 when comparing the SP+DP arm to the SP arm and at 0.03 when comparing the SP+DP arm to the DP arm. For the primary outcome of specific aim 3a, we assume that the risk of detection of malaria parasites at delivery will be 12.5% in the SP arm based on our previous study. Given our sample size, we will have 80% power (2-sided alpha = 0.05) to detect a 32% or greater reduction in the prevalence of malaria parasites at delivery in the SP+DP arm or the DP arm compared to the SP arm.

5 General Considerations

5.1 Timing of Analyses

The final trial analysis for the study will be performed after the last enrolled woman has given birth, complete 4 weeks of follow-up postpartum, and all outcome measures have been assessed. Prior to the final analysis we will perform an interim safety analysis when the first 300 women enrolled have been followed through delivery (phase 1) and then every 6 months during phase 2 of the study. The interim safety analyses will compare the incidence of significant adverse events (grade 3/4 & SAEs).

5.2 Analysis Populations

(ICH E3; 9.7.1, 11.4.2.5. ICH E9; 5.2)

A modified intention-to-treat approach to all analyses will be used, including all study participants randomized to therapy and have the outcome of interest measured including all follow-up time until the study participant completes the study or early study termination regardless of whether the intervention was stopped due to an adverse event. Analyses for safety and tolerability endpoints will be conducted using a subset of the modified intention-to-treat population, consisting of all randomised subjects who received at least one dose of the study drug. All subjects will be analysed according to their randomized treatment arm.

5.2.1 Full Analysis Population

- *All subjects who were randomized*
- *All subjects with evaluable data on specific outcomes*

5.2.2 Per Protocol Population

A per protocol analysis is not planned.

5.3 Covariates and Subgroups

(ICH E3; 9.7.1, 11.4.2.1. ICH E9; 5.7)

Covariates of interest will include baseline measures of maternal age, gestational age at enrollment, gravidity, LLIN ownership, socio-economic status as estimated by a household wealth index, and prevalence of malaria parasites.

Sub-group analyses will be performed based on categories of maternal age, gravidity, infant sex, and gestational age at the time study drugs were first administered.

5.4 Missing Data

(ICH E3; 9.7.1, 11.4.2.2. ICH E9;5.3. EMA Guideline on Missing Data in Confirmatory Clinical Trials)

Women in the trial who are prematurely withdrawn from the study or are not able to provide data for specific outcomes will be considered un-evaluable and will not be included in the primary trial analysis.

5.5 Interim Analyses and Data Monitoring

(ICH E3; 9.7.1, 11.4.2.3. ICH E9; 4.1, FDA Feb 2010 "Guidance for Industry Adaptive Design Clinical Trials for Drugs and Biologics")

5.5.1 Purpose of Interim Analyses

Over the course of the trial, we will perform interim safety analyses when the first 300 women enrolled have been followed through delivery (phase 1) and then every 6 months during phase 2 of the study. The interim safety analyses will compare the incidence rate ratio of significant adverse events (grade 3/4 & SAEs). The interim analyses will not include an evaluation of efficacy outcomes.

5.5.2 Planned Schedule of Interim Analyses

An interim safety analysis for pregnant women will be performed when the first 300 women enrolled have been followed through delivery (phase 1) and then every 6 months during phase 2 of the study. A standardized test statistic will be calculated for the incident rate ratio of significant adverse events (grade 3/4 & SAEs). If this statistic exceeds the nominal critical value calculated using the error spending function (Table 4), then a statistically significant result will have been achieved at the time of the analysis. In that event, the sponsor will be notified and a report submitted for review by the Data Safety Monitoring Board (DSMB). For the interim safety analyses, the study team will present information on recruitment and the results of interim safety analyses to the DSMB, which will review the data and recommend a course of action.

Table 4. Schedule of interim safety analysis and boundaries to monitor study outcome

Number of Evaluable Subjects Accrued	Test Statistic		Alpha	Cumulative Alpha
	Lower Bound	Upper Bound		
N=300 (phase 1)	-5.37	5.37	0.0000001	0.0000001
N=791 (phase 2)	-3.71	3.71	0.0002069	0.000207
N=1282 (phase 2)	-2.97	2.97	0.002844	0.003051
N=1773 (phase 2)	-2.54	2.54	0.009046	0.0121
N=2264 (phase 2)	-2.25	2.25	0.01605	0.02815
N=2757 or 100% of accrual	-2.04	2.04	0.02185	0.05

This analysis assumes α=0.05 (two-sided test), O'Brien-Fleming boundaries (DeMets error-spending function) and 2757 trial participants. We will utilize Programs for Computing Group Sequential Boundaries Using the Lan-DeMets Method.

5.5.3 Scope of Adaptations

At the time of the interim analyses, the DSMB may decide to continue, stop, or modify the trial based on the interim safety analysis. This may include the discontinuation of a study arm and re-randomization or cessation of subject participation in the stopped arm.

5.5.4 Stopping Rules

The DSMB will determine whether to stop the study for early evidence of intervention safety problems after a thorough review of interim data. Interim reports will provide cumulative enrollment figures and cumulative adverse birth outcomes, serious adverse events (classified according to grade), sorted by study arm. Brief clinical descriptions of key events will also be provided. The PIs will be responsible for immediately reporting to the funding agency any temporary or permanent suspension of the project and the reason for the suspension.

5.5.5 Adjustment of Confidence Intervals and p-values

As appropriate we will adjust p-values and confidence intervals taking into account the specified error spending functions and interim evaluation of the data.

5.5.6 Interim Analysis for Sample Size Adjustment

The sample size will not be adjusted based on the results of the interim analysis.

5.5.7 Practical Measures to Minimize Bias

The study will establish and control who will have access to what information at each stage of the trial. Uncontrolled reporting of interim analyses to study investigators responsible for recruiting subjects will not occur.

The following measure will be taken to minimize bias:

- Only the study statistician and assistant statistician will perform the interim analysis.
- Only the statisticians and the DSMB will see any data or analyses at the interim analysis
- No information will be publicly available following an interim analysis
- Information will be provided to the sponsor and investigators as per recommendation of the study DSMB.
- Only the statisticians will be unblinded at for the interim analysis

5.5.8 Documentation of Interim Analyses

Snapshots of the data available at each interim analysis will be preserved, as will all documentation of analysis plans, programming code and reporting provided at the interim analysis.

5.6 Multi-center Studies

(ICH E3;9.7.1, 11.4.2.4. ICH E9; 3.2)

This is a single center study.

5.7 Multiple Testing

(ICH E3; 9.7.1, 11.4.2.5. ICH E9; 2.2.5)

This study has one primary outcome for each objective and multiple secondary outcomes. No formal adjustment of p-values and confidence intervals will be made for the comparison of multiple a priori outcomes.

6 Summary of Study Data

All continuous variables will be summarized using the following descriptive statistics: n (non-missing sample size), mean, standard deviation, median, interquartile range, maximum and minimum. The frequency and percentages (based on the non-missing sample size) of observed levels will be reported for all categorical measures. All summary tables will be structured with a column for each treatment and will be annotated with the total population size relevant to that table/treatment, including any missing observations.

6.1 Trial Profile

The overall study profile will be presented as a figure following CONSORT guidelines. The total numbers of women screened, excluded during screening (including criteria for exclusion) and enrolled will be presented. The numbers of women enrolled in each treatment arm and followed through each stage of the trial profile are presented in skeleton Table 5 below.

Table 5. Trial profile

Screened		###	
Excluded during screening*		###	
	**Treatment arm**		
	**SP**	**DP**	**DP+SP**
Enrolled and randomized	###	###	###
Withdrawn before receiving study drugs*	###	###	###
Received at least one dose of study drugs	###	###	###
Withdrawn before delivery*	###	###	###
Delivered	###	###	###
Withdrawn after delivery*	###	###	###
Completed 4-week post-partum visit	###	###	###

* Specific reasons for exclusion or withdrawal will be reported

6.2 Baseline Characteristics

Skeleton table of all baseline variables collected on the day of enrollment that will be presented are provided in Table 6 below.

Table 6. Baseline characteristics of study participants randomized to IPTp

Characteristic	Treatment arm		
	SP (n=XXX)	DP (n=XXX)	DP+SP (n=XXX)
Age in years, mean (SD)			
Gestational age in weeks, mean (SD)			
Gestational age categories, n (%)			
12-16 weeks			
>16-20 weeks			
Gravidity, n (%)			
1			
2			
≥ 3			
Bednet ownership, n (%)			
None			
Untreated net			
Long-lasting insecticide-treated net			
Household wealth index, n (%)			
Lowest tertile			
Middle tertile			
Highest tertile			
Weight in kg, mean (SD)			
Height in cm, mean (SD)			
Maternal MUAC, mean (SD)			
Maternal malnutrition, n (%)			
Laboratory values, mean (SD)			
WBC count per mm^3			
Neutrophil count per mm^3			
Platelet count per mm^3			
Hemoglobin g/dL			
Detection of malaria parasites by microscopy, n (%)			
Detection of malaria parasites by microscopy or qPCR, n (%)			

6.3 Treatment Adherence

During pregnancy, women will be given 1 of 3 treatment regimens: 1) SP given every 4 weeks during pregnancy, 2) DP given every 4 weeks, or 3) SP+DP given every 4 weeks during pregnancy. Each treatment with SP will be given as a single dose consisting of 3 full strength tablets. Each treatment with DP will consist of 3 full strength tablets given once a day for 3 consecutive days. In addition, placebos will be used to mimic the identical dosing strategy such that every 4 weeks women will receive two drugs on day 1 (SP and placebo, DP and placebo, or SP and DP) followed by one drug on days 2 and 3 (DP or placebo). Two placebos will be used, one that mimics the appearance of SP and one that mimics the appearance of DP. Administration of all study drugs will be double blinded such that study participants and study staff will be blinded to study treatments with the exception of the study pharmacist and pharmacy technician, who will not be involved with patient care or assessment

of study outcomes. All doses of study drugs will be pre-packaged by a study pharmacist and administered by a study nurse blinded to the study participant's treatment regimen. All doses of SP (or SP placebo) administered will be directly observed in the clinic. For DP (or DP placebo), the first of the 3 daily doses will be directly observed in the clinic and the 2nd and 3rd daily doses will be administered at home using pre-packaged study drugs in opaque envelopes with dosing instructions written on the outside. For doses of study drugs administered in the clinic, if a study participant vomits the study drug within 30 minutes of administration, the drug will be re-administered. For doses of study drugs administered at home, if a study participant vomits the study drug within 30 minutes of administration or study drug is lost, the study participant will be instructed to come to the study clinic as soon as possible where the study drug will be re-administered/replaced. For pregnant women all doses of study drugs will be given between 16 and 40 weeks gestation. Measures of treatment adherence are summarized in skeleton Table 7 below.

Table 7. Measures of treatment adherence and tolerability

	**Treatment arm**		
	**SP**	**DP**	**DP+SP**
At the level of each individual woman receiving at least one dose of study drugs			
At least one dose of study drug held for adverse event	n/N (%)	n/N (%)	n/N (%)
Missed at least 1 course of study drugs (all 3 doses)	n/N (%)	n/N (%)	n/N (%)
Reported not taking at least 1 dose of study drug at home	n/N (%)	n/N (%)	n/N (%)
At least one dose of study drug vomited	n/N (%)	n/N (%)	n/N (%)
At the level of each scheduled dose of study drug			
Study drugs (all 3 doses) held for adverse event	n/N (%)	n/N (%)	n/N (%)
Study drugs (all 3 doses) missed	n/N (%)	n/N (%)	n/N (%)
Vomited day 1 dose of study drugs (observed)	n/N (%)	n/N (%)	n/N (%)
Reported not taking day 2 dose of study drugs at home	n/N (%)	n/N (%)	n/N (%)
Reported vomiting day 2 dose of study drugs at home	n/N (%)	n/N (%)	n/N (%)
Reported not taking day 3 dose of study drugs at home	n/N (%)	n/N (%)	n/N (%)
Reported vomiting day 3 dose of study drugs at home	n/N (%)	n/N (%)	n/N (%)

7 Efficacy Analyses (Objectives 1 and 3)

7.1 Efficacy outcomes

Definitions and criteria used to generate estimates of all primary and secondary efficacy outcomes are presented in Table 8 below.

Table 8. Primary and secondary outcomes

Outcome	Category	Type of measurement	Timing of measurement	Numerator	Denominator	Missing data
Composite adverse birth outcome	Primary outcome objective 1	Proportion	At delivery and 28 days postpartum	Any of the following: spontaneous abortion, stillbirth, low birth weight, preterm delivery, small for gestational age, or neonatal death	All women who deliver	Withdrawn prior to delivery
Low birth weight	Secondary outcome objective 1	Proportion	At delivery	Birth weight < 2500 gm	Deliveries with a live birth	Withdrawn prior to delivery or deliveries with fetal loss
Preterm delivery	Secondary outcome objective 1	Proportion	At delivery	Gestational age < 37 weeks	Deliveries with a live birth	Withdrawn prior to delivery or deliveries with fetal loss
Small for gestational age	Secondary outcome objective 1	Proportion	At delivery	< 10th percentile based on INTERGROWTH-21st standards[17]	Deliveries with a live birth	Withdrawn prior to delivery or deliveries with fetal loss
Spontaneous abortion	Secondary outcome objective 1	Proportion	At delivery	Delivery at < 28 weeks gestational age	All deliveries	Withdrawn prior to delivery
Stillbirth	Secondary outcome objective 1	Proportion	At delivery	Infant born deceased	Deliveries ≥ 28 weeks gestational age	Withdrawn prior to delivery or < 28 weeks gestational age
Neonatal death	Secondary outcome objective 1	Proportion	28 days postpartum	Infant death in the first 28 days of life	Deliveries with a live birth	Withdrawn prior to delivery or deliveries with fetal loss
Birthweight	Secondary outcome objective 1	Mean, quantile	At delivery	Mean birthweight in grams among live births, differences in birthweight quantiles (i.e., 10th, 50th, and 90th percentile) among live births		Withdrawn prior to delivery or deliveries with fetal loss
Gestational age at delivery	Secondary outcome objective 1	Mean	At delivery	Mean gestational age in weeks among live births		Withdrawn prior to delivery or deliveries with fetal loss
Birthweight-for-gestational age Z-score	Secondary outcome objective 1	Mean	At delivery	Birthweight-for-gestational age z-scores among live births. Z-scores calculated based on INTERGROWTH-21st standards[17]		Withdrawn prior to delivery or deliveries with fetal loss
Composite of fetal loss and neonatal mortality	Secondary outcome objective 1	Proportion	At delivery and 28 days postpartum	Any of the following: spontaneous abortion, stillbirth, or neonatal death	All women who deliver	Withdrawn prior to delivery

Detection of malaria parasites at delivery	Primary outcome objective 3a	Proportion	At delivery	Any malaria parasites detected by microscopy or qPCR from maternal blood, placental blood, or placental tissue collected at delivery	Number of deliveries with samples collected	Withdrawn prior to delivery or no delivery samples collected
Detection of placental malaria	Primary outcome objective 3a	Proportion	At delivery	Any evidence of malaria parasites or pigment by placental histopathology	All women who deliver	Withdrawn prior to delivery or failure to collect samples for placental histopathology
Incidence of symptomatic malaria during pregnancy	Secondary outcome objective 3a	Incidence	Time at risk during pregnancy	Number of episodes of fever and positive blood smear by microscopy	Duration of observation from day following 1st dose of study drugs to delivery or premature study withdrawal	None
Prevalence of parasitemia during pregnancy	Secondary outcome objective 3a	Proportion	At the time of each routine visit	Maternal blood samples with parasites detected by microscopy or qPCR	Routine visits at 20, 24, 28, 32, 36, and 40 weeks of gestational age following 1st dose of study drugs to delivery or premature study withdrawal	Missed routine visits or samples not collected
Prevalence of congenital malaria infection	Secondary outcome objective 3a	Proportion	At the time of delivery	Presence of parasites detected by microscopy or PCR in fetal cord blood	All women who deliver	Withdrawn prior to delivery, fetal cord blood not collected,
Maternal anemia	Secondary outcome objective 3a	Proportion	At the time of each routine visit and at delivery when phlebotomy done	Hemoglobin level < 11 g/dL	Routine visits at 20,28, 36 weeks of gestational age following 1st dose of study drugs to delivery or premature study withdrawal	Missed routine visits or sample not collected when scheduled for phlebotomy
Prevalence of molecular markers of DP resistance	Secondary outcome objective 3a	Proportion	At the time of each routine visit or when malaria diagnosed	Detection of malaria parasites containing molecular markers of DP resistance	Detection of malaria parasites following 1st dose of study drugs	Samples where malaria parasites detected but assays for molecular markers of DP resistance failed
Prevalence of STIs/RTIs	Primary outcome objective 3b	Proportion	At the time of delivery	Women who deliver and test positive for the presence of STIs/RTIs	Women who deliver and successfully tested for the presence of STIs/RTIs	Failure to collect samples at delivery or failure to generate results
Changes in vaginal/gut microbiome	Secondary outcome objective 3b	Abundance of microorganisms	At enrollment and at 36 weeks gestational age	Changes in relative and absolute abundance of microorganisms		Failure to collect samples at enrollment and 36 weeks gestational age
Prevalence of molecular markers of SP resistance	Secondary outcome objective 3b	Proportion	At the time of each routine visit or when malaria diagnosed	Detection of malaria parasites containing molecular markers of SP resistance	Detection of malaria parasites following 1st dose of study drugs	Samples where malaria parasites detected but assays for molecular markers of SP resistance failed
Maternal MUAC at last clinic visit before delivery	Secondary outcome objective 3b	Mean	At enrollment and at last clinic visit before delivery	Mid-upper arm circumference (MUAC) values measured at last clinic visit before delivery; adjusted for MUAC values on the day study drugs first given among women followed at least until > 27 weeks gestational age (3rd trimester)		Failure to collect MUAC values on the day study drugs first given
Gestational weight gain per week	Secondary outcome objective 3b	Mean	At each clinic visit (from enrollment to delivery)	Maternal weight measured at the last clinic visit before delivery minus the maternal weight on the day study drugs first given, divided by the number of weeks between those dates; adjusted for weight on the day study drugs first given. Only include women followed at least until > 27 weeks gestational age (3rd trimester)		Failure to collect weight values on the day study drugs first given

7.2 Primary Efficacy Analysis

Objective 1

For objective 1, we will test the hypothesis that pregnant women who receive IPTp every 4 weeks with a combination of SP+DP will have a lower risk of our composite adverse birth outcome compared to those who receive either SP or DP alone.

Primary analysis. Using a modified intent-to-treat approach, we will compare the prevalence of our primary outcome between the study arms using unadjusted log-binomial models. Results will be presented as the risk ratio (RR) or reduction of the RR (1-RR x 100%) if the RR is lower than 1. We will explore for any differences of potential confounders between the treatment arms and if necessary adjust our analysis using multivariate log-binomial models. If the log-binomial model does not converge, modified Poisson regression with robust standard errors will be used to estimate RR.

Secondary analyses. For secondary binary outcomes, we will compare the prevalence of our individual adverse birth and neonatal outcomes using the same approach as our primary analysis. For comparing secondary continuous outcomes, linear regression will be used to compare differences between arms. Quantile regression may also be used if assumptions for linear regression cannot be met (e.g., linearity, homoscedasticity, independence, or normality) and to determine whether treatment arms were associated with nuanced changes in the lower and upper percentiles distribution of continuous outcomes (e.g., changes in the 10th or 90th percentile of birthweight). For outcomes that that were also measured at enrollment (e.g., fetal weight), enrollment measures will be included as a covariate in the model.

For subgroup analyses, we will include a two-way interaction term between treatment arm and subgroup of interest in our models. If the p-value of the interaction term is less than 0.1, we will consider this finding to be statistically significant. For all other p-values, values less than 0.05 will be considered statistically significant.

Objective 3

For objective 3a, we will test the hypotheses that pregnant women who receive IPTp regimens containing DP will have a lower risk of measures of malaria during pregnancy and at delivery but a higher risk of infection with malaria parasites containing mutations associated with DP resistance compared to those who receive SP alone. For objective 3b, we will test the hypotheses that pregnant women who receive IPTp regimens containing SP will have a lower risk of STIs/RTIs, greater changes in their vaginal/intestinal microbiota, improved maternal nutrition, and a higher risk of infection with malaria parasites containing mutations associated with SP resistance compared to those who receive DP alone.

Analysis. We will compare proportions between the study arms using log-binomial models and presented as the risk ratio (RR) or reduction of the RR (1-RR x 100%) if the RR is lower than 1. For repeated measures in the same study participant (parasite prevalence and anemia during pregnancy,

prevalence of molecular markers of drug resistance) we will use generalized estimating equations with a log-binomial model and robust standard errors. We will compare the incidence of malaria during pregnancy using negative binomial regression models. These models will include the logarithm of the follow-up time as an offset. We will translate the fitted coefficients and their confidence bounds into percentage effects with the formula 100*[exp(coefficient)-1]. This approach is closely related to exponential survival models for analyzing events per follow-up time, but is better able to adjust for violated assumptions. If necessary, multivariate analyses will be performed to adjust for potential confounders and effect modifiers. The main results will be based on the adjusted analyses if residual confounding is present. Comparisons of incidence measures will be expressed at the incidence rate ratio (IRR) or the protective efficacy (PE = 1-IRR x 100%). For continuous outcomes, either linear regression or quantile regression will be used to compare differences between arms, with a similar rationale to those stated in Objective 1. For outcomes that that were also measured at enrollment (e.g., fetal weight), enrollment measures will be included as a covariate in the model. Subgroup analyses will be conducted using the same approach as Objective 1.

Skeleton tables for the presentation of primary and secondary efficacy outcomes are presented in Tables 9-11 below.

Table 9. Composite primary outcome and its components assessed at the time of delivery

Outcome	Treatment arm						
	SP [a]	DP			DP+SP		
	Prevalence	Prevalence	RR (95% CI)	p-value	Prevalence	RR (95% CI)	p-value
Composite adverse birth outcome	n/N (%)	n/N (%)			n/N (%)		
Individual birth outcomes							
Spontaneous abortion	n/N (%)	n/N (%)			n/N (%)		
Stillbirth	n/N (%)	n/N (%)			n/N (%)		
Low birth weight	n/N (%)	n/N (%)			n/N (%)		
Preterm delivery	n/N (%)	n/N (%)			n/N (%)		
Small for gestational age	n/N (%)	n/N (%)			n/N (%)		
Neonatal death	n/N (%)	n/N (%)			n/N (%)		
Fetal or neonatal loss	n/N (%)	n/N (%)			n/N (%)		

[a] Reference group

Table 10. Secondary efficacy outcomes assessed at the time of delivery

Outcome	Treatment arm						
	SP[a]	DP			SP+DP		
	Mean (SD)	Mean (SD)	MD (95% CI)	p-value	Mean (SD)	MD (95% CI)	p-value
Birthweight in grams	XX (XX)	XX (XX)			XX (XX)		
Gestational age at delivery in weeks	XX (XX)	XX (XX)			XX (XX)		
Birthweight-for-gestational age z-score	XX (XX)	XX (XX)			XX (XX)		
Maternal MUAC in cm[b]	XX (XX)	XX (XX)			XX (XX)		
	Prevalence	Prevalence	RR (95% CI)	p-value	Prevalence	RR (95% CI)	p-value
Detection of malaria parasites at delivery	n/N (%)	n/N (%)			n/N (%)		
Detection of placental malaria	n/N (%)	n/N (%)			n/N (%)		
Detection of congenital malaria infection	n/N (%)	n/N (%)			n/N (%)		
Prevalence of STIs/RTIs at delivery	n/N (%)	n/N (%)			n/N (%)		

[a] Reference group

[b] Adjusted for baseline values

Table 11. Longitudinal secondary outcomes assessed during pregnancy

Outcome	Treatment arm						
	SP [a]	DP			SP+DP		
**Incidence measures**	**Events[b]**	**Events[b]**	**IRR (95% CI)**	**p-value**	**Events[b]**	**IRR (95% CI)**	**p-value**
Symptomatic malaria	xx (x.xx)	xx (x.xx)			xx (x.xx)		
**Prevalence measures**	**Prevalence**	**Prevalence**	**RR (95% CI)**	**p-value**	**Prevalence**	**RR (95% CI)**	**p-value**
Detection of malaria parasites by microscopy or qPCR							
All routine visits	n/N (%)	n/N (%)			n/N (%)		
20 weeks gestational age	n/N (%)	n/N (%)			n/N (%)		
24 weeks gestational age	n/N (%)	n/N (%)			n/N (%)		
28 weeks gestational age	n/N (%)	n/N (%)			n/N (%)		
32 weeks gestational age	n/N (%)	n/N (%)			n/N (%)		
36 weeks gestational age	n/N (%)	n/N (%)			n/N (%)		
40 weeks gestational age	n/N (%)	n/N (%)			n/N (%)		
Anemia defined as hemoglobin level < 11 g/dL							
All routine visits and delivery	n/N (%)	n/N (%)			n/N (%)		
20 weeks gestational age	n/N (%)	n/N (%)			n/N (%)		
28 weeks gestational age	n/N (%)	n/N (%)			n/N (%)		
36 weeks gestational age	n/N (%)	n/N (%)			n/N (%)		
At the time of delivery	n/N (%)	n/N (%)			n/N (%)		
Prevalence of molecular markers of DP resistance	n/N (%)	n/N (%)			n/N (%)		
Prevalence of molecular markers of SP resistance	n/N (%)	n/N (%)			n/N (%)		
**Changes in measures of abundance**	**Mean Δ (SD)**	**Mean Δ (SD)**		**p-value**	**Mean Δ (SD)**		**p-value**
Changes in vaginal/gut microbiome	xx (x.xx)	xx (x.xx)			xx (x.xx)		
Gestational weight gain per week (kg)[c]	xx (x.xx)	xx (x.xx)			xx (x.xx)		

[a] Reference group
[b] Number of events (incidence per person year at risk)
[c] Adjusted for baseline values

8 Safety and Tolerability Analyses (Objective 2)

Safety and tolerability will be evaluated during the period following the 1st dose of study drug administration through the end of the observation period (4 weeks post-partum) or premature study withdrawal.

8.1 Adverse Events

An adverse event will be defined as "any untoward medical occurrence in a patient or clinical investigation subject administered a pharmaceutical product that does not necessarily have a causal relationship with this treatment" (ICH Guidelines E2A). An adverse event can further be broadly defined as any untoward deviation from baseline health, which includes:

- Worsening of conditions present at the onset of the study
- Deterioration due to the primary disease
- Intercurrent illness
- Events related or possibly related to concomitant medications

(International Centers for Tropical Disease Research Network Investigator Manual, Monitoring and Reporting Adverse Events, 2003).

At each scheduled and unscheduled visit to the clinic, study clinicians will assess patients according to a standardized case record form. A severity grading scale, based on toxicity grading scales developed by the NIH Divisions of AIDS (DAIDS) and the Division of Microbiology and Infectious Diseases (DMID) Pediatric Toxicity Tables, will be used to grade severity of all symptoms, physical exam findings, and laboratory results. All participants, regardless of treatment arm, will be assessed using the same standardized case record form. Adverse event monitoring will occur during the period when study drugs are given and up to 4 weeks following delivery. Data will be captured on the incidence of all adverse events, regardless of severity. For each adverse event identified as severity grade 3-4 or a serious adverse event (SAE), an additional adverse event report form will be completed.

8.2 Serious Adverse Events

A Serious Adverse Event (SAE) will be defined as any adverse event that results in any of the following outcomes:

- Death
- Life-threatening adverse experience
- Inpatient hospitalization or prolongation of existing hospitalization
- Persistent or significant disability/incapacity
- Congenital malformation/birth defect
- Any other experience that suggests a significant hazard, contraindication, side effect or precaution that may require medical or surgical intervention to prevent one of the outcomes listed above
- Event that changes the risk/benefit ratio of the study

8.3 Analytical Methods

A modified intention-to-treat approach to all will be used, including all study participants who received at least one dose of study drugs, regardless of whether subsequently the intervention was not given for any reason. We will compare the proportions of study participants with vomiting following each dose of study drugs using generalized estimating equations with a log-binomial family and robust standard errors to account for repeated measures in the same study participant. We will compare the incidence of various adverse events using negative binomial regression models. The These models will include the logarithm of the follow-up time as an offset. We will translate the fitted coefficients and their confidence bounds into percentage effects with the formula 100*[exp(coefficient)-1]. This approach is closely related to exponential survival models for analyzing events per follow-up time, but is better able to adjust for violated assumptions. Comparisons of incidence measures will be expressed at the incidence rate ratio (IRR) or the protective efficacy (PE = 1-IRR x 100%). A skeleton table for the presentation of safety and tolerability outcomes is presented in Table 11 below.

Table 11. Measures of safety and tolerability

Outcome	Treatment arm						
	SP [a]	DP			SP+DP		
**Prevalence measures**	**Prevalence**	**Prevalence**	**RR (95% CI)**	**p-value**	**Prevalence**	**RR (95% CI)**	**p-value**
Vomiting following administration of study drugs							
Observed after administration of 1st dose in clinic	n/N (%)	n/N (%)					
Reported after administration of 2nd dose at home	n/N (%)	n/N (%)					
Reported after administration of 3rd dose at home	n/N (%)	n/N (%)					
**Incidence measures**	**Events [b]**	**Events [b]**	**IRR (95% CI)**	**p-value**	**Events [b]**	**IRR (95% CI)**	**p-value**
Individual adverse events of any severity [c]							
XXXX	xx (x.xx)	xx (x.xx)					
XXXX	xx (x.xx)	xx (x.xx)					
XXXX	xx (x.xx)	xx (x.xx)					
XXXX	xx (x.xx)	xx (x.xx)					
XXXX	xx (x.xx)	xx (x.xx)					
Individual grade 3-4 adverse events [c]							
XXXX	xx (x.xx)	xx (x.xx)					
XXXX	xx (x.xx)	xx (x.xx)					
XXXX	xx (x.xx)	xx (x.xx)					
XXXX	xx (x.xx)	xx (x.xx)					
XXXX	xx (x.xx)	xx (x.xx)					
All grade 3-4 adverse events	xx (x.xx)	xx (x.xx)					
Grade 3-4 adverse events possibly related to study drugs	xx (x.xx)	xx (x.xx)					
All serious adverse events	xx (x.xx)	xx (x.xx)					

[a] Reference group
[b] Number of events (incidence per person year at risk)
[c] Includes only those categories with at least five total events

9 References

1. Dellicour S, Tatem AJ, Guerra CA, Snow RW, ter Kuile FO. Quantifying the number of pregnancies at risk of malaria in 2007: a demographic study. PLoS Med 2010;7(1):e1000221. DOI: 10.1371/journal.pmed.1000221.
2. Desai M, ter Kuile FO, Nosten F, et al. Epidemiology and burden of malaria in pregnancy. Lancet Infect Dis 2007;7(2):93-104. (In eng). DOI: S1473-3099(07)70021-X [pii]

10.1016/S1473-3099(07)70021-X.
3. Guyatt HL, Snow RW. Malaria in pregnancy as an indirect cause of infant mortality in sub-Saharan Africa. Trans R Soc Trop Med Hyg 2001;95(6):569-76. (https://www.ncbi.nlm.nih.gov/pubmed/11816423).
4. Walker PG, ter Kuile FO, Garske T, Menendez C, Ghani AC. Estimated risk of placental infection and low birthweight attributable to Plasmodium falciparum malaria in Africa in 2010: a modelling study. The Lancet Global health 2014;2(8):e460-7. DOI: 10.1016/S2214-109X(14)70256-6.
5. Gutman J, Kalilani L, Taylor S, et al. The A581G Mutation in the Gene Encoding Plasmodium falciparum Dihydropteroate Synthetase Reduces the Effectiveness of Sulfadoxine-Pyrimethamine Preventive Therapy in Malawian Pregnant Women. J Infect Dis 2015;211(12):1997-2005. DOI: 10.1093/infdis/jiu836.
6. Ranson H, N'Guessan R, Lines J, Moiroux N, Nkuni Z, Corbel V. Pyrethroid resistance in African anopheline mosquitoes: what are the implications for malaria control? Trends Parasitol 2011;27(2):91-8. DOI: 10.1016/j.pt.2010.08.004.
7. A head-to-head comparison of four artemisinin-based combinations for treating uncomplicated malaria in African children: a randomized trial. PLoS medicine 2011;8(11):e1001119. (Comparative Study

Multicenter Study

Randomized Controlled Trial

Research Support, Non-U.S. Gov't) (In eng). DOI: 10.1371/journal.pmed.1001119.
8. White NJ. Intermittent presumptive treatment for malaria. PLoS Med 2005;2(1):e3. (In eng) (http://www.ncbi.nlm.nih.gov/entrez/query.fcgi?cmd=Retrieve&db=PubMed&dopt=Citation&list_uids=15696210).
9. Desai M, Gutman J, L'Lanziva A, et al. Intermittent screening and treatment or intermittent preventive treatment with dihydroartemisinin-piperaquine versus intermittent preventive treatment with sulfadoxine-pyrimethamine for the control of malaria during pregnancy in western Kenya: an open-label,

three-group, randomised controlled superiority trial. Lancet 2015;386(10012):2507-19. DOI: 10.1016/S0140-6736(15)00310-4.
10. Kajubi R, Ochieng T, Kakuru A, et al. Monthly sulfadoxine-pyrimethamine versus dihydroartemisinin-piperaquine for intermittent preventive treatment of malaria in pregnancy: A randomized controlled trial. The Lancet 2019.
11. Kakuru A, Jagannathan P, Muhindo MK, et al. Dihydroartemisinin-Piperaquine for the Prevention of Malaria in Pregnancy. N Engl J Med 2016;374(10):928-39. DOI: 10.1056/NEJMoa1509150.
12. Committee WHOMPA, Secretariat. Malaria Policy Advisory Committee to the WHO: conclusions and recommendations of eighth biannual meeting (September 2015). Malar J 2016;15:117. DOI: 10.1186/s12936-016-1169-x.
13. Chico RM, Moss WJ. Prevention of malaria in pregnancy: a fork in the road? Lancet 2015;386(10012):2454-6. DOI: 10.1016/S0140-6736(15)00325-6.
14. Chico RM, Chaponda EB, Ariti C, Chandramohan D. Sulfadoxine-Pyrimethamine Exhibits Dose-Response Protection Against Adverse Birth Outcomes Related to Malaria and Sexually Transmitted and Reproductive Tract Infections. Clin Infect Dis 2017;64(8):1043-1051. DOI: 10.1093/cid/cix026.
15. ter Kuile FO, van Eijk AM, Filler SJ. Effect of sulfadoxine-pyrimethamine resistance on the efficacy of intermittent preventive therapy for malaria control during pregnancy: a systematic review. Jama 2007;297(23):2603-16. (In eng) (http://www.ncbi.nlm.nih.gov/entrez/query.fcgi?cmd=Retrieve&db=PubMed&dopt=Citation&list_uids=17579229).
16. Schmiegelow C, Scheike T, Oesterholt M, et al. Development of a fetal weight chart using serial trans-abdominal ultrasound in an East African population: a longitudinal observational study. PLoS ONE 2012;7(9):e44773. DOI: 10.1371/journal.pone.0044773.
17. Villar J, Ismail LC, Victora CG, et al. International standards for newborn weight, length, and head circumference by gestational age and sex: the Newborn Cross-Sectional Study of the INTERGROWTH-21st Project. The Lancet 2014;384(9946):857-868.
18. Services DoANIoAaIDNIoHUDoHaH. Division of AIDS (DAIDS) Table for Grading the Severity of Adult and Pediatric Adverse Events. (https://rsc.tech-res.com/docs/default-source/safety/daids_ae_grading_table_v2_nov2014.pdf).
